# Morphological Innovations and Vast Extensions of Mountain Habitats Triggered Rapid Diversification Within the Species-Rich Irano-Turanian Genus *Acantholimon* (Plumbaginaceae)

**DOI:** 10.3389/fgene.2018.00698

**Published:** 2019-01-21

**Authors:** Farideh Moharrek, Isabel Sanmartín, Shahrokh Kazempour-Osaloo, Gonzalo Nieto Feliner

**Affiliations:** ^1^Department of Plant Biology, Faculty of Biological Sciences, Tarbiat Modares University (TMU), Tehran, Iran; ^2^Department of Biodiversity and Conservation, Real Jardín Botánico (CSIC), Madrid, Spain

**Keywords:** *Acantholimon*, allopatric speciation, ancestral area reconstruction, extinction, Irano-Turanian, key innovations, mountain habitats, rapid diversification

## Abstract

The Irano-Turanian floristic region spans a topographically complex and climatically continental territory, which has served as a source of xerophytic taxa for neighboring regions and is represented by a high percent of endemics. Yet, a comprehensive picture of the abiotic and biotic factors that have driven diversification within this biota remains to be established due to the scarcity of phylogenetic studies. *Acantholimon* is an important component of the subalpine steppe flora of the Irano-Turanian region, containing c. 200 cushion-forming sub-shrubby pungent-leaved species. Our recent molecular phylogenetic study has led to enlarging the circumscription of this genus to include eight mono- or oligospecific genera lacking the characteristic life-form and leaves. Using the same molecular phylogeny, here we investigate the tempo and mode of diversification as well as the biogeographic patterns in this genus, to test the hypothesis that a combination of key morphological innovations and abiotic factors is behind *Acantholimon* high species diversity. Molecular dating analysis indicates that *Acantholimon* s.l. started to diversify between the Late Miocene and the Pliocene and the biogeographic analysis points to an Eastern Iran–Afghanistan origin. Macroevolutionary models support the hypothesis that the high diversity of the genus is explained by accelerated diversification rates in two clades associated with the appearance of morphological key innovations such as a cushion life-form and pungent leaves; this would have favored the colonization of water-stressed, substrate-poor mountainous habitats along the newly uplifted IT mountains during the Mio-Pliocene. Given the apparent similarity of mountain habitats for most species of *Acantholimon*, we hypothesize that its current high species diversity responds to a scenario of non-adaptive radiation fueled by allopatric speciation rather than evolutionary radiation driven by ecological opportunity. Similar scenarios might underlie the high diversity of other speciose genera in the topographically complex Irano-Turanian landscape, though this remains to be tested with fine-grained distribution and climatic data.

## Introduction

The disparity of organismal diversification rates (speciation minus extinction) and its causes are a hot topic in evolutionary biology (Ricklefs, 2006; Linder, 2008; Losos, 2010; Morlon et al., 2011; Pyron and Burbrink, 2013; Stadler, 2013; Morlon, 2014; Alexander et al., 2016; López-Estrada et al., 2018). Variations in the rates of speciation and extinction over time and across lineages and the factors underlying those changes are key to understanding how diversity is generated, which in turn is instrumental in designing how it can be preserved (Linder, 2005; Condamine and Hines, 2015; Sanmartín and Meseguer, 2016). Molecular phylogenies of species-rich groups are suitable models for investigating the tempo, rate and mode of diversification (Nee et al., 1992; Paradis, 1997; Pybus and Harvey, 2000; Harmon et al., 2003), and for inferring the spatial component of diversification by testing hypotheses about biogeography and migration patterns (Moore and Donoghue, 2007; Antonelli and Sanmartín, 2011; Condamine et al., 2012).

Evolutionary radiations are usually defined as involving rapid cladogenesis from a common ancestor and yielding taxon rich clades (Simpson, 1953; Ober and Heider, 2010). Most studies on radiations have focused on adaptive radiation, defined as “the evolution of ecological and phenotypic diversity within a rapidly multiplying lineage” (Schluter, 2000), a process in which ecological opportunity and key innovations are important features (Givnish, 2010; Yoder et al., 2010). While there is both empirical (Seehausen, 2006; Fior et al., 2013; Joly et al., 2013; Lagomarsino et al., 2016) and theoretical (Gavrilets and Vose, 2005) support for the occurrence of this model, which elements are central to the hypothesis of adaptive radiation remain controversial (Glor, 2010; Losos and Mahler, 2010; Givnish, 2015). For instance, in line with the view that adaptive radiation is distinct from ecological speciation, some models suggest that speciation during adaptive radiation is largely non-ecological and involves processes other than niche evolution (Grant and Grant, 2008; Rundell and Price, 2009; Glor, 2010). Other views question the focus on extraordinary diversification (Glor, 2010; Givnish, 2015), connecting with the old idea that adaptive radiation in a wider sense may be the predominant mode of biological diversification (Simpson, 1953; Stebbins, 1974). In addition, others openly advocate the role than non-adaptive radiation, i.e., involving niche conservatism, may play in radiative processes (Gittenberger, 1991; Kozak et al., 2006). Such alternative models and views on how radiations may be generated suggest that theoretical preconceptions should be minimized when examining new empirical data that involve rapid diversification in species-rich groups.

In flowering plants, there has been substantial research into the morphological, ecological (environmental), and physiological correlates of cladogenesis (Ree, 2005; Hughes and Eastwood, 2006; Lagomarsino et al., 2016; Meseguer et al., 2018). Whether abiotic factors such as climate and geography or the appearance of morphological evolutionary novelties are behind changes in a lineage's diversification trajectory is the subject of a rich recent literature (Donoghue and Edwards, 2014; Donoghue and Sanderson, 2015). Causality in correlations between key innovations and changes in diversification rates are difficult to demonstrate because evolution rarely affects a separate evolutionary trait (Donoghue and Sanderson, 2015), and often these changes appear only once in the phylogeny, preventing adequate statistical testing (Maddison and FitzJohn, 2015). Yet, the causal link between key innovation and rapid diversification is one of the major appeals of studies of radiations.

Covering one third of the surface of Eurasia, the Irano-Turanian (hereafter, IT) region is one of the largest floristic regions in the world (Takhtajan, 1986) but its limits have been debated and recently reviewed in Manafzadeh et al. (2017). It encompasses a vast topographically complex and climatically very continental territory (Djamali et al., 2012a) that includes two hotspots sensu Mittermeier et al. (2005), the Irano-Anatolian and the Mountains of Central Asia, as well as the southern and eastern parts of a third one, the Caucasus. Overall, the IT region extends from the Anatolian Plateau (excluding the southeastern Mediterranean fringe) and Levant region (up to northern part of the Sinai peninsula) on the west to the western China provinces of Gansu and western Sichuan on the east; and from southeastern Russia, Kazakhstan and Southern Mongolia on the north to the Iranian plateau (except the Iranian Gulf and Gulf of Oman), Afghanistan and the western Himalayas on the south. The IT biota, represented by a high percent of endemics, has also served as a source of xerophytic taxa for neighboring regions, in particular the Mediterranean region (Blondel et al., 2010; Manafzadeh et al., 2017), via migration corridors during dry climate episodes. Its dynamic geological history, resulting from the tectonic collision of three major plates, Eurasia, Arabia, and India, offers good opportunities for studying patterns of episodic biotic exchange between independently evolving biotas. Yet, the IT region has been scarcely studied from a biogeographical and in general an evolutionary point of view (Manafzadeh et al., 2014).

A key step in explaining the high botanical diversity of IT hotspots is demonstrating whether such diversity is the result of a rapid diversification compared to neighboring regions, and, if so, whether this is the result of a higher rate of speciation or a lower rate of extinction. The recent origin and radiation of IT species-rich groups, such as *Cousinia* Cass. subgen. *Cousinia* (c. 8.7 Mya; López-Vinyallonga et al., 2009) and *Acanthophyllum* C.A.Mey. s.l. (c. 11.1 Mya; Pirani et al., 2014), lends support to the idea of high diversification rates in the IT region, though old lineages with slower diversification rates also occur in this region, e.g., *Haplophyllum* A. Juss. (Manafzadeh et al., 2014). Yet, explicit comparisons of diversification rates between groups within and outside IT hotspots remain rare.

The IT region is characterized by a dynamic geological history, with several active tectonic lines, ongoing plate subduction (e.g., the African Plate beneath the Anatolian Plate), and several orogenic uplift events (e.g., Tibet, Caucasus). This has probably resulted in numerous opportunities for species formation, through geographic isolation (allopatry), colonization of novel environmental niches (mountain peaks and slopes), or adaptation to a more continental climate.

*Acantholimon* Boiss. is one of the richest angiosperm plant genera in the IT region and the second most species-rich genus in the Plumbaginaceae, with c. 200 species, most of which are geographically restricted (Mobayen, 1964; Kubitzki, 1993). All the species occur in the IT region but < 5% of them also occur in the Mediterranean region (Bunge, 1872; Mobayen, 1964; Linczevski, 1967; Kubitzki, 1993; Peng and Kamelin, 1996; Assadi, 2005, 2006; Dogan and Akaydin, 2007; Dogan et al., 2011). *Acantholimon* species have been traditionally recognized by their pulvinate (cushion forming) to densely-branched cespitose pungent subshrubby life-form. They occur in mountainous regions, mostly at mid- and higher elevations, growing in gravelly and stony soils or on exposed rocks. A recent molecular phylogenetic study, including c. two-thirds of the species diversity, has revealed that *Acantholimon* as currently defined is not monophyletic, and supports a widely circumscribed *Acantholimon*, with the small genus *Goniolimon* Boiss. (c. 20 species) as its sister group (Moharrek et al., 2017). This new definition of the genus—which we refer below as *Acantholimon* s.l.—includes the species-poor genera *Bamiania* Lincz., *Bukiniczia* Lincz., *Chaetolimon* (Bunge) Lincz., *Cephalorhizum* Popov & Korovin, *Dictyolimon* Rech.f., *Gladiolimon* Mobayen, *Popoviolimon* Lincz., and *Vassilczenkoa* Lincz., which differ markedly from *Acantholimon* s.str. in their overall morphology, in particular life-form and leaves. Moharrek et al. (2017) placed these genera, except for *Gladiolimon*, as early diverging lineages relative to two different clades that contained the species traditionally included within *Acantholimon*, hereafter *Acantholimon* s.str. Ancestral character state reconstruction of morphological traits revealed that the cushion life-form and pungent leaves, characteristic of *Acantholimon* (it is also present in the IT genus *Acanthophyllum*, Pirani et al., 2014), evolved independently in the two main *Acantholimon* s.str. clades (Moharrek et al., 2017). These authors suggested that the current high diversity of *Acantholimon* s.l. could be related to the evolution of these key innovations and driven by orography and climate change in the IT region. Yet, the absence of an explicit spatiotemporal framework prevented the statistical testing of this hypothesis. Because of its large species-richness in the IT region, compared to its sister genus *Goniolimon*, and the inclusion of species-poor and rich lineages, *Acantholimon* represents an ideal model system for testing the potential correlation between changes in diversification rates, morphological key innovations, and ecological opportunity in driving the high diversity of the IT region.

Here, we used the phylogenetic hypothesis of *Acantholimon* s.l. in Moharrek et al. (2017), c. 65% of all species, to investigate the tempo and mode of diversification and reconstruct the spatiotemporal evolution of this genus. Specifically, we aimed to test the hypothesis that key morphological innovations in life form are behind *Acantholimon s.l*.'s current high species diversity, and to explore the role that biogeographic events such as the colonization of new areas and abiotic factors (e.g., climate change toward aridification and continentality) played in the rapid diversification of this genus. For this, we (1) estimated the age of divergence of major lineages within *Acantholimon* using relaxed molecular clocks; (2) inferred point changes in diversification rates along the evolutionary history of *Acantholimon* over time and across clades; (3) tested for a causal relationship between transitions in life-form and leaf morphology and changes in speciation and extinction rates; and (4) reconstructed the biogeographic pathways by which this genus reached its current range. By reconstructing evolutionary patterns in the second largest genus of Plumbaginaceae, we also provide new insights on the evolution of this family.

## Materials and Methods

### Taxon Sampling

For the reconstruction of the temporal evolution and biogeographic patterns of the study group, we used a simplified dataset of that in Moharrek et al. (2017). The original was a combined dataset of nuclear ribosomal DNA ITS and plastid *trn*Y-T sequences. Since no incongruence was found between the two markers, subsequent analyses were based on the concatenated nuclear-plastid matrix (2,021 bp). This dataset was trimmed to include a single accession per species covering 130 species of *Acantholimon*, as the ingroup, and 26 species, belonging to subfamilies Limonioideae and Plumbaginoideae, as the outgroup. The sampling encompasses the major areas of occurrence of *Acantholimon* in the IT region, including its main center of diversity in Iran and Afghanistan, as well as Anatolia, the southern Caucasus, Central Asia, and Pakistan. All samples except that of *A. ulicinum* from Greece lie within the limits of the IT region. Voucher information and GenBank accession numbers are shown in Data Sheet S1.

### Divergence Time Estimation

Divergence times were inferred with BEAST v1.8.0 (Drummond et al., 2012a), ran at the CIPRES Science Gateway (http://www.phylo.org/; Miller et al., 2010) assuming a Bayesian relaxed molecular clock. Molecular rates were allowed to vary among lineages around an average value, by enforcing an uncorrelated lognormal clock of evolutionary rates. A birth–death branch process prior was used in the analysis. A general time reversible model with rate variation across sites, modeled using a gamma distribution and invariant proportion of sites (GTR + G + I), was applied to the concatenated data matrix.

**Figure 1:**
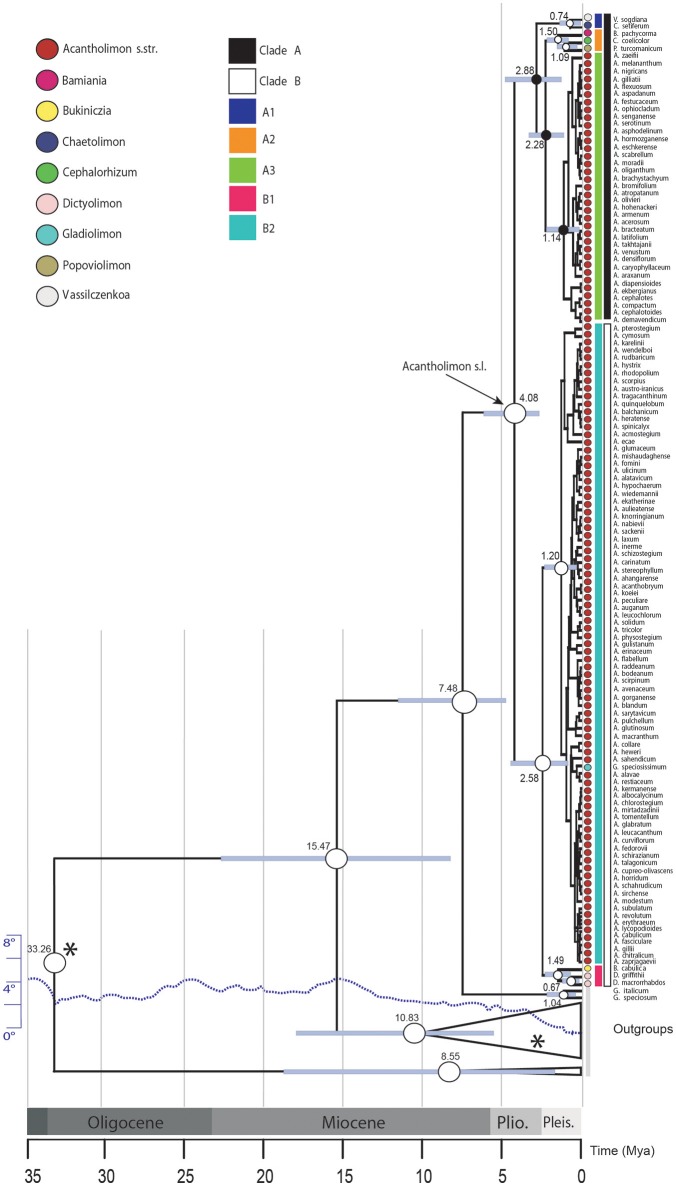
Bayesian inference analysis of *Acantholimon* s.l. based on two DNA regions (ITS and *trn*Y-T). Chronogram represents the maximum clade credibility tree estimated in BEAST, with mean divergence dates in million years ago (Mya) shown for key nodes, some of which are labeled following Moharrek et al. (2017). Colored circles label samples according to formerly recognized genera as well as *Acantholimon* traditional circumscription. White dots denote strongly supported nodes (≥95% posterior probability, PP); black dots, intermediately supported nodes (85–94% PP); gray bars represent 95% highest posterior density credibility intervals for node ages. Asterisks indicate nodes used as calibration points. Dotted line, scaled on the left, indicates change of average global deep ocean temperature over time based on benthic foraminiferal oxygen-isotope records corrected to account for ice-sheet accumulation according to Hansen et al. (2008).

There are no known macrofossils in Plumbaginaceae, which is mainly composed of herbs and subshrubs, but there is a pollen record. Pollen of *Limonium* Mill.*, Armeria* Willd.*, Goniolimon, Acantholimon, Psylliostachys* (Jaub. & Spach) Nevski, and most likely the other genera in the Limonioideae, are virtually indistinguishable. They consist of two morphs associated to an incompatibility system (Baker, 1948; Bokhari, 1972; Weber, 1981; Weber-El-Ghobary, 1987; Morretti et al., 2015) and are usually reported in palynological papers indistinctly either as *Limonium* or *Armeria* type. Using the available fossil pollen from the Upper Miocene (van Campo, 1976; Muller, 1981), Upper-Middle Miocene (Rivas-Carballo et al., 1994) or Middle Miocene (Valle et al., 1995) is thus problematic. Therefore, we calibrated our dating analysis with age estimates from a recently published time tree of angiosperms calibrated with 171 macrofossils (Magallón et al., 2015). Specifically, we calibrated the root node of our tree, the crown age of the family, using a normal distribution prior with median = 41.67 Mya and standard deviation (*SD*) = 10.8 Mya that covers the 95% high posterior density (HPD) credibility interval for that node in the original study (S. Magallón, pers. comm.). However, because the use of secondary calibrations has been recently criticized (Schenk, 2016), we used the Limonioideae pollen record as an additional calibration point, after performing a sensitivity analysis to explore the influence of the assignment of this fossil pollen to different nodes in our tree. Focusing conservatively on the Upper Miocene records only, we assigned the *Limonium* or *Armeria* type pollen record to the following nodes, in four alternative analyses (using exclusively this calibration): (1) the most-recent-common-ancestor (MRCA) of all *Armeria, Psylliostachys*, and *Limonium* species in our analysis, (2) the MRCA of *Armeria* and *Psylliostachys* species, (3) the MRCA of all *Armeria* species, and (4) the MRCA of all *Limonium* species in our analysis. These nodes were assigned a lognormal distribution prior with offset = 5.33 Mya, and *SD* = 1.15 Mya, covering the entire Upper Miocene geological interval (5.333–11.62 Mya). In doing this, we accounted for the possibility that the pollen belonged to either genus *Armeria* or *Limonium*, since these two genera are well-represented in the Iberian Peninsula where the original fossils were collected, or to a common ancestor of these two genera. Since the results of the analyses using the fossil pollen calibration point applied in four different positions were within the 95% HPD of each other (Table S1), we assigned the Limonioideae pollen record to the *Armeria* stem-node. We then performed the final dating analysis using two calibration points: the secondary calibration for the Plumbaginaceae crown-node and the fossil calibration for *Armeria* stem-node in our analysis (Data Sheet S2). The resulting chronogram (Figure 1) was used for the subsequent diversification and biogeographic analyses.

The MCMC chain was run in two separate analyses for 50 million generations sampling every 1000th generation. LogCombiner v1.7.5 (Drummond et al., 2012a) was used to combine the log and tree files after discarding the initial 5 million iterations of each analysis as burn-in. Tracer v1.6 (Rambaut et al., 2014) was used to assess that Effective Sample Sizes (ESS) were above 200 for optimal convergence and tree likelihood stationarity. Tracer automatically removed the first 10% of the log statistics from the combined sample, so we correspondingly discarded the first 10% of trees. A maximum clade credibility (MCC) tree was constructed in TreeAnnotator v1.7.5 (Drummond et al., 2012a) depicting the maximum sum of posterior clade probabilities (nexus file in Data Sheet S3). The MCC Tree was visualized in FigTree v1.4.1 (Drummond et al., 2012a).

### Diversification Analyses

We used macroevolutionary methods implemented in R (R Development Core Team, 2018) to evaluate potential factors underlying diversification in *Acantholimon*. All analyses were run on the BEAST MCC tree pruned to leave only the 130 terminal taxa belonging to *Acantholimon* s.l., to avoid biases related to incomplete taxon sampling in the outgroup. We first visualized variation in diversification rates over time by plotting a semilogarithm lineage-through-time (LTT; Nee et al., 1992) plot with the R package *ape* (Paradis et al., 2004) using both the MCC tree and a random sample of 1,000 trees from the BEAST MCMC posterior distribution to account for dating uncertainty. Second, we used the whole-tree maximum likelihood methods implemented in the R package *TreePar* (Stadler, 2011) to detect the signal of mass extinctions and temporal shifts in the diversification rate using the MCC tree as phylogenetic hypothesis. The function “bd.shifts.optim” with the option ME = FALSE was used to estimate the time and magnitude of changes in the rate of diversification (*r* = speciation minus extinction) and background extinction (ε = extinction/speciation) at discrete points in time. Sampling fraction at present was set to rho = 0.65 to reflect incomplete taxon sampling, and we estimated potential rate shifts in an interval grid of every 0.1 Mya, using the option posdiv = FALSE to allow for negative diversification rates (periods of declining diversity). We compared a constant birth-death model against models with an increasing number of rate shifts by using likelihood ratio tests (LRTs) at a significant alpha level of 0.05 (Stadler, 2011). We also run analyses with the ME option set to TRUE to detect mass extinction events (MEEs), i.e., tree-wide sampling events in which a percentage of extant diversity is instantaneously removed at a point in time. This option assumes *r* and ε to be constant before and after the MEE but allows estimating the magnitude of the MEEs. Though in theory it is possible to estimate simultaneously tree-wide rate shifts and MEEs, in practice this leads to problems of parameter non-identifiability, since these two types of events are modeled identically in the likelihood BD framework (Stadler, 2011). We tested models with increasing number of MEEs via LRTs.

Third, we used Bayesian Analysis of Macroevolutionary Mixture (BAMM v2.5, Rabosky, 2014) to detect heterogeneity in evolutionary rates across lineages within the phylogeny of *Acantholimon* s.l. This method employs MCMC and Bayes Factor comparisons and Compound Poisson Process (CPP) models to explore alternative diversification dynamic regimes, allowing for both rate-heterogeneity across lineages and variation in speciation rates over time. To account for incomplete taxon sampling, we specified a global sampling probability of 0.65. The R package BAMMtools v2.1 (Rabosky et al., 2014) was used to choose appropriate prior distributions for all parameters in the analysis, and to analyze and visualize the output, using 15% of samples as burn-in. BAMM has been questioned, in particular its initial versions, because of the unrealistic treatment of extinction in unobserved (non-sampled or extinct) lineages and flawed CPP priors (Moore et al., 2016). The first one was suggested to be a minor bias for most datasets based on simulations (Rabosky et al., 2017), but its effect on empirical cases remains to be tested. Sensitivity to the choice of the CPP prior, which informs the expected number of rate shifts, was assessed by performing multiple BAMM analyses with varying CPP priors (1, 5, and 10). We ran each analysis for 5 million generations, sampling every 5,000th generation. Since the selected CPP prior had little effect on our results, we ran the final analysis with the default prior in BAMMtools (a prior rate shift of 1) and four independent chains of 10 million generations each, sampled every 10,000th generation.

### Trait-Dependent Diversification Analyses

We tested for the effect of life-form and leaf type on diversification rates using the binary-state speciation and extinction (BiSSE) algorithm (Maddison et al., 2007) implemented in the DIVERSITREE R package (FitzJohn et al., 2009; FitzJohn, 2012). Coding of morphological states for each species was based on Moharrek et al. (2017), who used the taxonomic literature and direct observations to distinguish between two predominant overall morphologies or “syndromes” within *Acantholimon* s.l., defined mostly on the basis of life-form and leaves. The “*Acantholimon* syndrome” consists of a pulvinate to densely branched caespitose subshrub bearing mostly spike-like or capitate inflorescences, with linear rigid acuminate leaves, and occurs in *Acantholimon* s.str. and *Gladiolimon*. The “*Limonium* syndrome” is a perennial herb with thick rootstock, rosulate, spathulate, and slightly fleshy basal leaves, and flowering stems bearing paniculate inflorescences. This syndrome occurs in the species-poor *Bamiania, Bukiniczia, Cephalorhizum, Dictyolimon*, and *Popoviolimon*, but was here ascribed also to *Chaetolimon* and *Vassilczenkoa* since, although they do not exhibit spatulate leaves, their life-form is closer to the representatives of this syndrome than to those showing *Acantholimon* syndrome. We compared a model with syndrome-dependent speciation and extinction rates, and asymmetrical transition rates between the *Acantholimon* and *Limonium* syndromes (full BiSSE model, six parameters), against simpler, nested models where speciation, extinction or transition rates were constrained alternatively to be equal for the two states (five parameters). We assessed the significance of model differences by LRTs. To account for uncertainty in the estimation of model parameters, a Bayesian MCMC was run for 10,000 generations using an exponential prior under the best fitting model. Chain convergence was verified using the R package coda (Plummer et al., 2006). All chains converged within the first 1,000 generations. However, to be conservative, we discarded the first 2,500 steps of every chain and concatenated the last 7,500 steps for each tree together to construct the posterior probability distributions.

### Ancestral Area Reconstruction

The biogeographic history of *Acantholimon* and allied genera was reconstructed using Lagrange (Ree and Smith, 2008) with the C++ implementation (http://code.google.com/p/lagrange). As for diversification, we removed all outgroup taxa. Lagrange implements the maximum likelihood dispersal–extinction–cladogenesis (DEC) model (Ree et al., 2005) to estimate the most likely ancestral geographic range based on current distributions of extant lineages. The DEC model has been used broadly to study biogeographic patterns in closely related taxa with restricted geographic ranges (e.g., Fabre et al., 2013). This model assumes extinction or dispersal by contraction or expansion of the ancestral geographic range, respectively, and allows estimating the probability of ancestral areas at each node of a phylogenetic tree. The range of *Acantholimon* s.l. and its close relatives was divided into seven major areas. In defining the boundaries of our operational areas, we considered the current distribution patterns of *Acantholimon* s.l. species, i.e., geographic areas defined by the congruence in distribution of two or more species, but also the existence of significant geological features that could have acted as barriers to dispersal, or whose appearance could have resulted in the formation of new species due to vicariance (Sanmartín, 2014). For instance, area B (Levant and E Anatolia) is separated from area A (Eastern Mediterranean region and W Anatolia) by the Anatolian Diagonal, an area that is both species-rich and seems to have functioned as barrier for many organisms (Davis, 1971; Avci, 1996). The large deserts lying in the middle of the Iranian plateau, Dasht-e Kavir and Dasht-e Lut, mark the geographical boundary between areas D (W Iran) and E (E Iran and Afghanistan). This “mixed” biotic-geological criterion was adopted to maximize congruence with other biogeographic studies on animal and plant organisms endemic to this region but with slightly different ecological and distribution patterns (Sanmartín, 2003; Roquet et al., 2009; Barres et al., 2013, etc.). We did not strictly follow Takhtajan's floristic regions because in our case it did not entirely capture the fine-grained distribution patterns in *Acantholimon*, and because boundaries between floristic regions in Takhtajan's classification reflect current geological and climatic settings but not necessarily older history. Use of Takhtajan's subregions within the IT floristic region was also problematic because of the intractability of a DEC analysis including more than 9 areas (Landis et al., 2013). The seven areas considered were: (A) Eastern Mediterranean region and W Anatolia including Greece and western Turkey; (B) Levant and E Anatolia; (C) Caucasus region including Georgia, Azerbaijan, and Armenia; (D) W Iran including most of the Iranian Plateau except for the eastern part; (E) Eastern Iran and Afghanistan including the eastern part of the Iranian plateau and Afghanistan; (F) Central Asia defined as the Turanian or trans-Caspian region, including the republics of Turkmenistan, Uzbekistan, Tajikistan, Kirzigistan, and most of Kazakhstan; and (G) Western Himalayas. The BEAST MCC tree was used for the Lagrange analysis. A geographical range matrix was constructed, coding each species as present or absent in each of the seven geographical areas. To reduce the size of this matrix and avoid non-identifiability with optimized ranges, we constrained widespread ancestral ranges to include only geographically adjacent areas assuming that transitions between widespread non-adjacent ancestral ranges involve an additional extinction event.

## Results

### Estimated Divergence Time

*Acantholimon* s.l. diverged from its sister genus 7.48 Mya (4.91–11.72 95% HPD) and started to diversify 4.08 Mya (2.61–6.22 95% HPD). Ages of the earliest diverging lineages within *Acantholimon* s.l., i.e., clades A and B, were estimated to be 2.88 Mya (1.21–4.88 95% HPD) and 2.58 Mya (0.98–4.28 95% HPD), respectively (Figure 1). Within clade A, non-cushion species corresponding to the former *Vassilczenkoa* and *Chaetolimon* (A1) diverged 0.74 Mya (0.22–1.46), whereas those corresponding to *Cephalorhizum, Bamiania, and Popoviolimon* (clade A2) diverged 1.50 Mya (0.79–2.37). Within clade B, the lineage including non-cushion species gathered in the former genera *Dictyolimon* and *Bukiniczia* (B1) diverged 1.49 Mya (0.53–2.10). Cushion forming species—exhibiting *Acantholimon* syndrome—originated around 1.14 Mya (0.19–1.88; clade A3) and 1.20 Mya (0.50–2.10; clade B2), respectively (Figure 1).

### Diversification Analyses

**Figure 2:**
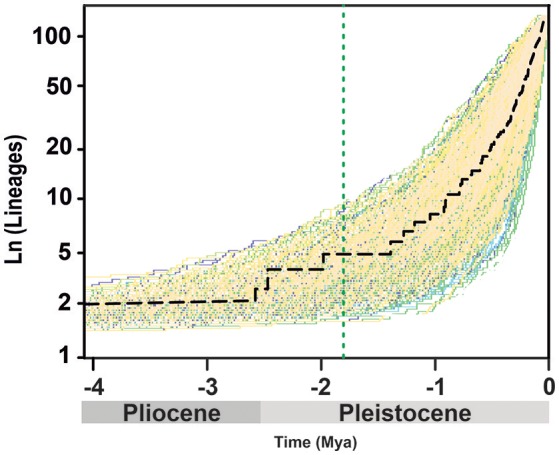
Accumulation of *Acantholimon* s.l. lineages through time, showing the maximum clade credibility tree (dash black line) and 1,000 trees from the post-burnin 95% highest posterior density distribution, representing uncertainty time estimates. The dotted green line marks a possible mass extinction event inferred by *TreePar*.

The lineage-through-time (LTT) plot shows slow diversification before c. 1.5 Mya, after which the slope of the curve (rate of lineage accumulation) increases, accelerating toward 0.1 Mya (Figure 2). Among rate-variable models, *TreePar* supported a one-rate shift model, within *Acantholimon* s.l., with a slight increase of diversification rate and a joint marked decrease in turnover at time *t* = 0.015 Mya (Table 1). This model showed a significantly better fit (*p* = 0.99) to the curve than a constant birth-death model based on LRT tests; the two-rate-shift model was not significantly better than the one-rate-shift model (*p* = 0.83). The model supporting one MEE showed a better fit to the data than a constant birth-death model, albeit non-significant (*p* = 0.90), but worse than the one-rate-shift model (*p* = 0.99). Such MEE removed c. 89% of extant lineages at *t* = 1.81 Mya (sp = 0.11, Table 1). Subsequent models allowing for a higher number of MEEs were not significant (Table 1).

**Table 1:** Results of *TreePar* diversification analyses with or without mass extinction.

**Models**	**Mass extinction events disallowed**			**Mass extinction events allowed**		
	**Birth-death**	**One-rate-shift**	**Two-rate-shifts**	**Models**	**One MEE**	**Two MEEs**
NP	2	5	8	NP	4	6
logL	3.5723197	−2.794629	−5.2786554	logL	1.2051645	−3.534386e-01
*P* (LRT)	null model	0.9947511^a^	0.8258485^b^	*P* (LRT)	0.9953212^c^ 0.906253^d^	0.4615936^e^
r1	0.7409803	7.341848e-01	−2.49688967	r	1.2957501	1.525927
ε1	0.8875540	1.479852e-07	4.56728246	ε	0.7877387	7.441365e-01
st1	–	1.530905e-02	0.01530905	st1	1.8153090	1.815309
r2	–	6.188155e-01	2.93064817	sp1	0.1140093	1.156866e-01
ε2	–	9.179178e-01	0.52331151	st2	–	3.215309
st2	–	–	0.41530905	sp2	–	3.420166e-08
r3	–	–	0.29091967		–	–
ε3	–	–	0.92531393		–	–

a *LRT (constant birth-death vs. one-rate-shift)*.

b *LRT (one-rate-shift model vs. two-rate-shift model)*.

c *LRT (1 MEE vs. one-rate-shift model)*.

d *LRT (constant birth-death vs. one MEE)*.

e *LRT (one MEE vs. two MEEs)*.

*NP, number of parameters; logL, (–log-likelihood value); mass extinction event (MEE), P (LRT), p-value of the likelihood ratio test; r, rate of diversification; **ε**, turnover (extinction/speciation); st, shift time; sp, survival probability after a MEE; in which “r1”, “**ε**1”, and “sp1” denote the diversification rate, the turnover, and the probability of survival, respectively, inferred between present (0 Mya) and the first rate or MEE (st1)*.

**Figure 3:**
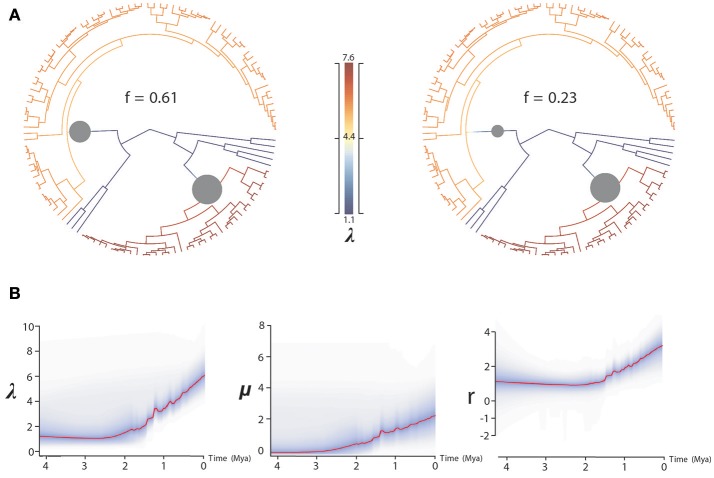
BAMM analysis of rate shifts in diversification within *Acantholimon* s.l. **(A)** Rate shift configurations (credible rate shift sets) with the highest posterior probability model showing two significant rate shifts in speciation (λ)—color coded by λ and marked by circles in the nodes. **(B)** Rates-through-time analysis of speciation (λ), extinction (μ) and net diversification (r) in *Acantholimon* s.l. over the last 4.08 Mya.

BAMM supported significant rate heterogeneity within *Acantholimon* s.l., recovering two significantly supported shifts in diversification at the crown nodes of *Acantholimon* s.str. species within clade A and clade B (A3, B2; Figures 1, 3A); the two most credible rate shift sets differed only in the position of the shift along the stem or crown node of *Acantholimon* s.str. species within clade B (B2; Figures 1, 3A). Mean diversification rates in clades A3 and B2 were inferred to be almost twice as high as in the rest of the tree (Table 2; Figure 3B). Rate-through-time plots showed that this was due to accelerated speciation rates, up to 7.6 (Figure 3A), starting c. 1 Mya (Figure 3B).

**Table 2:** Speciation (λ), extinction (μ) and mean diversification rates (λ-μ) for the entire tree, and for *Acantholimon* s.str. from BAMM.

**Entire tree**	
Speciation rate (λ)	3.26 (90% HPD: 2.61–4.07)
Extinction rate (μ)	1.38 (90% HPD: 0.52–2.50)
Mean diversification rate (λ-μ)	1.88 species Mya^−1^
***Acantholimon*** **s.str. within clade A (subclade A3)**	
Speciation rate (λ) for subclade A3	5.58 (90% HPD: 4.58–6.48)
Extinction rate (μ) for subclade A3	2.45 (90% HPD: 0.31–5.55)
Mean diversification rate (λ-μ)	3.13 species Mya^−1^
***Acantholimon*** **s.str. within clade B (subclade B2)**	
Speciation rate (λ) for subclade B2	4.98 (90% HPD: 3.67–5.99)
Extinction rate (μ) for subclade B2	1.53 (90% HPD: 0.17–3.53)
Mean diversification rate (λ-μ)	3.45 species Mya^−1^

**Figure 4:**
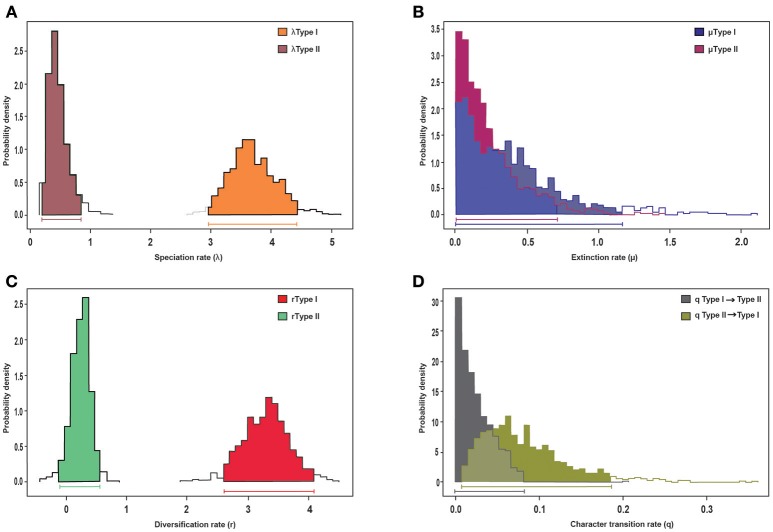
BiSSE analysis of diversification of the MCC tree and its association to morphological trait evolution, specifically life-form and leaves, represented by the *Acantholimon* (Type I) and the *Limonium* (Type II) syndromes. Posterior distributions of parameters obtained in the MCMC-BiSSE analysis: **(A)** speciation rates, **(B)** extinction rates, **(C)** diversification rates, **(D)** character transition rates. Horizontal bars indicate the 95% credibility interval for each parameter.

Trait-dependent diversification models (BiSSE) suggest that the diversification rate heterogeneity detected by BAMM is an effect of morphological syndrome on diversification rates (Table S2). In particular, we detected a significant effect on speciation rates, as the “symmetric speciation” model was rejected for the MCC tree. Bayesian estimation of BiSSE parameters revealed significantly higher speciation and diversification rates for the *Acantholimon* syndrome than for the *Limonium* syndrome, as shown by the non-overlapping 95% credibility intervals obtained when analyzing the MCC tree (Figures 4A,C). No effect on extinction rates was detected (see the widely overlapping 95% credibility intervals in Figure 4B). Transition rates were not significantly different between syndromes, as indicated by overlapping Bayesian posterior densities of the estimated rate (Figure 4D).

### Ancestral Area Reconstruction

**Figure 5:**
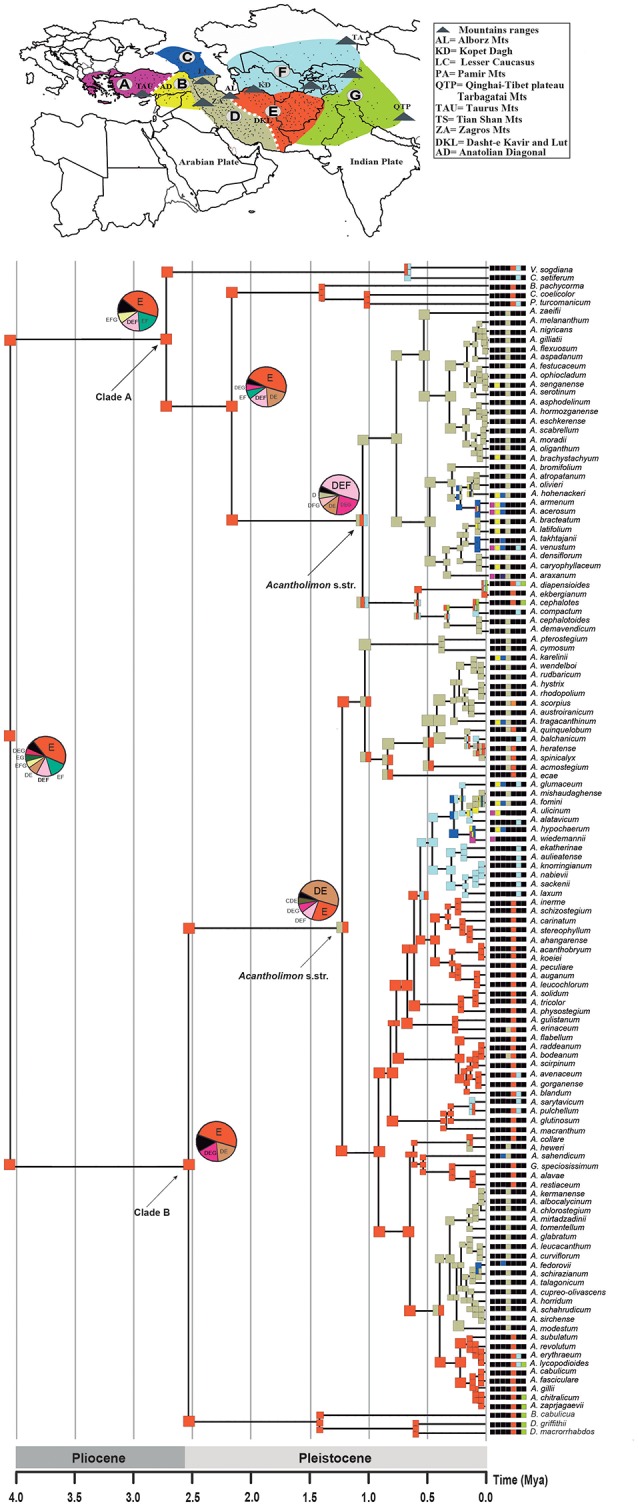
Ancestral Area Reconstruction of *Acantholimon* s.l. estimated in LAGRANGE using the seven operational areas shown in the map above. Current species distribution across the seven areas is indicated by color-coded squares next to the terminals; black squares indicate absence in the corresponding area. Ancestral areas and pie charts at nodes represent alternative range probabilities; black slices in pie charts indicate equivocal range probabilities. Scale depicts geological period time scale. An outline of *Acantholimon* distribution is provided by dots representing specimen locations obtained from GBIF.org (7th October 2015). GBIF Occurrence Available online at: (http://doi.org/10.15468/dl.aeqcxf).

The eastern Iran–Afghanistan region (area E) was inferred as the most likely ancestral area for *Acantholimon* s.l., as well as for the two main lineages (clades A and B; Figure 5; Table S3). Many species were also reconstructed as having originated within this region. The ancestral area for the MRCA of *Acantholimon* s.str. species within clade B (subclade B2) was widespread in Western Iran and Eastern Iran–Afghanistan (areas DE), implying a dispersal event to the west. Within clade A, the ancestral area of the MRCA of *Acantholimon* s.str. species (subclade A3) was inferred to be widespread in Iran–Afghanistan and Central Asia (DEF). The other regions (A to C, and G) seem to have been colonized by later dispersal events westwards and eastwards, followed by peripatric speciation, or rarely vicariance (e.g., between C and D; Figure 5).

## Discussion

### Parallel Diversification Rate Shifts and Key Innovations Explain Diversity Patterns Within *Acantholimon*

The uneven distribution of species diversity across clades and regions is a candent topic in evolutionary biology (Wiens, 2017). Species-rich clades can be explained by either their age of origin, i.e., older clades have more time to accumulate lineages (McPeek and Brown, 2007), or by events of rapid diversification (evolutionary radiations). Our analyses using several diversification rate methods support the hypothesis that *Acantholimon* s.l. species diversity resulted from two parallel events of accelerated speciation rates in subclades A3 and B2 (Figure 3A), which were linked to the acquisition of morphological novelties in habit and leaf form. Clade heterogeneity-aware method BAMM identified these two events, with only slight uncertainty on the timing along the stem/crown node of clade B2, and linked them to an increase in speciation rates (Figure 3). Trait-dependent diversification method BiSSE supported an association between diversification rates and morphological evolution: the (independent) acquisition of the *Acantholimon* syndrome, i.e., the joint occurrence of cushion life-form and linear rigid pungent leaves, was associated to higher speciation rates, with no significant effect on extinction rates, compared to the *Limonium* syndrome.

Time-dependent diversification models implemented in *TreePar* supported one rate shift toward higher diversification rates at 0.015 Mya, and a potential mass extinction event removing more than 90% of extant diversity at 1.81 Mya, which predates the clade-dependent rate shifts detected by BAMM (Figure 2, Table 1). However, we recommend caution in interpreting these results since it has been shown that methods such as *TreePar* exhibit a high Type II error in the case of rate heterogeneity due to clade-specific shifts (Laurent et al., 2015). Thus, sister-clades exhibiting different diversification dynamics such as in the case of *Acantholimon* s.str. species (A3, B2) and their species-poor relatives (A1, A2, B1) (Figure 1) could have misled the *TreePar* results. Yet, we cannot reject the possibility that high extinction rates in *Acantholimon* before the divergence of the major clades could have contributed to the observed diversification pattern.

Diversification rates estimated in *Acantholimon* (up to *r* = 3.45 species Myr^−1^; Table 2, Figure 3B) are comparable to those in other lineages proposed as examples of rapid radiations (e.g., *Pelargonium* L'Hér., Bakker et al., 2005; *Lupinus* L., Hughes and Eastwood, 2006; Drummond et al., 2012b; *Astragalus* L., Scherson et al., 2008; *Castilleja* Mutis ex L. f., Tank and Olmstead, 2008, 2009; *Indigofera* L., Schrire et al., 2009; Dianthus L., Valente et al., 2010; *Tragopogon* L., Bell et al., 2012; *Lobelioideae*, Lagomarsino et al., 2016). These radiations have generally been attributed to ecological opportunity and/or evolutionary innovation, but relatively few studies have identified multiple diversification rate shifts coinciding with the evolution of the same derived traits (Drummond et al., 2012b). Our analyses with BAMM and BiSSE indicate that the newly arisen *Acantholimon* syndrome facilitated two parallel events of accelerated speciation rates in clades A3 and B2, suggesting that these morphological novelties in life form and leaf shape provided a competitive advantage for species in those lineages.

What could have been those advantages? We can hypothesize that dry continental environmental conditions, poor soils, and herbivory exerted strong selection pressures on *Acantholimon* s.l. lineages. Cushion life-form is a classic example of a convergent character that has repeatedly favored the colonization of cold and dry environments (Boucher et al., 2016). Not all species in the two speciose lineages (A3 and B2) are cushion-forming, but those that are not form densely branched subshrubs. Though there are some species living below 1,000 m in the Mediterranean parts of Anatolia (Dogan and Akaydin, 2007), most *Acantholimon* species occur in mountainous habitats, where cushion (or densely cespitose) life-forms are suitable to thrive. Only 14 (8.53%) of the 164 *Acantholimon* species recognized in the *Flora Iranica* include herbarium records below 1,000 m of elevation (Rechinger and Schiman-Czeika, 1974). And for 13 of those species with records below 1,000 m, there are additional records well above such elevation, even up to 3,000 m. The highest specimen recorded for this genus in *Flora Iranica* is at 4,650, 4,800 m in the *Flora of China* (Peng and Kamelin, 1996; note that *Flora Iranica* covers not only Iran but also Afghanistan and adjacent territories of Turkmenistan, Pakistan and Iraq).

The other morphological character associated to the parallel acceleration in diversification rates is leaf shape. Flattened leaves with an expanded limb occur in most of the genera merged under *Acantholimon* based on molecular data (Moharrek et al., 2017), i.e., *Bamiania, Bukiniczia, Cephalorhizum, Dictyolimon*, and *Popoviolimon*, as well as, profusely, in the outgroups, e.g., *Goniolimon* and *Limonium*. In contrast, those species included in the two highly diversified lineages (clades A3 and B2, Figure 1) typically show linear rigid acuminate leaves, representing a minimal leaf surface that reduces water loss, and whose pungency defends the plant against large herbivores. Nine of those 14 species with records below 1,000 m in *Flora Iranica* belong in sect. *Tragacanthina* Bunge. This was characterized by some degree of seasonal leaf dimorphism consisting of non-rigid spring leaves—a season when evapotranspiration is lower—and rigid summer leaves. This example suggests that rigid acuminate leaves may have been selected for extreme dry continental climates and that only *Acantholimon* species with relatively mild climate during part of the year exhibit non-rigid leaves. It is likely that the joint occurrence of these two characters—cushion life-form and rigid acuminate leaves—, with important functions for survival in extreme continental environments helped *Acantholimon* to become one of the dominant genera along vast extensions of the IT region. Interestingly, a strikingly similar morphological syndrome including life-form and leaves is exhibited by the genus *Acanthophyllum*, another large IT genus occurring in similar habitats, but belonging to another family, Caryophyllaceae (Pirani et al., 2014).

But, is the *Acantholimon* syndrome a key innovation? This term is subjected to controversy; possibly in part because of the recurring comparison with the most paradigmatic example, i.e., the pharyngeal jaw apparatus of cichlid fishes (Liem, 1973; Givnish, 2015). This anatomical structure triggered the invasion of a wide range of adaptive zones based on its maximum versatility. In contrast, the two morphological traits acquired independently in *Acantholimon* seem to lack the potential adaptive versatility of the pharyngeal jaw apparatus, but could have facilitated the colonization of vast territories of seemingly homogeneous continental high elevation environments and promoted a rapid subsequent diversification. It is in this sense that key innovation may be applied to the cushion life-form and pungent leaves in *Acantholimon* although alternatively a new term could be needed (Losos and Mahler, 2010).

### Biogeographic History of *Acantholimon*

What was the climatic and geological framework within which *Acantholimon* evolved? Molecular dating analysis suggests that *Acantholimon* s.l. started to diversify sometime between the Late Miocene and Pliocene, whereas the onset of differentiation within the two lineages that constitute *Acantholimon* s.str. (clades A3 and B2) is dated in the Pleistocene (Figure 1). These dates are much younger than the tectonic and climatic events that shaped the current environmental conditions of the Irano-Turanian region. Climatically, the history of the region is shaped by both aridification and cooling. The Pleistocene glacial episodes took place over an already existing global gradual cooling trend that had started in the Oligocene (Zachos et al., 2008) and an aridification in central continental Asia that started by the early Miocene (Guo et al., 2002; Miao et al., 2012). Two major tectonic events shaped the topography and climate of IT region: the collisions of the Indian and Eurasian plates, started in the early Eocene (Yin, 2010; Smit et al., 2013), and that of the Arabian and Eurasian plates, started in the late Oligocene (Mouthereau et al., 2012). These two collisions triggered the uplifts of mountain ranges (Taurus, Caucasus, Zagros, Alborz, Kopet Dagh, Tian Shan, Pamir) and plateaus (Iranian, Anatolian, Qinghai-Tibetan) in the region, in a rather asynchronic and discontinuous fashion over the last 20 Ma (Li et al., 1996; Koçyigit et al., 2001; Wu et al., 2001; Meulenkamp and Sissingh, 2003; Popov et al., 2004; Guest et al., 2007; Buslov et al., 2008; Hatzfeld and Molnar, 2010; Mosar et al., 2010; Djamali et al., 2012b; Mouthereau et al., 2012; Favre et al., 2015). The uplift of these mountain ranges and plateau regions caused an increasing climate cooling and aridification through the formation of rain shadows on a large scale (Manafzadeh et al., 2017). It is in this context of increasing aridity and cooling in the IT region where diversification and expansion of *Acantholimon* species could have found favorable conditions over the last two million years. The Pleistocene date for the accelerated diversification events found in *Acantholimon* (Figure 1) contrasts with the older, Miocene, radiation reported in other speciose IT genera such as *Cousinia* (López-Vinyallonga et al., 2009; Djamali et al., 2012b). In this genus, Pleistocene climatic oscillations were inferred to have caused only moderately negative effects on preexisting diversification. By contrast, in *Acantholimon*, the altitudinal distribution of its cold-adapted xerophytic species, together with the inferred tempo of diversification, is consistent with an allopatric speciation scenario, in which Pleistocene climatic oscillations actively promoted diversification: range contraction and differentiation in isolation in the highest peaks during interglacial periods vs. range expansion and migration during glacial periods likely contributed to the divergence of gene pools and eventually to allopatric speciation.

The biogeographic analysis suggests that the origin of *Acantholimon* s.l. is most likely eastern Iran–Afghanistan; an area that may have acted as a “species pump” (Figure 5). This possibility is consistent with the fact that Iran and Afghanistan are the major centers of the taxonomic diversity of the genus (Kubitzki, 1993). The origin of the two major clades A and B is inferred to be eastern Iran–Afghanistan and migrations from this region appear to have occurred mainly following several lines: (1) westwards into W Iran and then northwards to the Caucasus, Anatolia and Eastern Mediterranean regions, (2) eastwards into the northern parts of Pakistan (Baluchistan) and the western Himalayas (India), (3) northwards into Central Asia and the Pamir mountains and then westwards to the Caucausus and Anatolia (Figure 5). A westward colonization of the Mediterranean region from IT lineages has been also described in other IT groups such as *Haplophyllum* (Manafzadeh et al., 2014). However, our phylogenetic results in *Acantholimon* indicate that most species from the same floristic region do not form monophyletic groups (Figure 5). The Anatolian and eastern Mediterranean representatives of the genus do not all cluster together but are embedded within clades that primarily include species from the inferred origin of the genus, Iran and Afghanistan (area E), and species that occur in more than one floristic region (Figure 5). This suggests that multiple westward invasions of the western regions from the core of the IT region took place during the evolution of the genus. A similar lack of monophyly is found for species occurring north and east of Iran and Afghanistan (areas F, G) although several central Asian species seem to have a single origin (*A. glumaceum* to *A. laxum* in clade B; Figure 5). Our results suggest that the IT region acted both as a cradle for new species, via high speciation rates, and as a museum, preserving older lineages that began to radiate outside this region. Additionally, one could ask about the relative importance of colonization vs. *in situ* diversification in assembling the IT biota (e.g., Xing and Ree, 2017). The high number of endemics in *Acantholimon* concentrated in an area inferred as the origin of the genus indicates a major contribution of *in situ* diversification.

### Non-adaptive Radiation?

The elements and limits of the classical adaptive radiation model (Schluter, 2000; Glor, 2010; Givnish, 2015) and its relative importance compared to other models of evolutionary radiations (Simões et al., 2016) have been much debated. Niche modeling and niche overlap studies of every *Acantholimon* species are needed to determine the interactions between environmental factors and the fast diversification and associated key innovations documented in this genus. However, available data suggest that speciation in the IT *Acantholimon* was not accompanied by niche differentiation. A large amount of information on habitats of *Acantholimon* species recorded in floras, herbarium records, regional works (Linczevski, 1967; Rechinger and Schiman-Czeika, 1974; Peng and Kamelin, 1996; Assadi, 2005; Dogan and Akaydin, 2007) and personal observations consistently depict a rather uniform habitat: stony mountain slopes or summit areas with poorly developed soils, often dry, sometimes directly on rocky outcrops both limestone and granite. These environments where *Acantholimon* species occur are frequent and even dominant in the IT region, and host also other species-rich genera, such as *Acanthophyllum, Astragalus, Cousinia, Haplophyllum, Onobrychis* Mill. (Podlech and Maassoumi, 2003; Ranjbar and Karamian, 2003; López-Vinyallonga et al., 2009; Djamali et al., 2012b; Manafzadeh et al., 2014; Pirani et al., 2014; Amirahmadi et al., 2016).

If such a lack of niche differentiation is confirmed in *Acantholimon* species, a non-adaptive model, i.e., involving niche conservatism (Gittenberger, 1991; Kozak et al., 2006; Rundell and Price, 2009), would be the best explanation for its diversification patterns. Specifically, such a scenario could approach a geographic radiation model sensu Simões et al. (2016) and Givnish (2015), which states that geographic speciation in “extensive cordilleras, archipelagoes, lake systems or submarine outcrops dissected by multiple natural barriers to gene flow and species dispersal” can also result in explosive diversification.

In sum, rapid diversification in *Acantholimon* was probably facilitated by colonization of available mountainous environments enabled by key innovations, and by climatic-driven Pleistocene range shifts. Further work is needed to assess the role that niche differentiation vs. allopatry have played in its evolutionary radiation and the contribution of those elements to the assembly of the entire IT flora.

## Data Availability Statement

The xml file used for the molecular dating in BEAST and the resulting phylogenetic tree in nexus file format are published as Data Sheets S1 and S3, respectively, in the Supplementary Material.

## Author Contributions

FM, GNF, and IS conceived and designed this study. FM made field collections and molecular data compilation with contributions from SK-O on both aspects. FM and IS analyzed the data with help from GNF. FM and GNF wrote the manuscript with important contributions from IS. All authors contributed to manuscript revision, read, and approved the submitted version.

### Conflict of Interest Statement

The authors declare that the research was conducted in the absence of any commercial or financial relationships that could be construed as a potential conflict of interest.

## Supplementary Material

Data Sheet S1 Voucher information: species name, locality, *collector(s), voucher* (herbarium), GenBank accession numbers for nrDNA ITS and *trn*Y-T, respectively.

Data Sheet S2 XML file used for molecular phylogenetic dating of the complete dataset comprising 130 species from *Acantholimon* s.l. and 26 outgroup taxa, using the two-calibration point strategy (see main article).

Data Sheet S3 Maximum clade credibility (MCC) tree showing mean estimates and 95% high-posterior-density (HPD) credibility intervals obtained from the BEAST analysis of the molecular dataset comprising 130 species from *Acantholimon* s.l. and 26 outgroups taxa, using the two-calibration point strategy (see Table S1). The tree is the same as the one shown in Figure 1.
