# Supplementary material for: Morphological Innovations and Vast Extensions of Mountain Habitats Triggered Rapid Diversification Within the Species-Rich Irano-Turanian Genus Acantholimon (Plumbaginaceae)
Source: Front Genet. 2019 Jan 21;9:698. doi: 10.3389/fgene.2018.00698 (PMC6360523; doi:10.3389/fgene.2018.00698)
Supplement: Supplementary file 1 [file Table_1.docx]

**Table S1.** Divergence time estimates for major clades using relaxed clock models implemented in BEAST. The values under the heading "One calibration point" were obtained in four different analysis using a single calibration on the node of the most recent common ancestor (MRCA) of *Armeria + Psylliostachys + Limonium, Armeria + Psylliostachys, Armeria,* and *Limonium*, respectively, based on fossil pollen from the Upper Miocene (estimates from these four analyses are separated by /). This calibration point was assigned a lognormal distribution prior with offset = 5.33 Mya, standard deviation = 1.15, that comprises the entire geological interval of the Upper Miocene (5.333 – 11.62 Mya). The values under the heading "Two calibration points" were obtained using the following calibration points: the fossil pollen assigned to the stem node of *Armeria* (MRCA of *Armeria + Psylliostachys*), and a secondary calibration for the Plumbaginaceae crown-node based on Magallón et al. (2015), using a normal distribution prior with median = 41.67 Mya and standard deviation (SD) = 10.8 Mya that covers the 95% high posterior density (HPD) credibility interval for that node in the original study (S. Magallón, pers. comm). Median age estimates as well as the 95 HPD intervals are shown for all five analyses.

|  | One calibration point:  Miocene pollen (4 analyses) | | Two calibration points:  Miocene pollen and secondary calibration point from Magallón et al. (2015) | |
| --- | --- | --- | --- | --- |
| Node (mrca) | Median age (Mya) | 95% HPD (Mya) | Median age (Mya) | 95% HPD (Mya) |
| Root | 23.54/27.10/ 33.20/28.26 | 10.07-41.38/14.16-42.78/20.20-47.65/15.66-43.90 | 33.26 | 20.14-51.81 |
| *Plumbago*- *Dyerophytum* | 5.95/6.79/8.42/7.08 | 1.58-14.13/2.37-14.93/4.08-16.23/3.08-15.33 | 8.55 | 19.01-1.98 |
| *Limonium – Armeria - Psylliostachys* | 6.34/7.86/11.52/9.50 | 5.34-10.42/6.81-11.86/9.14-15.94/7.80-14.02 | 10.83 | 18.20-5.12 |
| *Goniolimon*-*Acantholimon* | 6.02/6.90/7.77/7.08 | 4.40-9.22/5.39-10.59/6.17-10.78/5.48-10.60 | 7.48 | 4.91–11.72 |
| *Acantholimon* s.l. | 2.14/2.62/4.31/2.99 | 0.92-4.20/1.04-4.56/2.59-5.75/1.77-4.48 | 4.08 | 2.61–6.22 |
| Clade A | 1.69/2.07/3.00/2.54 | 0.70-3.26/1.08-3.64/2.01-4.57/1.55-4.11 | 2.88 | 1.21–4.88 |
| Clade B | 1.59/1.92/2.84/2.43 | 0.66-3.16/0.99-3.43/1.85-4.41/1.50-4.01 | 2.58 | 0.98–4.28 |
