# Supplementary material for: Morphological Innovations and Vast Extensions of Mountain Habitats Triggered Rapid Diversification Within the Species-Rich Irano-Turanian Genus Acantholimon (Plumbaginaceae)
Source: Front Genet. 2019 Jan 21;9:698. doi: 10.3389/fgene.2018.00698 (PMC6360523; doi:10.3389/fgene.2018.00698)
Supplement: Supplementary file 2 [file Table_2.docx]

**Table S2.** Model testing of syndrome-dependent speciation, extinction, and transition rates in *Acantholimon* s.l. using BiSSE.

|  | **df** | **lnL** | **AIC** | **ChiSq** | **Pr(>\|Chi\|)** |
| --- | --- | --- | --- | --- | --- |
| **Full model** | 6 | 9.6855 | -7.3709 |  |  |
| **Equal speciation rates** | 5 | 1.2711 | 7.4579 | 16.829 | 4.091e-05* |
| **Equal extinction rates** | 5 | 8.3792 | -6.5510 | 2.8952 | 0.08885 |
| **Equal transition rates** | 5 | 8.2123 | -6.4246 | 2.9464 | 0.08607 |

| We used likelihood-ratio tests implemented in the R package *diversitree* to compare alternative diversification models with different association degrees among the parameters speciation, extinction, and transition rates between the *Acantholimon* and *Limonium* morphological syndromes. Results showed that the model allowing for different speciation rates but equal extinction and transition rates between syndromes did not perform significantly worse than the full model, and was therefore used in the subsequent Bayesian MCMC analyses (see Fig. 4). Abbreviation: df, degrees of freedom; lnLik, log likelihood; AIC, Akaike Information Criterion; *, Pr < 0.05. |
| --- |
