# Supplementary material for: Morphological Innovations and Vast Extensions of Mountain Habitats Triggered Rapid Diversification Within the Species-Rich Irano-Turanian Genus Acantholimon (Plumbaginaceae)
Source: Front Genet. 2019 Jan 21;9:698. doi: 10.3389/fgene.2018.00698 (PMC6360523; doi:10.3389/fgene.2018.00698)
Supplement: Supplementary file 3 [file Table_3.docx]

**Table S3.** Relative Likelihood values for alternative nodal ancestral range reconstructions for major nodes within *Acantholimon* s.l. estimated under the DEC model using LAGRANGE (Ree & Smith, 2008). "Equivocal": ancestral ranges receiving less than 0.1 relative likelihood were pooled together under this category.

| Node | Ancestral states | Likelihood |
| --- | --- | --- |
| *Acantholimon* s.l. | E | 0.44 |
|  | EF | 0.11 |
|  | DEF | 0.10 |
|  | DE | 0.09 |
|  | EFG | 0.06 |
|  | EG | 0.06 |
|  | DEG | 0.06 |
|  | Equivocal | 0.08 |
| Clade B | E | 0.49 |
|  | DE | 0.17 |
|  | DEG | 0.17 |
|  | Equivocal | 0.17 |
| Subclade B2 | DE | 0.48 |
|  | E | 0.17 |
|  | DEF | 0.10 |
|  | DEG | 0.09 |
|  | CDE | 0.08 |
|  | Equivocal | 0.08 |
| Clade A | E | 0.45 |
|  | EF | 0.15 |
|  | DEF | 0.15 |
|  | EFG | 0.12 |
|  | Equivocal | 0.13 |
| Subclades A3 + A2 | E | 0.48 |
|  | DE | 0.15 |
|  | DEF | 0.14 |
|  | EF | 0.09 |
|  | DEG | 0.07 |
|  | Equivocal | 0.07 |
| Subclade A3 | DEF | 0.48 |
|  | DEG | 0.17 |
|  | DE | 0.11 |
|  | DFG | 0.09 |
|  | D | 0.08 |
|  | Equivocal | 0.07 |
