# Supplementary material for: Morphological Innovations and Vast Extensions of Mountain Habitats Triggered Rapid Diversification Within the Species-Rich Irano-Turanian Genus Acantholimon (Plumbaginaceae)
Source: Front Genet. 2019 Jan 21;9:698. doi: 10.3389/fgene.2018.00698 (PMC6360523; doi:10.3389/fgene.2018.00698)
Supplement: Data Sheet S1 — Voucher information: species name, locality, collector(s), voucher (herbarium), GenBank accession numbers for nrDNA ITS and trnY-T, respectively. [file Data_Sheet_1.docx]

**APPENDIX S1.**

Voucher information: species name, locality*, collector(s), voucher* (herbarium), GenBank accession numbers for nrDNA ITS and *trn*Y-T, respectively.

*Acantholimon acanthobryum* Rech.f. & Schiman-Czeika, Afghanistan, Ghazni, Dasht-e Nawar, *Freitag 1561* (MSB 006140), LC153804, -; *Acantholimon acerosum* (Willd.) Boiss., Turkey, Antalya, Bey Dağları, *Eren s.n.* (W 2014-12433), LC153805, LC153954; *Acantholimon acmostegium* Boiss. & Buhse, Iran, Khorasan, Bojnord, *Kazempour-Osaloo & Moharrek s.n.* (TMUH 92351), LC153807,LC153956; *Acantholimon ahangarense* Rech.f. & Schiman-Czeika, Afghanistan, Ghorat, Shutur Khan Kotal, *Rechinger 18860* (W 1975-0013927), LC153808, LC153957; *Acantholimon alatavicum* Bunge, Uzbekistan, Tian-Shan Mts., Kuraminski khrebet, *Vašák s.n.* (MA 642281), LC153809, LC153958; *Acantholimon alavae* Rech.f. & Schiman-Czeika, Iran, Khorasan, Fariman, *Joharchi & Zanguei 36488* (FUMH), AB979533, AB979601; *Acantholimon albocalycinum* Assadi & Mirtadzadini, Iran, Kerman, Sirch, *Mirtadzadini s.n.* (SHBU 1239), AB979534, AB979602; *Acantholimon araxanum* Bunge, Iran, W Azarbaijan, Mt. Seyed Hajidin, *Assadi 86873* (TARI), AB979535, AB979603; *Acantholimon armenum* Boiss. & A.Huet, Turkey, Gümüşhane, Ak Dağ, *Aldasoro et al. 2504* (MA 690163), LC153810, LC153959; *Acantholimon aspadanum* Bunge, Iran, Kashan, Mt. Karkas, *Assadi 82735* (TARI), AB979536, -; *Acantholimon asphodelinum* Mobayen, Iran, Fars, Bambo National Park, *Jamzad et al. 69399* (TARI), LC153812, LC153961; *Acantholimon atropatanum* Bunge, Iran, W Azarbaijan, Oshnavieh road, *Kazempour-Osaloo s.n.* (TMUH 90125), AB979537, AB979604; *Acantholimon auganum* Bunge, Afghanistan, Bamian, Band-e Amir, *Schloeder & Jacobs 1776* (MSB 150698), LC153813, LC153962; *Acantholimon aulieatense* Czerniak., Kyrgyzstan, Talas vall kizovska lake, *Lažkov s.n.* (W 2001-0001267), LC153815, LC153964; *Acantholimon austroiranicum* Rech.f. & Schiman-Czeika, Iran, Kerman, Sirjan, *Mirtadzadini 83216* (TARI), AB979538, AB979605; *Acantholimon avenaceum* Bunge, Iran, Khorasan, Mashhad, *Mozaffarian 48909* (TARI), AB979539, -; *Acantholimon balchanicum* Korovin, Turkmenistan, Krasnovodskaya Oblast', Malyi Balkhan Mts., *Nikitin s.n.* (W 1985-0007275), LC153816, LC153965; *Acantholimon blandum* Czernjak., Iran, Khorasan, Bojnord, *Memariani & Zanguei 38376* (FUMH), AB979540, AB979606; *Acantholimon bodeanum* Bunge subsp. *bodeanum*, Iran, Golestan, Gorgan, *Assadi & Hamdi 85657* (TARI), AB979541, AB979607; *Acantholimon brachystachyum* Boiss. ex Bunge, Iran, Kurdistan, Marivan, *Mozaffarian 74817* (TARI), LC153817, LC153966; *Acantholimon bracteatum* (Girard) Boiss., Iran, W Azarbaijan, Salmas road, *Assadi 85186* (TARI), AB979544, AB979609; *Acantholimon bromifolium* Boiss. ex Bunge, Iran, Hamedan, Alvand Valley, *Assadi 61066* (TARI), AB979545, AB979610; *Acantholimon cabulicum* Boiss., Afghanistan, Bamian, Kelikan, *Schloeder & Jacobs 1629* (MSB 150699), LC153820,LC153969; *Acantholimon carinatum* Rech.f. & Schiman-Czeika, Afghanistan, Ghorat, Kuh-Tscheling-Safed-Daraq, *Rechinger 19063* (W 1974-0027625), LC153821, LC153970; *Acantholimon caryophyllaceum* Hausskn. ex Bunge, Turkey, Gümüşhane, *Herrero et al. AH 1337* (MA 688175), LC153822, LC153971; *Acantholimon cephalotes* Boiss., Afghanistan, Bamian, Band-e Amir, *Rechinger 18217* (MA 496821), LC153824, LC153973; *Acantholimon cephalotoides* Rech.f., Iran, Semnan, *Assadi 85403* (TARI), AB979546, AB979611; *Acantholimon chitralicum* Rech.f. & Schiman-Czeika, Pakistan, Rosh Gol, Tirich Mir, *Stainton 2810* (W 1960-0004579), LC153827, LC153974; *Acantholimon chlorostegium* Rech.f. & Schiman-Czeika, Iran, Kerman, *Mirtadzadini s.n.* (SHBU 1248), AB979547, AB979612; *Acantholimon collare* Köie & Rech.f., Iran, Khorasan, Ghayen, *Joharchi & Zanguei 36270* (FUMH), AB979548, AB979613; *Acantholimon compactum* Korovin, Kyrgyzstan, Turkestan range, Tortgul' lake, *Lažkov s.n.* (W 2007-0012047), LC153828, LC153975; *Acantholimon cupreo-olivascens* Rech.f. & Schiman-Czeika, Iran, Kerman, Lalehzar, *Mirtadzadini s.n.* (SHBU 1245), LC153829, LC153976; *Acantholimon curviflorum* Bunge, Iran, Isfahan, Kashan, *Assadi 82680* (TARI), AB979549, AB979614; *Acantholimon cymosum* Bunge, Iran, Semnan, Momen Abad, *Kazempour-Osaloo s.n.* (TMUH 90213), AB979550, AB979615; *Acantholimon demavendicum* Bornm., Iran, Tehran, Mt. Damavand, *Assadi & Hamdi 85720* (TARI), AB979551, AB979616; *Acantholimon densiflorum* Assadi , Iran, W Azarbaijan, Khoy, *Assadi & Olfat 68561* (TARI), LC153831, LC153978; *Acantholimon diapensioides* Boiss., Afghanistan, Bamian, Band-e Amir, *Uotila 18555* (W 1979-0003252), LC153834, -; *Acantholimon ecae* Aitch. & Hemsl., Afghanistan, Herat, Hari Rud Valley, *Freitag 5413* (W 1974-0027754), LC153835, LC153981; *Acantholimon ekatherinae* (B.Fedtsch.) Czerniak., Kyrgyzstan, Tian-Shan Mts., Chatkalski khrebet, *Vašák s.n.* (MA 642280), LC153836,LC153982; *Acantholimon ekbergianum* Rech.f. & Schiman-Czeika, Afghanistan, Bamian, Band-e Amir, *Wendelbo & Ekberg* *9765* (W 1975-0014142), LC153837, -; *Acantholimon erinaceum* (Jaub. & Spach) Lincz., Iran, Khorasan, *Mozaffarian 87080* (TARI), LC153839, LC153983; *Acantholimon erythraeum* Bunge, Kyrgyzstan, Turkestan range, Koshbulak, *Lažkov s.n.* (W 2007-0004898), LC153842, LC153985; *Acantholimon eschkerense* Boiss. & Hausskn. ex Boiss., Iran, Fars, Shiraz, *Mozaffarian 83648* (TARI), LC153844,LC153987; *Acantholimon fasciculare* Boiss., Afghanistan, Ghorat, Kuh-Tscheling-Safed-Daraq, *Rechinger 19117* (W 1975-0013899), LC153845, LC153988; *Acantholimon fedorovii* Tamamsch. & Mirzoeva, Armenia, Mer Gimsky, Agarak, *Gabrielian et al. s.n.* (W 2010-0004277), LC153847, LC153989; *Acantholimon festucaceum* (Juab. & Spach) Boiss., Iran, Kohgiluyeh & Boyer-Ahmad, Mt. Nil, *Assadi & Abu Hamzeh 46516* (TARI), LC153848, LC153990; *Acantholimon flabellum* Assadi, Iran, Khorasan, Esfarayen, *Joharchi & Zanguei 7566* (FUMH), AB979556, AB979618; *Acantholimon flexuosum* Boiss. ex Bunge, Iran, Chahar Mahaal-e Bakhtiari, Mt. Kelar*, Mozaffarian 58150* (TARI), AB979557, LC153993; *Acantholimon fominii* Kusn., Iran, W Azarbaijan, *Alizadeh & Ghasempour s.n.* (HWANRC 5254), LC153853, -; *Acantholimon gilliatii* Turril, Iran, E Azarbaijan, Mishu Dağ, *Assadi 85328* (TARI), AB979558, -; *Acantholimon gillii* Rech.f. & Köie, Afghanistan, Kunar, Schaschgaltal, *Rasoul 3489* (W 1970-0011241), LC153854, -; *Acantholimon glabratum* Assadi subsp. *Kashanense* Batuli & Assadi, Iran, Isfahan, Kashan, *Batuli 78415* (TARI), LC153855, -; *Acantholimon glumaceum* (Jaub. & Spach) Boiss., Turkey, Kütahya, Murat Dağ*, Vašák s.n.* (W 2003-0005690), LC153857, LC153994; *Acantholimon glutinosum* Rech.f. & Köie, Afghanistan, Kandahar, *Rechinger 35069* (MA 416837), LC153858, LC153995; *Acantholimon gorganense* Mobayen, Iran, Khorasan, Bojnord, *Joharchi & Zanguei 40571* (FUMH), AB979559, AB979619; *Acantholimon gulistanum* Bunge, Iran, Khorasan, Mashhad, Torghabeh, *Joharcii & Zanguei* *39162* (FUMH), AB979560, AB979620; *Acantholimon heratense* Bunge, Iran, Khorasan, Taybad, *Faghihnia & Zanguei 21914* (FUMH), AB979561, AB979621; *Acantholimon heweri* Rech.f. & Schiman-Czeika, Iran, Khorasan, Birjand, *Ayathollahi & Zangooi 12094* (FUMH), AB979562, AB979622; *Acantholimon hohenackeri* Boiss., Iran, Ardabil, Khalkhal road, *Jamzad & Azimi 82613* (TARI), LC153859, LC153996; *Acantholimon hormozganense* Assadi, Iran, Hormozagan, Bandar Abbas, *Wendelbo & Foroughi 15767* (TARI), LC153861, LC153997; *Acantholimon horridum* Bunge, Iran, Yazd, Mehriz to Herat road, *Assadi & Ranjbar 82864* (TARI), AB979564, AB979624; *Acantholimon hypochaerum* Mobayen, Turkey, Erzinkan, *Aldasoro et al. 2595* (MA 690037), LC153862, LC153998; *Acantholimon hystrix* Stapf, Iran, Isfahan, Natanz, *Assadi & Ranjbar 82768* (TARI), LC153863, -; *Acantholimon inerme* Rech.f. & Köie, Afghanistan, Ghazni, Luman, *Podlech 19398* (M 0276204), LC153864, LC153999; *Acantholimon karelinii* (Stschegl.) Bunge, Iran, E Azarbaijan, Jolfa to Poldasht road, *Assadi 86799* (TARI), AB979565, AB979625; *Acantholimon kermanense* Assadi& Mirtadz., Iran, Kerman, Mt. Bahr Aseman, *Mirtadzadini s.n.* (SHBU 1243), AB979566, AB979626; *Acantholimon knorringianum* Lincz., Kyrgyzstan, Fergan ridge, Banbash-Ata Mts*., Lažkov s.n.* (W 2007-0006249), LC153867, LC154001; *Acantholimon koeiei* Rech.f. & Schiman-Czeika, Afghanistan, Ghazni, Dasht-e Nawar, *Breckle 4606* (W 1977-0005153), LC153868, LC154002; *Acantholimon latifolium* Boiss., Iran, Kurdistan, Mt. Avalan, *Maassoumi & Safavi 86028* (TARI), AB979567, AB979627; *Acantholimon laxum* Czerniak., Kyrgyzstan, Kavak-Too ridge, Sary-Bulun, *Lažkov s.n.* (W 2007-0012055), LC153870, LC154003; *Acantholimon leucacanthum* Boiss., Iran, Isfahan, Isfahan to Shahreza road, *Parishani 14391* (MA 748552), LC153871, LC154004; *Acantholimon leucochlorum* Rech.f. & Schiman-Czeika, Afghanistan, Ghazni, Pam koh, Qablan, *Podlech 31998* (MSB 006150), LC153872, LC154005; *Acantholimon lycopodioides* Boiss., Afghanistan, Parwan, Salang-Tal, *Breckle 2694* (MSB 006155), LC153873, LC154006; *Acantholimon macranthum* Rech.f. & Köie, Afghanistan, Bamian, Koh-e Baba, Kotal-e Shatu, *Podlech 19550* (M 0276210), LC153874, LC154007; *Acantholimon melananthum* Boiss., Iran, Kohgiluyeh & Boyer-Ahmad, Mt. Dena, *Assadi & Mozaffarian 31198* (TARI), LC153875, LC154008; *Acantholimon mirtadzadinii* Assadi, Iran, Kerman, Hanza, *Mirtadzadini 83220* (TARI), LC153876, -; *Acantholimon mishaudaghense* Mobayen, Iran, E Azarbaijan, Mishu Dağ, *Ghahramani et al. 6640* (TARI), LC153877, -; *Acantholimon modestum* Bornm. ex Rech.f., Iran, Kerman, Kuhbanan, *Mirtadzadini 86987* (TARI), AB979568, AB979628; *Acantholimon moradii* Assadi, Iran, Kurdistan, Sanandaj road, *Assadi 84925* (TARI), LC153878, -; *Acantholimon nabievii* Lincz., Kyrgyzstan, Chatkalski khrebet, Mt. Bozbutoo*, Lažkov s.n.* (W 2007-0004887), LC153879, LC154009; *Acantholimon nigricans* Mobayen, Iran, Yazd, Deh Bala village, *Assadi & Ranjbar 82847* (TARI), LC153880, LC154010; *Acantholimon oliganthum* Boiss., Iran, Fars, Shiraz, *Assadi & Mozaffarian 31402* (TARI), AB979570, AB979630; *Acantholimon olivieri* Boiss., Iran, Kurdistan, Kamyaran, *Assadi 60652* (TARI), LC153882, LC154011; *Acantholimon ophiocladum* Rech.f. & Schiman-Czeika, Iran, Kurdistan, Bijar road, *Yousefi 927* (TARI), LC153884, LC154013; *Acantholimon peculiare* Rech.f., Afghanistan, Panjao, *Köie 2723* (W 1965-0002643), LC153885, LC154014; *Acantholimon physostegium* Rech.f. & Schiman-Czeika, Afghanistan, Mukur, Agodhjan, *Freitag 3443* (W 1975-0014055), LC153887, -; *Acantholimon pterostegium* Bunge, Iran, Khorasan, Mashhad, Torghabeh, *Assadi 84499* (TARI), AB979571, AB979631; *Acantholimon pulchellum* Korovin, Kyrgyzstan, Tian-Shan Mts., Chatkalski khrebet, *Vašák s.n* (MA 642280), LC153888, LC154016; *Acantholimon quinquelobum* Bunge, Iran, Khorasan, Neyshabur, *Assadi & Mozaffarian 35440* (TARI), LC153889, LC154017; *Acantholimon raddeanum* Czernjak., Iran, Khorasan, Shirvan, *Faghihi & Zanguei* *18846* (FUMH), AB979573, AB979633; *Acantholimon restiaceum* Bunge, Iran, Khorasan, Torbat-e Jam, *Joharchi 34077* (FUMH), AB979574, AB979634; *Acantholimon revolutum* Rech.f. & Köie, Afghanistan, Kabul, Baba Qashkhar, *Podlech 15860* (M 0276215), LC153892, LC154018; *Acantholimon rhodopolium* Rech.f. & Schiman-Czeika, Iran, Hamadan, Mt. Ağdash, *Mozaffarian 64461* (TARI), AB979575, AB979635; *Acantholimon rudbaricum* Bornm., Iran, Gilan, Lushan, *Kazempour-Osaloo & Moharrek s.n.* (TMUH 92421), LC153894, LC154019; *Acantholimon sackenii* Bunge, Kyrgyzstan, Alai ridge, Gulcha river gorge, *Lažkov s.n.* (W 2007-0010495), LC153896, LC154021; *Acantholimon sahendicum* Boiss. & Buhse, Iran, Gilan, Damash to Jirandeh road, *Wendelbo 13523* (TARI), AB979576, -; *Acantholimon sarytavicum* Lincz., Kyrgyzstan, Turkestan range, Mt. Batkan, *Lažkov s.n.* (W 2007-04890), LC153897, LC154022; *Acantholimon scabrellum* Boiss. & Hausskn. ex Boiss., Iran, Fars, Ardekan to Yasuj road, *Assadi & Mozaffarian 31112* (TARI), LC153898, -; *Acantholimon schahrudicum* Bunge, Iran, Semnan, Damghan, *Assadi & Hamdi 85414* (TARI), AB979577, AB979636; *Acantholimon schirazianum* Boiss., Iran, Fars, Bambo National Park (TARI 32716), LC153899, -; *Acantholimon schizostegium* Rech.f. & Schiman-Czeika, Afghanistan, Ghorat, Parjuman, *Rechinger 19078* (W 1975-0013931), LC153900, -; *Acantholimon scirpinum* Bunge, Iran, Khorasan, Jaghargh Village, *Joharchi & Zanguei 41300* (FUMH), AB979578, AB979637; *Acantholimon scorpius* Boiss., Iran, Isfahan, Anarak, *Amin & Reymand 32957* (W 1986-0000093), LC153901, LC154023; *Acantholimon senganense* Bunge, Iran, Chahar Mahaal-e Bakhtiari, Tang-e Sayyad, *Mozaffarian 97176* (TARI), LC153906, LC154028; *Acantholimon serotinum* Rech.f. & Schiman-Czeika, Iran, Kerman, Baft, *Pourmirzae & Ghonchee 83211* (TARI), LC153909, LC154029; *Acantholimon sirchense* Assadi & Mirtadzadini, Iran, Kerman, Kuh-e Siah, *Mirtadzadini s.n.* (SHBU 1266), AB979581, AB979640; *Acantholimon solidum* Rech.f. & Köie, Afghanistan, Bamian, Darrah-e Godar, *Podlech 18948* (M 0276223), LC153910,LC154030; *Acantholimon spinicalyx* Köie & Rech.f., Iran, Kerman, Zarand, *Mirtadzadini 86984* (TARI), LC153912, LC154031; *Acantholimon stereophyllum* Rech.f. & Schiman-Czeika, Afghanistan, Ghorat, Chehelghazi, *Podlech 21869* (W 1974-0023252), LC153915, -; *Acantholimon subulatum* Boiss., Afghanistan, Maidan, Darrah-Syahsang*, Dieterle 695* (M 0276221), LC153916, LC154034; *Acantholimon takhtajanii* Ogan., Armenia, Kotayk, W Abovyan, Gegham Mts., *Vitek et al. 03-0746* (MA 742678), LC153917, LC154035; *Acantholimon talagonicum* Boiss., Iran, Tehran, Karaj, *Jamzad 57103* (TARI), LC153918, AB979642; *Acantholimon tomentellum* Boiss., Iran, Isfahan, Mt. Ghabri, *Assadi & Mozaffarian 31694* (TARI), AB979584, AB979643; *Acantholimon tragacanthinum* Boiss., Iran, W Azarbaijan, Urmia lake, *Assadi 86918* (TARI), AB979585, AB979644; *Acantholimon tricolor* Rech.f. & Köie, Afghanistan, Gardez, Safed Kuh, *Rechinger 31886* (MA 417521), LC153919, -; *Acantholimon ulicinum* Boiss., Greece, Krete, Chania, Levka Ori, *Merxmüller* & *Podlech 30886* (M 0276189), LC153920, LC154036; *Acantholimon venustum* Boiss., Turkey, Sivas, Doğançal, *Aldasoro et al. 2724* (MA 689899), LC153925, LC154040; *Acantholimon wendelboi* Rech.f. & Schiman-Czeika, Iran, Isfahan, *Assadi & Ranjbar 83046* (TARI), AB979586, AB979645; *Acantholimon wiedemanii* Bunge, Turkey, Kayseri, Erciyes Dağı, *Aedo et al. 6680* (MA 688346), LC153930, -; *Acantholimon zaeifii* Assadi, Iran, Kerman, Raber, *Mirtadzadini s.n.* (SHBU 1249), AB979587, AB979646; *Acantholimon zaprjagaevii* Lincz, Afghanistan, Kunar, Bashgal valley, *Podlech 16364* (M 0276224), LC153931, LC154045; *Armeria hirta* Pourr. ex Willk. & Lange, Spain, Cádiz, Arcos, *Nieto Feliner 3865GN* (MA 762586) AJ225571, -; *Armeria maderensis* Lowe, Portugal, Madeira, Areeiro peak, *Piñeiro 112RP10* (MA), LC153932, LC154046; *Armeria maritima* Willd., cultivated National Botanical Garden Iran, AB979588, AB979647; *Armeria pungens* Hoffmanns. & Link, Portugal, Alentejo, Bordeira, *Nieto Feliner 4457GN* (MA), LC153933, LC154047; *Armeria villosa* Girard subsp*. longiaristata* (Boiss. & Reut.) Nieto Fel., Spain, Córdoba, Sierra de la Lastra, *Nieto Feliner 4255GN* (MA), LC153934, LC154048; *Armeria welwitschii* Boiss., cultivated National Botanical Garden Iran, AB979589, AB979648; *Bamiania pachycorma* (Rech.f.) Lincz., Afghanistan, Bamian, Ajar Valley, *Hedge & Wendelbo 4103* (E), LC153935, LC154049; *Bukiniczia cabulica* (Boiss.) Lincz., Afghanistan, Farah, Hakumate Purchaman, *Podlech 21781* (M 0276239), LC153936, LC154050; *Cephalorhizum coelicolor* (Rech.f.) Rech.f., Afghanistan, Baghlan, Andarab Tal, Kaftarkhana, *Anders 9314* (MSB 006131), LC153938, LC154052; *Chaetolimon setiferum* (Bunge) Lincz., Kazakhstan, Tian-Shan Mts., Alym-tau Mts., *Neustruyeva et al. s.n.* (M), LC153940, -; *Dictyolimon griffithii* (Aitch. & Hemsl.) Rech.f., Afghanistan, Nuristan, Kunar Tal, *Podlech 16138* (MSB 006130), LC153941, -; *Dictyolimon macrorrhabdos* (Boiss.) Rech.f., Afghanistan, Kabul, Tang-e Gharu, *Podlech 30260* (MSB 006135), LC153942, LC154054; *Dyerophytum socotranum* J.R. Edmondson, Yemen, Socotra, *Miller et al. 10300* (E 0035670), LC153944, LC154056; *Gladiolimon speciosissimum* (Aitch. & Hemsl.) Mobayen, Afghanistan, Badghis, Kotale Sabzak, *Podlech 16945* (MSB 006125), LC153945, LC154057; *Goniolimon italicum* Tammaro, Pignatti & G.Frizzi, Appenines, Gran Sasso National Park, *Conti s.n.*, LC217873, -; *Goniolimon speciosum* Boiss. Russia, Altai Mts., *Castroviejo 14315SC* (MA 614247), LC217874, -; *Limonium axillare* Kuntze, Iran, Hormozagan, Bandar Abbas, *Maassoumi & Abu Hamzeh 52016* (TARI), LC153947, LC154059; *Limonium carnosum* Kuntze, Iran, E Azarbaijan, *Assadi 79082* (TARI), LC153948, LC154060*; Limonium gmelinii* Kuntze, Iran, Bojnord, *Memariani & Zanguei 41461* (FUMH), AB979591, AB979650; *Limonium iranicum* (Bornm.) Lincz., Iran, Khorasan, Kashmar, *Memariani & Akhani 39323* (FUMH), AB979592, AB979651; *Limonium meyeri* Kuntze, Iran, W Azarbaijan, *Kazempour-Osaloo s.n*. (TMUH 89213), AB979593, AB979652; *Limonium narbonense* Mill., Spain, *Roselló JAR 96132*, AJ222838, *-; Limonium nudum* Kuntze, Iran, Semnan, Damghan, *Assadi & Maassoumi 21019* (TARI), LC153950, LC154062; *Limonium otolepis* Kuntze, Iran, Fars, Maharlu lake, *Bukhari et al. 14843* (TARI), LC153951, -; *Limonium reniforme* (Girard) Lincz., Iran, Khorasan, Bojnord, *Joharchi & Zanguei 1484* (FUMH), AB979594, AB979653; *Limonium sogdianum* Ikonn.-Gal., Iran, Khorasan, Torbat-e Jam, *Joharchi 34168* (FUMH), AB979595, AB979654; *Limonium suffruticosum* Kuntze, Iran, Khorasan, Birjand, *Joharchi 35188* (FUMH), pending, AB979655; *Limonium vulgare* Hill, Spain, *Roselló* *JAR 96085*, AJ222839, -; *Plumbago europaea* L., Iran, Khorasan, Birjand, *Zanguei 38466* (FUMH), AB979599, AB979659; *Popoviolimon turcomanicum* (Popov ex Lincz.) Lincz., Iran, Khorasan, Mashhad to Sarakhs road, *Joharchi & Zanguei 36270* (FUMH), AB979590, AB979649; *Psylliostachys beludshistanica* Roshk., Iran, Khorasan, Kelateh bala village, *Faghihnia & Zanguei 18122* (FUMH), AB979596, AB979656; *Psylliostachys leptostachya* (Boiss.) Roshk., Iran, Khorasan, Sarakhs road, *Joharchi & Zanguei 14525* (FUMH), AB979597, AB979657; *Psylliostachys spicata* (Willd.) Nevski, Iran, Khorasan, Torbat-e Jam, *Zanguei 34446* (FUMH), AB979598, AB979658; *Psylliostachys suvorovii* (Regel) Roshk., cultivated Insbruck Botanical Garden, AJ132446, -; *Vassilczenkoa sogdiana* (Lincz.) Lincz., Afghanistan, Baghlan, *Podlech 21130* (M 0276244), LC153953, LC154063

TMUH: Tarbiat Modares University Herbarium, Tehran, Iran

HWANRC: Herbarium of West Azerbaijan Agricultural and Natural Resource Research Center
