# Supplementary material for: Morphological Innovations and Vast Extensions of Mountain Habitats Triggered Rapid Diversification Within the Species-Rich Irano-Turanian Genus Acantholimon (Plumbaginaceae)
Source: Front Genet. 2019 Jan 21;9:698. doi: 10.3389/fgene.2018.00698 (PMC6360523; doi:10.3389/fgene.2018.00698)
Supplement: Data Sheet S2 — XML file used for molecular phylogenetic dating of the complete dataset comprising 130 species from Acantholimon s.l. and 26 outgroup taxa, using the two-calibration point strategy (see main article). [file Data_Sheet_2.pdf]

```
<?xml version="1.0" standalone="yes"?>

<!-- Generated by BEAUTi v1.8.3 Prerelease r20160103
-->
<!--       by Alexei J. Drummond, Andrew Rambaut and Marc A.
Suchard         -->
<!--       Department of Computer Science, University of
Auckland and        -->
<!--       Institute of Evolutionary Biology, University of
Edinburgh        -->
<!--       David Geffen School of Medicine, University of
California, Los Angeles-->
<!--       http://beast.bio.ed.ac.uk/
-->
<beast>

     <!-- The list of taxa to be analysed (can also include
dates/ages).          -->
     <!-- ntax=156
-->
     <taxa id="taxa">
          <taxon id="Plumeuropa"/>
          <taxon id="DyerSoconu"/>
          <taxon id="Limvulgare"/>
          <taxon id="Limmeyerii"/>
          <taxon id="Limrenifoe"/>
          <taxon id="Limsuffruo"/>
          <taxon id="Limcarnos1"/>
          <taxon id="Limotoleps"/>
          <taxon id="Limiranicm"/>
          <taxon id="Limnudummm"/>
          <taxon id="Limnarbone"/>
          <taxon id="Limaxillar"/>
          <taxon id="Limsogdiam"/>
          <taxon id="Limgmelini"/>
          <taxon id="Psysuworou"/>
          <taxon id="Psyspicata"/>
          <taxon id="Psybeludsh"/>
          <taxon id="Psyleptosh"/>
          <taxon id="Armhirtaaa"/>
          <taxon id="Armeriamar"/>
          <taxon id="Armeriawel"/>
          <taxon id="Armeriamad"/>
          <taxon id="Armeriaspe"/>
          <taxon id="Armeriasin"/>
          <taxon id="Cephturcoc"/>
          <taxon id="Cephcoe32"/>
          <taxon id="Bamianiaaa"/>
          <taxon id="Dicgrif30"/>
          <taxon id="Dicmacr34"/>
          <taxon id="Bukcabu39"/>
          <taxon id="Vassizso44"/>
          <taxon id="ChaetolSet"/>
          <taxon id="Glaspeci25"/>
          <taxon id="Alavaeaaaa"/>
          <taxon id="Bodeanum1m"/>
```

<taxon id="scorpiu927"/>
<taxon id="Aspadanumm"/>
<taxon id="Bromifoliu"/>
<taxon id="Bracteatum"/>
<taxon id="Flexuosumm"/>
<taxon id="Pterostegi"/>
<taxon id="Cymosummmm"/>
<taxon id="Gilliatiii"/>
<taxon id="Quniquelob"/>
<taxon id="Scirpinumm"/>
<taxon id="Zaefii1iii"/>
<taxon id="Kermanense"/>
<taxon id="Chlorosteg"/>
<taxon id="Albocalyci"/>
<taxon id="Avenaceumm"/>
<taxon id="Modestum1a"/>
<taxon id="Oliganthum"/>
<taxon id="Blandummmm"/>
<taxon id="Raddeanumm"/>
<taxon id="Sirchensee"/>
<taxon id="Collareeee"/>
<taxon id="Heweriiiii"/>
<taxon id="Restiacumm"/>
<taxon id="Spinicaly1"/>
<taxon id="Gulistanum"/>
<taxon id="Austroiran"/>
<taxon id="Schahrudic"/>
<taxon id="Cephaltoid"/>
<taxon id="Demawendic"/>
<taxon id="Gorganense"/>
<taxon id="Heratensee"/>
<taxon id="Horridummm"/>
<taxon id="Rodopolium"/>
<taxon id="Tragacanth"/>
<taxon id="Atropatanu"/>
<taxon id="Sahendicu2"/>
<taxon id="Latifolium"/>
<taxon id="Asphodelin"/>
<taxon id="Acmostegi1"/>
<taxon id="Brachystac"/>
<taxon id="Caryophyll"/>
<taxon id="Cupreoliva"/>
<taxon id="Eschkerese"/>
<taxon id="Melananthu"/>
<taxon id="Mischodage"/>
<taxon id="Nigricanse"/>
<taxon id="Fominiiiii"/>
<taxon id="Olivieri1i"/>
<taxon id="Ophiocladu"/>
<taxon id="wendORO10k"/>
<taxon id="Serotinumo"/>
<taxon id="Curviflor1"/>
<taxon id="Densiflor1"/>
<taxon id="Hystrixxxx"/>
<taxon id="Karelinii1"/>
<taxon id="Rudbaricu1"/>

<taxon id="Mirtajadin"/>
<taxon id="Sacberllum"/>
<taxon id="Shirazianu"/>
<taxon id="Hormozgane"/>
<taxon id="Glabratum1"/>
<taxon id="Moradiiiii"/>
<taxon id="Tomentellu"/>
<taxon id="Hohenacer1"/>
<taxon id="Acanthob40"/>
<taxon id="ahangare92"/>
<taxon id="AuganumW86"/>
<taxon id="Cabulicu99"/>
<taxon id="Carinatu25"/>
<taxon id="Cephalot39"/>
<taxon id="erinaceu70"/>
<taxon id="fascicul45"/>
<taxon id="Festucas88"/>
<taxon id="Chitrali79"/>
<taxon id="gilliiW141"/>
<taxon id="inermeM004"/>
<taxon id="koeieiW153"/>
<taxon id="leucochl50"/>
<taxon id="lycopoio55"/>
<taxon id="macrathu10"/>
<taxon id="peculiar19"/>
<taxon id="physoste55"/>
<taxon id="pulchell79"/>
<taxon id="revolutu15"/>
<taxon id="sakeniW295"/>
<taxon id="schizost31"/>
<taxon id="senga85995"/>
<taxon id="solidum174"/>
<taxon id="stereop252"/>
<taxon id="subulat221"/>
<taxon id="Talagon103"/>
<taxon id="Zaprjaga24"/>
<taxon id="acerosum08"/>
<taxon id="alatavic81"/>
<taxon id="armenum647"/>
<taxon id="Takhtaj678"/>
<taxon id="aulieten67"/>
<taxon id="compact047"/>
<taxon id="diapens252"/>
<taxon id="ecae024754"/>
<taxon id="ekatheri80"/>
<taxon id="ekbergia42"/>
<taxon id="erythrae98"/>
<taxon id="federov277"/>
<taxon id="glumace690"/>
<taxon id="glutinos37"/>
<taxon id="hypochar7h"/>
<taxon id="knorri9KYG"/>
<taxon id="laxuW2055K"/>
<taxon id="leucant552"/>
<taxon id="nabievi887"/>
<taxon id="sarytavi90"/>

```
        <taxon id="tricol21AN"/>
        <taxon id="venustum99"/>
        <taxon id="ulicinum79"/>
        <taxon id="wiedema346"/>
        <taxon id="AcaAraxanu"/>
        <taxon id="AcaFlabeum"/>
        <taxon id="Balohani75"/>
        <taxon id="Gonitilcum"/>
        <taxon id="Gonispecoi"/>
    </taxa>
    <taxa id="crown">
        <taxon idref="AcaAraxanu"/>
        <taxon idref="AcaFlabeum"/>
        <taxon idref="Acanthob40"/>
        <taxon idref="Acmostegi1"/>
        <taxon idref="Alavaeaaaa"/>
        <taxon idref="Albocalyci"/>
        <taxon idref="Armeriamad"/>
        <taxon idref="Armeriamar"/>
        <taxon idref="Armeriasin"/>
        <taxon idref="Armeriaspe"/>
        <taxon idref="Armeriawel"/>
        <taxon idref="Armhirtaaa"/>
        <taxon idref="Aspadanumm"/>
        <taxon idref="Asphodelin"/>
        <taxon idref="Atropatanu"/>
        <taxon idref="AuganumW86"/>
        <taxon idref="Austroiran"/>
        <taxon idref="Avenaceumm"/>
        <taxon idref="Balohani75"/>
        <taxon idref="Bamianiaaa"/>
        <taxon idref="Blandummmm"/>
        <taxon idref="Bodeanum1m"/>
        <taxon idref="Brachystac"/>
        <taxon idref="Bracteatum"/>
        <taxon idref="Bromifoliu"/>
        <taxon idref="Bukcabu39"/>
        <taxon idref="Cabulicu99"/>
        <taxon idref="Carinatu25"/>
        <taxon idref="Caryophyll"/>
        <taxon idref="Cephalot39"/>
        <taxon idref="Cephaltoid"/>
        <taxon idref="Cephcoe32"/>
        <taxon idref="Cephturcoc"/>
        <taxon idref="ChaetolSet"/>
        <taxon idref="Chitrali79"/>
        <taxon idref="Chlorosteg"/>
        <taxon idref="Collareeee"/>
        <taxon idref="Cupreoliva"/>
        <taxon idref="Curviflor1"/>
        <taxon idref="Cymosummmm"/>
        <taxon idref="Demawendic"/>
        <taxon idref="Densiflor1"/>
        <taxon idref="Dicgrif30"/>
        <taxon idref="Dicmacr34"/>
        <taxon idref="DyerSoconu"/>
```

<taxon idref="Eschkerese"/>
<taxon idref="Festucas88"/>
<taxon idref="Flexuosumm"/>
<taxon idref="Fominiiiii"/>
<taxon idref="Gilliatiii"/>
<taxon idref="Glabratum1"/>
<taxon idref="Glaspeci25"/>
<taxon idref="Gonispecoi"/>
<taxon idref="Gonitilcum"/>
<taxon idref="Gorganense"/>
<taxon idref="Gulistanum"/>
<taxon idref="Heratensee"/>
<taxon idref="Heweriiiii"/>
<taxon idref="Hohenacer1"/>
<taxon idref="Hormozgane"/>
<taxon idref="Horridummm"/>
<taxon idref="Hystrixxxx"/>
<taxon idref="Karelinii1"/>
<taxon idref="Kermanense"/>
<taxon idref="Latifolium"/>
<taxon idref="Limaxillar"/>
<taxon idref="Limcarnos1"/>
<taxon idref="Limgmelini"/>
<taxon idref="Limiranicm"/>
<taxon idref="Limmeyerii"/>
<taxon idref="Limnarbone"/>
<taxon idref="Limnudummm"/>
<taxon idref="Limotoleps"/>
<taxon idref="Limrenifoe"/>
<taxon idref="Limsogdiam"/>
<taxon idref="Limsuffruo"/>
<taxon idref="Limvulgare"/>
<taxon idref="Melananthu"/>
<taxon idref="Mirtajadin"/>
<taxon idref="Mischodage"/>
<taxon idref="Modestum1a"/>
<taxon idref="Moradiiiii"/>
<taxon idref="Nigricanse"/>
<taxon idref="Oliganthum"/>
<taxon idref="Olivieri1i"/>
<taxon idref="Ophiocladu"/>
<taxon idref="Plumeuropa"/>
<taxon idref="Psybeludsh"/>
<taxon idref="Psyleptosh"/>
<taxon idref="Psyspicata"/>
<taxon idref="Psysuworou"/>
<taxon idref="Pterostegi"/>
<taxon idref="Quniquelob"/>
<taxon idref="Raddeanumm"/>
<taxon idref="Restiacumm"/>
<taxon idref="Rodopolium"/>
<taxon idref="Rudbaricu1"/>
<taxon idref="Sacberllum"/>
<taxon idref="Sahendicu2"/>
<taxon idref="Schahrudic"/>
<taxon idref="Scirpinumm"/>

<taxon idref="Serotinumo"/>
<taxon idref="Shirazianu"/>
<taxon idref="Sirchensee"/>
<taxon idref="Spinicaly1"/>
<taxon idref="Takhtaj678"/>
<taxon idref="Talagon103"/>
<taxon idref="Tomentellu"/>
<taxon idref="Tragacanth"/>
<taxon idref="Vassizso44"/>
<taxon idref="Zaefii1iii"/>
<taxon idref="Zaprjaga24"/>
<taxon idref="acerosum08"/>
<taxon idref="ahangare92"/>
<taxon idref="alatavic81"/>
<taxon idref="armenum647"/>
<taxon idref="aulieten67"/>
<taxon idref="compact047"/>
<taxon idref="diapens252"/>
<taxon idref="ecae024754"/>
<taxon idref="ekatheri80"/>
<taxon idref="ekbergia42"/>
<taxon idref="erinaceu70"/>
<taxon idref="erythrae98"/>
<taxon idref="fascicul45"/>
<taxon idref="federov277"/>
<taxon idref="gilliiW141"/>
<taxon idref="glumace690"/>
<taxon idref="glutinos37"/>
<taxon idref="hypochar7h"/>
<taxon idref="inermeM004"/>
<taxon idref="knorri9KYG"/>
<taxon idref="koeieiW153"/>
<taxon idref="laxuW2055K"/>
<taxon idref="leucant552"/>
<taxon idref="leucochl50"/>
<taxon idref="lycopoio55"/>
<taxon idref="macrathu10"/>
<taxon idref="nabievi887"/>
<taxon idref="peculiar19"/>
<taxon idref="physoste55"/>
<taxon idref="pulchell79"/>
<taxon idref="revolutu15"/>
<taxon idref="sakeniW295"/>
<taxon idref="sarytavi90"/>
<taxon idref="schizost31"/>
<taxon idref="scorpiu927"/>
<taxon idref="senga85995"/>
<taxon idref="solidum174"/>
<taxon idref="stereop252"/>
<taxon idref="subulat221"/>
<taxon idref="tricol21AN"/>
<taxon idref="ulicinum79"/>
<taxon idref="venustum99"/>
<taxon idref="wendORO10k"/>
<taxon idref="wiedema346"/>
</taxa>

```
      <taxa id="stem">
            <taxon idref="Armeriamad"/>
            <taxon idref="Armeriamar"/>
            <taxon idref="Armeriasin"/>
            <taxon idref="Armeriaspe"/>
            <taxon idref="Armeriawel"/>
            <taxon idref="Armhirtaaa"/>
            <taxon idref="Psybeludsh"/>
            <taxon idref="Psyleptosh"/>
            <taxon idref="Psyspicata"/>
            <taxon idref="Psysuworou"/>
      </taxa>

      <!-- The sequence alignment (each sequence refers to a
taxon above).          -->
      <!-- ntax=156 nchar=731
-->
      <alignment id="alignment1" dataType="nucleotide">
            <sequence>
                  <taxon idref="Plumeuropa"/>
                  TCGAAAACTGC--ACAG---AACGACCCGAG--AACCCGTTCCTT-
ACACCCACT-TACCTCGCCGGGCGCGTCGGCGGAGCT---------------
TCCGCGCCGT-CCGCCGCCCCGGCTTGAACAA---AACCC-
GGCGCGGGCCGCGCCAAGGACGAATCAAACTGTTGAAAAACCCT--GCCGCCCTCCCCTCC-
GTCCTTTGGACGGGGCG-----GGCGGCG--TCC-GG-TTGGCAGA--
CACGATA------------ACAATAAACGAC--
TCTCGACAACGGATATCTCGGCTCTCGCATCGATGAAGAACG--TAGCGAAAT---
GCGATACTTGGTGTGAATTGCA-GAATCCCGTGAACCATCGA---
GTCTTTGAACGCAAGTT-G--
CGCCCGAAGCCTTCTGGCCGAGGGCACGTCTGCCTGGGCGTCATGCATCGCG--
TCGCCGCCCCC-G-GACACCCGAC-----------CGGGG--G-CGGC-
GTAGTGTGGCCTCCCGCGCGCTT-CCGTGCGCGGCCGGCTTAAATTGGGGGCTT---
GCGGCAT--CGAGCGCCGTGGCAGTTGGTGG-TTTGTAAGGCCGTCGC-AGGCCGGAG---
GACATC----GTGCCGCGACGCGCCGTGCC-GCTCGA--GCCCGTATGACCCTGAGGAC--
GCCGCGCGAGCCCAGCGCTCGGGCGGC------CGATCCAC-CCA
            </sequence>
            <sequence>
                  <taxon idref="DyerSoconu"/>
                  TCGAAACCTCTCAACAG---GACAACTCGAG--
AACCCGTTTTTTCACYAACCAT----
CTTGTCGGGAAYGGGCGGCCTGGCGCCR----------------TCCGT-CCTT-CG----
GCTTGAACAA---AAACC-CGGCGCGGGTTGCGCCAAGGACYAATGAA-TGTTGAA---
CCTT--GCCTGCCCTMCCTYG-GTC-TTTYGGCTGGGGG-----AGGCGGC--AGTCGG-
TCGGCAAA--AAYGCAA------------ACTATTAACGAC--
TCTCGACAACGGATATCTCGGCTCTCGCATCGATGAAGAACG--TAGCGAAAT---
GCGATACTTGGTGTGAATTGCA-GAATCCCGTGAACCATCGA---
GTCTTTGAACGCAAGTT-G--
CGCCCGAAGCCTTCTGGCCGAGGGCACGTCTGCCTGGGCGTCATGCATCGCG--
TCGCCACCCC--A-GCCACCCTAACCCAGTGGACTGAGGG--G-CG-C---
AATGTGGCCGTCCGTGCGCAT-GCACGCGCGGTCGGCTGAAATACGGGGCTT---
GCGGCAA--CGAGCGCCGCGGCAGTTGGTGG-TTTTTTGAAGGCCTTG-
AGGCCAGAGACGCACATC----GTGCCGCGATGCGCCGTGCCTAACACA--
GCCCGTACGACCC-AAGTGT--------------------------------------
TCCAC-CCA
            </sequence>
            <sequence>
```

<taxon idref="Limvulgare"/>
TCGAAAACTGC-ACAGTTA-AGCCACATGTT--AACTTGTTTTTT-
AACCATTCC--TTGTTGCTGGTAGGAA-GGTTGC-CT---------------TGGC-
TGCCT-CCTT-CCATC-ACATGAACAA---TTTTC-
GGCGCGGAATGCGTCAAGGAGTACAGACATTAACGTAAGGCTAG--CCC-----TCTTGCT-
GCCGGTTGGTTTTGATG-----AG-GGTG--TG--GCATTGTTGAA--
ATATT--------------TGACTTAATGAC--
TCTTGGCAACGGATATCTCGGCTCTTGCATCGATGAAGAACG--TAGCGAAAT---
GCGATACTTGGTGTGAATTGCA-RAATCCCGTGAACCATCGA---
GTCTTTGAACGCAAGTT-
GCGCCTGAAGCTATTTGGCCGAGGGCACGTCTTCCTGGGTGTCATACTTTGCGCTGCCCCCC
TC-T-CCCTGTTATTCTGTGG----ATGAGA--T-GGGC-
GGAGATTGGTCTCCTATGTGT---CTT-GCGCACATTGGTGTCTTAAACATGGAGCTT---
GATGTAG--TGAGCACCGTGATGATCGGTGG-TTGGAT-GGGCCTTTC-GCCCTAGA----
AGCATC----TTGTCAGGAAGTGTCGTGCT-GAGTTC--GGTAGGAGGATTTGAATTGG--
TCTGAATTCTATGTGAATAAATTGCTATGACTTGTGTCAAC-AGT
</sequence>
<sequence>
<taxon idref="Limmeyerii"/>
TCGAAAACTGC-ACAGTTA-AGCCACATGTT--AACTTG-TTTTT-
AACCATTCC--TTGTTGCTGGTAGGAT-GGTTGC-CT---------------TGGC-
TGCCT-CCTT-CCATC-GCATGAACAA---TTTTC-
GGCGCGGAATGCGTCAAGGAGTACAGACATTAACGTAAGGCTAG--CCC-----TCTTGCT-
GCCGGTTGGTTTTGATG-----AG-GGTG--TT--GCATTGTTGAA--
ATATT--------------TGACTTAATGAC--
TCTTGGCAACGGATATCTCGGCTCTTGCATCGATGAAGAACG--TAGCGAAAT---
GCGATACTTGGTGTGAATTGCA-GAATCCCGTGAACCATCGA---
GTCTTTGAACGCAAGTT-
GCGCCTGAAGCTATTTGGCTGAGGGCACGTCTTCCTGGGTGTCATACTTTGCGCTGCCCCCC
TC-T-CCCTGTTATTGTGTGG----ATGAGA--T-GGGC-
GGAGATTGGTCTCCTATGTGT---CTT-GCGCACATGGGTGTCTTAAACATGGAGCTT---
GATGTAG--TGAGCACCGTGATGATCGGTGG-TTGGAT-GGGCCTTTC-GCCCTAGA----
AGCATC----TTGTCAGGAAGTGTCGTGCT-GAGTTC--GGTAGGAGGATTTGAATTGG--
TCTGAATTCTGTGTGAATAAATTGCTATGACTTGTGTCAAC-AGT
</sequence>
<sequence>
<taxon idref="Limrenifoe"/>
TCGAAAACTGC-ACAGTTA-AGCCACATGCT--AACTTGTTTTTT-
AAAAATTCC--TTGTTGTTTGTAGGAA-GGTTGC-CT---------------TGGC-
TGCCT-CCTG-CGATG-ACATGAAAAA---ATTTC-
GGCGCGGAATGCGTCAAGGAAAATAGACATTAATG-AAGGCTAG--CCC-----TATTGTT-
GCTGTTTTGTTGCATTT-----AG-GGTG--TT--GCGTCGTTGAA--
ATATATT------------TGGTTGAATGAC--
TCTCGGCAACGGATATCTCGGCTCTCGCATCGATGAAGAACG--TAGCGAAAT---
GCGATACTTGGTGTGAATTGCA-GAATCCCGTGAACCATCGA---
GTCTTTGAACGCAAGTT-
GCGCCTGAAGCTATTTGGCTGAGGGCACGTCTTCCTGGGTGTCATTCTCTGCGCTG-
CCCCTTC-A-CCCTCCTACTGTGTCG----ATGTGA--GTGGGC-
GGAGATTGGTCTCCTATGTGT---CTT-GCGCACATGGGTGTCTTAAACACGGAGCTT---
GATGTGG--TGAGCACTGTGATGATCGGTGG-TTGAAT-GGGCCTTTA-GCCCTAGA----
AGCATC---TTTGTCAGGAAGTGTCGTGCT-GAGATT--GCTACAAGGATTTGAATTGG--
TTTGAAATCATTGTGAATAAATTGGTATGACTTGTGTCAAC-AGT
</sequence>
<sequence>
<taxon idref="Limsuffruo"/>
TCGAAAACTGC-ACAGTTA-AGCCACACGTT--AACTTGTTTTTT-

AACCATTCC--ATGTTGCTGGTAGGAA-GGTTGC-CG--------------TGGC-
TTCCT-CCTT-CCRTC-GCATGAACAACAATTTCC-
GGCGCGGAATGCGTCAAGGAGTACAGACATTMATGTAAGGCTAG--CCCCC---TCTTGCT-
GCCGTTTGGTTTTGATG-----AG-GGTGTGTA--GCATAGTTGAA--
ATATT-------------TGGCTTAATGAC--
TCTTGGCAACGGATATCTCGGCTCTTGCATCGATGAAGAACG--TAGCGAAAT---
GCGATACTTGGTGTGAATTGCA-GAATCCCGTGAACCATCGA---
GTCTTTGAACGCAAGTT-
GCGCTTGAAGCTATTTGGCTGAGGGCACGTCTTCCTGGGTGTCATACTTTGCGCTGCCCCCC
TC-TCCCCCCTTATTGTGTGG----ATGAGA--G-GGGC-
GGAGATTGGTCTCCTATGTGT---CTT-GCGCACATGGGTGTCTGAAACATGGAGCTT---
GACGTAG--TGAGCGCCGTGATGATCGGTGG-TTGGAT-GGGCCTTTC-GCCCTAGA----
AGCATC----TTGTCAGGAAGTGCCGTGCT-GAGTTC--GGTAGGAG--TTTGAATTGG--
TTTGAATTCTATGTGAATAAATTGCTATGACTT--GTCAAC-ATT
</sequence>
<sequence>
<taxon idref="Limcarnos1"/>
TCGAAAACTGC-ACAGTTA-AGCCACACGTG--AACTTGTTTTTT-
AACCATTCC--ATGCTGCTGGTAGGAA-GGTTGC-CC--------------TGGC-
TTCCT-CCTT-CCATC-GCATGAACAACAATTTTC-
GGCGCGGAATGCGTCAAGGAGTACAGAGATTAATGTAAGGCTAG--CCCCC---TCTAGCT-
GCCGTTTGGTTTTGATG-----AG-GGTG--TA--GCATAGTTGAA--
ATATT-------------TGGCTTAATGAC--
TCTTGGCAACGGATATCTCGGCTCTTGCATCGATGAAGAACG--TAGCGAAAT---
GCGATACTTGGTGTGAATTGCA-GAATCCCGTGAACCATCGA---
GTCTTTGAACGCAAGTT-
GCGCTTGATGCTATTTGGCTGAGGGCACGTCTTCCTGGGTGTCATACTTTGCGCTGCCCCCC
TC-TCCCCCCTTATTGTGTGG----ATGAGA--G-GGGC-
GGAGATTGGTCTCCTATGTGT---CTT-GCGCACATGGGTGTCTGAAACATGGAGCTT---
GACGTAG--TGAGCGCCGTGATGATCGGTGG-TTGGAT-GGGCCTTTC-GCCCTAGA----
AGCATC----TTGTCAGGAAGTGCCGTGCT-GAGTTC--GGTAGGAG--TTTGAATTGG--
TTTGAATTCTATGTGAATAAATTGCTATGACTT--GTCAAC-ATT
</sequence>
<sequence>
<taxon idref="Limotoleps"/>
TCGAAAACTGC-ACGCTTA-
AGCCACACATGCTAACTTGTTTTTTTAAAAATTCC--TTGCTGTTTGTAGGAA-GGCTGC-
CT--------------TGGC-TGCCT-CCTG-TGACG-ACATGAAAAA---ATTTC-
GGCGCGGAATGCGTCAAGGAAAATAGACATTAATG-AAGGCTAG--CCC-----TCTTGTT-
GCTGTTTTGTTGCGTTT-----AG-GGTG--TC--GCGTCGTTGAA--
ATATATT------------TGGTTGAATGAC--
TCTCGGCAACGGATATCTAGGCTCTTGCATCGATGAAGAACG--TAGCGAAAT---
GCGATACTTGGTGTGAATTGCA-GAATCCCGTGAACCATCGA---
GTCTTTGAATGCAAGTT-
GCGCCTCAAGCTATTTGGCTGAGGGCACGTCTTCCTGGGTGTCATTCTCTGCGCTG-
CCCCTTC-A-CCCTCCTACTGTGTTG----ATGTGA--GTGGGC-
GGAGATTGGTCTCCTATGTGT-C-TTT-
GCGCACATGGGTGTCTTAAACACGGAGCTTTTTGATGTAG--
TGAGCACTGTGATGATCGGTGG-TTGATT-GGGCCTTTA-GCCCTAGA----AGCAGA----
TTGTCAGGAAGTGTTGTGCT-GAGATT--
GCTACGAGGATTTGAATTGGGGTTTGAAATCAATGTGAATAAATTGGTATGATTT--
GTCAAC-AGT
</sequence>
<sequence>
<taxon idref="Limiranicm"/>
TCGAAAACTGC-ACAGTTA-AGCCACACGTT--AACTTGTTTTTT-

AACCATTCC--ATGTTGCTGGTAGGAA-GGTTGC-CT---------------TGGC-
TTCCT-CCTT-CCATC-GCATGAACAACAATTTTC-
GGCGCGGAATGCGTCAAGGAGTACAGACATTAATGTAAGGCTAG--CCCCC---TCTTGCT-
GCCGTTTGGTTTTGATG-----AG-GGTG--TA--GCATAGTTGAA--
ATATT--------------TGGCTTAATGAC--
TCTTGGCAACGGATATCTCGGCTCTTGCATCGATGAAGAACG--TAGCGAAAT---
GCGATACTTGGTGTGAATTGCA-GAATCCCGTGAACCATCGA---
GTCTTTGAACGCAAGTT-
GCGCTTGAAGCTATTTGGCTGAGGGCACGTCTTCCTGGGTGTCATACTTTGCGCTGCCCCCC
TC-TCCCCCCTTATTGTGTGG----ATGAGA--G-GGGC-
GGAGATTGGTCTCCTATGTGT---CTT-GCGCACATGGGTGTCTGAAACATGGAGCTT---
GATGTAG--TGAGCACCGTGATGATCGGTGG-TTGGAT-
GGGCCTTT--

</sequence>
<sequence>
<taxon idref="Limnudummm"/>
TCGAAAACTGC-ACAGTTA-AGCCACATGTT--AACTTG-TTATT-
AACCATTCC--ATGTTGTTTGTAGGAA-GGTTGC-CT---------------TGGC-
TCCGT-CGTA-CGATG-ACATGAACAA--TTTTTC-
GGCGCGGAATGCGTCAAGGAAAACCAACATTAACGTAAGGCTAG--CCC-----TCTTGCT-
GCTGTTTGGTTGTATGGAGTGGAG-GGTG--TT--GCATCGTTGAA--
ATATA--------------TGGTTAAATGAC--
TCTCGGCAACGGATATCTCGGCTCTTGCATCGATGAAGAACG--TAGCGAAAT---
GTGATACTTGGTGTGAATTGCA-GAATCCCGTGAACCATCGA---
GTCTTTGAACGCAAGTT-
GCGCCTGAAGCTATTTGGCTGAGGGCATGTCTTCCTGGGTGTCATACTTTATGCTG-
CCCCTTC-T-CCCTCCAACTGTGTGG----ATGAGA--G-GGGC-
GAAGATTGGTCTCCTATGTGT-C-CCC-GTGCACATAGGTGTCTTAAACATGGAGCTT---
GATGTAG--TGAGCACTGTGATGATTGGTGG-TTGGAT-GGGCTTTTT-GCCC-AGA----
AGCATC----TTGTCAGGAAGTGCCGTGCT-GAGTTTT-GCTAGAATGATTTGAATTGG--
TTTGAATTCAATGTGAATAAATTGCTATGATTT--GACAAC-AGT
</sequence>
<sequence>
<taxon idref="Limnarbone"/>
TCGAAAACTGC-ACAGTTA-AGCCACATGTT--AACTTGTTTTTT-
AACCATTCC--TTGTTGCTGGTAGGAA-GGTTGC-CT---------------TGGC-
TGCCT-CCTT-CCATC-GCATGAACAA---TTTTC-
GGCGCGGAATGCGTCAAGGAGTACAGACATTAACGTAAGGCTAG--CCC-----TCTTGCT-
GCCGGTTGGTTTTGATG-----AG-GGTG--TT--GCATTGTTGAA--
ATATT--------------TGACTTAATGAC--
TCTTGGCAACGGATATCTCGGCTCTTGCATCGATGAAGAACG--TAGCGAAAT---
GCGATACTTGGTGTGAATTGCAGGAATCCCGTGAACCATCGA---
GTCTTTGAACGCAAGTTGGCGCCTGAAGCTATTTGGCTGAGGGCACGTCTTCCTGGGTGTCA
TACTTTGCGCTGCCCCCCTC-T-CCCTGTTATTGTGTGG----ATGAGA--T-GGGC-
GGAGATTGGTCTCCTATGTGT---CTT-GCGCACATGGGTGTCTTAAACATGGAGCTT---
GATGTAG--TGAGCACCGTGATGATCGGTGG-TTGGAT-GGGCCTTTC-GCCCTAGA----
AGCATC----TTGTCAGGAAGTGACGTGCT-GAGTTC--GGTAGGAGGATTTGAATTGG--
TCTGAATTCTATGTGAATAAATTGCTATGACTTGTGTCAAC-AGT
</sequence>
<sequence>
<taxon idref="Limaxillar"/>
TCGAAAGCTGC-ACAGTTA-AGCCACTTGTT--AACCTGTTTTTT-
AACTTCTCC--TTGTTACTGGTGGGAA-GGCCGC-CC---------------CGGC-
AGCCT-CGCA-CCATG-ACATGAACAA---TTTTC-

GGCGCGGAATGCGTCAAGGAGTATGGACATTGATGTAAGACTAG--CCC-----TCTTGCT-
GCCGCTTGGTTGTGTTG-----AG-GGTG--TTTCGCATCGTTGAA--
GTTTA--------------TGGTCTAATGAC--
TCTCGGCAACGGATATCTCGGCTCTTGCATCGATGAAGAACG--TAGCGAAAT---
GCGATACTTGGTGTGAATTGCA-GAATCCCGTGAACCATCGA---
GTCTTTGAACGCAAGTT-
GCGCCTGAAGCTATTTGGCTGAGGGCACGTCTTCCTGGGTGTCATGCTTTGTGTTG-
CCCCCTT-A-CCCTCCTATCATGCGG----ATGAGA--G-GGGC-
GGAGATTGGTCTCCTATGTGT---CTT-GCGCACATGGGTGTCTTGAATGTGGAGCTT---
CATGAG---TGAGCACTGTTGATGATCGGTGGTTGGAT-GGGCCTTGC-GCCCTAGA----
AGAAGC-ATCTTGTTAGGAAGTGTCGCTCG-GAGTTT--GCTAGCTGGATTTGAATTGG--
TTTGAATTCTAGGTGAATAAA--ACTATGA-TT-CGTCAACGGGT
</sequence>
<sequence>
<taxon idref="Limsogdiam"/>
TCGAAAACTGC-ATAGTTA-AGCCACATGTT--TACTTG-TTTTT-
AACCATTCC--TTGTTGTTTGTAGGAA-GGCTGC-CT---------------TGGC-
TGCCT-CCTA-CGATG-ACATGAACAA---TTTTC-
GGCGCGGAATGCGTCAAGGAAAACAGTCATTAACGTAAGGCTAG--CCCC----TCTTGCT-
GCTGTTTGGTTGTCTTG-----AG-GGTG--TT--GCATCGTTGAA--
ATATT--------------TGGTTTAATGAC--
TCTCGGCAACGGATATCTCGGCTCTTGCATCGATGAAGAACG--TAGCGAAAT---
GCGATACTTGGTGTGAATTGCA-GAATCCCGTGAACCATCGA---
GTCTTTGAACGCAAGTT-
GCGCCTGAAGCTATTTGGCTGAGGGCACGTCTTCCTGGGTGTCATACTTTGCGCAGCCCCCC
TC-A-CCCTTCCACAACGTGG----ATGAGA--G-GGGC-
GGAGATTGGTCTCCTATGTGA---CTT-GTGCACATGGGTGTCTTAAACATGGAGCTT---
GATGCAG--TGAGCACTGTGATGAATGGTGG-TTGGAT-GGGCCTTGT-GCCCTAGA----
AGCATC----TTGTCAGGAAGTGTCGTGCT-
GAGTT---

</sequence>
<sequence>
<taxon idref="Limgmelini"/>
TCGAAAACTGC-ACAGTTA-AGCCACATGTT--AACTTGTTTTTT-
AACCATTCC--TTGTTGCTGGTAGGAA-GGTTGC-CT---------------TGGC-
TTCCT-CCTT-CCATC-GCATGAACAA---TTTTC-
GGCGCGGAATGCGTCAAGGAGTACAGACATTAACGTAAGGCTAG--CCC-----TCTTGCT-
GCCGGTTGGTTTTGATG-----AG-GGTG--TT--GCATTGTTGAA--
ATATT--------------TGACTTAATGAC--
TCTTGGCAACGGATATCTCGGCTCTTGCATCGATGAAGAACG--TAGCGAAAT---
GCGATACTTGGTGTGAATTGCA-AAATCCCGTGAACCATCGA---
GTCTTTGAACGCAAGTT-
GCGCCTGAAGCTATTTGGCTGAGGGCACGTCTTCCTGGGTGTCATACTTT-----------
----

</sequence>
<sequence>
<taxon idref="Psysuworou"/>
TCGAAACCTGCAATAGCAG-AGCCACACGTG--AACTTGT-TTTT-
AAACCTTCT--TTGTCGTGCGATGGAT-GGTTGC-CG-------------CGTGCC-
TCCTC-CATT-GC-CG-GCATGAACAA---TTTCC-GGCGCGGAATGCGCCAAGGA------
CTATGAACG-TAAGCTTC--ACC-----ATCTCGC-GCCGACTGGTTGCGTTG-----AG-

G-TG--TT--GCGTCGTTG-A--AAATA-------------TAATCGAATGAC--
TCTCGGCAACGGATATCTCGGCTCTCGCATCGATGAAGAACG--TAGCGAAAT---
GCGATACTTGGTGTGAATTGCA-GAATCCCGTGAACCATCGA---
GTCTTTGAACGCAAGTT-
GCGCCCGAAGCCTTTTGGCCGAGGGCACGTCTGCCTGGGCGTCACGCATCGCGTCGCTCC--
CC-A-CCCATCTTCGGTTGGG-----TTAGA--G-GGGC-
GGAGAGTGGTCTCCCGTGTGCTT-CGT--CTTGCACGGATGGCCTAAAAATGGAGCTT---
GCAGCAG--TGAGCGCCGTGATGATTGGTGG-TTGGAC-GGGT-TTCG-GCCCTAGA----
AGCATC----TCATCACGAGGCGTCGGGCA--TGCAT--GCTTGGTTGACTTGACCATT--
TCGGTGTCCTTGCGACTAAG-TCGCTT-TTGCATCGTCCAC-CCT
</sequence>
<sequence>
<taxon idref="Psyspicata"/>
TCGAAACCTGCAATAGCAG-AGCCACACGTG--AACTTGT-TTTT-
AAACCTTCT--TTGTCGTGTGATGGAT-GGTTGC-CG------------CGTGCC-
TCCTC-CATT-GC-CG-GCATGAACAA---TTTCC-GGCGCGGAATGCGCCAAGGA------
CTATGAACG-TAAGCTTC--ACC-----ATCTCGC-GCCGACTGGTTGCGTTG-----AG-
G-TG--TT--GCGTCGTTG-A--AAATA-------------TAATCGAATGAC--
TCTCGGCAACGGATATCTCGGCTCTCGCATCGATGAAGAACG--TAGCGAAAT---
GCGATACTTGGTGTGAATTGCA-GAATCCCGTGAACCATCGA---
GTCTTTGAACGCAAGTT-
GCGCCCGAAGCCTTTTGGCCGARGGCACGTCTGCCTGGGCGTCACGCATCGCGTCGCTCC--
CC-A-CCCATCTTCGTTTGGG-----TTAGA--G-GGGC-
GGAGAGTGGTCTCCCGTGTGCTT-CGT--CTTGCACGGATGGCCTAAAAATGGAGCTT---
GCGGCAG--TGAGCGCCGTGATGATTGGTGG-TTGGAC-GGGT-TTCG-GCCCTAGA----
AGCATC----TCATCACGAGGCGTCGGGCA--TGCAT--GCTTGGTTGACTTGACCATT--
TCGGTGTCCTTGCGACTAAG-TCGCTT-TTGCATCGTCCAC-CCT
</sequence>
<sequence>
<taxon idref="Psybeludsh"/>
TCGAAACCTGCAATAGCAG-AGCCACACGTG--AACTTGT-TTTT-
AAACCTTCT--TTGTCGTGCGATGGAT-GGCTGC-CG------------CGTGCC-
TCCTC-CATT-GC-CG-GCATGAACAA---TTTCC-GGCGCGGAATGCGCCAAGGA------
CTATGAACG-TAAGCTTC--ACC-----ATCTCGC-GCCGACTGGTTGCGTTG-----AG-
G-TG--TT--GCGTCGTTGAA--AAATA-------------TAATCGAATGAC--
TCTCGGCAACGGATATCTCGGCTCTCGCATCGATGAAGAACG--TAGCGAAAT---
GCGATACTTGGTGTGAATTGCA-GAATCCCGTGAACCATCGA---
GTCTTTGAACGCAAGTT-
GCGCCCGAAGCCTTTTGGCCGAGGGCACGTCTGCCTGGGCGTCACGCATCGCGTCGCTCC--
CC-A-CCCATCTTCGGTTGGG-----TTAGA--G-GGGC-
GGAGAGTGGTCTCCCGTGTGCTT-CG--ACTTGCACGGATGGCCTAAAAATGGAGCTT---
GCGGCAG--TGAGCGCCGTGATGATTGGTGG-TTGGAC-GGGT-TTCG-GCCCTAGA----
AGCATC----TCATCACGAGGCGTCGGGCA--TGCAT--GCTTGGTTGACTTGACCATT--
TCGATGTCATTGCGAATAAG-TCGC-T-TTGCATCGTCCAC-CCT
</sequence>
<sequence>
<taxon idref="Psyleptosh"/>
TCGAAACCTGCAATAGCAG-AGCCACACGTG--AACTTGT-TTTT-
AAACCTTCT--TTGTCGCGCGATGGAT-GGTTGC-CA------------CGTGCC-
TCCTC-CATT-GC-CG-GCATGAACAA---TTTCC-GGCGCGGAATGCGCCAAGGA------
CTATGAACG-TAAGCTTC--ACC-----ATCTCGT-GCCGACTGGTTGCGTTG-----AG-
G-TG--TT--GCGTCGTTGAA--AAATA-------------TAATCGAATGAC--
TCTCGGCAACGGATATCTCGGCTCTCGCATCGATGAAGAACG--TAGCGAAAT---
GCGATACTTGGTGTGAATTGCA-GAATCCCGTGAACCATCGA---
GTCTTTGAACGCAAGTT-
GCGCCCGAAGCCTTTTGGCCGAGGGCACGTCTGCCTGGGCGTCACGCATCGCGTCGCTCC--

CC-A-CCCAGCTTTGGTTGGG-----TTCAA--G-GGGC-
GGAGAGTGGTCTCCCGTGTGCTT-TGT--CTGGCACGGATGGCCTAAAAATGGAGCTT---
GCAGCAG--TGAGCGCCGTGATGATTGGTGG-TTGGAC-GGGT-TTCG-GCCCTAGA----
AGCATC----TCATCACGAGGCGTCGGGCA--TGCAT--GCTTGGTTGACTTGACCATT--
TTGATGTCTTTGCGACTAAG-TCGC-T-TTGCATCGTTCAC-CCT
</sequence>
<sequence>
<taxon idref="Armhirtaaa"/>
TCGAAACCTGCAAAAGCAG-AGCCACGTGTG--AACTTGT-TTTA-
AACCATCCC--TTGTTGTGGTGTGGAC-GGCTGC-AT------------TGTGCA-
TCCTC-CATC-CT-CG-ACATGAACAA--CTTTCC-GGCGCGGAATGCGCCAAGGA------
ACTTGAACG-CAAGCTTC--ACC-----TTCTTGT-GCCGACTGGTTGCTTTG-----AG-
G-TG--TT--GCGGTGTTGAA--ATATA-------------TAATCAAATGAC--
TCTCGGCAACGGATATCTCGGCTCTCGCATCGATGAAGAACG--TAGCGAAAT---
GCGATACTTGGTGTGAATTGCA-GAATCCCGTGAACCATCGA---
GTCTTTGAACGCAAGTT-
GCGCCCGAAGCCTTTTGGCCGAGGGCACGTTTGCCTGGGCGTCACGCATTGCGTCGCTCC--
CC-A-CTTACCTTTTGTTGGG-----TTYGA--G-GGGC-
GGAGAGTGGTCTCCCGTGTGCTT------CTTGCACGGATGACTTAAACTTGGAGCTT---
ACGGCAG--TGAGCGCCGTGATGATTGGTGG-TTGGTT-GGGC-TTAR-ATCCCAAA----
AGCATC----TCGTCATGAAGCGTCSTGCC--TTTTT--GTTCGGATTACTTGAGTGGT--
TTGATGTCATTGTTTGTAAAATTCATT-TTGCTTCTTCCAC-CCT
</sequence>
<sequence>
<taxon idref="Armeriamar"/>
TCGAAACCTGCAAAAGCAG-AGCCACGTGTG--AACTTGT-TTTA-
AACCATCCC--TTGTTGTGGTGTGGAC-GGCTGC-AT------------TGTGCA-
TCCTC-CATC-CT-CG-ACATGAACAA--CTTTCC-GGCGCGGAATGCGCCAAGGA------
ACTTGAACG-CAAGCTTC--ACC-----TTCTTGT-GCCGACTGGTTGCTTTG-----AG-
G-TG--TT--GCGGTGTTGAA--ATATA-------------TAATCAAATGAC--
TCTCGGCAACGGATATCTCGGCTCTCGCATCGATGAAGAACG--TAGCGAAAT---
GCGATACTTGGTGTGAATTGCA-GAATCCCGTGAACCATCGA---
GTCTTTGAACGCAAGTT-
GCGCCCGAAGCCTTTTGGCCGAGGGCACGTTTGCCTGGGCGTCACGCATTGCGTTGCTCC--
CC-A-TCCACCTTTTGTTGGG-----TTAGA--G-GGGC-
GGAGAGTGGTCTCCCGTGTGCTT------CTTGCACGGATGACTTAAACTTGGAGCTT---
ACGGCAG--TGAGCGCCGTGATGATTGGTGG-TTGGTT-GGGC-TTAG-ATCCCAGA----
AGCATC----TCGTCATGAAGCGTCGTGCC--TTTTT--GTTCGGATTACTTGAGTGGT--
TTGATGTCATTGTTTGTAAAATTCATT-TTGCTTCTTCCAC-CCT
</sequence>
<sequence>
<taxon idref="Armeriawel"/>
TCGAAACCTGCAAAAGCAG-AGCCACGTGTG--AACTTGT-TTTA-
AACCATCCC--TTGTTGTGGTGTGGAC-GGCTGC-AT------------TGTGCA-
TCCTC-CATC-CT-CG-ACATGAACAA--CTTTCC-GGCGCGGAATGCGCCAAGGA------
ACTTGAACG-CAAGCTTC--ACC-----TTCTTGT-GCCGACTGGTTGCTTTG-----AG-
G-TG--TT--GCGGTGTTGAA--ATATA-------------TAATCAAATGAC--
TCTCGGCAACGGATATCTCGGCTCTCGCATCGATGAAGAACG--TAGCGAAAT---
GCGATACTTGGTGTGAATTGCA-GAATCCCGTGAACCATCGA---
GTCTTTGAACGCAAGTT-
GCGCCCGAAGCCTTTTGGCCGAGGGCACGTTTGCCTGGGCGTCWCGCATTGCGTYGCTCC--
CC-A-YCCACCTTTTGTTGGG-----TTAGA--G-GGGC-
GGAGAGTGGTCTCCCGTGTGCTT------CTTGCACGGATGACTTAAACTTGGAGCTT---
ACGRCAG--TGAGCGCCGTGATGATTGGTGG-TTGGTT-GGGC-TTAG-ATCCCAGA----
AGCATC----TCGTCATGAAGCGTCGTGCC--TTTTT--GTTCGGATTACTTGAGTGGT--
TTGATGTCATTGTTTGTAAAATTCATT-TTGCTTCTTCCAC-CCT

</sequence>
<sequence>
<taxon idref="Armeriamad"/>
TCGAAACCTGCAAAAGCAG-AGCCACGTGTG--AACTTGT-TTTA-
AACCATCCC--TTGTTGTGGTGTGGAC-GGCTGC-AT------------TGTGCA-
TCCTC-CATC-CT-CG-ACATGAACAA--CTTTCC-GGCGCGGAATGCGCCAAGGA------
ACTTGAACG-CAAGCTTC--ACC-----TTCTTGT-GCCGACTGGTTGCTTTG-----AG-
G-TG--TT--GCGGTGTTGAT--ATATA-------------TAATCAAAAGAC--
TCTCGGCAACGGATATCTTGGCTCTCGCATCGATGAAGAACG--TAGCGAAAT---
GCGATACTTGGTGTGAATTGCA-GAATCCCGTGAACCATCGA---
GTCTTTGAACGCAAGTT-
GCGCCCGAAGCCTTTTGGCCGAGGGCACGTTTGCCTGGGCGTCACGCATTGCGTCGCTCC--
CC-A-CCCACCTTTTGTTGGG-----TTAGA--G-GGGC-
GGAGAGTGGTCTCCCGTGTGCTT------CTTGCACGGATGACTTAAACTTGGAGCTT---
ACGGCAG--TGAGCGCCGTGATGATTGGTGG-TTGGTT-GGGC-TTAG-ATCCCAAA----
AGCATC----TCGTCATGAAGCGTCGTGCC--TTTTT--GTTCGGATTACTTGAGTGGT--
TTGATGTCATTGTTTGTAAAATTCATT-TTGCTTCTTCCAC-CCT
</sequence>
<sequence>
<taxon idref="Armeriaspe"/>
TCGAAACCTGCAAAAGCAG-AGCCACGTGTG--AACTTGT-TTTA-
AACCATCCC--TTGTTGTGGTGTGGAC-GGCTGC-AT------------TGTGCA-
TCCTC-CATC-CT-CG-ACATGAACAA--CTTTCC-GGCGCGGAATGCGCCAAGGA------
ACTTGAACG-CAAGCTTC--ACC-----TTCTTGT-GCCGACAGGTTGCTTTG-----AG-
G-TG--TT--GCGGTGTTGAA--ATATA-------------TAATCAAATGAC--
TCTCGGCAACGGATATCTCGGCTCTCGCATCGATGAAGAACG--TAGCGAAAT---
GCGATACTTGGTGTGAATTGCA-GAATCCCGTGAACCATCGA---
GTCTTTGAACGCAAGTT-
GCGCCCGAAGCCTTTTGGCCGAGGGCACGTTTGCCTGGGCGTCACGCATTGCGTCGCTCC--
CC-A-CCCACCTTTTGTTGGG-----TTAGA--G-GGGC-
GGAGAGTGGTCTCCCGTGTGCTT------CTTGCACGGATGACTTAAACTTGGAGCTT---
ACGGCAG--TGAGCGCCGTGATGATTGGTGG-TTGGTT-GGGC-TTAG-ATCCCAAA----
AGCATC----TCGTCATGAAGCGTCGTGCC--TTTTT--GTTCGGATTACTTGAGTGGT--
TTGATGTCATTGTTTGTAAAATTCATT-TTGCTTCTTCCAC-CCT
</sequence>
<sequence>
<taxon idref="Armeriasin"/>
TCGAAACCTGCAAAAGCAG-AGCCACGTGTG--AACTTGT-TTTA-
AACCATCCC--TTGTTGTGGTGTGGAC-GGCTGC-AT------------TGTGCA-
TCCTC-CATC-CT-CG-ACATGAACAA--CTTTCC-GGCGCGGAATGCGCCAAGGA------
ACTTGAACG-CAAGCTTC--ACC-----TTCTTGT-GCCGACTGGTTGCTTTG-----AG-
G-TG--TT--GCGGTGTTGAA--ATATA-------------TAATCAAATGAC--
TCTCGGCAACGGATATCTCGGCTCTCGCATCGATGAAGAACG--TAGCGAAAT---
GCGATACTTGGTGTGAATTGCA-GAATCCCGTGAACCATCGA---
GTCTTTGAACGCAAGTT-
GCGCCCGAAGCCTTTTGGCCGAGGGCACGTTTGCCTGGGCGTCACGCATTGCGTCGCTCC--
CC-A-CTTACCTTTTGTTGGG-----TTAGA--G-GGGC-
GGAGAGTGGTCTCCCGTGTGCTT------CTTGCACGGATGACTTAAACTTGGAGCTT---
ACGGCAG--TGAGCGCCGTGATGATTGGTGG-TTGGTT-GGGC-TTAG-ATCCCAAA----
AGCATC----TCGTCATGAAGCGTCGTGCC--TTTTT--GTTCGGATTACTTGAGTGGT--
TTGATGTCATTGTTTGTAAAATTCATT-TTGCTTCTTCCAC-CCT
</sequence>
<sequence>
<taxon idref="Cephturcoc"/>
TCGAAACCTGC-ATAGCAG-AGCAACATGCG--AACTTGTCTTTT-
CAACCTTCC--TTCCTGTGTGGCGGGTCAGAATA-CT------------CGTGCT-

TCCTC-CGCC-TTGCG-GGATGAACAA---TTTTC-GGCGCGGGTCGCGCCAAGGA------
CCATGAACGTTAGGCCTT--GCC-----TCTTTGC-GCCGTTAGGCTGCGGCG-----AG-
GG-G--CC--CCATCATTGAT--AACTA-C-----------CAATTGAATGAC--
TCTTGACAAAGGATATCTCGGCTCTCGCATCGATGAAGAACG--TAGCGAAAT---
GCGATACTTGGTGTGAATTGCA-GAATCCTGTGAACCATTGA---
GTCTTTGAACGCAAGTT-
GCGCCTGAAGCCTTTTGGCCAAGGGCACGTCTGCCTGGGCGTCACGCATTGCGTCGCCCCAA
CC-A-CCCAACTCGTGTTTGGATATATGTGT--G-GGGC-
GGAGAGTGGTCTCCCGTGTGCCT-CTT-GCTCACATGGATGGCCGAAATAGGGAGCTT---
GTGAAAG--TGAGTGCTGTGATGGTTGGTGG-TTGTTT-GGGC-TCTT-
GCCCTACAAAGCAGCATC----TTGTCACGAAGCGCCA-CCCTGTGTT-G-
GCTCGGAGGAGTCGACCTGG--
TCGATGTTGTCCCAACTTGTGCGCCGCCTTATATCGTCCAC-CTT
</sequence>
<sequence>
<taxon idref="Cephcoe32"/>
TCGAAACCTGC-ATAGCAG-AGCAACATGCG--AACTTGTCTTTT-
CAACCTTCC--TTTCCGTGTGGCGGGTCACAAGA-CT------------CGTGCT-
TCCTC-CGCC-TTGCG-GGATGAACAA---TTTTC-GGCGCGGGTTGCGCCAAGGA------
CCATGAACGTTAGGCCTT--GCC-----TCTTTGC-GCTGTTAGGCTGCGTTG-----AG-
GA-G--CC--CCATCATTGAA--AACTA-C-----------CAATTGAATGAC--
TCTCGACAAAGGATATCTTGGCTCTCGCATCGATGAAGAACG--TAGCGAAAT---
GCGATACTTGGTGTGAATTGCA-GAATCCTGTGAACCATTGA---
GTCTTTGAACGCAAGTT-
GCGCCTGAAGCCTTTTGGCCAAGGGCACGTCTGCCTGGGCGTCACACATCGCGTCGCCCCTA
CC-T-CCCAACTCGTGTTTGG--ATATGTGT--G-GGGC-
GGAGAGTGGTCTCCCGTGTGCCT-CTC-GCTCACATGGATGGCCGAAATAAGGAGCTT---
GTGGTAG--TGAACGCTGTGATGATTGGTGG-TTGGAT-GGGC-TCTT-GCCCCGCAA---
AGCATC----TTGTCACGAAGCGCCG-CCCTGTGTT-G-GCTCGGAGGAGTCGACCTGG--
TCGATGTCGTCTCAACTTGTGTGC--C-TCTTATTGTCCAC-CAT
</sequence>
<sequence>
<taxon idref="Bamianiaaa"/>
TCGAAACCTGT-ATAGCAG-AGCAACATGCG--AACTTGTCTTTT-
CAACCTTCC--TTTCTGTGTGGCGGATCAGAAGG-CT------------CGTGCT-
TCCTC-TGCC-ATGCG-GGATGAACAA---TTTTC-GGCGCGGGTTGCGCCAAGGA------
CCATGAATGTTAGGCCTT--GCC-----TCTTTGC-GCTGTTAGGCTGCGTTG-----AG-
GA-G--CC--CCATCATTGAA--AACTA-C-----------CAATTGAATGAC--
TCTCGACAAAGGATATCTCGGCTCTCGCATCGATGAAGAACG--TAGCGAAAT---
GCGATACTTGGTGTGAATTGCA-GAATCCTGTGAACCATTGA---
GTCTTTGAACGCAAGTT-
GCGCCTGAAGCCTTTTGGCCAAGGGCACGTCTGCCTGGGCGTCACGCATCGCGTCGCCCCTA
CC-T-CCCAACTCGTGTTTGG--ATATGTGT--G-GGGC-
GGAGAGTGGTCTCCCGTGTGCCT-CTT-GCTCACATGGATGGCCGAAATAAGGAGCTT---
GTGGCAGTGTGAGCGCTGTGATGATTGGTGG-TTGGAT-GGGC-TCTT-GCCCTACAG---
AGCATC----TTGTCACGAAGCGCCA-CCCTGTGTT-G-GCTCGGAGGAGTCGACCTGA--
TCGATGTTATCTCGACTTGTGCGC--C-TTTTATCGTCCAC-CAT
</sequence>
<sequence>
<taxon idref="Dicgrif30"/>
TCGAAACCTGC-ATAGCAG-AGCAACATGCA--AACTTGTCTTTT-
CAACCTTCC--TTTCCGTGTGGCGTGTTAGAAGA-CT------------TGTGCT-
TCCTC-CGCC-TTGCG-GTATGAACAT---TTTTC-GGCGCGGGTTGTGCCAAGGA------
CCATGAACGTTAGGCCTC--ACC-----TCTTTGT-GCCGTTAGGCTGCCTTG-----AG-
GA-G--TC--C-ATCATTGAA--AAATA-T-----------CAATTTAATGAC--
TCTTGACAAAGGATATCTTGGCTCTCGCATCGATGAAGAACG--TAGCGAAAT---

GCGATACTTGGTGTGAATTGCA-GAATCCTGTGAACCATTGA---
GTCTTTGAACGCAAGTT-
GCGCCCAAAGCCTTCTGGCTAAGGGCACGTCTGCTTGGGCGTCACGCATTGCATCGCCCCTA
CC-T-CCTAACTCGTGTTTGG--ATACGTGT--G-GGGC-
GCAGAGTGGTCTCCCGTGTGCCT-CTT-GCTCACATGGATGGCCGAAATAAGGAGCTT---
TCGGTGG--TGAGTGCCGTTATGATTGGTGG-TTGGAT-TGGY-TGTT-GCCCTACAA---
AGCATC----TTGTCATGAAGCGCCA-CCCCGTGTT-
G---
TCCAT-CGT
</sequence>
<sequence>
<taxon idref="Dicmacr34"/>
TCGAAACCTGC-ATAGCAG-AGCAACATGCA--AACTTGTCTTTT-
CAACCTTCC--TTTCCGTGTGGCGTGTTAGAAGA-CT-------------TGTGCT-
TCCTC-CGCC-TTGCG-GTATGAACAT---TTTTC-GGCGCGGGTTGTGCCAAGGA------
CCATGAACGTTAGGCCTC--ACC-----TCTTTGT-GCCGTTAGGCTGCCTTG-----AG-
GA-G--TC--C-ATCATTGAA--AAATA-T------------CAATTTAATGAC--
TCTTGACAAAGGATATCTTGGCTCTCGCATCGATGAAGAACG--TAGCGAAAT---
GCGATACTTGGTGTGAATTGCA-GAATCCTGTGAACCATTGA---
GTCTTTGAACGCAAGTT-
GCGCCCGAAGCCTTTTGGCTAAGGGCACGTCTGCTTGGGCGTCACACATCGCATCGCCCCTA
CC-T-CCTAACTCGTGTTTGG--ATATGTGT--G-GGGC-
GCAGAGTGGTCTCCCGTGTGCCT-CTT-GCTCACATGGATGGCCGAAATAAGGAGCTT---
GCGGTGG--TGAGCGCCTTTATGATAGGTCG-TTGGAT-TGGC-TGTT-GCCCTACAA---
AGCATC----TTGTCATGAAGTGCCA-CCCCGTGTT-G-GCTTGGAGGACTTGGCCTGG--
TCGATGTTATCCCGATTTGTGTGT--C-ATTTGTTTTCCAC-TGT
</sequence>
<sequence>
<taxon idref="Bukcabu39"/>
TCGAAACCTGC-ATAGCAG-AGCAACATGCA--AACTTGTCTTCT-
CAACCTTCC--TTTCCGTGTGGCGTGTCAGAAGA-CT-------------TGTGCT-
TCCTT-AGCC-ATGCG-GAATGAACAA---TTTTC-GGCGCGGGTTGCGCCAAGGA------
CCATGAATGCTAGGCCTC--ACC-----TCTTTGC-GCTGTTAGGCTGCCTTG-----AG-
GG-G--TC--CCATCATTGAA--AAATATT------------CAATATAATGAC--
TCTTGACAAAGGATATCTTGGCTCTTGCATCGATGAAGAACG--TAGCGAAAT---
GCGATACTTGGTGTGAATTGCA-GAATCCTGTGAACCATTGA---
GTCTTTGAACGCAAGTT-
GCGCCCGAAGCCTTCTGGCCAAGGGCACGTCTGCTTGGGCGTCACGCATCGCATCGCCCCTA
CA-T-CCTATCTCGTGTTTGG----ATGTGT--G-GGGC-
GTAGAGTGGTCTCCCGTGTGCCT-CTT-GCTCACACGGATGGCCGAAATAAGGAGCTT---
GCGGTGG--TGAGCGCCATGATGATTGGTGG-TTGGAT-TGGC-TGTT-GCCCTACAA---
AGCATC----TTGTCATGAAGCGCCA-CCCCGTGTT-G-GCTTGGAGGACTCAGCCTGG--
TCGATGTTATACCGATTTGTGTGC--C-TTTTATAGTCCAC-CAT
</sequence>
<sequence>
<taxon idref="Vassizso44"/>
TCGAAACCTGC-ATAGCAG-AGCAACATGCG--AACTTGTCTTAT-
CAACCTTCC--TTTCTCCGGGGCGGGTCAGAAGA-CT-------------TGTGCT-
TCCTC-CGCC-TTGTG-GGATGAACAA---TTTTG-GGCGCGGGTTGCGCCAAGGA------
CCATGAATGTTAGGCCTT--GCC-----TCTTTGC-GCCGTTAGGCCGCGTTG-----AG-
GG-G--TC--CCATCATTGAA--AACTA-T------------CAATTGAATGAC--
TCTCAACAAAGGATATCTCGGCTCTCGCATCGATGAAGAACG--TAGCGAAAT---
GCGATACTTGGTGTGAATTGCA-GAATCCTGTGAACCATTGA---
GTCTTTGAACGCAAGTT-
GCGCCCCAAGCCTTTTGGCCAAGGGCACGTCTGCCTGGGTGTCACGCATCGCGTCGCCCCTA
CC-T-CCCAACCTGTGTTTGG--CTATGTGT--G-GGGC-

GGAGAGTGGTCTCCCGTGTGCCT-CTT-GCTCACATGGATGGCCGAAATAAGGAGCTT---
GTGGCAG--TGAGCGCTGTGATGATTGGTGG-TTGGAT-GGAC-TCTT-GCCCTACAA---
AGCATC----TTGTCATGAAGCGCCA-CCCTGTGTT-G-GCTCGGAGGACTCGACATGG--
TTGATGTTATTCTGATTTGTGTGC--C-ATTTATTGTCCAC-CAT
</sequence>
<sequence>
<taxon idref="ChaetolSet"/>
TCGAAACCTGC-GTAGCAG-AGCAACATGCG--AACTTGTCTTAT-
CAAACCTCC--TTTCCCTGGGGCGGGTCAGAAGA-CT-------------TGTGCT-
TCCTC-CGCC-TTGTG-GGATGAACAA---TTTTC-GGCGCGGGTTGCGCCAAGGA------
CCATGAATGTTAGGCCTT--GCC-----TCTTTGC-GCCGTTAGGCCGCGTTG-----AG-
GA-G--TC--CCATCATTGAA--AACTA-T------------CAATTGAATGAC--
TCTCAACAAAGGATATCTCGGCTCTTGCATCGATGAAGAACG--TAGCGAAAT---
GCGATACTTGGTGTGAATTGCA-GAATCCTGTGAACCATTGA---
GTCTTTGAACGCAAGTT-
GCGCCCCAAGCCTTTTGGCCAAGGGCACGTCTGCCTGGGTGTCACGCATCGCGTCGCCCCTA
CC-T-
CCCAACCTGTGTTTGG---

</sequence>
<sequence>
<taxon idref="Glaspeci25"/>
TCGAAACCTGC-ATAGCAG-AGCAACATGCG--AACTTGTCTTTT-
CAACCTTCCTTTTTCCGTGTGGTGGGTTAGACGA-CT-------------CGTGCT-
TCCTC-CGCC-TTGCG-GGATGAACAA---CTTTC-GGCGCGGGTTGCGCCAAGGA------
CCATGAACGTTAGGCCTC--GCC-----TCTTTGC-GCCGTTAGGCTGCGTTG-----AG-
GA-G--TC--CCATCATTGAA--AAATA-T------------CAATTTAATGAC--
TCTTGACAAAGGATATCTCGGCTCTCGCATCGATGAAGAACG--TAGCGAAAT---
GCGATACTTGGTGTGAATTGCA-GAATCCTGTGAACCATTGA---
TTCTTTGAACGCAAGTT-
GCGCCCGAAGCCTTTTGGCCAAGGGCACGTCTGCCTGGGCGTCACGCATAGCGTCGCCCCTA
CC-A-CCCAACTCGTGTTTGG--ATGTGTAT--G-GGGT-
GGAGAGTGGTCTCCCGTGTGCC---TT-GCTCACATGGATGGCCGAAATAAGGAGCTT---
GCGGCGG--TCAGCGCTGTGATGATTGGTGG-TTGGAT-GGGC-TCTT-GCCCTACAA---
AGCATC----TTGTCATGAAGTGCCA-CCCCGTGTT-G-GCTCGGAGGACTCGGTCTGG--
TCGATGTTATCCAGATTTGTGCGC--C-TTTTATTGTACAC-CAT
</sequence>
<sequence>
<taxon idref="Alavaeaaaa"/>
TCGAAACCTGC-ATAGCAG-AGCAACATGCG--AACTTGTCTTTT-
CAACCTTCCTTTTTCCGTGTGGCGGGTCAGACGA-CT-------------CGTGCT-
TCCTC-CGCC-TTGCG-GGATGAACAA---CTTTC-GGCGCGGGTTGCGCCAAGGA------
CCATGAACGTTAGGCCTC--GCC-----TCTTTGC-GCCGTTAGGCTGCGTTG-----AG-
GA-G--TC--CCATCATTGAA--AAATA-T------------CAATTTAATGAC--
TCTTGACAAAGGATATCTCGGCTCTCGCATCGATGAAGAACG--TAGCGAAAT---
GCGATACTTGGTGTGAATTGCA-GAATCCTGTGAACCATTGA---
ATCTTTGAACGCAAGTT-
GCGCCCGAAGCCTTTTGGCCAAGGGCACGTCTGCCTGGGCGTCACGCATAGCGTCGCCCCTA
CC-T-CCCAACTCGTGTTTGG--ATGTGTAT--G-GGGT-
GGAGAGTGGTCTCCCGTGTGC---CTT-GCTCACATGGATGGCCGAAATAAGGAGCTT---
GCGGCGG--TGAGCGCTGTGATGATTGGTGG-TTGGAT-GGGC-TCTT-GCCCTACAA---
AGCATC----TTGTCATGAAGTGCCA-CCCCGTGTT-G-GCTTGGAGGACTCGGTCTGG--
TCGATGTTATCCAGATTTGTGCGC--C-TTTTATTGTCCAC-CAT

</sequence>
<sequence>
<taxon idref="Bodeanum1m"/>
TCGAAACCTGC-ATAGCAG-AGCAACATGCG--AACTTGTCTTTT-
CAACCTTCC--TTTCCGTCTGGCGGGTCAGACGA-CT-------------CGTGCT-
TCCTC-CGCC-TTACG-GGATGAACAA---CTTTC-GGCGCGGGTTGCGCCAAGGA------
CCATGAACGTTAGGCCTC--GCC-----TCTTTGC-GTTGTTASGCTGCGTTG-----AG-
GA-G--TC--CCATCATTGAA--AAATA-T------------CAATTTAATGAC--
TCTTGACAAAGGATATCTCGGCTCTCGCATCGATGAAGAACG--TAGCGAAAT---
GCGATACTTGGTGTGAATTGCA-GAATCCTGTGAACCATTGA---
GTCTTTGAACGCAAGTT-
GCGCCCGATGCCTTTTGGCCAAGGGCACGTCTGCCTGGGCGTCACGCATAGCGTCGCCCCTA
CC-T-CCCAACTCGTGTTTGG--ATATGTGT--G-GGGC-
GGAGAGTGGTCTCCCGTGTGC---CTT-GCTCACATGGATGGCCGAAATAAGGAGCTT---
GCGGCGG--TGAGCGCCGTGATGATTGGTGG-TTGGAT-GGGC-TCTT-GCCCTACAA---
AGCATC----TTGTCATGAAGTGCCA-CCCTGTGTT-G-GCTCGGAGGACTCGGTCTGG--
TCGATGTTATCCGGATTTGTGCGC--C-TTTTATTGTCCAC-CAT
</sequence>
<sequence>
<taxon idref="scorpiu927"/>
TCGAAACCTGC-ATAGCAG-AGCAACATGCG--AACTTGTCTTTT-
CAACCTTCC--TTTCCGTGTGGCGGGTCGGACGA-CT-------------CGTGCT-
TCCTC-CGCC-TTACG-GGATGAACAA---CTTTC-GGCGCGGGTTGCGCCAAGGA------
CCATGAACGTTAGGCCTC--GCC-----TCTTTGC-GCCGTTAGGCTGCGTTG-----AG-
GA-G--TC--ACATCATTGAA--AAATA-T------------CAATTTAATGAC--
TCTTGACAAAGGATATCTCGGCTCTCGCATCGATGAAGAACG--TAGCGAAAT---
GCGATACTTGGTGTGAATTGCA-GAATCCTGTGAACCATTGA---
GTCTTTGAACGCAAGTT-
GCGCCCGAAGCCTTTTGGCCAAGGGCACGTCTGCCTGGGTGTCACGCATAGCGTCGCCCCTA
CC-T-CCCAACTCGTGTTTGG--ATATGTGT--G-GGGC-
GGAGAGTGGTCTCCCGTGTGC---CTT-GCCCACATGGATGGCCGAAATAAGGAGCTT---
GCGGCGG--TGAGCGCCGTGATGATTGGTGG-TTGGATGGGGC-TCTT-GCCCTACAA---
AGCATC----TTGTCATGAAGTGCCACCCCCGTGTT-G-GCTCGGAGGACTCGGTCTGG--
TCGATGTTATCCAGATTTGTGCGC--C-TTCTATTGTCCAC-CAT
</sequence>
<sequence>
<taxon idref="Aspadanumm"/>
TCGAAACCTGC-ATAGCAG-AGCAACATGCG--AACTTGTCTTTT-
CAACCTTCC--TTTCCGTGTGGCGGGTTAGAAGA-CT-------------CGTGCT-
TCCTC-CGCC-TTGCG-GGATGAACAA---TTTTC-GGCGCGGGTTGCGCCAAGGA------
CCATGAACGTTAGGCCTT--GCC-----TCTTTGC-GCAGTTAGGATGCGTTG-----AG-
GA-G--CC--CCATCATTGAA--AACTA-T------------CTATTGAATGAC--
TCTCGACAAAGGATATCTCGGCTCTCGCATCGATGAAGAACG--TAGCGAAAT---
GCGATACTTGGTGTGAATTGCA-GAATCCTGTGAACCATTGA---
TTCTTTGAACGCAAGTT-
GCGCCTGAAGCCTTTTGGCCAAGGGCACGTCTGCCTGGGCGTCACGCATCGCGTCGCCCCTA
CC-T-CCCAACTCGTGTTTGG--ATATGTGT--G-GGGC-
GGAGAGTGGCCTCCCGTGTGCCT-CTT-GCTCACACGGATGGCCGAAATAAGGAGCTT---
GCGGCAG--CGAGCGCTGTGATGATTGGTGG-TTGGAT-GGGC-TCTT-GCCTTACAA---
AGCATC----TTGTCACGAAGCGCCG-CCCCGTGTT-G-GCTTGCAGGACTCGACCTGG--
TCGATGTTATCCCGATTTGTGTGC--C-TTTTATCGTCCAC-CAT
</sequence>
<sequence>
<taxon idref="Bromifoliu"/>
TCGAAACCTGC-ATAGCAG-AGCAACATGCG--AACTTGTCTTTT-
CAACCTTCC--TTTCCGTGTGGCGGGTCAGAAGA-CT-------------CGTGCT-

TCCTC-CGCC-TTGCG-GGATGAACAA---TTTTC-GGCGCGGGTTGCGCCAAGGA------
CCATGAACGTTAGGCCTT--GCC-----TCTTTGC-GCCGTTAGGCTGCGTTG-----AG-
GA-G--CC--CCATCATTGAA--AACTA-T-----------CTATTGAATGAC--
TCTCGACAAAGGATATCTCGGCTCTCGCATCGATGAAGAACG--TAGCGAAAT---
GCGATACTTGGTGTGAATTGCA-GAATCCTGTGAACCATTGA---
TTCTTTGAACGCAAGTT-
GCGCCTGAAGCCTTTTGGCCAAGGGCACGTCTGCCTGGGCGTCACGCATCGCGTCGCCCCTA
CC-T-CCCAACTCGTGTTTGG--ATATGTGT--G-GGGC-
GGAGAGTGGCCTCCCGTGTGCCT-CTT-GCTCACATGGATGGCCGAAATAAGGAGCTT---
GTGGCAG--CGAGCGCTGTGATGATTGGTGG-TTGGAT-GGGC-TTTT-GCCTTACAA---
AGCATC----TTGTCACGAAGCGCCG-CCCCGTGTT-G-GCTTGGAGGACTCGACCTGG--
TCGATGTTATCACGATTTGTGTGC--C-TTTTATCGTCCAC-CAT
</sequence>
<sequence>
<taxon idref="Bracteatum"/>
TCGAAACCTGC-ATAGCAG-AGCAACATGCG--AACTTGTCTTCT-
CAACCTTCC--TTTCCGTGTGGCGGGTCAGAAGA-CT------------CGTGCT-
TCCTC-CGCC-TTGCG-GGATGAACAA---TTTTC-GGCGCGGGTTGCGCCAAGGA------
CCATGAACGTTAGGCCTT--GCC-----TCTTTGC-GCCGTTAGGCTGCGTTG-----AG-
GA-G--CC--CCATCATTGAA--AACTA-T-----------CTATTGAATGAC--
TCTCGACAAAGGATATCTCGGCTCTCGCATCGATGAAGAACG--TAGCGAAAT---
GCGATACTTGGTGTGAATTGCA-GAATCCTGTGAACCATTGA---
TTCTTTGAACGCAAGTT-
GCGCCTGAAGCCTTTTGGCCAAGGGCACGTCTGCCTGGGCGTCACGCATCGCGTCGCCCCTA
CC-T-CCCAACTCGTGTTTGG--ATATGTGT--G-GGGC-
GGAGAGTGGCCTCCCGTGTGCCT-CTT-GCTCACATGGATGGCCGAAATAAGGAGCTT---
GTGGCAG--CGAGCGCTGTGATGATTGGTGG-TTGGAT-GGGC-TTTTTGCCTTACAA---
AGCATC----TGGTCACGAAGCGCCGCCCCGGTGTT-G-GCTTGGAGGACTCGACCTGG--
TCGATGTTGTCCCGATTTGTGTGC--CTTTTTATCGTCCAC-CAT
</sequence>
<sequence>
<taxon idref="Flexuosumm"/>
TCGAAACCTGC-ATAGCAG-AGCAACATGCG--AACTTGTCTTTT-
CAACCTTCC--TTTCCGTGTGGCGGGTTAGAAGA-CT------------CGTGCT-
TCCTC-CGCC-TTGCG-GGATGAACAA---TTTTC-GGCGCGGGTTGCGCCAAGGA------
CCATGAACGTTAGGCCTT--GCC-----TCTTTGC-GCAGTTAGGATGCGTTG-----AG-
GA-G--CC--CCATCATTGAA--AACTA-T-----------CTATTGAATGAC--
TCTCGACAAAGGATATCTCGGCTCTCGCATCGATGAAGAACG--TAGCGAAAT---
GCGATACTTGGTGTGAATTGCA-GAATCCTGTGAACCATTGA---
TTCTTTGAACGCAAGTT-
GCGCCTGAAGCCTTTTGGCCAAGGGCACGTCTGCCTGGGCGTCACGCATCGCGTCGCCCCTA
CC-T-CCCAACTCGTGTTTGG--ATATGTGT--G-GGGC-
GGAGAGTGGCCTCCCGTGTGCCT-CTT-GCTCACACGGATGGCCGAAATAAGGAGCTT---
GCGGCAG--CGAGCGCTGTGATGATTGGTGG-TTGGAT-GGGC-TCTT-GCCTTACAA---
AGCATC----TTGTCACGAAGCGCCG-CCCCGTGTT-G-GCTTGCAGGACTCGACCTGG--
TCGATGTTATCCCGATTTGTGTGC--C-TTTTATCGTCCAC-CAT
</sequence>
<sequence>
<taxon idref="Pterostegi"/>
TCGAAACCTGC-ATAGCAG-AGCAACATGCG--AACTTGTCAATT-
CAACCCTCC--TTTCCGTGTGGCGGGTTAGACGA-CC------------CGTGCT-
TCCTC-CGCC-TTGCG-GGATGAAAAA---CTTTT-GGCGCGGGTTGCGTCAAGGA------
CCATGAACGTTAGGCCTT--GCC-----TCTTTGC-GCCGTTAGGCTGCGTTG-----AG-
GA-G--TC--CTATCATTGAA--AAATA-T-----------CAATTTAATGAC--
TCTTGACAAAGGATATCTCGGCTCTCGCATCGATGAAGAACG--TAGCGAAAT---
GCGATACTTGGTGTGAATTGCA-GAATCCTGTGAACCATTGA---

GTCTTTGAACGCAAGTT-
GCGCCCGAAGCCTTTTGGCCAAGGGCACGTCTGCCTGGGCGTCACGCATAGCGTCGCCCCTA
CC-T-CCCAACTCGTGTTTGG--ATATGTGT--G-GGGC-
GGAGAGTGGTCTCCCGTGTGC---CTT-GCTCACATGGATGGCCGAAATAAGGAGCTT---
GCGGCGG--CGAGCGCCGTGATGATTGGTGG-TTGGAT-GGGC-TCTT-GCCCTACAA---
AGCATC----TTGTCACGAAGTGCCA-CCCCGTGTT-G-GCTTGGAGGACTCGGTCTGG--
TCGATGTTATCCAGATTTGTGCGC--G-TTTTATTGTCCAC-CAT
</sequence>
<sequence>
<taxon idref="Cymosummmm"/>
TCGAAACCTGC-ATAGCAG-ATCAACATGCG--AACTTGTCAATT-
CAACCCTCC--TTTCCGTGTGGCGGGTTAGACGA-CT-------------CGTGCT-
TCCTC-CGCC-TTGCG-GGATGAAAAA---CTTTC-GGCGCGGGTTGCGTCAAGGA------
CGATGAACGTTAGGCCTC--GCC-----TCTTTGC-GCCGTTAGGCTGCGTTG-----AG-
GA-G--TC--CTATCATTGAA--AAATA-T------------CAATTTAATGAC--
TCTTGACAAAGGATATCTCGGCTCTCGCATCGATGAAGAACG--TAGCGAAAT---
GCGATACTTGGTGTGAATTGCA-GAATCCTGTGAACCATTGA---
GTCTTTGAACGCAAGTT-
GCGCCCGAAGCCTTTTGGCCAAGGGCACGTCTGCCTGGGCGTCACGCATAGCGTCGCCCCTA
CC-T-CCCAACTCGTGTTTGG--ATATGTGT--G-GGGC-
GGAGAGTGGTCTCCCGTGTGC---CTT-GCTCACATGGATGGCCGAAATAAGGAGC-T---
ATGGCGG--TGAGTGCCGTGATGATTGGTGG-TTGGAT-GGGC-TCTT-GCCCTACAA---
AGCATC----TTGTCACGAAGTGCCA-CCCCGTGTT-G-GCTCGGAGGACTCGGTCTGG--
TCGATGTCATCCAGATTTGTGCGC--C-TTTTATTGTCCAC-CAT
</sequence>
<sequence>
<taxon idref="Gilliatiii"/>
TCGAAACCTGC-ATAGCAG-AGCAACATGCG--AACTTGTCTTTT-
CAACCTTCC--TTTCCGTGTGGCGGGTTAGAAGA-CT-------------CGTGCT-
TCCTC-CGCC-TTGCG-GGATGAACAA---TTTTC-GGCGCGGGTTGCGCCAAGGA------
CCATGAACGTTAGGCCTT--GCC-----TCTTTGC-GCAGTTAGGATGCGTTG-----AG-
GA-G--CC--CCATCATTGAA--AACTA-T------------CTATTGAATGAC--
TCTCGACAAAGGATATCTCGGCTCTCGCATCGATGAAGAACG--TAGCGAAAT---
GCGATACTTGGTGTGAATTGCA-GAATCCTGTGAACCATTGA---
TTCTTTGAACGCAAGTT-
GCGCCTGAAGCCTTTTGGCCAAGGGCACGTCTGCCTGGGCGTCACGCATCGCGTCGCCCCTA
CC-T-CCCAACTCGTGTTTGG--ATATGTGT--G-
GGGCGGGAGAGTGGCCTCCCGTGTGCCT-CTT-
GCTCACACGGATGGCCGAAATAAGGAGCTT---GCGGCAG--
CGAGCGCTGTGATGATTGGTGG-TTGGAT-GGGC-TCTT-GCCTTACAA---AGCATC----
TTGTCACGAAGCGCCG-CCCCGTGTT-G-GCTTGCAGGACTCGACCTGG--
TCGATGTTATCCCGATTTGTGTGC--C-TTTTATCGTCCAC-CAT
</sequence>
<sequence>
<taxon idref="Qunique1ob"/>
TCGAAACCTGC-ATAGCAG-AGCAACATGCG--AACTTGTCTTTT-
CAACCTTCC--TTTCCGTGTGGCGGGTCGGACGA-CT-------------CGTGCT-
TCCTC-CGCC-TTGCG-GGATGAACAA---CTTTC-GGCGCGGGTTGCGCCAAGGA------
CCATGAACGTTATGCCTC--GCC-----TCTTTGC-GCCGTTAGGCTGCGTTG-----AG-
GA-G--TC--ACATCATTGAA--AAATA-T------------CAATTTAATGAC--
TCTTGACAAAGGATATCTCGGCTCTCGCATCGATGAAGAACG--TAGCGAAAT---
GCGATACTTGGTGTGAATTGCA-GAATCCTGTGAACCATTGA---
GTCTTTGAACGCAAGTT-
GCGCCCGAAGCCTTTTGGCCAAGGGCACGTCTGCCTGGGTGTCACGCATAGCGTCGCCCCTA
CC-T-CCCAACTCGTGTTTGG--ATATGTGT--G-GGGC-
GGAGAGTGGTCTCCCGTGTGC---CTT-GCCCACATGGATGGCCGAAATAAGGAGCTT---

GCGGCGG--TGAGCGCCGTGATGATTGGTGG-TTGGATGGGGC-TCTT-GCCCTGCAA---
AGCATC----TTGTCATGAAGTGCCACCCCCGTGTT-G-GCTCGGAGGACTCGGTCTGG--
TCGATGTTATCCAGATTTGTGCGC--A-TTTTATTGTCCAC-CAT
</sequence>
<sequence>
<taxon idref="Scirpinumm"/>
TCGAAACCTGC-ATAGCAG-AGCAACATGCG--AACTTGTCTTTT-
CAACCTTCC--TTTCCGTCTGGCGGGTCAGACGA-CT------------CGTGCT-
TCCTC-CGCC-TTACG-GGATGAACAA---CTTTC-GGCGCGGGTTGCGCCAAGGA------
CCATGAACGTTAGGCCTC--GCC-----TCTTTGC-GTTGTTAGGCTGCGTTG-----AG-
GA-G--TC--CCATCATTGAA--AAATA-T-----------CAATTTAATGAC--
TCTTGACAAAGGATATCTCGGCTCTCGCATCGATGAAGAACG--TAGCGAAAT---
GCGATACTTGGTGTGAATTGCA-GAATCCTGTGAACCATTGA---
GTCTTTGAACGCAAGTT-
GCGCCCGATGCCTTTTGGCCAAGGGCACGTCTGCCTGGGCGTCACGCATAGCGTCGCCCCTA
CC-T-CCCAACTCGTGTTTGG--ATATGTGT--G-GGGC-
GGAGAGTGGTCTCCCGTGTGC---CTT-GCTCACATGGATGGCCGAAATAAGGAGCTT---
GCGGCGG--TGAGCGCCGTGATGATTGGTGG-TTGGAT-GGGC-TCTT-GCCCTACAA---
AGCATC----TTGTCATGAAGTGCCA-CCCTGTGTT-G-GCTCGGAGGACTCGGTCTGG--
TCGATGTTATCCGGATTTGTGCGC--C-TTTTATTGTCCAC-CAT
</sequence>
<sequence>
<taxon idref="Zaefii1iii"/>
TCGAAACCTGC-ATAGCAG-AGCAACATGCG--AACTTGTCTTTT-
CAACCTTCC--TTTCCGTGTGGCGGGTCAGAAGA-CT------------CGTGCT-
TCCTC-CGCC-TTGCG-GGATGAACAA---TTTTC-GGCGCGGGTTGCGCCAAGGA------
CCATGAACGTTAGGCCTT--GCC-----TCTTTGC-GCCGTTAGGATGCGTTG-----AG-
GA-G--CC--CCATCATTGAA--AACTG-T-----------CTATTGAATGAC--
TCTCGACAAAGGATATCTCGGCTCTCGCATCGATGAAGAACG--TAGCGAAAT---
GCGATACTTGGTGTGAATTGCA-GAATCCTGTGAACCATTGA---
TTCTTTGAACGCAAGTT-
GCGCCTGAAGCCTTTTGGCCAAGGGCACGTCTGCCTGGGCGTCACGCATCGCGTCGCCCCTA
CC-T-CCCAACTCGTGTTTGG--ATATGTGT--G-GGGC-
GGAGAGTGGCCTCCCGTGTGCCT-CTT-GCTCACATGGATGGCCGAAATAAGGAGCTT---
GTGGCAG--CGAGCGCTGTGATGATTGGTGG-TTGGAT-GGGC-TCTT-GCCTTACAA---
AGCATC----TTGTCACGAAGCGCCG-CCCCGTGTT-G-GCTTGGAGGACTCGACCTGG--
TCGATGTTATCCCGATTTGTGTGC--C-TTTTATCGTCCAC-CAT
</sequence>
<sequence>
<taxon idref="Kermanense"/>
TCGAAACCTGC-ATAGCAG-AGCAACATGCG--AACTTGTCTTTT-
CAACCTTCCTTTTTCCGTGTGGCGGGTCAGACGA-CT------------CGTGCT-
TCCTC-CGCC-TTGCG-GGATGAACAA---CTTTC-GGCGCGGGTTGCGCCAAGGA------
CCATGAACGTTAGGCCTC--GCC-----TCTTTGC-GCCGTTAGGCTGCGTTG-----GG-
GA-G--TC--CCATCATTGAA--AAATA-T-----------CAATTTAATGAC--
TCTTGACAAAGGATATCTCGGCTCTCGCATCGATGAAGAACG--TAGCGAAAT---
GCGATACTTGGTGTGAATTGCA-GAATCCTGTGAACCATTGA---
GTCTTTGAACGCAAGTT-
GCGCCCGAAGCCTTTTGGCCAAGGGCACGTCTGCCTGGGCGTCACGCATAGCGTCGCCCCTA
CC-T-CCCAACTCGTGTTTGG--ATGTGTGT--G-GGGC-
GGAGAGTGGTCTCCCGTGTGC---CTT-GCTCACATGGATGGCAGAAATAAGGAGCTT---
GCGGCGG--TGAGCGCCGTGATGATTGGTGG-TTGGAT-GGGC-TCTT-GCCCTACAA---
AGCATC----TTGTCATGAAGTGCCA-CCCCGTGTT-G-GCTCGGAGGACTCTGTCTGG--
TCGATGTTATCCAGATTTGTGCGC--C-TTTTATTGTCCAC-CAT
</sequence>
<sequence>

<taxon idref="Chlorosteg"/>
TCGAAACCTGC-ATAGCAG-AGCAACATGCG--AACTTGTCTTTT-
CAACCTTCCTTTTTCCGTGTGGCGGGTCAGACGA-CT-------------CGTGCT-
TCCTC-CGCC-TTGCG-GGATGAACAA---CTTTC-GGCGCGGGTTGCGCCAAGGA------
CCATGAACGTTAGGCCTC--GCC-----TCTTTGC-GCCGTTAGGCTGCGTTG-----GG-
GA-G--TC--CCATCATTGAA--AAATA-T------------CAATTTAATGAC--
TCTTGACAAAGGATATCTCGGCTCTCGCATCGATGAAGAACG--TAGCGAAAT---
GCGATACTTGGTGTGAATTGCA-GAATCCTGTGAACCATTGA---
GTCTTTGAACGCAAGTT-
GCGCCCGAAGCCTTTTGGCCAAGGGCACGTCTGCCTGGGCGTCACGCATAGCGTCGCCCCTA
CC-T-CCCAACTCGTGTTTGG--ATGTGTGT--G-GGGC-
GGAGAGTGGTCTCCCGTGTGC---CTT-GCTCACATGGATGGCAGAAATAAGGAGCTT---
GCGGCGG--TGAGCGCCGTGATGATTGGTGG-TTGGAT-GGGC-TCTT-GCCCTACAA---
AGCATC----TTGTCATGAAGTGCCA-CCCCGTGTT-G-GCTCGGAGGACTCTGTCTGG--
TCGATGTTATCCAGATTTGTGCGC--C-TTTTATTGTCCAC-CAT
</sequence>
<sequence>
<taxon idref="Albocalyci"/>
TCGAAACCTGC-ATAGCAG-AGCAACATGCG--AACTTGTCTTTT-
CAACCTTCCTTTTTCCGTGTGGCGGGTCAGACGA-CT-------------CGTGCT-
TCCTC-CGCC-TTGCG-GGATGAACAA---CTTTC-GGCGCGGGTTGCGCCAAGGA------
CCATGAACGTTAGGCCTC--GCC-----TCTTTGC-GCCGTTAGGCTGCGTTG-----GG-
GA-G--TC--CCATCATTGAA--AAATA-T------------CAATTTAATGAC--
TCTTGACAAAGGATATCTCGGCTCTCGCATCGATGAAGAACG--TAGCGAAAT---
GCGATACTTGGTGTGAATTGCA-GAATCCTGTGAACCATTGA---
GTCTTTGAACGCAAGTT-
GCGCCCGAAGCCTTTTGGCCAAGGGCACGTCTGCCTGGGCGTCACGCATAGCGTCGCCCCTA
CC-T-CCCAACTCGTGTTTGG--ATGTGTGT--G-GGGC-
GGAGAGTGGTCTCCCGTGTGC---CTT-GCTCACATGGATGGCAGAAATAAGGAGCTT---
GCGGCGG--TGAGCGCCGTGATGATTGGTGG-TTGGAT-GGGC-TCTT-GCCCTACAA---
AGCATC----TTGTCATGAAGTGCCA-CCCCGTGTT-G-GCTCGGAGGACTCTGTCTGG--
TCGATGTTATCCAGATTTGTGCGC--C-TTTTATTGTCCAC-CAT
</sequence>
<sequence>
<taxon idref="Avenaceumm"/>
TCGAAACCTGC-ATAGCAG-AGCAACATGCG--AACTTGTCTTTT-
CAACCTTCC--TTTCCGTCTGGCGGGTCAGACGA-CT-------------CGTGCT-
TCCTC-CGCC-TTACG-GGATGAACAA--TCTTTC-GGCGCGGGTTGCGCCAAGGA------
CCATGAACGTTAGGCCTC--GCC-----TCTTTGC-GTTGTTAGGCTGCGTTG-----AG-
GA-G--TC--CCATCATTGAA--AAATA-T------------CAATTTAATGAC--
TCTTGACAAAGGATATCTCGGCTCTCGCATCGATGAAGAACG--TAGCGAAAT---
GCGATACTTGGTGTGAATTGCA-GAATCCTGTGAACCATTGA---
GTCTTTGAACGCAAGTT-
GCGCCCGATGCCTTTTGGCCAAGGGCACGTCTGCCTGGGCGTCACGCATAGCGTCGCCCCTA
CC-T-CCCAACTCGTGTTTGG--ATATGTGT--G-GGGC-
GGAGAGTGGTCTCCCGTGTGC---CTT-GCTCACATGGATGGCCGAAATAAGGAGCTT---
GCGGCGG--TGAGCGCCGTGATGATTGGTGG-TTGGAT-GGGC-TCTT-GCCCTACAA---
AGCATC----TTGTCATGAAGTGCCA-CCCTGTGTT-G-GCTCGGAGGACTCGGTCTGG--
TCGATGTTGTCCGGATTTGTGCGC--C-TTTTATTGTCCAC-CAT
</sequence>
<sequence>
<taxon idref="Modestum1a"/>
TCGAAACCTGC-ATAGCAG-AGCAACATGCG--AACTTGTCTTTT-
CAACCTTCCTTTTTCCGTGTGGCGGGTCAGAAGA-CT-------------CGTGCT-
TTCTC-CGCC-TTGCG-GGATGAACAA---CTTTC-GGCGCGGGTTGCGCCAAGGA------
CCATGAACGTTAGGCCTC--GCC-----TCTTTGC-GCCGTTAGGCTGCGTTG-----AG-

GA-G--TC--CCATCATTGAA--AAATA-T-----------CAATTTAATGAC--
TCTTGACAAAGGATATCTCGGCTCTCGCATCGATGAAGAACG--TAGCGAAAT---
GCGATACTTGGTGTGAATTGCA-GAATCCTGTGAACCATTGA---
GTCTTTGAACGCAAGTT-
GCGCCCGAAGCCTTTTGGCCAAGGGCACGTCTGCCTGGGCGTCACGCATAGCGTCGCCCCTA
CC-T-CCCAACTCGTGTTTGG--ATGTGTGT--G-GGGC-
GGAGAGTGGTCTCCCGTGTGC---CTT-GCTCACATGGCTGGCAGAAATAAGGAGCTT---
GCGGCGG--TGAGCGCCGTGATGATTGGTGG-TTGGAT-GGGC-TCTT-GCCCTACAA---
AGCATC----TTGTCATGAAGTGCCA-CCCCATGTT-G-GCTCGGAGGACTCGGTCTGG--
TCGATGTTATCCAGATTTGTGCGC--C-TTTTATTGTCCAC-CAT
</sequence>
<sequence>
<taxon idref="Oliganthum"/>
TCGAAACCTGC-ATAGCAG-AGCAACATGCG--AACTTGTCTTTT-
CAACCTTCC--TTTCCGTGTGGCGGGTTAGAAGA-CT-------------CGTGCT-
TCCTC-CGCC-TTGCG-GGATGAACAA---TTTTC-GGCGCGGGTTGCGCCAAGGA------
CCATGAACGTTAGGCCTT--GCC-----TCTTTGC-GCAGTTAGGATGCGTTG-----AG-
GA-G--CC--CCATCATTGAA--AACTA-T-----------CTATTGAATGAC--
TCTCGACAAAGGATATCTCGGCTCTCGCATCGATGAAGAACG--TAGCGAAAT---
GCGATACTTGGTGTGAATTGCA-GAATCCTGTGAACCATTGA---
TTCTTTGAACGCAAGTT-
GCGCCTGAAGCCTTTTGGCCAAGGGCACGTCTGCCTGGGCGTCACGCATCGCGTCGCCCCTA
CC-T-CCCAACTCGTGTTTGG--ATATGTGT--G-GGGC-
GGAGAGTGGCCTCCCGTGTGCCT-CTT-GCTCACATGGATGGCCGAAATAAGGAGCTT---
GTGGCAG--CGAGCGCTGTGATGATTGGTGG-TTGGAT-GGGC-TCTT-GCCTTACAA---
AGCATC----TTGTCACGAAGCGCCA-CCCCGTGTT-G-GCTTGCAGGACTCGACCTGG--
TCGATGTTATCCCGATTTGTGTGC--C-TTTTATCGTCCAC-CAT
</sequence>
<sequence>
<taxon idref="Blandummmm"/>
TCGAAACCTGC-ATAGCAG-AGCAACATGCG--AACTTGTCTTTT-
CAACCTTCC--TTTCCGTCTGGCGGGTCAGACGA-CT-------------CGTGCT-
TCCTC-CGCC-TTACG-GGATGAACAA---CTTTC-GGCGCGGGTTGCGCCAAGGA------
CCATGAACGTTAGGCCTC--GCC-----TCTTTGC-GTTGTTAGGCTGCGTTG-----AG-
GA-G--TC--CCATCATTGAA--AAATA-T-----------CAATTTAATGAC--
TCTTGACAAAGGATATCTCGGCTCTCGCATCGATGAAGAACG--TAGCGAAAT---
GCGATACTTGGTGTGAATTGCA-GAATCCTGTGAACCATTGA---
GTCTTTGAACGCAAGTT-
GCGCCCGATGCCTTTTGGCCAAGGGCACGTCTGCCTGGGCGTCACGCATAGCGTCGCCCCTA
CC-T-CCCAACTCGTGTTTGG--ATATGTGT--G-GGGC-
GGAGAGTGGTCTCCCGTGTGC---CTT-GCTCACATGGATGGCCGAAATAAGGAGCTT---
GCGGCGG--TGAGCGCCGTGATGATTGGTGG-TTGGAT-GGGC-TCTT-GCCCTACAA---
AGCATC----TTGTCATGAAGTGCCA-CCCTGTGTT-G-GCTCGGAGGACTCGGTCTGG--
TCGATGTTATCCGGATTTGTGCGC--C-TTTTATTGTCCAC-CAT
</sequence>
<sequence>
<taxon idref="Raddeanumm"/>
TCGAAACCTGC-ATAGCAG-AGCAACATGCG--AACTTGTCTTTT-
CAACCTTCC--TTTCCGTCTGGCGGGTCAGACGA-CT-------------CGTGCT-
TCCTC-CGCC-TTACG-GGATGAACAA---CTTTC-GGCGCGGGTTGCGCCAAGGA------
CCATGAACGTTAGGCCTC--GCC-----TCTTTGC-GTTGTTACGCTGCGTTG-----AG-
GA-G--TC--CCATCATTGAA--AAATA-T-----------CAATTTAATGAC--
TCTTGACAAAGGATATCTCGGCTCTCGCATCGATGAAGAACG--TAGCGAAAT---
GCGATACTTGGTGTGAATTGCA-GAATCCTGTGAACCATTGA---
GTCTTTGAACGCAAGTT-
GCGCCCGATGCCTTTTGGCCAAGGGCACGTCTGCCTGGGCGTCACGCATAGCGTCGCCCCTA

CC-T-CCCAACTCGTGTTTGG--ATATGTGT--G-GGGC-
GGAGAGTGGTCTCCCGTGTGC---CTT-GCTCACATGGATGGCCGAAATAAGGAGCTT---
GCGGCGG--TGAGCGCCGTGATGATTGGTGG-TTGGAT-GGGC-TCTT-GCCCTACAA---
AGCATC----TTGTCATGAAGTGCCA-CCCTGTGTT-G-GCTCGGAGGACTCGGTCTGG--
TCGATGTTATCCGGATTTGTGCGC--C-TTTTATTGTCCAC-CAT
</sequence>
<sequence>
<taxon idref="Sirchensee"/>
TCGAAACCTGC-ATAGCAG-AGCAACATGCG--AACTTGTCTTTT-
CAACCTTCCTTTTTCCGTGTGGCGGGTCAGACGA-CT-------------CGTGCT-
TCCTC-CGCC-TTGCG-GGATGAACAA---CTTTC-GGCGCGGGTTGCGCCAAGGA------
CCATGAACGTTAGGCCTC--GCC-----TCTTTGC-GCCGTTAGGCTGCGTTG-----AG-
GA-G--TC--CCATCATTGAA--AAATA-T------------CAATTTAATGAC--
TCTTGACAAAGGATATCTCGGCTCTCGCATCGATGAAGAACG--TAGCGAAAT---
GCGATACTTGGTGTGAATTGCA-GAATCCTGTGAACCATTGA---
GTCTTTGAACGCAAGTT-
GCGCCCGAAGCCTTTTGGCCAAGGGCACGTCTGCCTGGGCGTCACGCATAGCGTCGCCCCTA
CC-T-CCCAACTCGTGTTTGG--ATGTGTGT--G-GGGC-
GGAGAGTGGTCTCCCGTGTGC---CTT-GCTCACATGGATGGCAGAAATAAGGAGCTT---
GCGGCGG--TGAGCGCCGTGATGATTGGTGG-TTGGAT-GGGC-TCTT-GCCCTACAA---
AGCATC----TTGTCATGAAGTGCCA-CCCCGTGTT-G-GCTCGGAGGACTCGGTCTGG--
TCGATGTTATCCAGATTTGTGCGC--C-TTTTATTGTCCAC-CAT
</sequence>
<sequence>
<taxon idref="Collareeee"/>
TCGAAACCTGC-ATAGCAG-AGCAACATGCG--AACTTGTCTTTT-
CAACCTTCCTTTTTCCGTGTGGCGGGTCAGACGA-CT-------------CGTGCT-
TCCTC-CGCC-TTGCG-GGATGAACAA---CTTTC-GGCGCGGGTTGCGCCAAGGA------
CCATGAACGTTAGGCCTC--GCC-----TCTTTGC-GCTGTTAGGCTGCGTTG-----AG-
GA-G--TC--CCATCATTGAA--AAATA-T------------CAATTTAATGAC--
TCTTGACAAAGGATATCTCGGCTCTCGCATCGATGAAGAACG--TAGCGAAAT---
GCGATACTTGGTGTGAATTGCA-GAATCCTGTGAACCATTGA---
GTCTTTGAACGCAAGTT-
GCGCCCGAAGCCTTTTGGCCAAGGGCACGTCTGCCTGGGCGTCACGCATAGCGTCGCCCCTA
CC-T-CCCAACTTGTGTTTGG--ATGTGTGT--G-GGGC-
GGAGAGTGGTCTCCCGTGTGC---CTT-GCTCACATGGATGGCCGAAATAAGGAGCTT---
GCGGCGG--TGAGCGCCGTGATGATTGGTGG-TTGGAT-GGGC-TCTT-GCCCTACAA---
AGCATC----TTGTCATGAAGTGCCA-CCCCGTGTT-G-GCTCGGAGGACTCGGTCTGG--
TCGATGTTATCCAGATTTGTGCGC--C-TTTTATTGTCCAC-CAT
</sequence>
<sequence>
<taxon idref="Heweriiiii"/>
TCGAAACCTGC-ATAGCAGAAGCAACATGCG--AACTTGTCTTTT-
CAACCTTCCTTTTTCCGTGTGGCGGGTCAGACGA-CT-------------CGTGCT-
TCCTC-CGCC-TTGCG-GGATGAACAA---CTTTC-GGCGCGGGTTGCGCCAAGGA------
CCATGAACGTTAGGCCTC--GCC-----TCTTTGC-GCTGTTAGGCTGCGTTG-----AG-
GA-G--TC--CCATCATTGAA--AAATA-T------------CAATTTAATGAC--
TCTTGACAAAGGATATCTCGGCTCTCGCATCGATGAAGAACG--TAGCGAAAT---
GCGATACTTGGTGTGAATTGCA-GAATCCTGTGAACCATTGA---
GTCTTTGAACGCAAGTT-
GCGCCCGAAGCCTTTTGGCCAAGGGCACGTCTGCCTGGGCGTCACGCATAGCGTCGCCCCTA
CC-T-CCCAACTTGTGTTTGG--ATGTGTGT--G-GGGC-
GGAGAGTGGTCTCCCGTGTGC---CTT-GCTCACATGGATGGCCGAAATAAGGAGCTT---
GCGGCGG--TGAGCGCCGTGATGATTGGTGG-TTGGAT-GGGC-TCTT-GCCCTACAA---
AGCATC----TTGTCATGAAGTGCCA-CCCCGTGTT-G-GCTCGGAGGACTCGGTCTGG--
TCGATGTTATCCAGATTTGTGCGC--C-TTTTATTGTCCAC-CAT

</sequence>
<sequence>
<taxon idref="Restiacumm"/>
TCGAAACCTGC-ATAGCAG-AGCAACATGCG--AACTTGTCTTTT-
CAACCTTCCTTTTTCCGTGTGGCGGGTCAGACGA-CT-------------CGTGCT-
TCCTC-CGCC-TTGCG-GGATGAACAA---CTTTC-GGCGCGGGTTGCGCCAAGGA------
CCATGAACGTTAGGCCTC--GCC-----TCTTTGC-GCCGTTAGGCTGCGTTG-----AG-
GA-G--TC--CCATCATTGAA--AAATA-T------------CAATTTAATGAC--
TCTTGACAAAGGATATCTCGGCTCTCGCATCGATGAAGAACG--TAGCGAAAT---
GCGATACTTGGTGTGAATTGCA-GAATCCTGTGAACCATTGA---
ATCTTTGAACGCAAGTT-
GCGCCCGAAGCCTTTTGGCCAAGGGCACGTCTGCCTGGGCGTCACGCATAGCGTCGCCCCTA
CC-T-CCCAACTCGTGTTTGG--ATGTGTAT--G-GGGA-
GGAGAGTGGTCTCCCGTGTGC---CTT-GCTCACATGGATGGCCGAAATAAGGAGCTT---
GCGGCGG--CGAGCGCTGTGATGATTGGTGG-TTGGAT-GGGC-TCTT-GCCCTACAA---
AGCATC----TTGTCATGAAGTGCCA-CCCCGTGTT-G-GCTTGGAGGACTCGGTCTGG--
TCGATGTTATCCAGATTTGTGCGC--C-TTTTATTGTCCAC-CAT
</sequence>
<sequence>
<taxon idref="Spinicaly1"/>
TCGAAACCTGC-ATAGCAG-AGCAACATGCG--AACTTGTCTTTT-
CAACCTTCC--TTTCCGTGTGGCGGGTCGGACGA-CT-------------CGTGCT-
TCCTC-CGCC-TTGCG-GGATGAACAA---CTTTC-GGCGCGGGTTGCGCCAAGGA------
CCATGAACGTTATGCCTC--GCC-----TCTTTGC-GCCGTTAGGCTGCGTTG-----AG-
GA-G--TC--ACATCATTGAA--AAATA-T------------CAATTTAATGAC--
TCTTGACAAAGGATATCTCGGCTCTCGCATCGATGAAGAACG--TAGCGAAAT---
GCGATACTTGGTGTGAATTGCA-GAATCCTGTGAACCATTGA---
GTCTTTGAACGCAAGTT-
GCGCCCGAAGCCTTTTGGCCAAGGGCACGTCTGCCTGGGTGTCACGCATAGCGTCGCCCCTA
CC-T-CCCAACTCGTGTTTGG--ATATGTGT--G-GGGC-
GGAGAGTGGTCTCCCGTGTGC---CTT-GCCCACATGGATGGCCGAAATAAGGAGCTT---
GCGGCGG--TGAGCGCCGTGATGATTGGTGG-TTGGATGGGGC-TCTT-GCCCTGCAA---
AGCATC----TTGTCATGAAGTGCCACCCCCGTGTT-G-GCTCGGAGGACTCGGTCTGG--
TCGATGTTATCCAGATTTGTGCGC--C-TTTTATTGTCCAC-CAT
</sequence>
<sequence>
<taxon idref="Gulistanum"/>
TCGAAACCTGC-ATAGCAG-AGCAACATGCG--AACTTGTCTTTT-
CAACCTTCC--TTTCCGTGTGGCGGGTCAGACGA-CT-------------CGTGCT-
TCCTC-CGCC-TTGCG-GGATGAACAA---CTTTC-GGCGCGGGTTGCGCCAAGGA------
CCATGAACGTTAGGCCTC--GCC-----TCTTTGC-GCCGTTAGGCTGCGTTG-----AG-
GA-G--TC--CCATCATTGAA--AAATA-T------------CAATTTAATGAC--
TCTTGACAAAGGATATCTCGGCTCTCGCATCGATGAAGAACG--TAGCGAAAT---
GCGATACTTGGTGTGAATTGCA-GAATCCTGTGAACCATTGA---
GTCTTTGAACGCAAGTT-
GCGCCCGAAGCCTTTTGGCCAAGGGCACGTCTGCCTGGGCGTCACGCATAGCGTCGCCCCTA
CC-T-CCCAACTCGTGTTTGG--ATATGTGT--G-GGGC-
GGAGAGTGGTCTCCCGTGTGC---CTT-GCTCACATGGATGGCCGAAATAAGGAGCTT---
GCGGCGG--TGAGCGCCGTGATGATTGGTGG-TTGGAT-GGGC-TCTT-GCCCTACAA---
AACATC----TTGTCATGAATTGTCA-CCCCGTGTT-G-GCTCGGAGGACTCCGTCTGG--
TCGATGTGATCCAGATTTGTGCGC--C-TTTTATTGTCCAC-CAT
</sequence>
<sequence>
<taxon idref="Austroiran"/>
TCGAAACCTGC-ATAGCAG-AGCAACATGCG--AACTTGTCTTTT-
CAACCTTCC--TTTCCGTGTGGCGGGTCGGACGA-CT-------------CGTGCT-

TCCTC-CGCC-TTACG-GGATGAACAA---CTTTC-GGCGCGGGTTGCGCCAAGGA------
CCATGAACGTTAGGCCTC--GCC-----TCTTTGC-GCCGTTAGGCTGCGTTG-----AG-
GA-G--TC--ACATCATTGAA--AAATA-T-----------CAATTTAATGAC--
TCTTGACAAAGGATATCTCGGCTCTCGCATCGATGAAGAACG--TAGCGAAAT---
GCGATACTTGGTGTGAATTGCA-CAATCCTGTGAACCATTGA---
GTCTTTGAACGCAAGTT-
GCGCCCGAAACCTTTTGGCCAAGGGGACGTCTGCCTGGGTGTCACGCATAGCGTCGCCCCTA
CC-T-CCCAACTCGTGTTTGG--ATATGTGT--G-GGGC-
GGAGAGTGGTCTCCCGTGTGC---CTT-GCCCACATGGATGGCCGAAATAAGGAGCTT---
GCGGCGG--TGAGCGCCGTGATGATTGGTGG-TTGGATGGGGC-TCTT-GCCCTACAA---
AGCATC----TTGTCATGAAGTGCCACCCCCGTGTT-G-GCTCGGAGGACTCGGTCTGG--
TCGATGTTATCCAGATTTGTGCGC--C-TTCTATTGTCCAC-CAT
</sequence>
<sequence>
<taxon idref="Schahrudic"/>
TCGAAACCTGC-ATAGCAG-AGCAACATGCG--AACTTGTCTTTT-
CAACCTTCCTTTTTCCGTGTGGCGGGTCAGACGA-CT------------CGTGCT-
TCCTC-CGCC-TTGCG-GGATGAACAA---CTTTC-GGCGCGGGTTGCGCCAAGGA------
CCATGAACGTTAGGCCTC--GCC-----TCTTTGC-GCCGTTAGGCTGCGTTG-----AG-
GA-G--TC--CCATCATTGAA--AAATA-T-----------CAATTTAATGAC--
TCTTGACAAAGGATATCTCGGCTCTCGCATCGATGAAGAACG--TAGCGAAAT---
GCGATACTTGGTGTGAATTGCA-GAATCCTGTGAACCATTGA---
GTCTTTGAACGCAAGTT-
GCGCCCGAAGCCTTTTGGCCAAGGGCACGTCTGCCTGGGCGTCACGCATAGCGTCGCCCCTA
CC-T-CCCAACTCGTGTTTGG--ATGTGTGT--G-GGGC-
GGAGAGTGGTCTCCCGTGTGC---CTT-GCTCACATGGATGGCAGAAATAAGGAGCTT---
GCGGCGG--TGAGCGCCGTGATGATTGGTGG-TTGGAT-GGGC-TCTT-GCCCTACAA---
AGCATC----TTGTCATGAAGTGCCA-CCCCGTGTT-G-GCTCGGAGGACTCGGTCTGG--
TCGATGTTATCCAGATTTGTGCGC--C-TTTTATTGTCCAC-CAT
</sequence>
<sequence>
<taxon idref="Cephaltoid"/>
TCGAAACCTGC-ATAGCAG-AGCAACATGCG--AACTTGTCTTTT-
CAACCTTCC--TTTCCGTGTGGCGGGTCTGAAGA-CT------------CGTGCT-
TCCTC-CGCC-TTGCG-GGATGAACAA---TTTCC-GGCGCGGGTTGCGCCAAGGA------
CCATGAACGTTAGGCCTT--GCC-----TCTTTGC-GCCGTTAGGCTGCGTTG-----AG-
GA-G--CC--CCACCATTGAA--AACTA-T-----------CAATTGAATGAC--
TCTCGACAAAGGATATCTCGGCTCTCGCATCGATGAAGAACG--TAGCGAAAT---
GCGATACTTGGTGTGAATTGCA-GAATCCTGTGAACCATTGA---
TTCTTTGAACGCAAGTT-
GCGCCTGAAGCCTTTTGGCCAAGGGCACGTCTGCCTGGGCGTCACGCATCGCGTCGCCCCTA
CC-C-CCCAACTCGTGTTTGG--ATATGTGT--A-GGGC-
GGAGAGTGGCCTCCCGTGTGGCT-CTT-GCTGACATGGATGGCCGAAATAAGGAGCTT---
GTGGCAG--CGAGCGCTGTGATGATTGGTGG-CTGGAC-GGGC-TCTT-GCCTTACAA---
AGCATC----TTGTCACGAAGCGCCG-CCCCGTGTT-G-GCTTGGAGGACTCGACCTGG--
TCGATGTTATCCCGATTTGTGTGG--C-TTTTATCGTCCAC-CAT
</sequence>
<sequence>
<taxon idref="Demawendic"/>
TCGAAACCTGC-ATAGCAG-AGCAACATGCG--AACTTGTCTTTT-
CAACCTTCC--TTTCCGTGTGGCGGGTCTGAAGA-CT------------CGTGCT-
TCCTC-CGCC-TTGCG-GGATGAACAA---TTTCC-GGCGCGGGTTGCGCCAAGGA------
CCATGAACGTTAGGCCTT--GCC-----TCTTTGC-GCCGTTAGGCTGCGTTG-----AG-
GA-G--CC--CCACCATTGAA--AACTA-T-----------CAATTGAATGAC--
TCTCGACAAAGGATATCTCGGCTCTCGCATCGATGAAGAACG--TAGCGAAAT---
GCGATACTTGGTGTGAATTGCA-GAATCCTGTGAACCATTGA---

TTCTTTGAACGCAAGTT-
GCGCCTGAAGCCTTTTGGCCAAGGGCACGTCTGCCTGGGCGTCACGCATCGCGTCGCCCCTA
CC-C-CCCAACTCGTGTTTGG--ATATGTGT--A-GGGC-
GGAGAGTGGCCTCCCGTGTGGCT-CTT-GCTGACATGGATGGCCGAAATAAGGAGCTT---
GTGGCAG--CGAGCGCTGTGATGATTGGTGG-CTGGAC-GGGC-TCTT-GCCTTACAA---
AGCATC----TTGTCACGAAGCGCCG-CCCCGTGTT-G-GCTTGGAGGACTCGACCTGG--
TCGATGTTATCCCGGTTTGTGTGG--C-TTTTATCGTCCAC-CAT
</sequence>
<sequence>
<taxon idref="Gorganense"/>
TCGAAACCTGC-ATAGCAG-AGCAACATGCG--AACTTGTCTTTT-
CAACCTTCC--TTTCCGTCTGGCGGGTCAGACGA-CT------------CGTGCT-
TCCTC-CGCC-TTACG-GGATGAACAA---CTTTC-GGCGCGGGTTGCGCCAAGGA------
CCATGAACGTTAGGCCTC--GCC-----TCTTTGC-GTTGTTAGGCTGCGTTG-----AG-
GA-G--TC--CCATCATTGAA--AAATA-T-----------CAATTTAATGAC--
TCTTGACAAAGGATATCTCGGCTCTCGCATCGATGAAGAACG--TAGCGAAAT---
GCGATACTTGGTGTGAATTGCA-GAATCCTGTGAACCATTGA---
GTCTTTGAACGCAAGTT-
GCGCCCGATGCCTTTTGGCCAAGGGCACGTCTGCCTGGGCGTCACGCATAGCGTCGCCCCTA
CC-T-CCCAACTCGTGTTTGG--ATATGTGT--G-GGGC-
GGAGAGTGGTCTCCCGTGTGC---CTT-GCTCACATGGATGGCCGAAATAAGGAGCTT---
GCGGCGG--TGAGCGCCGTGATGATTGGTGG-TTGGAT-GGGC-TCTT-GCCCTACAA---
AGCATC----TTGTCATGAAGTGCCA-CCCTGTGTT-G-GCTCGGAGGACTCGGTCTGG--
TCGATGTTATCCGGATTTGTGCGC--C-TTTTATTGTCCAC-CAT
</sequence>
<sequence>
<taxon idref="Heratensee"/>
TCGAAACCTGC-ATAGCAG-AGCAACATGCG--AACTTGTCTTTT-
CAACCTTCC--TTTCCGTGTGGCGGGTCGGATGA-CT------------CGTGCT-
TCCTC-CGCC-TTGCG-GGATGAACAA---CTTTC-GGCGCGGGTTGCGCCAAGGA------
CCATGAACGTTATGCCTC--GCC-----TCTTTGC-GCCGTTAGGCTGCGTTG-----AG-
GA-G--TC--ACATCATTGAA--AAATA-T-----------CAATTTAATGAC--
TCTTGACAAAGGATATCTCGGCTCTCGCATCGATGAAGAACG--TAGCGAAAT---
GCGATACTTGGTGTGAATTGCA-GAATCCTGTGAACCATTGA---
GTCTTTGAACGCAAGTT-
GCGCCCGAAGCCTTTTGGCCAAGGGCACGTCTGCCTGGGTGTCACGCATAGCGTCGCCCCTA
CC-T-CCCAACTCGTGTTTGG--ATATGTGT--G-GGGC-
GGAGAGTGGTCTCCCGTGTGC---CTT-GCCCACATGGATGGCCGAAATAAGGAGCTT---
GCGGCGG--TGAGCGCCGTGATGATTGGTGG-TTGGATGGGGC-TCTT-GCCCTGCAA---
AGCATC----TTGTCATGAAGTGCCACCCCCGTGTT-G-GCTCGGAGGACTCGGTCTGG--
TCGATGTTATCCAGATTTGTGCGC--C-TTTTATTGTCCAC-CAT
</sequence>
<sequence>
<taxon idref="Horridummm"/>
TCGAAACCTGC-ATAGCAG-AGCAACATGCG--AACTTGTCTTTT-
CAACCTTCCTTTTTCCGTGTGGCGGGTCAGACGA-CT------------CGTGCT-
TCCTC-CGCC-TTGCG-GGATGAACAA---CTTTC-GGCGCGGGTTGCGCCAAGGA------
CCATGAACGTTAGGCCTC--GCC-----TCTTTGC-GCCGTTAGGCTGCGTTG-----AG-
GA-G--TC--CCATCATTGAA--AAATA-T-----------CAATTTAATGAC--
TCTTGACAAAGGATATCTCGGCTCTCGCATCGATGAAGAACG--TAGCGAAAT---
GCGATACTTGGTGTGAATTGCA-GAATCCTGTGAACCATTGA---
GTCTTTGAACGCAAGTT-
GCGCCCGAAGCCTTTTGGCCAAGGGCACGTCTGCCTGGGCGTCACGCATAGCGTCGCCCCTA
CC-T-CCCAACTCGTGTTTGG--ATGTGTGT--G-
GGGCGGGAGAGTGGTCTCCCGTGTGC---CTT-
GCTCACATGGATGGCAGAAATAAGGAGCTT---GCGGCGG--

TGAGCGCCGTGATGATTGGTGG-TTGGATGGGGC-TCTT-GCCCTACAA---AGCATC----
TTGTCATGAAGTGACA-CCCCGTGTT-G-GCTCGGAGGACTCGGTCTGG--
TCGATGTTATCCAGATTTGTGCGC--C-TTTTATTGTCCAC-CAT
</sequence>
<sequence>
<taxon idref="Rodopolium"/>
TCGAAACCTGC-ATAGCAG-AGCAACATGCG--AACTTGTCTTTT-
CAACCTTCC--TTTCCGTGTGGCGGGTCGGACGA-CT------------CGTGCT-
TCCTC-CGCC-TTACG-GGATGAACAA---CTTTC-GGCGCGGGTTGCGCCAAGGA------
CCATGAACGTTAGGCCTC--GCC-----TCTTTGC-GCCGTTAGGCTGCGTTG-----AG-
GA-G--TC--ACATCATTGAA--AAATA-T-----------CAATTTAATGAC--
TCTTGACAAAGGATATCTCGGCTCTCGCATCGATGAAGAACG--TAGCGAAAT---
GCGATACTTGGTGTGAATTGCA-GAATCCTGTGAACCATTGA---
GTCTTTGAACGCAAGTT-
GCGCCCGAAGCCTTTTGGCCAAGGGCACGTCTGCCTGGGTGTCACGCATAGCGTCGCCCCTA
CC-T-CCCAACTCGTGTTTGG--ATATGTGT--G-GGGC-
GGAGAGTGGTCTCCCGTGTGC---CTT-GCCCACATGGATGGCCGAAATAAGGAGCTT---
GCGGCGG--TGAGCGCCGTGATGATTGGTGG-TTGGATGGGGC-TCTT-GCCCTACAA---
AGCATC----TTGTCATGAAGTGCCACCCCCGTGTT-G-GCTCGGAGGACTCGGTCTGG--
TCGATGTTATCCAGATTTGTGCGC--C-TTCTATTGTCCAC-CAT
</sequence>
<sequence>
<taxon idref="Tragacanth"/>
TCGAAACCTGC-ATAGCAG-AGCAACATGCG--AACTTGTCTTTT-
CAACCTTCC--TTTCCGTGTGGCGGGTCGGACGA-CT------------CGTGCT-
TCCTC-CGCC-TTACG-GGATGAACAA---CTTTC-GGCGCGGGTTGCGCCAAGGA------
CCATGAACGTTAGGCCTC--GCC-----TCTTTGC-GCCGTTAGGCTGCGTTG-----AG-
GA-G--TC--ACATCATTGAA--AAATA-T-----------CAATTTAATGAC--
TCTTGACAAAGGATATCTCGGCTCTCGCATCGATGAAGAACG--TAGCGAAAT---
GCGATACTTGGTGTGAATTGCA-GAATCCTGTGAACCATTGA---
GTCTTTGAACGCAAGTT-
GCGCCCGAAGCCTTTTGGCCAAGGGCACGTCTGCCTGGGTGTCACGCATAGCGTCGCCCCTA
CC-T-CCCAAATCGTGTTTGG--ATATGTGT--G-GGGC-
GGAGAGTGGTCTCCCGTGTGC---CTT-GCCCACATGGATGGCCGAAATAAGGAGCTT---
GCGGCGG--TGAGCGCCGTGATGATTGGTGG-TTGGATGGGGC-TCTT-GCCCTACAA---
AGCATC----TTGTCATGAAGTGCCACCCCCGTGTT-G-GCTCGGAGGACTCGGTCTGG--
TCGATGTTATCCAGATTTGTGCGC--C-TTCTATTGTCCAC-CAT
</sequence>
<sequence>
<taxon idref="Atropatanu"/>
TCGAAACCTGC-ATAGCAG-AGCAACATGCG--AACTTGTCTTTT-
CAACCTTCC--TTTCCGTGTGGCGGGTCAGAAGA-CT------------CGTGCT-
TCCTC-CGCC-TTGCG-GGATGAACAA---TTTTC-GGCGCGGGTTGCGCCAAGGA------
CCATGAACGTTAGGCCTT--GCC-----TCTTTGC-GCCGTTAGGCTGCGTTG-----AG-
GA-G--CC--CCATCATTGAA--AACTA-T-----------CTATTGAATGAC--
TCTCGACAAAGGATATCTCGGCTCTCGCATCGATGAAGAACG--TAGCGAAAT---
GCGATACTTGGTGTGAATTGCA-GAATCCTGTGAACCATTGA---
TTCTTTGAACGCAAGTT-
GCGCCTGAAGCCTTTTGGCCAAGGGCACGTCTGCCTGGGCGTCACGCATCGCGTCGCCCCTA
CC-T-CCCAACTCGTGTTTGG--ATATGTGT--G-GGGC-
GGAGAGTGGCCTCCCGTGTGCCT-CTT-GCTCACATGGATGGCCGAAATAAGGAGCTT---
GTGGCAG--CGAGCGCTGTGATGATTGGTGG-TTGGAT-GGGC-TTTT-GCCYTACAA---
AGCATC----TTGTCACGAAGCGCCG-CCCCGTGTT-G-GCTTGGAGGACTCGACCTGG--
TCGATGTTRTCCCGATTTGTGTGC--C-TTTTATCGTCCAC-CAT
</sequence>
<sequence>

<taxon idref="Sahendicu2"/>
TCGAAACCTGC-ATAGCAG-AGCAACATGCG--AACTTGTCTTTT-
CAACCYTCCTTTTTCCGTGTGGCGGGTCAGACGA-CT-------------CGTGTC-
TCCTC-CGCC-TTGCG-GGATGAACAA---CTTTC-GGCGCGGGTTGCGCCAAGGA------
CCATGAACGTTAGGCCTC--GCC-----TCTTTGC-GCCGTTAGGCTGCGTTG-----AG-
GA-G--TC--CCATCATTGAA--AAATA-T------------CAATTTAATGAC--
TCTTGACAAAGGATATCTCGGCTCTCGCATCGATGAAGAACG--TAGCGAAAT---
GCGATACTTGGTGTGAATTGCA-GAATCCTGTGAACCATTGA---
GTCTTTGAACGCAAGTT-
GCGCCCGACGCCTTTTGCCCAAGGGCACGTCTGCCTGGGCGTCACGCATAGCGTCGCCCCTA
CC-T-CCCAACTCGTGTTTGG--ATGTGTGT--G-GGGC-
GGAGAGTGGTCTCCCGTGTGC---CTT-GCTCACATGGATGGCMGAAATAAGGAGCTT---
GCGGCGG--TGAGCGCCGTGATGATTGGTGG-TTGGAT-GGGC-TCTT-GCCCTACAA---
AGCATC----TTGTCATGAAGTGCCA-CCCCGTGTT-G-GCTCGGAGGACTCGGTCTGG--
TCGATGTTATCCAGATTTGTGCGC--C-TTTTATTGTCTAC-CAT
</sequence>
<sequence>
<taxon idref="Latifolium"/>
TCGAAACCTGC-ATAGCAG-AGCAACATGCG--AACTTGTCTTCT-
CAACCTTCC--TTTCCGTGTGGCGGGTCAGAAGA-CT-------------CGTGCT-
TCCTC-CGCC-TTGCG-GGATGAACAA---TTTTC-GGCGCGGGTTGCGCCAAGGA------
CCATGAACGTTAGGCGTT--GCC-----TCTTTGC-GCCGTTAGGCTGCGTTG-----AG-
GA-G--CC--CCATCATTGAA--AACTA-T------------CTATTGAATGAC--
TCTCGACAAAGGATATCTCGGCTCTCGCATCGATGAAGAACG--TAGCGAAAT---
GCGATACTTGGTGTGAATTGCA-GAATCCTGTGAACCATTGA---
TTCTTTGAACGCAAGTT-
GCGCCTGAAGCCTTTTGGCCAAGGGCACGTCTGCCTGGGCGTCACGCATCGCGTCGCCCCTA
CC-T-CCCAACTCGTGTTTGG----ATGTGT--G-GGGC-
GGAGAGTGGCCTCCCGTGTGCCT-CTT-GCTCACATGGATGGCCGAAATAAGGAGCTT---
GTGGCAG--CGAGCGCTGTGATGATTGGTGG-TTGGAT-GGGC-TTTTTGCCTTACAA---
AGCATC----TTGTCACGAAGCGCCG-CCCCGTGTT-G-GCTTGGAGGACTCGACCTGG--
TCGATGTTGTCCCGATTTGTGTGC--C-TTTTATCGTCCAC-CAT
</sequence>
<sequence>
<taxon idref="Asphodelin"/>
TCGAAACCTGC-ATAGCAG-AGCAACATGCG--AACTTGTCTTTT-
CAACCTTCC--TTTCCGTGTGGCGGGTTAGAAGA-CT-------------CGTGCT-
TCCTC-CGCC-TTGCG-GGATGAACAA---TTTTC-GGCGCGGGTTGCGCCAAGGA------
CCATGAACGTTAGGCCTT--GCC-----TCTTTGC-GCAGTTAGGATGCGTTG-----AG-
GA-G--CC--CCATCATTGAA--AACTA-T------------CTATTGAACGAC--
TCTCGACAAAGGATATCTCGGCTCTCGCATCGATGAAGAACG--TAGCGAAAT---
GCGATACTTGGTGTGAATTGCA-GAATCCTGTGAACCATTGA---
TTCTTTGAACGCAAGTT-
GCGCCTGAAGCCTTTTGGCCAAGGGCACGTCTGCCTGGGCGTCACGCATCGCGTCGCCCCTA
CC-T-CCCAACTCGTGTTTGG--ATATGTGT--G-GGGC-
GGAGAGTGGCCTCCCGTGTGCCT-CTT-GCTCACATGGATGGCCGAAATAAGGAGCTT---
GTGGCAG--CGAGCGCTGTGATGATTGGTGG-TTGGAT-GGGC-TCTT-GCCTTACAA---
AGCATC----TTGTCACGAAGCGCCG-CCCCGTGTT-G-GCTTGCAGGACTCGACCTGG--
TCGATGTTATCCCGATTTGTGTGC--C-TTTTATCGTCCAC-CAT
</sequence>
<sequence>
<taxon idref="Acmostegi1"/>
TCGAAACCTGC-ATAGCAG-AGCAACATGCG--AACTTGTCTTTT-
CAACCTTCC--TTTCCGTGTGGCGGGTCGGACGA-CT-------------CGTGCT-
TCCTC-CGCC-TTGCG-GGATGAACAA---CTTTC-GGCGCGGGTTGCGCCAAGGA------
CCATGAACGTTAGGCCTC--GCC-----TCTTTGC-GCCGTTAGGCTGCGTTG-----AG-

GA-G--TC--ACATCATTGAA--AAATA-T-----------CAATTTAATGAC--
TCTTGACAAAGGATATCTCGGCTCTCGCATCGATGAAGAACG--TAGCGAAAT---
GCGATACTTGGTGTGAATTGCA-GAATCCTGTGAACCATTGA---
GTCTTTGAACGCAAGTT-
GCGCCCGAAGCCTTTTGGCCAAGGGCACGTCTGCCTGGGTGTCACGCATAGCGTCGCCCCTA
CC-T-CCCAACTCGTGTTTGG--ATATGTGT--G-GGGC-
GGAGAGTGGTCTCCCGTGTGC---CTT-GCCCACATGGATGGCCGAAATAAGGAGCTT---
GCGGCGG--TGAGCGCCGTGATGATTGGTGG-TTGGATGGGGC-TCTT-GCCCTACAA---
AGCATC----TTGTCATGAAGTGCTACCCCCGTTTT-G-GCTCGGAGGACTCGGTCTGG--
TCGATGTTATCCAGATTTGTGCGC--C-TTTTATTGTCCAC-CAT
</sequence>
<sequence>
<taxon idref="Brachystac"/>
TCGAAACCTGC-ATAGCAG-AGCAACATGCG--AACTTGTCTTTT-
CAACCTTCC--TTTCCGTGTGGCGGGTTAGAAGA-CT-------------CGTGCT-
TCCTC-CGCC-TTGCG-GGATGAACAA---TTTTC-GGCGCGGGTTGCGCCAAGGA------
CCATGAACGTTAGGCCTT--GCC-----TCTTTGC-GCAGTTAGGATGCGTTG-----AG-
GA-G--CC--CCATCATTGAA--AACTA-T-----------CTATTGAATGAC--
TCTCGACAAAGGATATCTCGGCTCTCGCATCGATGAAGAACG--TAGCGAAAT---
GCGATACTTGGTGTGAATTGCA-GAATCCTGTGAACCATTGA---
TTCTTTGAACGCAAGTT-
GCGCCTGAAGCCTTTTGGCCAAGGGCACGTCTGCCTGGGCGTCACGCATCGCGTCGCCCCTA
CC-T-CCCAACTCGTGTTTGG--ATATGTGT--G-GGGC-
GGAGAGTGGCCTCCCGTGTGCCT-CTT-GCTCACATGGATGGCCGAAATAAGGAGCTT---
GTGGCAG--CGAGCGCTGTGATGATTGGTGG-TTGGAT-GGGC-TCTT-GCCTTACAA---
AGCATC----TTGTCACGAAGCGCCG-CCCCGTGTT-G-GCTTGCAGGACTCGACCTGG--
TCGATGTTATCCCGATTTGTGTGC--C-TTTTATCGTCCAC-CAT
</sequence>
<sequence>
<taxon idref="Caryophyll"/>
TCGAAACCTGC-ATAGCAG-AGCAACATGCG--AACTTGTCTTCT-
CAACCTTCC--TTTCCGTGTGGCGGGTCAGAAGA-CT-------------CGTGCT-
TCCTC-CGCC-TTGCG-GGATGAACAA---TTTTC-GGCGCGGGTTGCGCCAAGGA------
CCATGAACGTTAGGCGTT--GCC-----TCTTTGC-GCCGTTAGGCTGCGTTG-----AG-
GA-G--CC--CCATCATTGAA--AACTA-T-----------CTATTGAATGAC--
TCTCGACAAAGGATATCTCGGCTCTCGCATCGATGAAGAACG--TAGCGAAAT---
GCGATACTTGGTGTGAATTGCA-GAATCCTGTGAACCATTGA---
TTCTTTGAACGCAAGTT-
GCGCCTGAAGCCTTTTGGCCAAGGGCACGTCTGCCTGGGCGTCACGCATCGCGTCGCCCCTA
CC-T-CCCAACTCGTGTTTGG--ATATGTGT--G-GGGC-
GGAGAGTGGCCTCCCGTGTGCCT-CTT-GCTCACATGGATGGCCGAAATAAGGAGCTT---
GTGGCAG--CGAGCGCTGTGATGATTGGTGG-TTGGAT-GGGC-TTTTTGCCTTACAA---
AGCATC----TTGTCACGAAGCGCCG-CCCCGTGTT-G-GCTTGGAGGACTCGACCTGG--
TCGATGTTGTCCCGATTTGTGTGC--C-TTTTATCGTCCAC-CAT
</sequence>
<sequence>
<taxon idref="Cupreoliva"/>
TCGAAACCTGC-ATAGCAG-AGCAACATGCG--AACTTGTCTTTT-
CAACCTTCCTTTTTCCGTGTGGCGGGTCAGACGA-CT-------------CGTGCT-
TCCTC-CGCC-TTGCG-GGATGAACAA---CTTTC-GGCGCGGGTTGCGCCAAGGA------
CCATGAACGTTAGGCCTC--GCC-----TCTTTGC-GCCGTTAGGCTGCGTTG-----GG-
GA-G--TC--CCATCATTGAA--AAATA-T-----------CAATTTAATGAC--
TCTTGACAAAGGATATCTCGGCTCTCGCATCGATGAAGAACG--TAGCGAAAT---
GCGATACTTGGTGTGAATTGCA-GAATCCTGTGAACCATTGA---
GTCTTTGAACGCAAGTT-
GCGCCCGAAGCCTTTTGGCCAAGGGCACGTCTGCCTGGGCGTCACGCATAGCGTCGCCCYTA

CC-T-CCCAACTCGTGTTTGG--ATGTGTGT--G-GGGC-
GGAGAGTGGTCTCCCGTGTGC---CTT-GCTCACATGGATGGCAGAAATAAGGAGCTT---
GCGGCGG--TGAGCGCCGTGATGATTGGTGG-TTGGAT-GGGC-TCTT-GCCCTACAA---
AGCATC----TTGTCATGAAGTGCCA-CCCCGTGTT-G-GCTCGGAGGACTCGGTCTGG--
TCGATGTTATCCAGATTTGTGCGC--C-TTTTATTGTCCAC-CAT
</sequence>
<sequence>
<taxon idref="Eschkerese"/>
TCGAAACCTGC-ATAGCAG-AGCAACATGCG--AACTTGTCTTTT-
CAACCTTCC--TTTCCGTGTGGCGGGTTAGAAGA-CT------------CGTGCT-
TCCTC-CGCC-TTGCG-GGATGAACAA---TTTTC-GGCGCGGGTTGCGCCAAGGA------
CCATGAACGTTAGGCCTT--GCC-----TCTTTGC-GCAGTTAGGATGCGTTG-----AG-
GA-G--CC--CCATCATTGAA--AACTA-T-----------CTATTGAATGAC--
TCTCGACAAAGGATATCTCGGCTCTCGCATCGATGAAGAACG--TAGCGAAAT---
GCGATACTTGGTGTGAATTGCA-GAATCCTGTGAACCATTGA---
TTCTTTGAACGCAAGTT-
GCGCCTGAAGCCTTTTGGCCAAGGGCACGTCTGCCTGGGCGTCACGCATCGCGTCGCCCCTA
CC-T-CCCAACTCGTGTTTGG--ATATGTGT--G-GGGC-
GGAGAGTGGCCTCCCGTGTGCCT-CTT-GCTCACATGGATGGCCGAAATAAGGAGCTT---
GTGGCAG--CGAGCGCTGTGATGATTGGTGG-TTGGAT-GGGC-TCTT-GCCTTACAA---
AGCATC----TCGTCACGAAGCGCCG-CCCCGTGTT-G-GCTTGCAGGACTCGACCTGG--
TCGATGTTATCCCGATTTGTGTGC--C-TTTTATCGTCCAC-CAT
</sequence>
<sequence>
<taxon idref="Melananthu"/>
TCGAAACCTGC-ATAGCAG-AGCAACATGCG--AACTTGTCTTTT-
CAACCTTCC--TTTCCGTGTGGCGGGTTAGAAGA-CT------------CGTGCT-
TCCTC-CGCC-TTGCG-GGATGAACAA---TTTTC-GGCGCGGGTTGCGCCAAGGA------
CCATGAACGTTAGGCCTT--GCC-----TCTTTGC-GCAGTTAGGATGCGTTG-----AG-
GA-G--CC--CCATCATTGAA--AACTA-T-----------CTATTGAAYGAC--
TCTCGACAAAGGATATCTCGGCTCTCGCATCGATGAAGAACG--TAGCGAAAT---
GCGATACTTGGTGTGAATTGCA-GAATCCTGTGAACCATTGA---
TTCTTTGAACGCAAGTT-
GCGCCTGAAGCCTTTTGGCCAAGGGCACGTCTGCCTGGGCGTCACGCATCGCGTCGCCCCTA
CC-T-CCCAACTCGTGTTTGG--ATATGTGT--G-GGGC-
GGAGAGTGGCCTCCCGTGTGCCT-CTT-GCTCACATGGATGGCCGAAATAAGGAGCTT---
GCGGCAG--CGAGCGCTGTGATGATTGGTGG-TTGGAT-GGGC-TCTT-GCCTTACAA---
AGCATC----TTGTCACGAAGCGCCG-CCCCGTGTT-G-GCTTGCAGGACTCGACCTGG--
TCGATGTTATCCCGATTTGTGTGC--C-TTTTATCGTCCAC-CAT
</sequence>
<sequence>
<taxon idref="Mischodage"/>
TCGAAACCTGC-ATAGCAG-AGCAACATGCG--AACTTGTCTTTT-
CAACCTTCC--TTTCCGTGTGGCGGGTCAGACGA-CT------------CGTGCT-
TCCTC-CGCC-TCGCG-GGATGAACAA---CTTTC-GGCGCGGGTTGCGCCAAGGA------
CCATGAACGTTAGGCCTT--GCC-----TCTTTGC-GCCGTTAGGCTGCGTTG-----AG-
GA-G--TC--CCATCATTGAA--CAATA-T-----------CAATTTAATGAC--
TCTTGACAAAGGATATCTCGGCTCTCGCATCGATGAAGAACG--TAGCGAAAT---
GCGATACTTGGTGTGAATTGCA-GAATCCTGTGAACCATTGA---
GTCTTTGAACGCAAGTT-
GCGCCCGAAGCCTTTTGGCCAAGGGCACGTCTGCCTGGGCGTCACGCATAGCGTCGCCCCTA
CC-T-CCCAACTCGTGTTTGG--ATATGTGT--G-GGGC-
GGAGAGTGGTCTCCCGTGTGCCT-CTT-GCTCACATGGATGGCCGAAATAAGGAGCTT---
GCGGCGG--TGAGCGCCGTGATGATTGGTGG-TTGGAT-GGGC-TCTT-GCCCTACAA---
AGCATC----TTGTCACGAAGTGCCA-CCCCGTGTT-G-GCTCGGAGGACTCGGTCTGG--
TCGATGTTATCCAGATTTGTGGGC--C-TTTTATTGTCCAC-CAT

</sequence>
<sequence>
<taxon idref="Nigricanse"/>
TCGAAACCTGC-ATAGCAG-AGCAACATGCG--AACTTGTCTTTT-
CAACCTTCC--TTTCCGTGTGGCGGGTTAGAAGA-CT-------------CGTGCT-
TCCTC-CGCC-TTGCG-GGATGAACAA---TTTTC-GGCGCGGGTTGCGCCAAGGA------
CCATGAACGTTAGGCCTT--GCC-----TCTTTGC-GCAGTTAGGATGCGTTG-----AG-
GA-G--CC--CCATCATTGAA--AACTA-T------------CTATTGAATGAC--
TCTCGACAAAGGATATCTCGGCTCTCGCATCGATGAAGAACG--TAGCGAAAT---
GCGATACTTGGTGTGAATTGCA-GAATCCTGTGAACCATTGA---
TTCTTTGAACGCAAGTT-
GCGCCTGAAGCCTTTTGGCCAAGGGCACGTCTGCCTGGGCGTCACGCATCGCGTCGCCCCTA
CC-T-CCCAACTCGTGTTTGG--ATATGTGT--G-GGGC-
GGAGAGTGGCCTCCCGTGTGCCT-CTT-GCTCACACGGATGGCCGAAATAAGGAGCTT---
GTGGCAG--CGAGCGCTGTGATGATTGGTGG-TTGGAT-GGGC-TCTT-GCCTTACAA---
AGCATC----TTGTCACGAAGCGCCG-CCCCGTGTT-G-GCTTGCAGGACTCGACCTGG--
TCGATGTTATCCCGATTTGTGTGC--C-TTTTATCGTCCAC-CAT
</sequence>
<sequence>
<taxon idref="Fominiiiii"/>
TCGAAACCTGC-ATAGCAG-AGCAACATGCG--AACTTGTCTTTT-
CAACCTTCC--TTTCCGTGTGGCGGGTCAGACGA-CT-------------CGTGCT-
TCCTC-CGCC-TCGCG-GGATGAACAA---CTTTC-GGCGCGGGTTGCGCCAAGGA------
CCATGAACGTTAGGCCTT--GCC-----TCTTTGC-GCCGTTAGGCTGCGTTG-----AG-
GA-G--TC--CCATCATTGAA--CAATA-T------------CAATTTAATGAC--
TCTTGACAAAGGATATCTCGGCTCTCGCATCGATGAAGAACG--TAGCGAAAT---
GCGATACTTGGTGTGAATTGCA-GAATCCTGTGAACCATTGA---
GTCTTTGAACGCAAGTT-
GCGCCCGAAGCCTTTTGGCCAAGGGCACGTCTGCCTGGGCGTCACGCATAGCGTCGCCCCTA
CC-T-CCCAACTCGTGTTTGG--ATATGTGT--G-GGGC-
GGAGAGTGGTCTCCCGTGTGCCT-CTT-GCTCACATGGATGGCCGAAATAAGGAGCTT---
GCGGCGG--TGAGCGCCGTGATGATTGGTGG-TTGGAT-GGGC-TCTT-GCCCTACAA---
AGCATC----TTGTCACGAAGTGCCA-CCCYGTGTT-G-GCTCGGAGGACTCGGTCTGG--
TCGATGTTATCCAGATTTGTGGGC--C-TTTTATTGTCCAC-CAT
</sequence>
<sequence>
<taxon idref="Olivieri1i"/>
TCGAAACCTGC-ATAGCAG-AGCAACATGCG--AACTTGTCTTTT-
CAACCTTCC--TTTCCGTGTGGCGGGTCAGAAGA-CT-------------CGTGCT-
TCCTC-CGCC-TTGCG-GGATGAACAA---TTTTC-GGCGCGGGTTGCGCCAAGGA------
CCATGTACGTTAGGCCTT--GCC-----TCTTTGC-GCCGTTAGGCTGCGTTG-----AG-
GA-G--CC--CCATCATTGAA--AACTA-T------------CTATTGAATGAC--
TCTCGACAAAGGATATCTCGGCTCTCGCATCGATGAAGAACG--TAGCGAAAT---
GCGATACTTGGTGTGAATTGCA-GAATCCTGTGAACCATTGA---
TTCTTTGAACGCAAGTT-
GCGCCTGAAGCCTTTTGGCCAAGGGCACGTCTGCCTGGGCGTCACGCATCGCGTCGCCCCTA
CC-T-CCCAACTCGTGTTTGG--ATATGTGT--G-GGGC-
GGAGAGTGGCCTCCCGTGTGCCT-CTT-GCTCACATGGATGGCCGAAATAAGGAGCTT---
GTGGCAG--CGAGCGCTGTGATGATTGGTGG-TTGGAT-GGGC-TTTT-GCCTTACAA---
AGCATC----TTGTCACGAAGCGCCG-CCCCGTGTT-G-GCTTGGAGGACTCGACCTGG--
TCGATGTTATCCCGRTTTGTGTGC--C-TTTTATCGTCCAC-CAT
</sequence>
<sequence>
<taxon idref="Ophiocladu"/>
TCGAAACCTGC-ATAGCAG-AGCAACATGCR--AACTTGTCTTTT-
CAACCTTCC--TTTCCGTGTGGCGGGTTAGAAGA-CT-------------CGTGCT-

TCCTC-CGCC-TTGCG-GGATGAACAA---TTTTC-GGCGCGGGTTGCGCCAAGGA------
CCATGAACGTTAGGCCTT--GCC-----TCTTTGC-GCAGTTAGGATGCGTTG-----AG-
GA-G--CC--CCATCATTGAA--AACTA-T-----------CTATTGAATGAC--
TCTCGACAAAGGATATCTCGGCTCTCGCATCGATGAAGAACG--TAGCGAAAT---
GCGATACTTGGTGTGAATTGCA-GAATCCTGTGAACCATTGA---
TTCTTTGAACGCAAGTT-
GCGCCTGAAGCCTTTTGGCCAAGGGCACGTCTGCCTGGGCGTCACGCATCGCGTCGCCCCTA
CC-T-CCCAACTCGTGTTTGG--ATATGTGT--G-GGGC-
GGAGAGTGGCCTCCCGTGTGCCT-CTT-GCTCACATGGATGGCCGAAATAAGGAGCTT---
GTGGCAG--CGAGCGCTGTGATGATTGGTGG-TTGGAT-GGGC-TCTT-GCCTTACAA---
AGCATC----TTGTCACGAAGCGCCG-CCCCGTGTT-G-GCTTGCAGGACTCGACCTGG--
TCGATGTTATCCCGATTTGTGTGC--C-TTTTATCGTCCAC-CAT
</sequence>
<sequence>
<taxon idref="wendORO10k"/>
TCGAAACCTGC-ATAGCAG-AGCAACATGCG--AACTTGTCTTTT-
CAACCTTCC--TTTCCGTGTGGCGGGTCGGACGA-CT------------CGTGCT-
TCCTC-CGCC-TTACG-GGATGAACAA---CTTTC-GGCGCGGGTTGCGCCAAGGA------
CCATGAACGTTAGGCCTC--GCC-----TCTTTGC-GCCGTTAGGCTGCGTTG-----AG-
GA-G--TC--ACATCATTGAA--AAATA-T-----------CAATTTAATGAC--
TCTTGACAAAGGATATCTCGGCTCTCGCATCGATGAAGAACG--TAGCGAAAT---
GCGATACTTGGTGTGAATTGCA-GAATCCTGTGAACCATTGA---
GTCTTTGAACGCAAGTT-
GCGCCCGAAGCCTTTTGGCCAAGGGCACGTCTGCCTGGGTGTCACGCATAGCGTCGCCCCTA
CC-T-CCCAACTCGTGTTTGG--ATATGTGT--G-GGGC-
GGAGAGTGGTCTCCCGTGTGC---CTT-GCCCACATGGATGGCCGAAATAAGGAGCTT---
GCGGCGG--TGAGCGCCGTGATGATTGGTGG-TTGGATGGGGC-TCTT-GCCCTACAA---
AGCATC----TTGTCATGAAGTGCCACCCCCGTGTT-G-GCTCGGAGGACTCGGTCTGG--
TCGATGTTATCCAGATTTGTGCGC--C-TTCTATTGTCCAC-CAT
</sequence>
<sequence>
<taxon idref="Serotinumo"/>
TCGAAACCTGC-ATAGCAG-AGCAACATGCG--AACTTGTCTTTT-
CAACCTTCC--TTTCCGTGTGGCGGGTTAGAAGA-CT------------CGTGCT-
TCCTC-CGCC-TTGCG-GGATGAACAA---TTTTC-GGCGCGGGTTGCGCCAAGGA------
CCATGAACGTTAGGCCTT--GCC-----TCTTTGC-GCAGTTAGGATGCGTTG-----AG-
GA-G--CC--CCATCATTGAA--AACTA-T-----------CTATTGAATGAC--
TCTCGACAAAGGATATCTCGGCTCTCGCATCGATGAAGAACG--TAGCGAAAT---
GCGATACTTGGTGTGAATTGCA-GAATCCTGTGAACCATTGA---
TTCTTTGAACGCAAGTT-
GCGCCTGAAGCCTTTTGGCCAAGGGCACGTCTGCCTGGGCGTCACGCATCGCGTCGCCCCTA
CC-T-CCCAACTCGTGTTTGG--ATATGTGT--G-GGGC-
GGAGAGTGGCCTCCCGTGTGCCT-CTT-GCTCACATGGATGGCCGAAATAAGGAGCTT---
GTGGCAG--CGAGCGCTGTGATGATTGGTGG-TTGGAT-GGGC-TCTT-GCCTTACAA---
AGCATC----TCGTCACGAAGCGCCG-CCCCGTGTT-G-GCTTGCAGGACTCGACCTGG--
TCGATGTTGTCCCGATTTGTGTGC--C-TTTTATCGTCCAC-CAT
</sequence>
<sequence>
<taxon idref="Curviflor1"/>
TCGAAACCTGC-ATAGCAG-AGCAACATGCG--AACTTGTCTTTT-
CAACCTTCCTTTTTCCGTGTGGCGGGTCAGACGA-CT------------CGTG----
CGTC-CGCC-TTGCG-GGATGAACAA---CTTTC-GGCGCGGGTTGCGCCAAGGA------
CCATGAACGTTAGGCCTC--GCC-----TCTTTGC-GCCGTTAGGCTGCGTTG-----AG-
GA-G--TC--CCATCATTGAA--AAATA-T-----------CAATTTAATGAC--
TCTTGACAAAGGATATCTCGGCTCTCGCATCGATGAAGAACG--TAGCGAAAT---
GCGATACTTGGTGTGAATTGCA-GAATCCTGTGAACCATTGA---

GTCTTTGAACGCAAGTT-
GCGCCCGAAGCCTTTTGGCCAAGGGCACGTCTGCCTGGGCGTCACGCATAGCGTCGCCCCTA
CC-T-CCCAACTCGTGTTTGG--ATGTGTGT--G-GGGC-
GGAGAGTGGTCTCCCGTGTGC---CTT-GCTCACATGGATGGCAGAAATAAGGAGCTT---
GCGGCGG--TGAGCGCCGTGATGATTGGTGG-TTGGAT-GGGC-TCTT-GCCCTACAA---
AGCATC----TTGTCATGAAGTGCCA-CCCCGTGTT-G-GCTCGGAGGACTCGGTCTGG--
TCGATGTTATCCAGATTTGTGCGC--C-TTTTATTGTCCAC-CAT
</sequence>
<sequence>
<taxon idref="Densiflor1"/>
TCGAAACCTGC-ATAGCAGAAGCAACATGCG--AACTTGTCTTCT-
CAACCTTCC--TTTCCGTGTGGCGGGTCAGAAGA-CT------------CGTGCT-
TCCTC-CGCC-TTGCG-GGATGAACAA---TTTTC-GGCGCGGGTTGCGCCAAGGA------
CCATGAACGTTAGGCGTT--GCC-----TCTTTGC-GCCGTTAGGCTGCGTTG-----AG-
GA-G--CC--CCATCATTGAA--AACTA-T-----------CTATTGAATGAC--
TCTCGACAAAGGATATCTCGGCTCTCGCATCGATGAAGAACG--TAGCGAAAT---
GCGATACTTGGTGTGAATTGCA-GAATCCTGTGAACCATTGA---
TTCTTTGAACGCAAGTT-
GCGCCTGAAGCCTTTTGGCCAAGGGCACGTCTGCCTGGGCGTCACGCATCGCGTCGCCCCTA
CC-T-CCCAACTCGTGTTTGG--ATATGTGT--G-GGGC-
GGAGAGTGGCCTCCCGTGTGCCT-CTT-GCTCACATGGATGGCCGAAATAAGGAGCTT---
GTGGCAG--CGAGCGCTGTGATGATTGGTGG-TTGGAT-GGGC-TTTTTGCCTTACAA---
AGCATC----TTGTCACGAAGCGCCG-CCCCGTGTT-G-GCTTGGAGGACTCGACCTGG--
TCGATGTTGTCCCGATTTGTGTGC--C-TTTTATCGTCCAC-CAT
</sequence>
<sequence>
<taxon idref="Hystrixxxx"/>
TCGAAACCTGC-ATAGCAG-AGCAACATGCG--AACTTGTCTTTT-
CAACCTTCC--TTTCCGTGTGGCGGGTCGGACGA-CT------------CGTGCT-
TCCTC-CGCC-TTACG-GGATGAACAA---CTTTC-GGCGCGGGTTGCGCCAAGGA------
CCATGAACGTTAGGCCTC--GCC-----TCTTTGC-GCCGTTAGGCTGCGTTG-----AG-
GA-G--TC--ACATCATTGAA--AAATA-T-----------CAATTTAATGAC--
TCTTGACAAAGGATATCTCGGCTCTCGCATCGATGAAGAACG--TAGCGAAAT---
GCGATACTTGGTGTGAATTGCA-GAATCCTGTGAACCATTGA---
GTCTTTGAACGCAAGTT-
GCGCCCGAAGCCTTTTGGCCAAGGGCACGTCTGCCTGGGTGTCACGCATAGCGTCGCCCCTA
CC-T-CCCAACTCGTGTTTGG--ATATGTGT--G-GGGC-
GGAGAGTGGTCTCCCGTGTGC---CTT-GCCCACATGGATGGCCGAAATAAGGAGCTT---
GCGGCGG--TGAGCGCCGTGATGATTGGTGG-TTGGATGGGGC-TCTT-GCCCTACAA---
AGCATC----TTGTCATGAAGTGCCACCCCCGTGTT-G-GCTCGGAGGACTCGGTCTGG--
TCGATGTTATCCAGATTTGTGCGC--C-TTCTATTGTCCAC-CAT
</sequence>
<sequence>
<taxon idref="Karelinii1"/>
TCGAAACCTGC-ATAGCAG-AGCAACATGCG--AACTTGTCTTTT-
CAACCTTCC--TTTCCGTGTGGCGGGTCGGACGA-CT------------CGTGCT-
TCCTC-CGCC-TTACG-GGATGAACAA---CTTTC-GGCGCGGGTTGCGCCAAGGA------
CCATGAACGTTAGGCCTC--GCC-----TCTTTGC-GCCGTTAGGCTGCGTTG-----AG-
GA-G--TC--ACATCATTGAA--AAATA-T-----------CAATTTAATGAC--
TCTTGACAAAGGATATCTCGGCTCTCGCATCGATGAAGAACG--TAGCGAAAT---
GCGATACTTGGTGTGAATTGCA-GAATCCTGTGAACCATTGA---
GTCTTTGAACGCAAGTT-
GCGCCCGAAGCCTTTTGGCCAAGGGCACGTCTGCCTGGGTGTCACGCATAGCGTCGCCCCTA
CC-T-CCCAAATCGTGTTTGG--ATATGTGT--G-GGGC-
GGAGAGTGGTCTCCCGTGTGC---CTT-GCCCACATGGATGGCCGAAATAAGGAGCTT---
GCGGCGG--TGAGCGCCGTGATGATTGGTGGGTTGGATGGGGC-TCTT-GCCCTACAA---

AGCATC----TTGTCATGAAGTGCCACCCCCGTGTT-G-GCTCGGAGGACTCGGTCTGG--
TCGATGTTATCCAGATTTGTGCGC--C-TTCTATTGTCCAC-CAT
</sequence>
<sequence>
<taxon idref="Rudbaricu1"/>
TCGAAACCTGC-ATAGCAG-AGCAACATGCG--AACTTGTCTTTT-
CAACCTTCC--TTTCCGTGTGGCGGGTCGGACGA-CT------------CGTGCT-
TCCTC-CGCC-TTACG-GGATGAACAA---CTTTC-GGCGCGGGTTGCGCCAAGGA------
CCATGAACGTTAGGCCTC--GCC-----TCTTTGC-GCCGTTAGGCTGCGTTG-----AG-
GA-G--TC--ACATCATTGAA--AAATA-T-----------CAATTTAATGAC--
TCTTGACAAAGGATATCTCGGCTCTCGCATCGATGAAGAACG--TAGCGAAAT---
GCGATACTTGGTGTGAATTGCA-GAATCCTGTGAACCATTGA---
GTCTTTGAACGCAAGTT-
GCGCCCGAAGCCTTTTGGCCAAGGGCACGTCTGCCTGGGTGTCACGCATAGCGTCGCCCCTA
CC-T-CCCAACTCGTGTTTGG--ATATGTGT--G-GGGC-
GGAGAGTGGTCTCCCGTGTGC---CTT-GCCCACATGGATGGCCGAAATAAGGAGCTT---
GCGGCGG--TGAGCGCCGTGATGATTGGTGG-TTGGATGGGGC-TCTT-GCCCTACAA---
AGCATC----TTGTCATGAAGTGCCACCCCCGTGTT-G-GCTCGGAGGACTCGGTCTGG--
TCGATGTTATCCAGATTTGTGCGC--C-TTCTATTGTCCAC-CAT
</sequence>
<sequence>
<taxon idref="Mirtajadin"/>
TCGAAACCTGC-ATAGCAG-AGCAACATGCG--AACTTGTCTTTT-
CAACCTTCCTTTTTCCGTGTGGCGGGTCAGACGA-CT------------CGTGCT-
TCCTC-CGCC-TTGCG-GGATGAACAA---CTTTC-GGCGCGGGTTGCGCCAAGGA------
CCATGAACGTTAGGCCTC--GCC-----TCTTTGC-GCCGTTAGGCTGCGTTG-----GG-
GA-G--TC--CCATCATTGAA--AAATA-T-----------CAATTTAATGAC--
TCTTGACAAAGGATATCTCGGCTCTCGCATCGATGAAGAACG--TAGCGAAAT---
GCGATACTTGGTGTGAATTGCA-GAATCCTGTGAACCATTGA---
GTCTTTGAACGCAAGTT-
GCGCCCGAAGCCTTTTGGCCAAGGGCACGTCTGCCTGGGCGTCACGCATAGCGTCGCCCCTA
CC-T-CCCAACTCGTGTTTGG--ATGTGTGT--G-GGGC-
GGAGAGTGGTCTCCCGTGTGC---CTT-GCTCACATGGATGGCAGAAATAAGGAGCTT---
GCGGCGG--TGAGCGCCGTGATGATTGGTGG-TTGGAT-GGGC-TCTT-GCCCTACGA---
AGCATC----TTGTCATGAAGTGCCA-CCYCGTGTT-G-GCTCGGAGGACTCTGTCTGG--
TCGATGTTATCCAGATTTGTGCGC--C-TTTTATTGTCCAC-CAT
</sequence>
<sequence>
<taxon idref="Sacberllum"/>
TCGAAACCTGC-ATAGCAG-AGCAACATGCG--AACTTGTCTTTT-
CAACCTTCC--TTTCCGTGTGGCGGGTYAGAAGA-CT------------CGTGCT-
TCCTC-CGCC-TTGCG-GGATGAACAA---TTTTC-GGCGCGGGTTGCGCCAAGGA------
CCATGAACGTTAGGCCTT--GCC-----TCTTTGC-GCMGTTAGGMTGCGTTG-----AG-
GA-G--CC--CCATCATTGAA--AACTA-T-----------CTATTGAATGAC--
TCTCGACAAAGGATATCTCGGCTCTCGCATCGATGAAGAACG--TAGCGAAAT---
GCGATACTTGGTGTGAATTGCA-GAATCCTGTGAACCATTGA---
TTCTTTGAACGCAAGTT-
GCGCCTGAAGCCTTTTGGCCAAGGGCACGTCTGCCTGGGCGTCACGCATCGCGTCGCCCCTA
CC-T-CCCAACTCGTGTTTGG--ATATGTGT--G-GGGC-
GGAGAGTGGCCTCCCGTGTGCCT-CTT-GCTCACATGGATGGCCGAAATAAGGAGCTT---
GYGGCAG--CGAGCGCTGTGATGATTGGTGG-TTGGAT-GGGC-TYTT-GCCTTACAA---
AGCATC----TTGTCACGAAGCGCCG-CCCCGTGTT-G-GCTTGSAGGACTCGACCTGG--
TCGATGTTATCCCGATTTGTGTGC--C-TTTTATCGTCCAC-CAT
</sequence>
<sequence>
<taxon idref="Shirazianu"/>

TCGAAACCTGC-ATAGCAG-AGCAACATGCG--AACTTGTCTTTT-
CAACCTTCCTTTTTCCGTGTGGCGGGTCAGACGA-CT------------CGTGCT-
TCCTC-CGCC-TTGCG-GGATGAACAA---CTTTC-GGCGCGGGTTGCGCCAAGGA------
CCATGAACGTTAGGCCTC--GCC-----TCTTTGC-GCCGTTAGGCTGCGTTG-----AG-
GA-G--TC--CCATCATTGAA--AAATA-T-----------CAATTTAATGAC--
TCTTGACAAAGGATATCTCGGCTCTCGCATCGATGAAGAACG--TAGCGAAAT---
GCGATACTTGGTGTGAATTGCA-GAATCCTGTGAACCATTGA---
GTCTTTGAACGCAAGTT-
GCGCCCGAAGCCTTTTGGCCAAGGGCACGTCTGCCTGGGCGTCACGCATAGCGTCGCCCCTA
CC-T-CCCAACTCGTGTTTGG--ATGTGTGT--G-GGGC-
GGAGAGTGGTCTCCCGTGTGC---CTT-GCTCACATGGATGGCAGAAATAAGGAGCTT---
GCGGCGG--TGAGCGCCGTGATGATTGGTGG-TTGGAT-GGGC-TCTT-GCCCTACAA---
AGCATC----TTGTCATGAAGTGCCA-CCCCGTGTT-G-GCTCGGAGGACTCGGTCTGG--
TCGATGTTATCCAGATTTGTGCGC--C-TTTTATTGTCCAC-CAT
</sequence>
<sequence>
<taxon idref="Hormozgane"/>
TCGAAACCTGC-ATAGCAG-AGCAACATGCG--AACTTGTCTTTT-
CAACCTTCC--TTTCCGTGTGGCGGGTTAGAAGA-CT------------CGTGCT-
TCCTC-CGCC-TTGCG-GGATGAACAA---TTTTC-GGCGCGGGTTGCGCCAAGGA------
CCATGAACGTTAGGCCTT--GCC-----TCTTTGC-GCAGTTAGGATGCGTTG-----AG-
GA-G--CC--CCATCATTGAA--AACTA-T-----------CTATTGAATGAC--
TCTCGACAAAGGATATCTCGGCTCTCGCATCGATGAAGAACG--TAGCGAAAT---
GCGATACTTGGTGTGAATTGCA-GAATCCTGTGAACCATTGA---
TTCTTTGAACGCAAGTT-
GCGCCTGAAGCCTTTTGGCCAAGGGCACGTCTGCCTGGGCGTCACGCATCGCGTCGCCCCTA
CC-T-CCCAACTCGTGTTTGG--ATATGTGT--G-GGGC-
GGAGAGTGGCCTCCCGTGTGCCT-CTT-GCTCACATGGATGGCCGAAATAAGGAGCTT---
GTGGCAG--CGAGCGCTGTGATGATTGGTGG-TTGGAT-GGGC-TCTT-GCCTTACAA---
AGCATC----TCGTCACGAAGCGCCG-CCCCGTGTT-G-GCTTGCAGGACTCGACCTGG--
TCGATGTTATCCCGATTTGTGTGC--C-TTTTATCGTCCAC-CAT
</sequence>
<sequence>
<taxon idref="Glabratum1"/>
TCGAAACCTGC-ATAGCAG-AGCAACATGCG--AACTTGTCTTTT-
CAACCTTCCTTTTTCCGTGTGGCGGGTCAGAGGG-CT------------CGTGCT-
TCCTC-CGCC-TTGCG-GGATGAACAA---CTTTC-GGCGCGGGTTGCGCCAAGGA------
CCATGAACGTTAGGCCTC--GCC-----TCTTTGC-GCCGTTAGGCTGCGTTG-----AG-
GA-G--TC--CCATCATTGAA--AAATA-T-----------CAATTTAATGAC--
TCTTGACAAAGGATATCTCGGCTCTCGCATCGATGAAGAACG--TAGCGAAAT---
GCGATACTTGGTGTGAATTGCA-GAATCCTGTGAACCATTGA---
GTCTTTGAACGCAAGTT-
GCGCCCGAAGCCTTTTGGCCAAGGGCACGTCTGCCTGGGCGTCACGCATAGCGTCGCCCCTA
CC-T-CCCAACTCGTGTTTGG--ATGTGTGT--G-GGGC-
GGAGAGTGGTCTCCCGTGTGC---CTT-GCTCACATGGATGGCAGAAATAAGGAGCTT---
GCGGCGG--TGAGCGCCGTGATGATTGGTGG-TTGGAT-GGGC-TCTT-GCCCTACAA---
AGCATC----TTGTCATGAAGTGCCA-CCCCGTGTT-G-GCTCGGAGGACTCGGTCTGG--
TCGATGTTATCCAGATTTGTGCGC--C-TTTTATTGTCCAC-CAT
</sequence>
<sequence>
<taxon idref="Moradiiiii"/>
TCGAAACCTGC-ATAGCAG-AGCAACATGCG--AACTTGTCTTTT-
CAACCTTCC--TTTCCGTGTGGCGGGTTAGAAGA-CT------------CGTGCT-
TCCTC-CGCC-TTGCG-GGATGAACAA---TTTTC-GGCGCGGGTTGCGCCAAGGA------
CCATGAACGTTAGGCCTT--GCC-----TCTTTGC-GCAGTTAGGATGCGTTG-----AG-
GA-G--CC--CCATCATTGAA--AACTA-T-----------CTATTGAATGAC--

TCTCGACAAAGGATATCTCGGCTCTCGCATCGATGAAGAACG--TAGCGAAAT---
GCGATACTTGGTGTGAATTGCA-GAATCCTGTGAACCATTGA---
TTCTTTGAACGCAAGTT-
GCGCCTGAAGCCTTTTGGCCAAGGGCACGTCTGCCTGGGCGTCACGCATCGCGTCGCCCCTA
CC-T-CCCAACTCGTGTTTGG--ATATGTGT--G-GGGC-
GGAGAGTGGCCTCCCGTGTGCCT-CTT-GCTCACATGGATGGCCGAAATAAGGAGCTT---
GTGGCAG--CGAGCGCTGTGATGATTGGTGG-TTGGAT-GGGC-TCTT-GCCTTACAA---
AGCATC----TTGTCACGAAGCGCCG-CCCCGTGTT-G-GCTTGCAGGACTCGACCTGG--
TCGATGTTATCCCGATTTGTGTGC--C-TTTTATCGTCCAC-CAT
</sequence>
<sequence>
<taxon idref="Tomentellu"/>
TCGAAACCTGC-ATAGCAG-AGCAACATGCG--AACTTGTCTTTT-
CAACCTTCCTTTTTCCGTGTGGCGGGTCAGACGA-CT------------CGTGCT-
TCCTC-CGCC-TTGCG-GGATGAACAA---CTTTC-GGCGCGGGTTGCGCCAGGGA------
CCATGAACGTTAGGCCTC--GCC-----TCTTTGC-GCCGTTGGGCTGCGTTG-----AG-
GA-G--TC--CCATCATTGAA--AAATA-T-----------CAATTTAATGAC--
TCTTGACAAARGATATCTCGGCTCTCGCATCGATGAAGAACG--TAGCGAAAT---
GCGATACTTGGTGTGAATTGCA-GAATCCTGTGAACCATTGA---
GTCTTTGAACGCAAGTT-
GCGCCCGAAGCCTTTTGGCCAGGGGCACGTCTGCCTGGGCGTCACGCATAGCGTCGCCCCTA
CC-T-CCCAACTCGTGTTTGG--ATGTGTGT--G-GGGC-
GGAGAGTGGTCTCCCGTGTGC---CTT-GCTCACATGGATGGCAGAAATAAGGAGCTT---
GCGGCGG--TGAGCGCCGTGATGATTGGTGG-TTGGAT-GGGC-TCTT-GCCCTACAA---
AGCATC----TTGTCRTGAAGTGCCA-CCCCGTGTT-G-GCTCGGAGGACTCTGTCTGG--
TCGATGTTATCCAGATTTGTGCGC--C-TTTTATTGTCCAC-CAT
</sequence>
<sequence>
<taxon idref="Hohenacer1"/>
TCGAAACCTGC-ATAGCAG-AGCAACATGCG--AACTTGTCTTTT-
CAACCTTCC--TTTCCGTGTGGCGGGTCAGAAGA-CT------------CGTGCT-
TCCTC-CGCC-TTGCG-GGATGAACAA---TTTTC-GGCGCGGGTTGCGCCAAGGA------
CCATGAACGTTAGGMCTT--GCC-----TCTTTGC-GCCGTTAGGCTGCGTTG-----AG-
GA-G--CC--CCATCATTGAA--AACTA-T-----------CTATTGAATGAC--
TCTCGACAAAGGATATCTCGGCTCTCGCATCGATGAAGAACG--TAGCGAAAT---
GCGATACTTGGTGTGAATTGCA-GAATCCTGTGAACCATTGA---
TTCTTTGAACGCAAGTT-
GCGCCTGAAGCCTTTTGGCCAAGGGCACGTCTGCCTGGGCGTCACGCATCGCGTCGCCCCTA
CC-T-CCCAACTCGTGTTTGG--ATATGTGT--G-GGGC-
GGAGAGTGGCCTCCCGTGTGCCT-CTT-GCTCACATGGATGGCCGAAATAAGGAGCTT---
GTGGCAG--CGAGCGCTGTGATGATTGGTGG-TTGGAT-GGGC-TTTT-GCCTTACAA---
AGCATC----TTGTCACGAAGCGCCG-CCCCGTGTT-G-GCTTGGAGGACTCGACCTGG--
TCGATGTTATCCCGATTTGTGTGC--C-TTTTATCGTCCAC-CAT
</sequence>
<sequence>
<taxon idref="Acanthob40"/>
TCGAAACCTGC-ATAGCAG-AGCAACATGCG--AACTTGTCTTTT-
CAACCTTCCTTTTCCCGTGTGGCGGGTCAGACGA-CT------------CGTGCT-
TCCTC-CGCC-TTGCG-GGATGAACAA---CTTTC-GGCGCGGGTTGCGCCAAGGA------
CCATGAACGTTAGGCCTC--GCC-----TCTTTGC-GCCGTTAGGCTGCGTTG-----AG-
GA-G--TC--CCATCATTGAA--AAATA-T-----------CAATTTAATGAC--
TCTTGACAAAGGATATCTCGGCTCTCGCATCGATGAAGAACG--TAGCGAAAT---
GCGATACTTGGTGTGAATTGCA-GAATCCTGTGAACCATTGA---
NTCTTTGAACGCAAGTT-
GCGCCCGAAGCCTTTTGGCCAAGGGCACGTCTGCCTGGGCGTCACGCATAGCGTCGCCCCTA
CC-T-CCCAACTCGTGTTTGG--ATGTGTGT--G-GGGC-

GGAGAGTGGTCTCCCGTGTGC---CTT-GCTCACATGGATGGCCGAAATAAGGAGCTT---
GCGGCGG--TGAGCGCCGTGATGATTGGTGG-TTGGAT-GGGC-TCTT-GCCCTACAR---
AGCATC----TTGTCATGAAGTGCCA-CCCCGTGTTTG-GCTCGGAGGACTCGGTCTGG--
TCGATGTTATCCAGATTTGTGCGC--C-TTTTATTGTCCAC-CAT
</sequence>
<sequence>
<taxon idref="ahangare92"/>
TCGAAACCTGC-ATAGCAG-AGCAACATGCG--AACTTGTCTTTT-
CAACCTTCC--TTTCCGTGTGGCGGGTCAGACGA-CT------------CGTGCT-
TCCTC-CGCC-TTGCG-GGATGAACAA---CTTTC-GGCGCGGGTTGCGCCAAGGA------
CCATGAACGTTAGGCCTC--GCC-----TCTTTGC-GCCGTTAGGCTGCGTTG-----AG-
GA-G--TC--CCATCATTGAA--AAATA-T-----------CAATTTAATGAC--
TCTTGACAAAGGATATCTCGGCTCTCGCATCGATGAAGAACG--TAGCGAAAT---
GCGATACTTGGTGTGAATTGCA-GAATCCTGTGAACCATTGA---
NTCTTTGAACGCAAGTT-
GCGCCCGAAGCCTTTTGGCCAAGGGCACGTCTGCCTGGGCGTCACGCATAGCGTCGCCCCTA
CC-T-CCCAACTCGTGTTTGG--ATATGTGT--G-GGGC-
GGAGAGTGGTCTCCCGTGTGC---CTT-GCTCACATGGATGGCCGAAATAAGGAGCTT---
GCGGCGG--TGAGCGCCGTGATGATTGGTGG-TTGGAT-GGGC-TCTT-GCCCTACAA---
AGCATC----TTGTCATGAAGTGCCA-CCCCGTGTT-G-GCTCGGAGGACTCGGTCTGG--
TCGATGTTATCCAGATTTGTGCGC--C-TTTTATTGTCCAC-CAT
</sequence>
<sequence>
<taxon idref="AuganumW86"/>
TCGAAACCTGC-ATAGCAG-AGCAACATGCG--AACTTGTCTTTC-
CAACCTTCCTTTTTCCGTGTGGCGGGTCAGACGA-CT------------CGTGCT-
TCCTC-CGCC-TTGCG-GGATGAACAA---CTTTC-GGCGCGGGTTGCGCCAAGGA------
CCATGAACGTTAGGCCTC--GCC-----TCTTTGC-GCCGTTAGGCTGCGTTG-----AG-
GA-G--TC--CCATCATTGAA--AAATA-T-----------CAATTTAATGAC--
TCTTGACAAAGGATATCTCGGCTCTCGCATCGATGAAGAACG--TAGCGAAAT---
GCGATACTTGGTGTGAATTGCA-GAATCCTGTGAACCATTGA---
NTCTTTGAACGCAAGTT-
GCGCCCGAAGCCTTTTGGCCAAGGGCACGTCTGCCTGGGCGTCACGCATAGCGTCGCCCCTA
CC-T-CCCAACTCGTGTTTGG--ATGTGTGT--G-GGGC-
GGAGAGTGGTCTCCCGTGTGC---CTT-GCTCACATGGATGGCCGAAATAAGGAGCTT---
GCGGCGG--TGAGCGCCGTGATGATTGGTGG-TTGGAT-GGGC-TCTT-GCCCTACAA---
AGCATC----TTGTCATGAAGTGCCA-CCCCGTGTTTG-GCTCGGAGGACTCGGTCTGG--
TCGATGTTATCCAGATTTGTGCGC--C-TTTTATTGTCCAC-CAT
</sequence>
<sequence>
<taxon idref="Cabulicu99"/>
TCGAAACCTGC-ATAGCAG-AGCAACATGCG--AACTTGTCTTTT-
CAACCTTCCTTTTTCCGTGTGGCGGGTCAGACGA-CT------------CGTGCT-
TCCTC-CGCC-TTGCG-GGATGAACAA---CTTTC-GGCGCGGGTTGCGCCAAGGA------
CCATGAACGTTAGGCCTC--GCC-----TCTTTGC-GCCGTTAGGCTGCGTTG-----AG-
GA-G--TC--CCATCATTGAA--AAATA-T-----------CAATTTAATGAC--
TCTTGACAAAGGATATCTCGGCTCTCGCATCGATGAAGAACG--TAGCGAAAT---
GCGATACTTGGTGTGAATTGCA-GAATCCTGTGAACCATTGA---
NTCTTTGAACGCAAGTT-
GCGGCCGAAGCCTTTTGGCCAAGGGCACGTCTGCCTGGGCGTCACGCATAGCGTCGCCCCTA
CC-T-CCCAACTCGTGTTTGG--ATGTGTGT--G-GGGC-
GGAGAGTGGTCTCCCGTGTGC---CTT-GCTCACATGGATGGCCGAAATAAGGAGCTT---
GCGGCGG--TGAGCGCCGTGATGATTGGTGG-TTGGAT-GGGC-TCTT-GCCCTACAA---
AGCATC----TTGTCATGAAGTGCCA-CCCCGTGTTTG-GCTCGGAGGACTCGGTCTGG--
TCGATGTTATCCAGATTTGTGCGC--C-TTTTATTGTCCAC-CAT
</sequence>

<sequence>
<taxon idref="Carinatu25"/>
TCGAAACCTGC-ATAGCAG-AGCAACATGCG--AACTTGTCTTTT-
CAACCTTCC--TTTCCGTGTGGCGGGTCAGACGA-CT-------------CGTGCT-
TCCTC-CGCC-TTGCG-GGATGAACAA---CTTTC-GGCGCGGGTTGCGCCAAGGA------
CCATGAACGTTAGGCCTC--GCC-----TCTTTGC-GCCGTTAGGCTGCGTTG-----AG-
GA-G--TC--CCATCATTGAA--AAATA-T------------CAATTTAATGAC--
TCTTGACAAAGGATATCTCGGCTCTCGCATCGATGAAGAACG--TAGCGAAAT---
GCGATACTTGGTGTGAATTGCA-GAATCCTGTGAACCATTGA---
NTCTTTGAACGCAAGTT-
GCGGCCGAAGCCTTTTGGCCAAGGGCACGTCTGCCTGGGCGTCACGCATAGCGTCGCCCCTA
CC-T-CCCAACTCGTGTTTGG--ATATGTGT--G-GGGC-
GGAGAGTGGTCTCCCGTGTGC---CTT-GCTCACATGGATGGCCGAAATAAGGAGCTT---
GCGGCGG--TGAGCGCCGTGATGATTGGTGG-TTGGAT-GGGC-TCTT-GCCCTACAA---
AGCATC----TTGTCATGAAGTGCCA-CCCCGTGTT-G-GCTCGGAGGACTCGGTCTGG--
TCGATGTTATCCAGATTTGTGCGC--C-TTTTATTGTCCAC-CAT
</sequence>
<sequence>
<taxon idref="Cephalot39"/>
TCGAAACCTGC-ATAGCAG-AGCAACATGCG--AACTTGTCTTTT-
CAACCTTCC--TTTCCGTGTGGCGGGTCAGAAGA-CT-------------CGTGCT-
TCCTC-CGCC-TTGCG-GGATGAACAA---TTTCC-GGCGCGGGTTGCGCCAAGGA------
CCATGAACGTTAGGCCTT--GCC-----TCTTTGC-GCCGTTAGGCTGCGTTG-----AG-
GA-G--CC--CCACCATTGAA--AACTA-T------------CAATTGAATGAC--
TCTCGACAAAGGATATCTCGGCTCTCGCATCGATGAAGAACG--TAGCGAAAT---
GCGATACTTGGTGTGAATTGCA-GAATCCTGTGAACCATTGA---
TTCTTTGAACGCAAGTT-
GCGCCTGAAGCCTTTTGGCCAAGGGCACGTCTGCCTGGGCGTCACGCATCGCGTCGCCCCTA
CC-T-CCCAACTCGTGTTTGG--ATATGTGT--A-GGGC-
GGAGAGTGGCCTCCCGTGTGGCT-CTT-GCTCACATGGATGGCCGAAATAAGGAGCTT---
GTGGCAG--CGAGCGCTGTGATGATTGGTGG-TTGGAC-GGGC-TCTT-GCCTTACAA---
AGCATC----TTGTCACGAAGCGCCG-CCCCGTGTT-G-GCTTGGAGGACTCGACCTGG--
TCGATGTTATCCCGATTTGTGTGC--C-TTTTATCGTCCAC-CAT
</sequence>
<sequence>
<taxon idref="erinaceu70"/>
TCGAAACCTGC-ATAGCAG-AGCAACATGCG--AACTTGTCTTTT-
CAACCTTCC--TTTCCGTGTGGCGGGTCAGACGA-CT-------------CGTGCT-
TCCTC-CGCC-TTGCG-GGATGAACAA---CTTTC-GGCGCGGGTTGCGCCAAGGA------
CCATGAACGTTAGGCCTC--GCC-----TCTTTGC-GCCGTTAGGCTGCGTTG-----AG-
GA-G--TC--CCATCATTGAA--AAATA-T------------CAATTTAATGAC--
TCTTGACAAAGGATATCTCGGCTCTCGCATCGATGAAGAACG--TAGCGAAAT---
GCGATACTTGGTGTGAATTGCA-GAATCCTGTGAACCATTGA---
GTCTTTGAACGCAAGTT-
GCGCCCGAAGCCTTTTGGCCAAGGGCACGTCTGCCTGGGCGTCACGCATAGCGTCGCCCCTA
CC-T-CCCAACTCGTGTTTGG--ATATGTGT--G-GGGC-
GGAGAGTGGTCTCCCGTGTGC---CTT-GCTCACATGGATGGCCGAAATAAGGAGCTT---
GCGGCGG--TGAGCGCCGTGATGATTGGTGG-TTGGAT-GGGC-TCTT-GCCCTACAA---
AGCATC----TTGTCATGAAGTGCCA-CCCCGTGTT-G-GCTCGGAGGACTCGGTCTGG--
TCGATGTTATCCAGATTTGTGCGC--C-TTTTATTGTCCAC-CAT
</sequence>
<sequence>
<taxon idref="fascicul45"/>
TCGAAACCTGC-ATAGCAG-AGCAACATGCG--AACTTGTCTTTT-
CAACCTTCCTTTTTCCGTGTGGCGGGTCAGACGA-CT-------------CGTGCT-
TCCTC-CGCC-TTGCG-GGATGAACAA---CTTTC-GGCGCGGGTTGCGCCAAGGA------

CCATGAACGTTAGGCCTC--GCC-----TCTTTGC-GCCGTTAGGCTGCGTTG-----AG-
GA-G--TC--CCATCATTGAA--AAATA-T-----------CAATTTAATGAC--
TCTTGACAAAGGATATCTCGGCTCTCGCATCGATGAAGAACG--TAGCGAAAT---
GCGATACTTGGTGTGAATTGCA-GAATCCTGTGAACCATTGA---
NTCTTTGAACGCAAGTT-
GCGCCCGAAGCCTTTTGGCCAAGGGCACGTCTGCCTGGGCGTCACGCATAGCGTCGCCCCTA
CC-T-CCCAACTCGTGTTTGG--ATGTGTGT--G-GGGC-
GGAGAGTGGYCTCCCGTGTGC---CTT-GCTCACATGGATGGCCGAAATAAGGAGCTT---
GCGGCGG--TGAGCGCCGTGATGATTGGTGG-TTGGAT-GGGC-TCTT-GCCCTACAA---
AGCATC----TTGTCATGAAGTGCCA-CCCCGTGTTTG-GCTCGGAGGACTCGGTCTGG--
TCGATGTTATCCAGATTTGTGCGC--C-TTTTATTGTCCAC-CAT
</sequence>
<sequence>
<taxon idref="Festucas88"/>
TCGAAACCTGC-ATAGCAG-AGCAACATGCG--AACTTGTCTTTT-
CAACCTTCC--TTTCCGTGTGGCGGGTTAGAAGA-CT------------CGTGCT-
TCCTC-CGCC-TTGCG-GGATGAACAA---TTTTC-GGCGCGGGTTGCGCCAAGGA------
CCATGAACGTTAGGCCTT--GCC-----TCTTTGC-GCAGTTAGGATGCGTTG-----AG-
GA-G--CC--CCATCATTGAA--AACTA-T-----------CTATTGAATGAC--
TCTCGACAAAGGATATCTCGGCTCTCGCATCGATGAAGAACG--TAGCGAAAT---
GCGATACTTGGTGTGAATTGCA-GAATCCTGTGAACCATTGA---
TTCTTTGAACGCAAGTT-
GCGCCTGAAGCCTTTTGGCCAAGGGCACGTCTGCCTGGGCGTCACGCATCGCGTCGCCCCTA
CC-T-CCCAACTCGTGTTTGG--ATATGTGT--G-GGGC-
GGAGAGTGGCCTCCCGTGTGCCT-CTT-GCTCACATGGATGGCCGAAATAAGGAGCTT---
GTGGCAG--CGAGCGCTGTGATGATTGGTGG-TTGGAT-GGGC-TCTT-GCCTTACAA---
AGCATC----TTGTCACGAAGCGCCG-CCCCGTGTT-G-GCTTGCAGGACTCGACCTGG--
TCGATGTTATCCCGATTTGTGTGC--C-TTTTATCGTCCAC-CAT
</sequence>
<sequence>
<taxon idref="Chitrali79"/>
TCGAAACCTGC-ATAGCAG-AGCAACATGCG--AACTTGTCTTTT-
CAACCTTCTATTTTCCGTGTGGCGGGTCAGACGA-CT------------CGTGCT-
TCCTC-CGCC-TTGCG-GGATGAACAA---CTTTC-GGCGCGGGTTGCGCCAAGGA------
CCATGAACGTTAGGCCTC--GCC-----TCTTTGC-GCCGTTAGGCTTCGTTG-----AG-
GA-G--TC--CCATCATTGAA--AAATA-T-----------CAATTTAATGAC--
TCTTGACAAAGGATATCTCGGCTCTCGCATCGATGAAGAACG--TAGCGAAAT---
GCGATACTTGGTGTGAATTGCA-GAATCCTGTGAACCATTGA---
NTCTTTGAACGCAAGTT-
GCGCCCGAAGCCTTTTGGCCAAGGGCACGTCTGCCTGGGCGTCACGCATAGCGTCGCCCCTA
CC-T-CCCAACTCGTGTTTGG--ATGTGTGT--G-GGGC-
GGAGAGTGGTCTCCCGTGTGC---CTT-GCTCACATGGATGGCCGAAATAAGGAGCTT---
GCGGCGG--TGAGCGCCGTGATGATTGGTGG-TTGGAT-GGGC-TCTT-GCCCTACAA---
AGCATC----TTGTCATGAAGTGCCA-CCCCGTGTTTG-GCTCGGAGGACTCGGTCTGG--
TCGATGTTATCCAGATTTGTGCGC--C-TTTTATTGTCCAC-CAT
</sequence>
<sequence>
<taxon idref="gilliiW141"/>
TCGAAACCTGC-ATAGCAG-AGCAACATGCG--AACTTGTCTTTT-
CAACCTTCTATTTTCCGTGTGGCGGGTCAGACGA-CT------------CGTGCT-
TCCTC-CGCC-TTGCG-GGATGAACAA---CTTTC-GGCGCGGGTTGCGCCAAGGA------
CCATGAACGTTAGGCCTC--GCC-----TCTTTGC-GCCGTTAGGCTGCGTTG-----AG-
GA-G--TC--CCATCATTGAA--AAATA-T-----------CAATTTAATGAC--
TCTTGACAAAGGATATCTCGGCTCTCGCATCGATGAAGAACG--TAGCGAAAT---
GCGATACTTGGTGTGAATTGCA-GAATCCTGTGAACCATTGA---
NTCTTTGAACGCAAGTT-

GCGCCCGAAGCCTTTTGGCCAAGGGCACGTCTGCCTGGGCGTCACGCATAGCGTCGCCCCTA
CC-T-CCCAACTCGTGTTTGG--ATGTGTGT--G-GGGC-
GGAGAGTGGTCTCCCGTGTGC---CTT-GCTCACATGGATGGCCGAAATAAGGAGCTT---
GCGGCGG--TGAGCGCCGTGATGATTGGTGG-TTGGAT-GGGC-TCTT-GCCCTACAA---
AGCATC----TTGTCATGAAGTGCCA-CCCCGTGTTTG-GCTCGGAGGACTCGGTCTGG--
TCGATGTTATCCAGATTTGTGCGC--C-TTTTATTGTCCAC-CAT
</sequence>
<sequence>
<taxon idref="inermeM004"/>
TCGAAACCTGC-ATAGCAG-AGCAACATGCG--AACTTGTCTTTT-
CAACCTTCC--TTTCCGTGTGGCGGGTTAGACGA-CT------------CGTGCT-
TCCTC-CGCC-TTGCG-GGATGAACAA---CTTTC-GGCGCGGGTTGCGCCAAGGA------
CCATGAACGTTAGGCCTT--GCC-----TCTTTGC-GCCGTTAGGCTGCGTTG-----AG-
GA-G--TC--CCATCACTGAA--AATTA-T-----------CAATTTAATGAC--
TCTTGACAAAGGATATCTCGGCTCTCGCATCGATGAAGAACG--TAGCGAAAT---
GCGATACTTGGTGTGAATTGCA-GAATCCTGTGAACCATTGA---
NTCTTTGAACGCAAGTT-
GCGCCCGAAGCCTTTTGGCCAAGGGCACGTCTGCCTGGGCGTCACGCATAGCGTCGCCCCTA
CC-T-CCCAACTCGTGTTTGG--ATATGTGT--G-GGGC-
GGAGAGTGGTCTCCCGTGTGC---CTA-GCTCACATGGATGGCCGAAATAAGGAGCTT---
GCGGCGG--TGAGCGCCGTGATGATTGGTGG-TTGGAT-GGGC-TCTT-GCCCTACAA---
AGCATC----TTGTCATGAAGTGCCA-CCCCGTGTT-G-GCTCGGAGGACTCGGTCTGG--
TCGATGTTATCCAGATTTGTGTGC--C-TTTTATTGTCCAC-CAT
</sequence>
<sequence>
<taxon idref="koeieiW153"/>
TCGAAACCTGC-ATAGCAG-AGCAACATGCG--AACTTGTCTTTT-
CAACCTTCCTTTTCCCGTGTGGCGGGTCAGACGA-CT------------CGTGCT-
TCCTC-CGCC-TTGCG-GGATGAACAA---CTTTC-GGCGCGGGTTGCGCCAAGGA------
CCATGAACGTTAGGCCTC--GCC-----TCTTTGC-GCCGTTAGGCTGCGTTG-----AG-
GA-G--TC--CCATCATTGAA--AAATA-T-----------CAATTTAATGAC--
TCTTGACAAAGGATATCTCGGCTCTCGCATCGATGAAGAACG--TAGCGAAAT---
GCGATACTTGGTGTGAATTGCA-GAATCCTGTGAACCATTGA---
NTCTTTGAACGCAAGTT-
GCGCCCGAAGCCTTTTGGCCAAGGGCACGTCTGCCTGGGCGTCACGCATAGCGTCGCCCCTA
CC-T-CCCAACTCGTGTTTGG--ATGTGTGT--G-GGGC-
GGAGAGTGGTCTCCCGTGTGC---CTT-GCTCACATGGATGGCCGAAATAAGGAGCTT---
GCGGCGG--TGAGCGCCGTGATGATTGGTGG-TTGGAT-GGGC-TCTT-GCCCTACAA---
AGCATC----TTGTCATGAAGTGCCA-CCCCGTGTTTG-GCTCGGAGGACTCGGTCTGG--
TCGATGTTATCCAGATTTGTGCGC--C-TTTTATTGTCCAC-CAT
</sequence>
<sequence>
<taxon idref="leucochl50"/>
TCGAAACCTGC-ATAGCAG-AGCAACATGCG--AACTTGTCTTTC-
CAACCTTCCTTTTTCCGTGTGGCGGGTCAGACGA-CT------------CGTGCT-
TCCTC-CGCC-TTGCG-GGATGAACAA---CTTTC-GGCGCGGGTTGCGCCAAGGA------
CCATGAACGTTAGGCCTC--GCC-----TCTTTGC-GCCGTTAGGCTGCGTTG-----AG-
GA-G--TC--CCATCATTGAA--AAATA-T-----------CAATTTAATGAC--
TCTTGACAAAGGATATCTCGGCTCTCGCATCGATGAAGAACG--TAGCGAAAT---
GCGATACTTGGTGTGAATTGCA-GAATCCTGTGAACCATTGA---
NTCTTTGAACGCAAGTT-
GCGCCCGAAGCCTTTTGGCCAAGGGCACGTCTGCCTGGGCGTCACGCATAGCGTCGCCCCTA
CC-T-CCCAACTCGTGTTTGG--ATGTGTGT--G-GGGC-
GGAGAGTGGTCTCCCGTGTGC---CTT-GCTCACATGGATGGCCGAAATAAGGAGCTT---
GCGGCGG--TGAGCGCCGTGATGATTGGTGG-TTGGAT-GGGC-TCTT-GCCCTACAA---
AGCATC----TTGTCATGAAGTGCCA-CCCCGTGTTTG-GCTCGGAGGACTCGGTCTGG--

TCGATGTTATCCAGATTTGTGCGC--C-TTTTATTGTCCAC-CAT
</sequence>
<sequence>
<taxon idref="lycopoio55"/>
TCGAAACCTGC-ATAGCAGAAGCAACATGCG--AACTTGTCTTTT-
CAACCTTCCTTTTTCCGTGTGGCGGGTCAGACGA-CT-------------CGTGCT-
TCCTC-CGCC-TTGCG-GGATGAACAA---CTTTC-GGCGCGGGTTGCGCCAAGGA------
CCATGAACGTTAGGCCTC--GCC-----TCTTTGC-GCCGTTAGGCTGCGTTG-----AG-
GA-G--TC--CCATCATTGAA--AAATA-T------------CAATTTAATGAC--
TCTTGACAAAGGATATCTCGGCTCTCGCATCGATGAAGAACG--TAGCGAAAT---
GCGATACTTGGTGTGAATTGCA-GAATCCTGTGAACCATTGA---
NTCTTTGAACGCAAGTT-
GCGCCCGAAGCCTTTTGGCCAAGGGCACGTCTGCCTGGGCGTCACGCATAGCGTCGCCCCTA
CC-T-CCCAACTCGTGTTTGG--ATRTGTGT--G-GGGC-
GGAGAGTGGTCTCCCGTGTGC---CTT-GCTCACATGGATGGCCGAAATAAGGAGCTT---
GCGGCGG--TGAGCGCCGTGATGATTGGTGG-TTGGAT-GGGC-TCTT-GCCCTACAA---
AGCATC----TTGTCATGAAGTGCCA-CCCCGTGTTTG-GCTCGGAGGACTCGGTCTGG--
TCAATGTTATCCAGATTTGTGCGC--C-TTTTATTGTCCAC-CAT
</sequence>
<sequence>
<taxon idref="macrathu10"/>
TCGAAACCTGC-ATAGCAG-AGCAACATGCG--AACTTGTCTTTT-
CAACCTTCC--TTTCCGTGTGGCGGGTCAGACGA-CT-------------CGTGCT-
TCCTC-CGCC-TTGCG-GGATGAACAA---CTTTC-GGCGCGGGTTGCGCCAAGGA------
CCATGAACGTTAGGCCTC--GCC-----TCTTTGC-GCCGTTAGGCTGCGTTG-----AG-
GA-G--TC--CCATCATTGAA--AAATA-T------------CAATTTAATGAC--
TCTTGACAAAGGATATCTCGGCTCTCGCATCGATGAAGAACG--TAGCGAAAT---
GCGATACTTGGTGTGAATTGCA-GAATCCTGTGAACCATTGA---
NTCTTTGAACGCAAGTT-
GCGCCCGAAGCCTTTTGGCCAAGGGCACGTCTGCCTGGGCGTCACGCATAGCGTCGCCCCTA
CC-T-CCCAACTCGTGTTTGG--ATATGTGT--G-GGGC-
GGAGAGTGGTCTCCCGTGTGC---CTT-GCTCACATGGATGGCCGAAATAAGGAGCTT---
GCGGCGG--TGAGCGCCGTGATGATTGGTGG-TTGGAT-GGGC-TCTT-GCCCTACAA---
AGCATC----TTGTCATGAAGTGCCA-CCCCGTGTT-G-GCTCGGAGGACTCGGTCTGG--
TCGATGTTATCCAGATTTGTGCGC--C-TTTTATTGTCCAC-CAT
</sequence>
<sequence>
<taxon idref="peculiar19"/>
TCGAAACCTGC-ATAGCAG-AGCAACATGCG--AACTTGTCTTTT-
CAACCTTCCTTTTTCCGTGTGGCGGGTCGGACGA-CT-------------CGTGCT-
TCCTC-CGCC-TTGCG-GGATGAACGA---CTTTC-GGCGCGGGTTGCGCCAAGGA------
CCATGAACGTTAGGCCTC--GCC-----TCTTTGC-GCTGTTAGGCTGCGTCG-----AG-
GA-G--TC--CCATCATTGAA--AAATA-T------------CAATTTAATGAC--
TCTTGACAAAGGATATCTCGGCTCTCGCATCGATGAAGAACG--TAGCGAAAT---
GCGATACTTGGTGTGAATTGCA-GAATCCTGTGAACCATTGA---
NTCTTTGAACGCAAGTT-
GCGCCCGAAGCCTTTTGGCCAAGGGCACGTCTGCCTGGGCGTCACGCATAGCGTCGCCCCTA
CC-T-CCCAACTCGTGTTTGG--ATGTGTGT--G-GGGC-
GGAGAGTGGTCTCCCGTGTGC---CTT-GCTCACATGGATGGCCGAAATAAGGAGCTT---
GTGGCGG--TGAGCGCCGTGATGATTGGTGG-TTGGAT-GGGC-TCTT-GCCCTACAA---
AGCATC----TTGTCATGAAGTGCCA-CCCCGTGTTTG-GCTCGAAGGACTCGGTCTGG--
TCGATGTTATCCAGATTTGTGCGC--C-TTTTATTGTCCAC-CAT
</sequence>
<sequence>
<taxon idref="physoste55"/>
TCGAAACCTGC-ATAGCAG-AGCAACATGCG--AACTTGTCTTTT-

CAACCTTCC--TTTCCGTGTGGCGGGTCAGACGA-CT------------CGTGCT-
TCCTC-CGCC-TTGCG-GGATGAACAA---CTTTC-GGCGCGGGTTGCGCCAAGGA------
CCATGAACGTTAGGCCTC--GCC-----TCTTTGC-GCCGTTAGGCCGCGTTG-----AG-
GA-G--TC--CCATCATTGAA--AAATA-T-----------CAATTTAATGAC--
TCTTGACAAAGGATATCTCGGCTCTCGCATCGATGAAGAACG--TAGCGAAAT---
GCGATACTTGGTGTGAATTGCA-GAATCCTGTGAACCATTGA---
NTCTTTGAACGCAAGTT-
GCGCCCGAAGCCTTTTGGCCAAGGGCACGTCTGCCTGGGCGTCACGCATAGCGTCGCCCCTA
CC-T-CCCAACTCGTGTTTGG--ATATGTGT--G-GGGC-
GGAGAGTGGTCTCCCGTGTGC---CTT-GCTCACATGGATGGCCGAAATAAGGAGCTT---
GCGGCGG--TGAGCGCCGTGATGATTGGTGG-TTGGAT-GGGC-TCTT-GCCCTACAA---
AGCATC----TTGTCATGAAGTGCCA-CCCCGTGTT-G-GCTCGGAGGACTCGGTCTGG--
TCGATGTTATCCAGATTTGTGCGC--C-TTTTATTGTCCAC-CAT
</sequence>
<sequence>
<taxon idref="pulchell79"/>
TCGAAACCTGC-ATAGCAG-AGCAACATGCG--AACTTGTCATTT-
CAACCTTCC--TTTCCGTGTGGCGGGTCAGACGA-CT------------TGTGCT-
TCCTC-CGCC-TTGCG-GGATGAACAA---CTTTC-GGCGCGGGTTGCGCCAAGGA------
CCATGAACGTTAGGCCTC--GCC-----TCTTTGC-GCCGTTAGGCTGCGTTG-----AG-
GA-G--TC--CCATCATTGAA--AAATA-T-----------CAATTTAATGAC--
TCTTGACAAAGGATATCTCGGCTCTCGCATCGATGAAGAACG--TAGCGAAAT---
GCGATACTTGGTGTGAATTGCA-GAATCCTGTGAACCATTGA---
GTCTTTGAACGCAAGTT-
GCGCCCGAAGCCTTTTGGCCAAGGGCACGTCTGCCTGGGCGTCACGCATAGCGTCGCCCCTA
CC-T-CCCAACTCGTGTTTGG--ATATGTGT--G-GGGC-
GGAGAGTGGTCTCCCGTGTGC---CTT-GCTCACATGGATGGCCGAAATAAGGAGCTT---
GCGGCGG--TGAGCGCCGTGATGATTGGTGG-TTGGAT-GGGC-TCTT-GCCCTACAA---
AACATC----TTGTCATGAAGTGCCA-CCCCGTGTT-G-GCTCGGAGGACTCGGTCTGG--
TCGATGTTATCCAGATTTGTGCGC--C-TTTTATTGTCCAC-CAT
</sequence>
<sequence>
<taxon idref="revolutu15"/>
TCGAAACCTGC-ATAGCAG-AGCAACATGCG--AACTTGTCTTTT-
CAACCTTCCTTTTTCCGTGTGGCGGGTCAGACGA-CT------------CGTGCT-
TCCTC-CGCC-TTGCG-GGATGAACAA---CTTTC-GGCGCGGGTTGCGCCAAGGA------
CCATGAACGTTAGGCCTC--GCC-----TCTTTGC-GCCGTTAGGCTGCGTTG-----AG-
GA-G--TC--CCATCATTGAA--AAATA-T-----------CAATTTAATGAC--
TCTTGACAAAGGATATCTCGGCTCTCGCATCGATGAAGAACG--TAGCGAAAT---
GCGATACTTGGTGTGAATTGCA-GAATCCTGTGAACCATTGA---
NTCTTTGAACGCAAGTT-
GCGCCCGAAGCCTTTTGGCCAAGGGCACGTCTGCCTGGGCGTCACGCATAGCGTCGCCCCTA
CC-T-CCCAACTCGTGTTTGG--ATGTGTGT--G-GGGC-
GGAGAGTGGTCTCCCGTGTGC---CTT-GCTCACATGGATGGCCGAAATAAGGAGCTT---
GCGGCGG--TGAGCGCCGTGATGATTGGTGG-TTGGAT-GGGC-TCTT-GCCCTACAA---
AGCATC----TTGTCATGAAGTGCCA-CCCCGTGTTTG-GCTCGGAGGACTCGGTCTGG--
TCGATGTTATCCAGATTTGTGCGC--C-TTTTATTGTCCAC-CAT
</sequence>
<sequence>
<taxon idref="sakeniW295"/>
TCGAAACCTGC-ATAGCTG-AGCAACATGCG--AACTTGTCTTTT-
CAACCTTCC--TTTCCGTGTGGCGGGTCAGACGA-CT------------CGTGCT-
TCCTC-CGCC-TTGCG-GGATGAACAA---CTTTC-GGCGCGGGTTGCGCCAAGGA------
CCATGAACGTTAGGCCTC--GCC-----TCTTTGC-GCCGTTAGGCTGCGTTG-----AG-
GT-G--TC--CCATCATTGAA--AAATA-T-----------CATATTAATGAC--
TCTTGACAAAGGATATCTCGGCTCTCGCATCGATGAAGAACG--TAGCGAAAT---

GCGATACTTGGTGTGAATTGCA-GAATCCTGTGAACCATTGA---
NTCTTTGAACGCAAGTT-
GCGCCCGAAGCCTTTTGGCCAAGGGCACGTCTGCCTGGGCGTCACGCATAGCGTCGCCCCTA
CC-T-CCCAACTCGTGTTTGG--ATATGTGT--G-GGGC-
GGAGAGTGGTCTCCCGTGTGCCT-CTT-GCTCACATGGATGGCCGAAATAAGGAGCTT---
GCGGCGG--TGAGCGCTGTGACGATTGGTGG-TTGGAT-GGGC-TCTT-GCCCTACAA---
AGCATC----TTGTCACGAAGTGCCA-CCCCGTGTT-G-GCTCGGAGGACTCGGTCTGG--
TCGATGTTATCCAGATTTGTGCGC--C-TTTTATTGTCCAC-CAT
</sequence>
<sequence>
<taxon idref="schizost31"/>
TCGAAACCTGC-ATAGCAG-AGCAACATGCG--AACTTGTCTTTT-
CAACCTTCC--TTTCCGTGTGGCGGGTCAGGCGA-CT------------CGTGCT-
TCCTC-CGCC-TTGCG-GGATGAACAA---CTTTC-GGCGCGGGTTGCGCCAAGGA------
CCATGAACGTTAGGCCTC--GCC-----TCTTTGC-GCCGTTAGGCTGCGTTG-----AG-
GA-G--TC--CCATCATTGAA--AAATA-T-----------CAATTTAATGAC--
TCTTGACAAAGGATATCTCGGCTCTCGCATCGATGAAGAACG--TAGCGAAAT---
GCGATACTTGGTGTGAATTGCA-GAATCCTGTGAACCATTGA---
NTCTTTGAACGCAAGTT-
GCGCCCGAAGCCTTTTGGCCAAGGGCACGTCTGCCTGGGCGTCACGCATAGCGTCGCCCCTA
CC-T-CCCAACTCGTGTTTGG--ATATGTGT--G-GGGC-
GGAGAGTGGTCTCCCGTGTGC---CTT-GCTCACATGGATGGCCGAAATAAGGAGCTT---
GCGGCGG--TGAGCGCCGTGATGATTGGTGG-TTGGAT-GGGC-TCTT-GCCCTACAA---
AGCATC----TTGTCATGAAGTGCCA-CCCCGTGTT-G-GCTCGGAGGACTCGGTCTGG--
TCGATGTTATCCAGATTTGTGCGC--C-TTTTATTGTCCAC-CAT
</sequence>
<sequence>
<taxon idref="senga85995"/>
TCGAAACCTGC-ATAGCAG-AGCAACATGCG--AACTTGTCTTTT-
CAACCTTCC--TTTCCGTGTGGCGGGTTAGAAGA-CT------------CGTGCT-
TCCTC-CGCC-TTGCG-GGATGAACAA---TTTTC-GGCGCGGGTTGCGCCAAGGA------
CCATGAACGTTAGGCCTT--GCC-----TCTTTGC-GCAGTTAGGATGCGTTG-----AG-
GA-G--CC--CCATCATTGAA--AACTA-T-----------CTATTGAATGAC--
TCTCGACAAAGGATATCTCGGCTCTCGCATCGATGAAGAACG--TAGCGAAAT---
GCGATACTTGGTGTGAATTGCA-GAATCCTGTGAACCATTGA---
TTCTTTGAACGCAAGTT-
GCGCCTGAAGCCTTTTGGCCAAGGGCACGTCTGCCTGGGCGTCACGCATCGCGTCGCCCCTA
CC-T-CCCAACTCGTGTTTGG--ATATGTGT--G-GGGC-
GGAGAGTGGCCTCCCGTGTGCCT-CTT-GCTCACATGGATGGCCGAAATAAGGAGCTT---
GTGGCAG--CGAGCGCTGTGATGATTGGTGG-TTGGAT-GGGC-TCTT-GCCTTACAA---
AGCATC----TTGTCACGAAGCGCCG-CCCCGTGTT-G-GCTTGCAGGACTCGACCTGG--
TCGATGTTATCCCGATTTGTGTGC--C-TTTTATCGTCCAC-CAT
</sequence>
<sequence>
<taxon idref="solidum174"/>
TCGAAACCTGC-ATAGCAG-AGCAACATGCG--AACTTGTCTTTT-
CAACCTTCC--TTTCCGTGTGGCGGGTCAGACGA-CT------------CGTGCT-
TCCTC-CGCC-TTGCG-GGATGAACAA---CTTTC-GGCGCGGGTTGCGCCAAGGA------
CCATGAACGTTAGGCCTC--GCC-----TCTTTGC-GCCGTTAGGCCGCGTTG-----AG-
GA-G--TC--CCATCATTGAA--AAATA-T-----------CAATTTAATGAC--
TCTTGACAAAGGATATCTCGGCTCTCGCATCGATGAAGAACG--TAGCGAAAT---
GCGATACTTGGTGTGAATTGCA-GAATCCTGTGAACCATTGA---
NTCTTTGAACGCAAGTT-
GCGCCCGAAGCCTTTTGGCCAAGGGCACGTCTGCCTGGGCGTCACGCATAGCGTCGCCCCTA
CC-T-CCCAACTCGTGTTTGG--ATATGTGT--G-GGGC-
GGAGAGTGGTCTCCCGTGTGG---CTT-GCTCACATGGATGGCCGAAATAAGGAGCTT---

GCGGCGG--TGAGGGCCGTGATGATTGGTGG-TTGGAT-GGGC-TCTT-GCCCTACAA---
AGCATC----TTGTCATGAAGTGCCA-CCCCGTGTT-G-GCTCGGAGGACTCGGTCTGG--
TCGATGTTATCCAGATTTGTGCGC--C-TTTTATGGTCCAC-CAT
</sequence>
<sequence>
<taxon idref="stereop252"/>
TCGAAACCTGC-ATAGCAG-AGCAACATGCG--AACTTGTCTTTT-
CAACCTTCC--TTTCCGTGTGGCGGGTCAGAAGA-CT------------CGTGCT-
TCCTC-CGCC-TTGCG-GGATGAACAA---CTTTC-GGCGCGGGTTGCGCCAAGGA------
CCATGAACGTTAGGCCTC--GCC-----TCTTTGC-GCCGTTAGGCTGCGTTG-----AG-
GA-G--TC--CCATCATTGAA--AAATA-T-----------CAATTTAATGAC--
TCTTGACAAAGGATATCTTGGCTCTCGCATCGATGAAGAACG--TAGCGAAAT---
GCGATACTTGGTGTGAATTGCA-GAATCCTGTGAACCATTGA---
NTCTTTGAACGCAAGTT-
GCGCCCGAAGCCTTTTGGCCAAGGGCACGTCTGCCTGGGCGTCACGCATAGCGTCGCCCCTA
CC-T-CCCAACTCGTGTTTGG--ATATGTGT--G-GGGC-
GGAGAGTGGTCTCCCGTGTGC---CTT-GCTCACATGGATGGCCGAAATAAGGAGCTT---
GCGGCGG--TGAGCGCCGTGATGATTGGTGG-TTGGAT-GGGC-TCTT-GCCCTACAA---
AGCATC----TTGTCATGAAGTGCCA-CCCCGTGTT-G-GCTCGGAGGACTCGATCTGG--
TCGATGTTATCCAGATTTGTGCGC--C-TTTTATTGTCCAC-CAT
</sequence>
<sequence>
<taxon idref="subulat221"/>
TCGAAACCTGC-ATAGCAG-AGCAACATGCG--AACTTGTCTTTT-
CAACCTTCCTTTTTCCGTGTGGCGGGTCAGACGA-CT------------CGTGCT-
TCCTC-CGCC-TTGCG-GGATGAACAA---CTTTC-GGCGCGGGTTGCGCCAAGGA------
CCATGAACGTTAGGCCTC--GCC-----TCTTTGC-GCCGTTAGGCTGCGTTG-----GG-
GA-G--TC--CCATCATTGAA--AAATA-T-----------CAATTTAATGAC--
TCTTGACAAAGGATATCTCGGCTCTCGCATCGATGAAGAACG--TAGCGAAAT---
GCGATACTTGGTGTGAATTGCA-GAATCCTGTGAACCATTGA---
GTCTTTGAACGCAAGTT-
GCGCCCGAAGCCTTTTGGCCAAGGGCACGTCTGCCTGGGCGTCACGCATAGCGTCGCCCCTA
CC-T-CCCAACTCGTGTTTGG--ATGTGTGT--G-GGGC-
GGAGAGTGGTCTCCCGTGTGC---CTT-GCTCACATGGATGGCCGAAATAAGGAGCTT---
GCGGCGG--TGAGCGCCGTGATGATTGGTGG-TTGGAT-GGGC-TCTT-GCCCTACAA---
AGCATC----TTGTCATGAAGTGCCA-CCCCGTGTTTG-GCTCGGAGGACTCGGTCTGG--
TCGATGTTATCCAGATTTGTGCGC--C-TTTTATTGTCCAC-CAT
</sequence>
<sequence>
<taxon idref="Talagon103"/>
TCGAAACCTGC-ATAGCAG-AGCAACATGCG--AACTTGTCTTTT-
CAACCTTCCTTTTTCCGTGTGGCGGGTCAGACGA-CT------------CGTGCT-
TCCTC-CGCC-TTGCG-GGATGAACAA---CTTTC-GGCGCGGGTTGCGCCAAGGA------
CCATGAACGTTAGGCCTC--GCC-----TCTTTGC-GCCGTTAGGCTGCGTTG-----AG-
GA-G--TC--CCATCATTGAA--AAATA-T-----------CAATTTAATGAC--
TCTTGACAAAGGATATCTCGGCTCTCGCATCGATGAAGAACG--TAGCGAAAT---
GCGATACTTGGTGTGAATTGCA-GAATCCTGTGAACCATTGA---
GTCTTTGAACGCAAGTT-
GCGCCCGAAGCCTTTTGGCCAAGGGCACGTCTGCCTGGGCGTCACGCATAGCGTCGCCCCTA
CC-T-CCCAACTCGTGTTTGG--ATGTGTGT--G-GGGC-
GGAGAGTGGTCTCCCGTGTGC---CTT-GCTCACATGGATGGCAGAAATAAGGAGCTT---
GCGGCGG--TGAGCGCCGTGATGATTGGTGG-TTGGAT-GGGC-TCTT-GCCCTACAA---
AGCATC----TTGTCATGAAGTGCCA-CCCCGTGTT-G-GCTCGGAGGACTCGGTCTGG--
TCGATGTTATCCAGATTTGTGCGC--C-TTTTATTGTCCAC-CAT
</sequence>
<sequence>

<taxon idref="Zaprjaga24"/>
TCGAAACCTGC-ATAGCAG-AGCAACATGCG--AACTTGTCTTTT-
CAACCTTCTATTTTCCGTGTGGCGGGTCAGACGA-CT------------CGTGCT-
TCCTC-CGCC-TTGCG-GGATGAACAA---CTTTC-GGCGCGGGTTGCGCCAAGGA------
CCATGAACGTTAGGCCTC--GCC-----TCTTTGC-GCCGTTAGGCTGCGTTG-----AG-
GA-G--TC--CCATCATTGAA--AAATA-T-----------CAATTTAATGAC--
TCTTGACAAAGGATATCTCGGCTCTCGCATCGATGAAGAACG--TAGCGAAAT---
GCGATACTTGGTGTGAATTGCA-GAATCCTGTGAACCATTGA---
NTCTTTGAACGCAAGTT-
GCGCCCGAAGCCTTTTGGCCAAGGGCACGTCTGCCTGGGCGTCACGCATAGCGTCGCCCCTA
CC-T-CCCAACTCGTGTTTGG--ATGTGTGT--G-GGGC-
GGAGAGTGGTCTCCCGTGTGC---CTT-GCTCACATGGATGGCCGAAATAAGGAGCTT---
GCGGCGG--TGAGCGCCGTGATGATTGGTGG-TTGGAT-GGGC-TCTT-GCCCTACAA---
AGCATC----TTGTCATGAAGTGCCA-CCCCGTGTTTG-GCTCGGAGGACTCGGTCTGG--
TCGATGTTATCCAGATTTGTGCGC--C-TTTTATTGTCCAC-CAT
</sequence>
<sequence>
<taxon idref="acerosum08"/>
TCGAAACCTGC-ATAGCAG-AGCAACATGCG--AACTTGTCTTTT-
TAACCTTCC--TTTCCGTGTGGCGGGTCAGAAGA-CT------------CGT-CT-
TCCTC-CGCC-TTGCG-GGATGAACAA---TTTTC-GGCGCGGGTTGCGCCAAGGA------
CCATGAACGTTAGGCCTT--GCC-----TCTTTGC-GCCGTTAGGCTGCGTTG-----AG-
GA-G--CC--CCATCATTGAA--AACTA-T-----------CTATTGAATGAC--
TCTCGACAAAGGATATCTCGGCTCTCGCATCGATGAAGAACG--TAGCGAAAT---
GCGATACTTGGTGTGAATTGCA-GAATCCTGTGAACCATTGA---
TTCTTTGAACGCAAGTT-
GCGCCTGAAGCCTTTTGGCCAAGGGCACGTCTGCCTGGGCGTCACGCATCGCGTCGCCCCTA
CC-T-CCCAACTCGTGTTTGG--ATATGTGT--G-GGGC-
GGAGAGTGGCCTCCCGTGTGCCT-CTT-GCCCACATGGATGGCCGAAATAAGGAGCTT---
GTGGCAG--CGAGCGCTGTGATGATTGGTGG-TTGGAT-GGGC-TTTT-GCCTTACAA---
AGCATC----TTGTCACGAAGCGCCG-CCCCGTGTT-G-GCTTGGAGGACTCGACCTGG--
TCGATGTTATCCCGATTTGTGTGC--C-TTTTATCGTCCAC-CAT
</sequence>
<sequence>
<taxon idref="alatavic81"/>
TCGAAACCTGC-ATAGCAG-AGCAACATGCG--AACTTGTCTTTT-
CAACCTTCC--TTTCCGTGTGGCGGGTCAGACGA-CT------------CGTGCT-
TCCTC-CGCC-TCGCG-GGATGAACAA---CTTTC-GGCGCGGGTTGCGCCAAGGA------
CCATGAACGTTAGGCCTY--GCC-----TCTTTGC-GCCGTTAGGCTGCGTTG-----AG-
GA-G--TC--CCATCATTGAA--CAATA-T-----------CAATTTAATGAC--
TCTTGACAAAGGATATCTCGGCTCTCGCATCGATGAAGAACG--TAGCGAAAT---
GCGATACTTGGTGTGAATTGCA-GAATCCTGTGAACCATTGA---
GTCTTTGAACGCAAGTT-
GCGCCCGAAGCCTTTTGGCCAAGGGCACGTCTGCCTGGGCGTCACGCATAGCGTCGCCCCTA
CC-T-CCCAACTCGTGTTTGG--ATATGTGT--G-GGGC-
GGAGAGTGGTCTCCCGTGTGCCT-CTT-GCTCACATGGATGGCCGAAATAAGGAGCTT---
GCGGCGG--TGAGCGCCGTGATGATTGGTGG-TTGGAT-GGGC-TCTT-GCCCTACAA---
AGCATC----TTGTCACGAAGTGCCA-CCCCGTGTT-G-GCTCGGAGGACTCGGTCTGG--
TCGATGTTATCCAGATTTGTGCGC--C-TTTTATTGTCCAC-CAT
</sequence>
<sequence>
<taxon idref="armenum647"/>
TCGAAACCTGC-ATAGCAG-AGCAACATGCG--AACTTGTCTTTT-
TAACCTTCC--TATCCGTGTGGCGGGTCAGAAGA-CT------------CGT-CT-
TCCTC-CGCC-TTGCG-GGATGAACAA---TTTTC-GGCGCGGGTTGCGCCAAGGA------
CCATGAACGTTAGGCCTT--GCC-----TCTTTGC-GCCGTTAGGCTGCGTTG-----AG-

GA-G--CC--CCATCATTGAA--AACTA-T-----------CTATTGAATGAC--
TCTCGACAAAGGATATCTCGGCTCTCGCATCGATGAAGAACG--TAGCGAAAT---
GCGATACTTGGTGTGAATTGCA-GAATCCTGTGAACCATTGA---
TTCTTTGAACGCAAGTT-
GCGCCTGAAGCCTTTTGGCCAAGGGCACGTCTGCCTGGGCGTCACGCATCGCGTCGCCCCTA
CC-T-CCCAACTCGTGTTTGG--ATATGTGT--G-GGGC-
GGAGAGTGGCCTCCCGTGTGCCT-CTT-GCCCACATGGATGGCCGAAATAAGGAGCTT---
GTGGCAG--CGAGCGCTGTGATGATTGGTGG-TTGGAT-GGGC-TTTT-GCCTTACAA---
AGCATC----TTGTCACGAAGCGCCG-CCCCGTGTT-G-GCTTGGAGGACTCGACCTGG--
TCGATGTTATCCCGATTTGTGTGC--C-TTTTATCGTCCAC-CAT
</sequence>
<sequence>
<taxon idref="Takhtaj678"/>
TCGAAACCTGC-RTAGCAG-AGCAACATGCG--AACTTGTCTTCT-
CAACCTTCC--TTTCCGTGTGGCGGGTCAGAAGA-CT-------------CGTGCT-
TCCTC-CGCC-TTGCG-GGATGAACAA---TTTTC-GGCGCGGGTTGCGCCAAGGR------
CCATGAACGTTAGGCGCT--GCC-----TCTTTGG--CCGTTAGGCTGCGTTG-----AG-
GA-G--CC--CCATCATTGAA--AACTA-T-----------CTATYGAATGAC--
TCTCGACAAAGGATATCTCGGCTCTCGCATCGATGAAGAACG--TAGCGAAAT---
GCGATACTTGGTGTGAATTGCA-GAATCCTGTGAACCATTGA---
TTCTTTGAACGCAAGTT-
GCGCCTGAAGCCTTTTGGCCAAGGGCACGTCTGCCTGGGCGTCACGCATCGCGTCGCCCCTA
CC-T-CCCAACTCGTGTTTGG--ATATGTGT--G-GGGC-
GGAGAGTGGCCTCCCGTGTGCCT-CTT-GCTCACATGGATGGCCGAAATAAGGAGCTT---
GTGGCAG--CGAGCGCTGTGATGATTGGTGG-TTGGAT-GGGCTTTTT-GCCTTACAA---
AGCATC----TTGTCACGAAGCGCCG-CCCCGTGTT-G-GCTTGGAGGACTCGACCTGG--
TCGATGTTGTCCCGATTTGTGTGC--C-TTTTATCGTCCAC-CAT
</sequence>
<sequence>
<taxon idref="aulieten67"/>
TCGAAACCTGC-ATAGCTG-AGCAACATGCG--AACTTGTCTTTT-
CAACCTTCC--TTTCCGTGTGGCGGGTCAGACGA-CT-------------CGTGCT-
TCCTC-CGCC-TTGCG-GGATGAACAA---CTTTC-GGCGCGGGTTGCGCCAAGGA------
CCATGAACGTTAGGCCTC--GCC-----TCTTTGC-GCCGTTAGGCTGCGTTG-----AG-
GA-G--TC--CCATCATTGAA--AAATA-T-----------CATATTAATGAC--
TCTTGACAAAGGATATCTCGGCTCTCGCATCGATGAAGAACG--TAGCGAAAT---
GCGATACTTGGTGTGAATTGCA-GAATCCTGTGAACCATTGA---
GTCTTTGAACGCAAGTT-
GCGCCCGAAGCCTTTTGGCCAAGGGCACGTCTGCCTGGGCGTCACGCATAGCGTCGCCCCTA
CC-T-CCCAACTCGTGTTTGG--ATATGTGT--G-GGGC-
GGAGAGTGGTCTCCCGTGTGCCT-CTT-GCTCACATGGATGGCCGAAATAAGGAGCTT---
GCGGCGG--TGAGCGCTGTGATGATTGGTGG-TTGGAT-GGGC-TCTT-GCCCTACAA---
AGCATC----TTGTCACGAAGTGCCA-CCCCGTGTT-G-GCTCGGAGGACTCGGTCTGG--
TCGATGTTATCCAGATTTGTGCGC--C-TTTTATTGTCCAC-CAT
</sequence>
<sequence>
<taxon idref="compact047"/>
TCGAAACCTGC-ATAGCAG-AGCAACATGCG--AACTTGTCTTTT-
CAACCTTCC--TTTCCGTGTGGCGGGTCAGAAGA-CT-------------CGTGCT-
TCCTC-CGCC-TTGCG-GGATGAACAA---TTTCC-GGCGCGGGTTGCGCCAAGGA------
CCATGAACGTTAGGCCTT--GCC-----TCTTTGC-GCCGTTAGGCTGCGTTG-----AG-
GA-G--CC--CCACCATTGAA--AACTA-T-----------CAATTGAATGAC--
TCTCGACAAAGGATATCTCGGCTCTCGCATCGATGAAGAACG--TAGCGAAAT---
GCGATACTTGGTGTGAATTGCA-GAATCCTGTGAACCATTGA---
TTCTTTGAACGCAAGTT-
GCGCCTGAAGCCTTTTGGCCAAGGGCACGTCTGCCTGGGCGTCACGCATCGCGTCGCCCCTA

CC-T-CCCAACTCGTGTTTGG--ATATGTGT--A-GGGC-
GGAGAGTGGCCTCCCGTGTGGCT-CTTTGCTCACATGGATGGCCGAAATAAGGAGCTT---
GTGGCAG--CGAGCGCTGTGATGAATGGTGG-TTGGAC-GGGC-TCTT-GCCTTACAA---
AGCATC----TTGTCACGAAGCGCCG-CCCCGTGTT-G-GCTTGGAGGACTCGACCTGG--
TCGATGTTATCCCGATTTGTGTGC--C-TTTTATCGTCCAC-CAT
</sequence>
<sequence>
<taxon idref="diapens252"/>
TCGAAACCTGC-ATAGCAG-AGCAACATGCG--AACTTGTCTTTT-
CAACCTTCC--TTTCCGTGTGGCGGGTCAGAAGA-CT------------CGTGCT-
TCCTC-CGCC-TTGCG-GGATGAACAA---TTTCC-GGCGCGGGTTGCGCCAAGGA------
CCATGAACGTTAGGCCTT--GCC-----TCTTTGC-GCCGTTAGGCTGCGTTG-----AG-
GA-G--CC--CCACCATTGAA--AACTA-T-----------CAATTGAATGAC--
TCTCGACAAAGGATATCTCGGCTCTCGCATCGATGAAGAACG--TAGCGAAAT---
GCGATACTTGGTGTGAATTGCA-GAATCCTGTGAACCATTGA---
TTCTTTGAACGCAAGTT-
GCGCCTGAAGCCTTTTGGCCAAGGGCACGTCTGCCTGGGCGTCACGCATCGCGTCGCCCCTA
CC-T-CCCAACTCGTGTTTGG--ATATGTGT--A-GGGC-
GGAGAGTGGCCTCCCGTGTGGCT-CTT-GCTCACATGGATGGCCGAAATAAGGAGCTT---
GTGGCAG--CGAGCGCTGTGATGATTGGTGG-TTGGAC-GGGC-TCTT-GCCTTACAA---
AGCATC----TTGTCACGAAGCGCCG-CCCCGTGTT-G-GCTTGGAGGACTCGACCTGG--
TCGATGTTAKCCCGATTTGTGTGC--C-TTTTGTCGTCCAC-CAT
</sequence>
<sequence>
<taxon idref="ecae024754"/>
TCGAAACCTGC-ATAGCAG-AGCAACATGCG--AACTTGTCTTTT-
CAACCTTCC--TTTCCGTGTGGCGGGTCGGACGA-CT------------CGTGCT-
TCCTC-CGCC-TTGCG-GGATGAACAA---CTTTC-GGCGCGGGTTGCGCCAAGGA------
CCATGAACGTTAGGCCTC--GCC-----TCTTTGC-GCCGTTAGGCTGCGTTG-----AG-
GA-G--TC--CCATCATTGAA--AAATA-T-----------SAATTTAATGAC--
TCTTGACAAAGGATATCTCGGCTCTCGCATCGATGAAGAACG--TAGCGAAAT---
GCGATACTTGGTGTGAATTGCA-GAATCCTGTGAACCATTGA---
GTCTTTGAACGCAAGTT-
GCGCCCGAAGCCTTTTGGCCAAGGGCACGTCTGCCTGGGCGTCACGCATAGCGTCGCCCCTA
CC-T-CCCAACTCGTGTTTGG--ATATGTGT--G-GGGC-
GGAGAGTGGTCTCCCGTGTGC---CTT-GCTCACATGGATGGCCGAAATAAGGAGCTT---
GCGGCGG--TGAGCGCCGTGATGATTGGTGG-TTGGAT-GGGC-TCTT-GCCCTACAA---
AGCATC----TTGTCATGAAGTGCCA-CCCCGTGTT-G-GCTCGGAGGACTCGGTCTGG--
TCGATGTTATCCAGATTTGTGCGC--C-TTTTATTGTCCAC-CAT
</sequence>
<sequence>
<taxon idref="ekatheri80"/>
TCGAAACCTGC-ATAGCTG-AGCAACATGCG--AACTTGTCTTTT-
CAACCTTCC--TTTCCGTGTGGCGGGTCAGACGA-CT------------CGTGCT-
TCCTC-CGCC-TTGCG-GGATGAACAA---CTTTC-GGCGCGGGTTGCGCCAAGGA------
CCATGAACGTTAGGCCTC--GCC-----TCTTTGCCGCCGTTAGGCTGCGTTG-----AG-
GA-G--TC--CCATCATTGAA--AAATA-T-----------CATATTAATGAC--
TCTTGACAAAGGATATCTCGGCTCTCGCATCGATGAAGAACG--TAGCGAAAT---
GCGATACTTGGTGTGAATTGCA-GAATCCTGTGAACCATTGA---
GTCTTTGAACGCAAGTT-
GCGCCCGAAGCCTTTTGGCCAAGGGCACGTCTGCCTGGGCGTCACGCATAGCGTCGCCCCTA
CC-T-CCCAACTCGTGTTTGG--ATATGTGT--G-GGGC-
GGAGAGTGGTCTCCCGTGTGCCT-CTT-GCTCACATGGTTGGCCGAAATAAGGAGCTT---
GCGGCGG--TGAGCGCTGTGATGATTGGTGG-TTGGAT-GGGC-TCTT-GCCCTACAA---
AGCATC----TTGTCACGAAGTGCCA-CCCCGTTTT-G-GCTTGGAGGACTCGGTCTGG--
TCGATGTTATCCAGATTTGTGCGC--C-TTTTATTGTCCAC-CAC

</sequence>
<sequence>
<taxon idref="ekbergia42"/>
TCGAAACCTGC-ATAGCAG-AGCAACATGCG--AACTTGTCTTTT-
CAACCTTCC--TTTCCGTGTGGCGGGTCAGAAGA-CT------------CGTGCT-
TCCTC-CGCC-TTGCG-GGATGAACAA---TTTCC-GGCGCGGGTTGCGCCAAGGA------
CCATGAACGTTAGGCCTT--GCC-----TCTTTGC-GCCGTTAGGCTGCGTTG-----AG-
GA-G--CC--CCACCATTGAA--AACTA-T-----------CAATTGAATGAC--
TCTCGACAAAGGATATCTCGGCTCTCGCATCGATGAAGAACG--TAGCGAAAT---
GCGATACTTGGTGTGAATTGCA-GAATCCTGTGAACCATTGA---
TTCTTTGAACGCAAGTT-
GCGCCTGAAGCCTTTTGGCCAAGGGCACGTCTGCCTGGGCGTCACGCATCGCGTCGCCCCTA
CC-T-CCCAACTCGTGTTTGG--ATATGTGT--A-GGGC-
GGAGAGTGGCCTCCCGTGTGGCT-CTT-GCTCACATGGATGGCCGAAATAAGGAGCTT---
GTGGCAG--CGAGCGCTGTGATGATTGGTGG-TTGGAC-GGGC-TCTT-GCCTTACAA---
AGCATC----TTGTCACGAAGCGCCG-CCCCGTGTT-G-GCTTGGAGGACTCGACCTGG--
TCGATGTTATCCCGATTTGTGTGC--C-TTTTGTCGTCCAC-CAT
</sequence>
<sequence>
<taxon idref="erythrae98"/>
TCGAAACCTGC-ATAGCAG-AGCAACATGCG--AACTTGTCTTTT-
CAACCTTCCTTTTTCCGTGTGGCGGGTCAGACGA-CT------------TGTGCT-
TCCTC-CGCC-TTGCG-GGATGAACAA---CTTTC-GGCGCGGGTTGCGCCAAGGA------
CCATGAACGTTAGGCCTC--GCC-----TCTTTGC-GCCGTTAGGCTGCGTTG-----AG-
GA-G--TC--CCATCATTGAA--AAATA-T-----------CAATTTAATGAC--
TCTTGACAAAGGATATCTCGGCTCTCGCATCGATGAAGAACG--TAGCGAAAT---
GCGATACTTGGTGTGAATTGCA-GAATCCTGTGAACCATTGA---
GTCTTTGAACGCAAGTT-
GCGCCCGAAGCCTTTTGGCCAAGGGCACGTCTGCCTGGGCGTCACGCATAGCGTCGCCCCTA
CC-T-CCCAACTCGTGTTTGG--ATGTGTGT--G-GGGC-
GGAGAGTGGTCTCCCGTGTGC---CTT-GCTCACATGGATGGCCGAAATAAGGAGCTT---
GCGGCGG--TGAGCGCCGTGATGATTGGTGG-TTGGAT-GGGC-TCTT-GCCCTACAA---
AGCATC----TTGTCATGAAGTGCCA-CCCYGTGTTTG-GCTTGGAGGACTCGGTCTGG--
TCGATGTTATCCAGATTTGTGCGC--C-TTTTATTGTCCAC-CAT
</sequence>
<sequence>
<taxon idref="federov277"/>
TCGAAACCTGC-ATAGCAG-AGCAACATGCG--AACTTGTCTTTT-
CAACCTTCCTTTTTCCGTGTGGCGGGTCAGACGA-CT------------CGTGCT-
TCCTC-CGCC-TTGCG-GGATGAACAA---CTTTC-GGCGCGGGTTGCGCCAAGGA------
CCATGAACGTTAGGCCTC--GCC-----TCTTTGC-GCCGTTAGGCTGCGTTG-----AG-
GA-G--TC--CCATCATTGAA--AAATA-T-----------CAATTTAATGAC--
TCTTGACAAAGGATATCTCGGCTCTCGCATCGATGAAGAACG--TAGCGAAAT---
GCGATACTTGGTGTGAATTGCA-GAATCCTGTGAACCATTGA---
GTCTTTGAACGCAAGTT-
GCGCCCGAAGCCTTTTGGCCAAGGGCACGTCTGCCTGGGCGTCACGCATAGCGTCGCCCCTA
CC-T-CCCAACTCGTGTTTGG--ATGTGTGT--G-GGGC-
GGAGAGTGGTCTCCCGTGTGC---CTT-GCTCACATGGATGGCAGAAATAAGGAGCTT---
GCGGCGG--TGAGCGCCGTGACGATTGGTGG-TTGGAT-GGGCTTCTT-GCCCTACAA---
AGCATC----TTGTCATGAAGTGCCA-CCCCGTGTT-G-GCTCGGAGGACTCGGTCTGG--
TCGATGTTATCCAGATTTGTGTGC--C-TTTTATTGTCCAC-CAT
</sequence>
<sequence>
<taxon idref="glumace690"/>
TCGAAACCTGC-ATAGCAG-AGCAACATGCG--AACTTGTCTTTT-
CAACCTTCC--TTTCCGTGTGGCGGGTCAGACGA-CT------------CGTGCT-

TCCTC-CGCC-TCGCG-GGATGAACAA---CTTTC-GGCGCGGGTTGCGCCAAGGA------
CCATGAACGTTAGGCCTT--GCC-----TCTTTGC-GCCGTTAGGCTGCGTTG-----AG-
GA-G--TC--CCATCATTGAA--CAATA-T-----------CAATTTAATGAC--
TCTTGACAAAGGATATCTCGGCTCTCGCATCGATGAAGAACG--TAGCGAAAT---
GCGATACTTGGTGTGAATTGCA-GAATCCTGTGAACCATTGA---
GTCTTTGAACGCAAGTT-
GCGCCCGAAGCCTTTTGGCCAAGGGCACGTCTGCCTGGGCGTCACGCATAGCGTCGCCCCTA
CC-T-CCCAACTCGTGTTTGG--ATATGTGT--G-GGGC-
GGAGAGTGGTCTCCCGTGTGCCT-CTT-GCTCACATGGATGGCCGAAATAAGGAGCTT---
GCGGCGG--TGAGCGCCGTGATGATTGGTGG-TTGGAT-GGGC-TCTT-GCCCTACAA---
AGCATC----TTGTCACGAAGTGCCA-CCCCGTGTT-G-GCTCGGAGGACTCGGTCTGG--
TCGATGTTATCCAGATTTGTGCGC--C-TTTTATTGTCCAC-CAT
</sequence>
<sequence>
<taxon idref="glutinos37"/>
TCGAAACCTGC-ATAGCAG-AGCAACATGCG--AACTTGTCTTTT-
CAACCTTCC--TTTCCGTGTGGCGGGTCAGACGA-CT------------CGTGCT-
TCCTC-CGCC-TTGCG-GGATGAACAA---CTTTC-GGCGCGGGTTGCGCCAAGGA------
CCATGAACGTTAGGCCTC--GCC-----TCTTTGC-GCCGTTAGGCTGCGTTG-----AG-
GA-G--TC--CCATCATTGAA--AAATA-T-----------CAATCTAATGAC--
TCTTGACAAAGGATATCTCGGCTCTCGCATCGATGAAGAACG--TAGCGAAAT---
GCGATACTTGGTGTGAATTGCA-GAATCCTGTGAACCATTGA---
GTCTTTGAACGCAAGTT-
GCGCCCGAAGCCTTTTGGCCAAGGGCACGTCTGCCTGGGCGTCACGCATAGCGTCGCCCCTA
CC-T-CCCAACTCGTGTTTGG--ATATGTGT--G-GGGC-
GGAGAGTGGTCTCCCGTGTGC---CTT-GCTCACATGGATGGCCGAAATAAGGAGCTT---
GCGGCGG--TGAGCGCCGTGATGATTGGTGG-TTGGAT-GGGC-TCTT-GCCCTACAA---
AGCATC----TTGTCATGAAGTGCCA-CCCCGTGTT-G-GCTCGGAGGACTCGGTCTGG--
TCGATGTTATCCAGATTTGTGCGC--A-TTTTATTGTCCAC-CAT
</sequence>
<sequence>
<taxon idref="hypochar7h"/>
TCGAAACCTGC-ATAGCAG-AGCAACATGCG--AACTTGTCTTTT-
CAACCTTCC--TTTCCGTGTGGCGGGTCAGACGA-CT------------CGTGCT-
TCCTC-CGCC-TCGCG-GGATGAACAA---CTTTC-GGCGCGGGTTGCGCCAAGGA------
CCATGAACGTTAGGCCTT--GCC-----TCTTTGC-GCCGTTAGGCTGCGTTG-----AG-
GA-G--TC--CCATCATTGAA--CAATA-T-----------CAATTTAATGAC--
TCTTGACAAAGGATATCTTGGCTCTCGCATCGATGAAGAACG--TAGCGAAAT---
GCGATACTTGGTGTGAATTGCA-GAATCCTGTGAACCATTGA---
GTCTTTGAACGCAAGTT-
GCGCCCGAAGCCTTTTGGCCAAGGGCACGTCTGCCTGGGCGTCACGCATAGCGTCGCCCCTA
CC-T-CCCAACTCGTGTTTGG--ATATGTGT--G-GGGC-
GGAGAGTGGTCTCCCGTGTGCCT-CTT-GCTCACATGGATGGCCGAAATAAGGAGCTT---
GCGGCGG--TGAGCGTCGTGATGATTGGTGG-TTGGAT-GGGC-TCTT-GCCCTACAA---
AGCATC----TTGTCACGAAGTGCCA-CCCCGTGTT-G-GCTCGGAGGACTCGGTCTGG--
TCGATGTTATCCAGATTTGTGCGC--C-TTTTATTGTCCAC-CAT
</sequence>
<sequence>
<taxon idref="knorri9KYG"/>
TCGAAACCTGC-ATAGCTG-AGCAACATGCG--AACTTGTCTTTT-
CAACCTTCC--TTTCCGTGTGGCGGGTCAGACGA-CT------------CGTGCT-
TCCTC-CGCC-TTGCG-GGATGAACAA---CTTTC-GGCGCGGGTTGCGCCAAGGA------
CCATGAACGTTAGGCCTC--GCC-----TCTTTGC-GCCGTTAGGCTGCGTTG-----AG-
GT-G--TC--CCATCATTGAA--AAATA-T-----------CATATTAATGAC--
TCTTGACAAAGGATATCTCGGCTCTCGCATCGATGAAGAACG--TAGCGAAAT---
GCGATACTTGGTGTGAATTGCA-GAATCCTGTGAACCATTGA---

GTCTTTGAACGCAAGTT-
GCGCCCGAAGCCTTTTGGCCAAGGGCACGTCTGCCTGGGCGTCACGCATAGCGTCGCCCCTA
CC-T-CCCAACTCGTGTTTGG--ATATGTGT--G-GGGC-
GGAGAGTGGTCTCCCGTGTGCCT-CTT-GCTCACATGGATGGCCGAAATAAGGAGCTT---
GCGGCGG--TGAGCGCTGTGACGATTGGTGG-TTGGAT-GGGC-TCTT-GCCCTACAA---
AGCATC----TTGTCACGAAGTGCCA-CCCCGTGTT-G-GCTCGGAGGACTCGGTCTGG--
TCGATGTTATCCAGATTTGTGCGC--C-TTTTATTGTCCAC-CAT
</sequence>
<sequence>
<taxon idref="laxuW2055K"/>
TCGAAACCTGC-ATAGCTG-AGCAACATGCG--AACTTGTCTTTT-
CAACCTTCC--TTTCCGTGTGGCGGGTCAGACGA-CT------------CGTGCT-
TCCTC-CGCC-TTGCG-GGATGAACAA---CTTTC-GGCGCGGGTTGCGCCAAGGA------
CCATGAACGTTAGGCCTC--GCC-----TCTTTGC-GCCGTTAGGCTGCGTTG-----AG-
GT-G--TC--CCATCATTGAA--AAATA-T-----------CATATTAATGAC--
TCTTGACAAAGGATATCTCGGCTCTCGCATCGATGAAGAACG--TAGCGAAAT---
GCGATACTTGGTGTGAATTGCA-GAATCCTGTGAACCATTGA---
GTCTTTGAACGCAAGTT-
GCGCCCGAAGCCTTTTGGCCAAGGGCACGTCTGCCTGGGCGTCACGCATAGCGTCGCCCCTA
CC-T-CCCAACTCGTGTTTGG--ATATGTGT--G-GGGC-
GGAGAGTGGTCTCCCGTGTGCCT-CTT-GCTCACATGGATGGCCGAAATAAGGAGCTT---
GCGGCGG--TGAGCGCTGTGACGATTGGTGG-TTGGAT-GGGC-TCTT-GCCCTACAA---
AGCATC----TTGTCACGAAGTGCCA-CCCCGTGTT-G-GCTCGGAGGACTCGGTCTGG--
TCGATGTTATCCAGATTTGTGCGC--C-TTTTATTGTCCAC-CAT
</sequence>
<sequence>
<taxon idref="leucant552"/>
TCGAAACCTGC-ATAGCAG-AGCAACATGCG--AACTTGTCTTTT-
CAACCTTCCTTTTTCCGTGTGGCGGGTCAGACGA-CT------------CGTGCG-
TCC----GCC-TTGCG-GGATGAACAA---CTTTC-GGCGCGGGTTGCGCCAAGGA------
CCATGAACGTTAGGCCTC--GCC-----TCTTTGC-GCCGTTAGGCTGCGTTG-----AG-
GA-G--TC--CCATCATTGAA--AAATA-T-----------CAATTTAATGAC--
TCTTGACAAAGGATATCTCGGCTCTCGCATCGATGAAGAACG--TAGCGAAAT---
GCGATACTTGGTGTGAATTGCA-GAATCCTGTGAACCATTGA---
GTCTTTGAACGCAAGTT-
GCGCCCGAAGCCTTTTGGCCAAGGGCACGTCTGCCTGGGCGTCACGCATAGCGTCGCCCCTA
CC-T-CCCAACTCGTGTTTGG--ATGTGTGT--G-GGGC-
GGAGAGTGGTCTCCCGTGTGC---CTT-GCTCACATGGATGGCAGAAATAAGGAGCTT---
GCGGCGG--TGAGCGCCGTGATGATTGGTGG-TTGGAT-GGGC-TCTT-GCCCTACAA---
AGCATC----TTGTCATGAAGTGCCA-CCCCGTGTT-G-GCTCGGAGGACTCGRTCTGG--
TCGATGTTATCCAGATTTGTGCGC--C-TTTTATTGWCCAC-CAT
</sequence>
<sequence>
<taxon idref="nabievi887"/>
TCGAAACCTGC-ATAGCTG-AGCAACATGCG--AACTTGTCTTTT-
CAACCTTCC--TTTCCGTGTGGCGGGTCAGACGA-CT------------CGTGCT-
TCCTC-CGCC-TTGCG-GGATGAACAA---CTTTC-GGCGCGGGTTGCGCCAAGGA------
CCATGAACGTTAGGCCTC--GAC-----TCTTTGC-GCCGTTAGGCTGCGTTG-----AG-
GT-G--TC--CCATCATTGAA--AAATA-T-----------CATATTAATGAC--
TCTTGACAAAGGATATCTCGGCTCTCGCATCGATGAAGAACG--TAGCGAAAT---
GCGATACTTGGTGTGAATTGCA-GAATCCTGTGAACCATTGA---
GTCTTTGAACGCAAGTT-
GCGCCCGAAGCCTTTTGGCCAAGGGCACGTCTGCCTGGGCGTCACGCATAGCGTCGCCCCTA
CC-T-CCCAACTCGTGTTTGG--ATATGTGT--G-GGGC-
GGAGAGTGGTCTCCCGTGTGCCT-CTT-GCTCACATGGATGGCCGAAATAAGGAGCTT---
GCGGCGG--TGAGCGCTGTGACGATTGGTGG-TTGGAT-GGGC-TCTT-GCCCTACAA---

AGCATC----TTGTCACGAAGTGCCA-CCCCGTGTT-G-GCTCGGAGGACTCGGTCTGG--
TCGATGTTATCCAGATTTGTGCGC--C-TTTTATAGTCCAC-CAT
</sequence>
<sequence>
<taxon idref="sarytavi90"/>
TCGAAACCTGC-ATAGCAG-AGCAACATGCG--AACTTGTCATTT-
CAACCTTCC--TTTCCGTGTGGCGGGTCAGACGA-CT-------------TGTGCT-
TCCTC-CGCC-TTGCG-GGATGAACAA---CTTTC-GGCGCGGGTTGCGCCAAGGA------
CCATGAACGTTAGGCCTC--GCC-----TCTTTGC-GCCGTTAGGCTGCGTTG-----AG-
GA-G--TC--CCATCATTGAA--AAATA-T------------CAATTTAATGAC--
TCTTGACAAAGGATATCTCGGCTCTCGCATCGATGAAGAACG--TAGCGAAAT---
GCGATACTTGGTGTGAATTGCA-GAATCCTGTGAACCATTGA---
GTCTTTGAACGCAAGTT-
GCGCCCGAAGCCTTTTGGCCAAGGGCACGTCTGCCTGGGCGTCACGCATAGCGTCGCCCCTA
CC-T-CCCAACTCGTGTTTGG--ATATGTGT--G-GGGC-
GGAGAGTGGTCTCCCGTGTGC---CTT-GCTCACATGGATGGCCGAAATAAGGAGCTT---
GCGGCGG--TGAGCGCCGTGATGATTGGTGG-TTGGAT-GGGC-TCTT-GCCCTACAA---
AGCATC----TTGTCATGAAGTGCCA-CCCCGTGTT-G-GCTTGGAGGACTCGGTCTGG--
TCGATGTTATCCAGATTTGTGCGC--C-TTTTATTGTCCAC-CAT
</sequence>
<sequence>
<taxon idref="tricol21AN"/>
TCGAAACCTGC-ATAGCAG-AGCAACATGCG--AACTTGTCTTTT-
CAACCTTCC--TTTCCGTGTGGCGGGTCAGACGA-CT-------------CGTTCT-
TCCTC-CGCC-TTGCG-GGATGAACAA---CTTTC-GGCGCGGGTTGCGCCAAGGA------
CCATGAACGTTAGGCCTC--GCC-----TCTTTGC-GCCGTTAGGCCGCGTTG-----AG-
GA-G--TC--CCATCATTGAA--AAATA-T------------CAATTTAATGAC--
TCTTGACAAAGGATATCTCGGCTCTCGCATCGATGAAGAACG--TAGCGAAAT---
GCGATACTTGGTGTGAATTGCA-GAATCCTGTGAACCATTGA---
GTCTTTGAACGCAAGTT-
GCGCCCGAAGCCTTTTGGCCAAGGGCACGTCTGCCTGGGCGTCACGCATAGCGTCGCCCCTA
CC-T-CCCAACTCGTGTTTGG--ATATGTGT--G-GGGC-
GGAGAGTGGTCTCCCGTGTGG---CTT-GCTCACATGGATGGCCGAAATAAGGAGCTT---
GCGGCGG--TGAGGGCCGTGATGATTGGTGG-TTGGAT-GGGC-TCTT-GCCCTACAA---
AGCATC----TTGTCATGAAGTGCCA-CCCCGTGTT-G-GCTCGGAGGACTCGGTCTGG--
TCGATGTTATCCAGATTTGTGCGC--C-TTTTATTGTCCAC-CAT
</sequence>
<sequence>
<taxon idref="venustum99"/>
TCGAAACCTGC-ATAGCAG-AGCAACATGCG--AACTTGTCTTCT-
CAACCTTCC--TTTCCGTGTGGCGGGTCAGAAGA-CT-------------CGTGCT-
TCCTC-CGCC-TTGCG-GGATGAACAA---TTTTC-GGCGCGGGTTGCGCCAAGGA------
CCATGAACGTTAGGCGCT--GCC-----TCTTTGC-GCCGTTAGGCTGCGTTG-----AG-
GA-G--CC--CCATCATTGAA--AACTA-T------------CTATTGAATGAC--
TCTCGACAAAGGATATCTCGGCTCTCGCATCGATGAAGAACG--TAGCGAAAT---
GCGATACTTGGTGTGAATTGCA-GAATCCTGTGAACCATTGA---
TTCTTTGAACGCAAGTT-
GCGCCTGAAGCCTTTTGGCCAAGGGCACGTCTGCCTGGGCGTCACGCATCGCGTCGCCCCTA
CC-T-CCCAACTCGTGTTTGG--ATATGTGT--G-GGGC-
GGAGAGTGGCCTCCCGTGTGCCT-CTT-GCTCACATGGATGGCCGAAATAAGGAGCTT---
GTGGCAG--CGAGCGCTGTGATGATTGGTGG-TTGGRT-GGGCTTTTT-GCCTTACAA---
AGCATC----TTGTCACGAAGCGCCG-CCCCGTGTT-G-GCTTGGAGGACTCGACCTGG--
TCGATGTTGTCCCGATTTGTGTGC--C-TTTTATCGTCCAC-CAT
</sequence>
<sequence>
<taxon idref="ulicinum79"/>

TCGAAACCTGC-ATAGCAG-AGCAACATGCG--AACTTGTCTTTT-
CAACCTTCC--TTTCCGTGTGGCGGGTCAGACGA-CT------------CGTGCT-
TCCTC-CGCC-TCGCG-GGATGAACAA---CTTTC-GGCGCGGGTTGCGCCAAGGA------
CCATGAACGTTAGGCCTY--GCC-----TCTTTGC-GCCGTTAGGCTGCGTTG-----AG-
GA-G--TC--CCATCATTGAA--CAATA-T-----------CAATTTAATGAC--
TCTTGACAAAGGATATCTCGGCTCTCGCATCGATGAAGAACG--TAGCGAAAT---
GCGATACTTGGTGTGAATTGCA-GAATCCTGTGAACCATTGA---
GTCTTTGAACGCAAGTT-
GCGCCCGAAGCCTTTTGGCCAAGGGCACGTCTGCCTGGGCGTCACGCATAGCGTCGCCCCTA
CC-T-CCCAACTCGTGTTYGG--ATATGTGT--G-GGGC-
GGAGAGTGGTCTCCCGTGTGCCT-CTT-GCTCACATGGATGGCCGAAATAAGGAGCTT---
GCGGCGG--TGAGCGCCGTGATGATTGGTGG-TTGGAT-GGGC-TCTT-GCCCTACAA---
AGCATC----TTGTCACGAAGTGCCA-CCCCGTGTT-G-GCTCGGAGGACTCGGTCTGG--
TCGATGTTATCCAGATTTGTGCGC--C-TTTTATTGTCCAC-CAT
</sequence>
<sequence>
<taxon idref="wiedema346"/>
TCGAAACCTGC-ATAGCAG-AGCAACATGCG--AACTTGTCTTTT-
CAACCTTCC--TTTCCGTGTGGCGGGTCAGACGA-CT------------CGTGCT-
TCCTC-CGCC-TCGCG-GGATGAACAA---CTTTC-GGCGCGGGTTGCGCCAAGGA------
CCATGAACGTTAGGCCTT--GCC-----TCTTTGC-GCCGTTAGGCTGCGTTG-----AG-
GA-G--TC--CCATCATTGAA--CAATA-T-----------CAATTTAATGAC--
TCTTGACAAAGGATATCTTGGCTCTCGCATCGATGAAGAACG--TAGCGAAAT---
GCGATACTTGGTGTGAATTGCA-GAATCCTGTGAACCATTGA---
GTCTTTGAACGCAAGTT-
GCGCCCGAAGCCTTTTGGCCAAGGGCACGTCTGCCTGGGCGTCACGCATAGCGTCGCCCCTA
CC-T-CCCAACTCGTGTTTGG--ATATGTGT--G-GGGC-
GGAGAGTGGTCTCCCGTGTGCCT-CTT-GCTCACATGGATGGCCGAAATAAGGAGCTT---
GCGGCGG--TGAGCGTCGTGATGATTGGTGG-TTGGAT-GGGC-TCTT-GCCCTACAA---
AGCATC----TTGTCACGAAGTGCCA-CCCCGTGTT-G-GCTCGGAGGACTCGGTCTGG--
TCGATGTTATCCAGATTTGTGCGC--C-TTTTATTGTCCAC-CAT
</sequence>
<sequence>
<taxon idref="AcaAraxanu"/>
TCGAAACCTGC-ATAGCAG-AGCAACATGCG--AACTTGTCTTCT-
CAACCTTCC--TTTCCGTGTGGCGGGTCAGAAGA-CT------------CGTGCT-
TCCTC-CGCC-TTGCG-GGATGAACAA---TTTTC-GGCGCGGGTTGCGCCAAGGA------
CCATGAACGTTAGGCCTT--GCC-----TCTTTGC-GCCGTTAGGCTGCGTTG-----AG-
GA-G--CC--CCATCATTGAA--AACTA-T-----------CTATTGAATGAC--
TCTCGACAAAGGATATCTCGGCTCTCGCATCGATGAAGAACG--TAGCGAAAT---
GCGATACTTGGTGTGAATTGCA-GAATCCTGTGAACCATTGA---
TTCTTTGAACGCAAGTT-
GCGCCTGAAGCCTTTTGGCCAAGGGCACGTCTGCCTGGGCGTCACGCATCGCGTCGCCCCTA
CC-T-CCCAACTCGTGTTTGG--ATATGTGT--G-GGGC-
GGAGAGTGGCCTCCCGTGTGCCT-CTT-GCTCACATGGATGGCCGAAATAAGGAGCTT---
GTGGCAG--CGAGCGCTGTGATGATTGGTGG-TTGGAT-GGGC-TTTT-GCCCTACAA---
AGCACC----TTGTCATGAAGTGCCA-CCCCGTGTT-G-GCTTGCAGGACTCGACCTGG--
TCGATGTTATCCCGATTTGTGTGC--C-TTTTATCGTCCAC-CAT
</sequence>
<sequence>
<taxon idref="AcaFlabeum"/>
TCGAAACCTGC-ATAGCAG-AGCAACATGCG--AACTTGTCTTTT-
CAACCTTCC--TTTCCGTCTGGCGGGTCAGACGA-CT------------CGTGCT-
TCCTC-CGCC-TTACG-GGATGAACAA---CTTTC-GGCGCGGGTTGCGCCAAGGA------
CCATGAACGTTAGGCCTC--GCC-----TCTTTGC-GTTGTTAGGCTGCGTTG-----AG-
GA-G--TC--CCATCATTGAA--AAATA-T-----------CAATTTAATGAC--

TCTTGACAAAGGATATCTCGGCTCTCGCATCGATGAAGAACG--TAGCGAAAT---
GCGATACTTGGTGTGAATTGCA-GAATCCTGTGAACCATTGA---
GTCTTTGAACGCAAGTT-
GCGCCCGATGCCTTTTGGCCAAGGGCACGTCTGCCTGGGCGTCACGCATAGCGTCGCCCCTA
CC-T-CCCAACTCGTGTTTGG--ATATGTGT--G-GGGC-
GGAGAGTGGTCTCCCGTGTGC---CTT-GCTCACATGGATGGCCGAAATAAGGAGCTT---
GCGGCGG--TGAGCGCCGTGATGATTGGTGG-TTGGAT-GGGC-TCTT-GCCCTACAA---
CGCATC----
CTGTCATGAATCGCCA--

</sequence>
<sequence>
<taxon idref="Balohani75"/>
TCGAAACCTGC-ATAGCAG-AGCAACATGCG--AACTTGTCTTTT-
CAACCTTCC--TTTCCGTGTGGCGGGTCGGACGA-CT-------------CGTGCT-
TCCTC-CGCC-TTGCG-GGATGAACAA---CTTTC-GGCGCGGGTTGCGCCAAGGA------
CCATGAACGTTATGCCTC--GCC-----TCTTTGC-GCCGTTAGGCTGCGTTG-----AG-
GA-G--TC--ACATCATTGAA--AAATA-T------------CAATTTAATGAC--
TCTTGACAAAGGATATCTCGGCTCTCGCATCGATGAAGAACG--TAGCGAAAT---
GCGATACTTGGTGTGAATTGCA-GAATCCTGTGAACCATTGA---
NTCTTTGAACGCAAGTT-
GCGCCCGAAGCCTTTTGGCCAAGGGCACGTCTGCCTGGGTGTCACGCATAGCGTCGCCCCTA
CC-T-CCCAACTCGTGTTTGG--ATATGTGT--G-GGGC-
GGAGAGTGGTCTCCCGTGTGC---CTT-GCCCACATGGATGGCCGAAATAAGGAGCTT---
GCGGCGG--TGAGCGCCGTGATGATTGGTGG-TTGGATGGGGC-TCTT-GCCCTGCAA---
AGCATC----TTGTCATGAAGTGCCACCCCCGTGTT-G-GCTCGGAGGACTCGGTCTGG--
TCGATGTTATCCAGATTTGTGCGC--C-TTTTATTGTCCAC-CAT
</sequence>
<sequence>
<taxon idref="Gonitilcum"/>
TCGAAACCTGC-ATAGCAG-AGCCACTTGTG--CACTT--TTTTT-
CAACCCCTCC-TTTCCGTGCGGTTGGTCGGTAGC-CT-------------CGTGCT-
TCCTC-CACC-CCACG-GGATGAACAA----TTTCCGGCGCGGGTTGCGCCAAGGA------
TCATGAACGTTCGGTCTT--GCC-----TCTCTGC-ACCGTTAGGCTGCGTTG-----AG-
GCA---TC--CCATCGTTGAA--AATTA-T------------CAATCGAATGAC--
TCTCGGCAACGGATATCTCGGCTCTCGCATCGATGAAGAACG--TAGCGAAAT---
GCGATACTTGGTGTGAATTGCA-GAATCCCGTGAACCATCGA---
GTCTTTGAACGCAAGTT-
GCGCCCGAAGCCTTCCGGCCAAGGGCACGTCTGCCTGGGCGTCACGCATCGCGTCGCCCCCC
ACCT-TCCAA------TTTGG--ATGTGTGC--G-GGGC-
GGAGAGTGGCCTCCCGTGTGCCT-CTT-GCCCACACGGATGGCCGAAACAAGGAGCTT---
GCGGCGG--TGAGCGCCGTGACAATCGGTGG-TTGGAT-GGGC-ACTT-GTCCTACGA---
AGCATC----TTGTCACGAAGCGCCA-CCTTGTGTT---GCTCGGAGGACTCGAATCGG--
TCGATGTTATTGCGATCTGATCGC--C-TCTCATCGATCAC-CAT
</sequence>
<sequence>
<taxon idref="Gonispecoi"/>
TCGAAACCTGC-ATAGCAG-AGCCACCTGTG--AACTT--TTTTT-
CAACCCCTCC-TTTCCGTGCGGTTGGTCGGTAGC-CT-------------TGTGCT-
CCCTC-CGCC-CCCACGGGATGAACAA----TTTCCGGCGCGGGTTGCGCCAAGGA------
TCATGAACGTTCGGTCTT--GCC-----TCTCCGC-ACCGTTAGGCTGCGTYG-----AG-
GCA---TC--CCATCGTTGAA--AATTA-T------------CAATCGAATGAC--
TCTCGGCAACGGATATCTCGGCTCTCGCATCGATGAAGAACG--TAGCGAAAT---
GCGATACTTGGTGTGAATTGCA-GAATCCCGTGAACCATCGA---
GTCTTTGAACGCAAGTT-
GCGCCCGAAGCCTTCCGGCCAAGGGCACGTCTGCCTGGGCGTCACGCATCGCGTCGCCCCC-

ACCT-CCCAA------TCTGG--ATGTGTGC--G-GGGC-
GGAGAGTGGCCTCCCGTGTGCCT-CTT-GCCCACACGGATGGCCGAAACAAGGAGCTT---
GCGGCGG--TGAGCGCCGTGACATTCGGTGG-TTGGAT-GGGC-ACTT-GTCCTACGA---
AGCATC----TTGTCACGAAGCGCCACCCTGTGTTT---GCTCGGAGGACTCGAATCGG--
TCGATGTTATTGCGATCTGATCGC--C-TATCATCGATCAC-CAT
</sequence>
</alignment>

<!-- The sequence alignment (each sequence refers to a taxon above). -->
<!-- ntax=156 nchar=1309
-->
<alignment id="alignment2" dataType="nucleotide">
<sequence>
<taxon idref="Plumeuropa"/>
A-------------------------------------
TCCTGAATGCTTCATTGTGTGGCTGAGATAT------ATA-TCT---------------
CCATGGAGT-ACATAC-TA-AGTTATTCTAGACTAT--CTA-
ATGAC----------------TATATAAT---------------
AAAGTAGACTCATAGTGGCTAGTGCCTTCTTCCGGAATGAGAAAGTGAATTTCACTATCGAG
TCTAGGAT-AAAT--------------------
GATCTTTTTAAAATATGACTTCACTGAATCAAGCCAACCCTAA-
TTTCTTTTCTTTTATTAAATTGCTACTC------------AGCAGAACAAAA-
CTCACTTGATACATGTACCTAACAATTCGACATAGAT-
CGGAGATATTTCA-------------------
TTCAAAAATGGGATGAAGAAGTTCGATTTGTCCCCTG----TCTTAGATG-
AAAAAAAAAA---GAATTTCCGAAACTCTCTTCATCCTTC----TTACC------
AGTTTTATCATCCCGGATTTCTTCTTTTTTC-------
CTTTTTTGGTATAGATATAGATATTTCTTTTTTTCTAGATTCTATTTCTACTAGATTCTA-
T---CTAT-CTATATTTTCTTTTTTT----ATT-CAAAAGTTAGTTATATAAC--------
AAAAAAAGAAA-AA-----AAAAATACACACAAAAT-----TTTTAT--
GTCATTTTT-----
CTATTCGAAACTCTCTTCGAATAATTCTTCCAATTCATAAATAAGATCTCATTTACGAAATT
TCAATTTAATAAGGATATATTCTATTAAATTATAGAATCGAAATGGACTTCAATCAAAAAAT
TCGAAGAATTTCTAGA--

</sequence>
<sequence>
<taxon idref="DyerSoconu"/>
A-------------------------------------
TCCTGAATGCTTCATTGTGTGGCTGAGATAT------ATA-TCT---------------
CCATGGAGT-ACATAC-TA-
AGTTATTATAGGGATAGACTATATAATGAC-------------
TATATAAT---------------
AAAGTAGACTCATAGTGGCTAGTGCCTTCTTCCGGAATGAGAAAGTGAATTTCACTATCGAG
TCTAGGAT-AAGT-------TTATT--------
GATCTTTTTAAAATATGACTTCACTGAATCAAGCCGATCCCAA-TTTCTTTT-----
ATTAAATTGCTACTC------------AGCAGAACAAAA-
CTCACTTGATACATGTACCTAGCAATTCGACATAGAT-
CGGAGATATTTCA-------------------
TTCAAAAATGGGATGGAGAAGTTGGATTTGTCCCCTGAAATTCTTAGATG-

AAAAAATAAA---GAATTTCCGAAACTCTCTTGATCCTTC----TTACC------
AGCTTTATCATCCCAGATTTCTTATTTTTTC-------ATTTT------
CTGGTATAGATCTTTCTTTTTTTCTAGATTCTATTTATACTAGATTCTA-T---CTAT-
ATATATTTTCTTTTTTT----ATT-CAAAAGTTAGTTATATAAC--------
AAATAAAATAA-AT-----AGAAAAAAAAACACACAA----TTTTTTT-
GATTTCATTTTT--
CTATTCGAAACTCTCTTAGAATAATTCTTACAATTAGTAAATAATATATCATTTACGAAATT
TCAATTTAATAATGATATATTCTATTAAATTTAGAATCTAAATGGACTTCAATCAAAATAAT
TAGAATAAATTCTAGA--

</sequence>
<sequence>
<taxon idref="Limvulgare"/>

T--

</sequence>
<sequence>
<taxon idref="Limmeyerii"/>
T-------------------------------------
TCCTGACTGTTTCATTATGTGGATAAGATAT------ATA-T-----------------
GCATGTAGT-ACATAC-TA-
AGTTATTATAGACTATAACTATATAAGTATA----------------
TAAG---------------
AAAGTAGACTCATACTGGCTAGTGACTTTTTCCGGAATGAGAAGGTTGATTTAACTATCGAG
TCTAGGATCAAATT----ATTTATT--------
GATCTTTTTAAAATACGACTTCAATACATCAAGCCGACCCTAA-TTTCTTTT-----
AGTAAATTGATACCC------------ATCAGAAAAAAA-
TTCACTTGATACATGTACCTACCAATTCGACATAGATTCCAAGATATTTCA-----------
--------TTTTAAAATGGGATGGAGAAGTTCGATTTGGCCACAGAAATTCTTAGAT--
AAAAAAAA-----GAATTTCCGAAACTCCCTTAAGCCTTC----TTACT------

AGCTTTATCATCCCTGATTTCTTCTTGATTC-------ATTTTATTTT-
CTAGTATAGATATTTCGTTTTT-----------------CTAGATTCTA-T---CTTT-
CTTTTTTTTTTTT--------ATT-CAAAAGTGAGTTATATAAC--------
AAAGAAAAAAA-TG-----AGTTTCTTTCTTTTTTTTT--
CTTTTGCCTTTTCTTTTTT----
ATTTTCAAATAAAA--

TGGATTTATATATTGAATCGAGTGAAGGAAGAATTCAAGGAATGCGGAATCAAAAAAAA---
-----TCATGACAGGCGGAAAGATCCTTTGGGTCGA------
ATTCGCTTCTTACAAAATATTGACAATTTCAAAAAA-
CTGATCATACTATGATCATAGTATGATGGCGGTTGGACACGTATGCCCCCATCGTCTAGTGG
TTCAGGACATCTCTCTTTCAAGGAGGCAGCGGGGATTCGACTTCCCCTGGGGG-
TAGGTTACTACA-AAAGAAAGTTAATCGTGCATTATCAATAA-------GCCTAAA----
ATGGAATTCATTCTTTCTGGGTCGATGCCCGAGGGGTTAATGGGGACGGACTGTAAATTCGT
TGGCAATATGTCTACGC
</sequence>
<sequence>
<taxon idref="Limrenifoe"/>
T-------------------------------------
TCCTGACTGTTTCATTATGTGGATAAGATAT------ATA-T-----------------
GCATGTAGT-ACATAC-TA-
AGTTATTATAGACTATAACTATATAAGTATA----------------
TAAG---------------
AAAGTAGACTCATACTGACTAGTGACTTCTTCCGGAATGAGAAGGTTAATTTAACTATCGAG
TCTAGGATCAAATT----ATTTATTTATT----
GATTTTTTAAAAATACAACTTCAATACATCAAGCCGACCCGAA-TTTCGTTT-----
AGTAAATTGATACCC------------ATCAGAAAAAAT-
TTCACTTGATACATGTACCTACCAATTCGACATAGATTCCAAGATATTTCA-----------
--------TTTTAAAATGGGATGGAGAAGTTCGATTTGGCCACATAAATTCTTAGAT--
AAAAAAAAAAAAAGAATTTCCGAAACTCCCTTAATCCTTC----TTACT------
AGCTTTATCATCCCTGATTTCTTCTTGATTC-------ATTTT------
CTGGTATAGATATTTAGTTTTT-----------------CTAGATTCTA-T---CTTT-
CTTTTTTTTTTTTT-------ATT-
C---

</sequence>
<sequence>
<taxon idref="Limsuffruo"/>
T-------------------------------------
TCCTGACTGTTTCATTATGTGGATAAGATAT------ATA-T-----------------
GCATGTAGT-ACATAC-TA-
AGTTATTATAGACTATAACTATATAAGTATATAAGAAAATAAGTATATAAG-----------
----
AAAGTAGACTCATACTGGCTAGTGACTGTTTCCGGAATGAGAAGGTTGATTTAACTATCGAG
TCTAGGATCAAATT----ATTTATT--------
GATCTTTTTAAAATATGACTTGAATACATCAAGCCGACCCTAC-TTTCTTTT-----

AGTAAATTGATACCC------------ATCAGAAAAAAA-
TTCACTTGATACATGTACCTACCAATTCGACATAGATTCCAAGATATTTCA-----------
--------TTTTAAAATGGGATGGAGAAGTTCGATTTGGCCACAGAAATTCTTAGAT--
AAAAAAAAAA---GAATTTCCGAAACTCCCTTAATCCTTC----TTACT------
AGCCTTATCATCCCTGATTTCTTCTTGATTC-------ATTTT------
CTGGTATAGATATTTCGTTTTT-----------------CTAGATTCTA-T---CTTT-
CTTTTTTTTTTT---------ATT-CAAAAGTGAGTTATATAAC--------
AAAGAAAAAAA-TG-----
AGTTTCTTTCTTTTTTTTTTTCTTTTGCCTTTTCTTTTTT----------------------
----

</sequence>
<sequence>
<taxon idref="Limcarnos1"/>
T------------------
TAAAGGCCTTTTAGTAAATTCCTGACTGTTTCATTATGTGGATAAGATAT------ATA-
TAT---------------GCATGTAGT-ACATAC-TA-
AGTTATTATAGACTATAACTATATAAGTATATAAGAAAATAAGTATATAAG-----------
----
AAAGTAGACTCATACTGGCTAGTGACTTTTTCCGGAATGAGAAGGTTGATTTAACTATCGAG
TCTAGGATCAAATT----ATTTATT--------
GATCTTTTTAAAATATGACTTGAATACATCAAGCCGACCCTAC-TTTCTTTT-----
AGTAAATTGATACCC------------ATCAGAAAAAAA-
TTCACTTGATACATGTACCTACCAATTCGACATAGATTCCAAGATATTTCATAGATTCCAAG
ATATTTCATTTTAAAATGGGATGGAGAAGTTCGATTTGGCCACAGAAATTCTTAGAT--
AAAAAAAAA----GAATTTCCGAAACTCCCTTAATCCTTC----TTACT------
AGCCTTATCATCCCTGATTTCTTCTTGATTC-------ATTTT------
CTGGTATAGATATTTCGTTTTT-----------------CTAGATTCTA-T---CTTT-
CTTTTTTTT------------ATT-CAAAAGTGAGTTATATAAC--------
AAAGAAAAAAA-TG-----
AGTTTCTTTCTTTTTTTTTTTCTTTTGCCTTTTCTTTTTT----
ATTTGAAAATAAAA--

CGGATTTATATATTGAATCGAGTGGAGGAAGAATTCAAGGAATGCGGAATCAAAAAGAA---
-----TAATGACAGGCGGAAAGATCCTTTGGGTCGA------
ATTCGATTCTTACAAAATATTGACAATTTCAAAAAA-
CTGATCATACTATGATCATAGTATGATGGCGGTTGGACACGTATGCCCCCATCGTCTAGTGG
TTCAGGACATCTCTCTTTCAAGGAGGCAGCGGGGATTCGACTTCCCCTGGGGG-
TAGGTTACTACA-AAAGAAAGTTAATCGTGCATTATCAATAA-------GCCTAAA----
TTTGAATTCATTCTTCCTGGGTCGATGCCCGAGCGGTTAATGGGGACGGACTGTAAATTCGT
TGGCAATATGTCTACGC
</sequence>
<sequence>
<taxon idref="Limotoleps"/>

T--

</sequence>
<sequence>
<taxon idref="Limiranicm"/>

TCCTGAATGTTTCATTATGTGGATAAGATAT------ATA-TAT---------------
GCATGTAGT-ACATAC-TA-
AGTTATTATAGACTATAACTATATAAGTATATAAGAAAATAAGTATATAAG-----------
----
AAAGTAGACTCATACTGGCTAGTGACTTTTTCCGGAATGAGAAGGTTGATTTAACTATCGAG
TCTAGGATCAAATT----ATTTTTT--------
GATCTTTTTAAAATATGACTTGAATACATCAAGCCGACCCTAC-TTTCTTTT-----
AGTAAATTGATACCC------------ATCAGAAAAAAA-
TTCACTTGATACATGTACCTACCAATTCGACATAGATTCCAAGATATTTCA-----------
--------TTTTAAAATGGGATGGAGAAGTTCGATTTGGCCACAGAAATTCTTAGAT--
AAAAAAAAA----GAATTTCCGAAACTCCCTTAATCCTTC----TTACT------
AGCCTTATCATCCCTGATTTCTTCTTGATTC-------ATTTT------
CTGGTATAGAGATTTCGTTTTT-----------------CTAGATTCTA-T---CTTT-
CTTTTTTTTTTTTT-------ATT-
CAAA--

</sequence>
<sequence>
<taxon idref="Limnudummm"/>
T------------------
TAAAGGCCTTTTAGTAAATCCCTGACTGTTTCATTATGTGGATAAGATAT------ATA-
T-----------------GCATGTAGT-ACATAC-TA-
AGTTATTATAGACTATAACTATATAAGTATA----------------
TAAG---------------

AAAGTAGACTCATACTGACTAGTGACTTCTTCCGGAATGAGAAGGTTTATTTAACTATCGAG
TCTAGGATCAAATT----ATTTATT--------
GATCTTTTTAAAATACGACTTCAATACATCAAGCCGACCCTAA-TTTCTTTT-----
AGTAAATTGATACCC------------
ATCAGAAAAAAAATTCACTTGATACATGTACCTACCAATTCGACATAGATTCCAAGATATTT
CA-------------------
TTTTCAAATGGGATGGAGAAGTTCGATTTGGCCACAGAAATTCTTAGATC-
AAAAAAA------GAATTTCCGAAACTCCCTTAATCCTTCCTTCTTACT------
AGCTTTATCATCCCTGATTTCTTCTTGATTC-------ATTTT------
CTGGTATAGATATTTCGTTTTT-----------------CTAGATTCTA-TA--CTTT-
CTTTTTTTTTTT--
--------------------------------TTT-CCTTTTCTTTTTT----
ATTTGAAAATAAAA--

TGGATTTTTATATTGAATCGAGTGGAGGAAGAATTCAAGGAATGCATAATCAAAAAGAA---
-----TCATGACAGGCGGAAAGATCCTTTGGGTCGA------
ATTCGATTCTTACAGAATATTGACAATTTCAAAAAA-
CGGATCATACTATGATCATAGTATGATGGCGGTTGGACACGTATGCCCCCATCGTCTAGTGG
TTCAGGACATCTCTCTTTCAAGGAGGCAGCGGGGATTCGACTTCCCCTGGGGG-
TAGGTTACTACA-AAAGAAAGTTAATCGTGCATTATCAATAA-------GCCTAAA----
TTTGAATTCATTCTTCCTGGGTCGATGCCCGAGCGGTTAATGGGGACGGACTGTAAATTCGT
TGGCAATATGTCTACGC
</sequence>
<sequence>
<taxon idref="Limnarbone"/>

T--

</sequence>
<sequence>
<taxon idref="Limaxillar"/>
T------------------
TAAAGGCCTTTTAGTAAATTCCTGACAGTTTCATTATGTGGATAAGATAT------ATA-
T-----------------GCATGTAGT-ACATAC-TA-
AGTTATTATAGACTATAATTATATAAGTATA----------------

TAAG--------------
AAAGTAGACTCATACTGGCTAGTGACTTCTTCCGGAATGAGAAGGTTGATTTAACTATCGAG
TCTAGGATCAAATT----TTTTATT--------
GATCTTTTTAAAATACGACTTCAATACATCAAGCCGACCCTAA-TTTCTTTT-----
AGTAAATTGATACCC------------ATCAGAACAAAA-
TTCACTTGATACATGTACCTACCAATTCGACATAGATTCCAAGATATTTCA-----------
--------TTTTAAAATGGGATGGAGAAGTTCGATTTGGCCACAGAAATTCTTAGAT--
AAAAAAAA-----GAATTTCCAAAACTCCCTTAATCCTTC----TTACT------
AGCTTTATCATCCCTGATTTCTTCTTGATTC-------ATTTT------
CTGGTATAGATATTTCGTTTTT-----------------ATAGATTCTA-T---CTTT-
CTTTTTTTTTTTT---------ATT-CAAAAGTGAGTTATATAAC--------
AAAGAAAAAAAATG-----
AGTTTCTTTC--

-------------GGAAAGATCCTTTGGGTCGA------
ATTCGATTCTTACATAATATTGACAATTTCAAAAAA-
CTGATCATACTATGATCATAGTATGATGGCGGTTGGACACGTATGCCCCCATCGTCTAGTGG
TTCAGGACATCTCTCTTTCAAGGAGGCAGCGGGGATTCGACTTCCCCTGGGGG-
TAGGTTACTACA-AAAGAAAGTTAATCGTGCATTATCAATAA-------
GCCTAAA---

</sequence>
<sequence>
<taxon idref="Limsogdiam"/>

TCCTGACTGTTTCATTATGTGGATAAGATAT------ATA-T-----------------
GCATGTAGT-ACATAC-TA-
AGTTATTATAGACTATAACTATATAAGTATA---------------
TAAG--------------
AAAGTAGACTCATACTGACTAGTGACTTTTTCCGGAATGAGAAGGTTGATTTAACTATCGAG
TCTAGGATCAAATT----ATTTATT--------
GATCTTTTTAAAATACGACTTCAATACATCAAGCCGACCCTAA-TTTCTTTT-----
AGTAAATTGATACCC------------ATCAGAAAAAAA-
TTCACTTGATACATGTACCTACCAATTCGACATAGATTCCAAGATATTTCA-----------
--------TTTTAAAATGGGATGGAGAAGTTCGATTTGGCCACAGAAATTCTTAGAT--
AAAAAAAAA----GAATTTCCGAAACTCCCTTAATCCTTC----TTACT------
AGCTTTATCATTCCTGATTTCTTCTTGATTC-------ATTTT------
CTGGTATAGATATTTCGTTTTT-----------------CTAGATTCTA-T---CTTT-
CTTTTTTTTT-----------ATT-CAAAAGTGAGTTATATAAC--------
AAAGAAAAAAA-TG-----AGTTTCTTTCTTTTTTTTT--
CTTTTGCCTTTTCTTTTTT----
ATTTGAAAAAAAAAAAA--

TGGATTTATATATTGAATCGAGTGAAGGAAGAATTCAAGGAATGCGGAATCAAAAAAAA---
-----TCATGACAGGCGGAAAGATCCTTTGGGTCGA------
ATTCGATTCTTACAAAATATTGACAATTTCAAAAAA-
CTGATCATACTATGATCATAGTATGATGGCGGTTGGACACGTATGCCCCCATCGTCTAGTGG
TTCAGGACATCTCTCTTTCAAGGAGGCAGCGGGGATTCGACTTCCCCTGGGGG-
TAGGTTACTACA-AAAGAAAGTTAATCGTGCATTATCAATAA-------GCCTAAA----
ATGGAATTCATTCTTTCTGGGTCGATGCCCGAGGGGTTAATGGGGACGGACTGTAAATTCGT
TGGCAATATGTCTACGC
</sequence>
<sequence>

<taxon idref="Limgmelini"/>

TCCTGACTGTTTCATTATGTGGATAAGATAT------ATA-T-----------------
GCATGTAGT-ACATAC-TA-
AGTTATTATAGACTATAACTATATAAGTATA----------------
TAAG---------------
AAAGTAGACTCATACTGGCTAGTGACTTTTTCCGGAATGAGAAGGTTGATTTAACTATCGAG
TCTAGGATCAAATT----ATTTATT--------
GATCTTTTTAAAATACGACTTCAATACATCAAGCCGACCCTAA-TTTCTTTT-----
AGTAAATTGATACCC------------ATCAGAAAAAAA-
TTCACTTGATACATGTACCTACCAATTCGACATAGATTCCAAGATATTTCA-----------
--------TTTTAAAATGGGATGGAGAAGTTCGATTTGGCCACAGAAATTCTTAGAT--
AAAAAAAA-----GAATTTCCGAAACTCCCTTAATCCTTC----TTACT------
AGCTTTATCATCCCTGATTTCTTCTTGATTC-------ATTTTATTTT-
CTAGTATAGATATTTCGTTTTT-----------------CTAGATTCTA-T---CTTT-
CTTTTTTTTTTTTTT------ATTTCAAAAGTGAGTTATATAAC--------
AAAGAAAAAAA-TG-----
AGTTTCTTTCTTTTTTTTTTTCTTTTGCCTTTTCTTTTTT----------------------
----

</sequence>
<sequence>
<taxon idref="Psysuworou"/>

T--

</sequence>
<sequence>
<taxon idref="Psyspicata"/>

T-------------------------------------
TCCTGAATGCTTCATTATGTGGATAAGATAT------ATA-T-----------------
GCATGTAGT-ACATAC-TA-
AGTTATTATAGACTATAACTATATAAGTATA-------------------------------
----
AAAGTAGACTCATACTGGCTAGTGGCTTCTTCCGGAATGGTAAAGTTGATTTAACTATCGAG
TCTAGCAT-A--------ATTTTT---------
TTTCTTTTAAAAATAGGACTTCAATCCATCAAGCCGACC-TAAATTTTTTTA--------
AAATTAATACCC------------ATCAGAAAAAAA-
TTCACTTGATACATGTACCTACCAATTCGACATAGATTCCAAGATATTTCA-----------
--------TTCAAAAATGGATGGAGAAGTTAGATTTGTCCACAGAAATTCTTAG----
AAAAAAAAA---

------------GCCACTCATTTTTT----
CTATTCGAG---

TGGAGGAAGAATTCAAGGAATGCGGAACCAAAAAAATCTAGGAAATCATGAAAGGCGGAAAG
ATCCTTTGGGTCGA------ATTCGATTCTTACAGAATATTGACAATTTCAAAAAA-
CTGATCATACTATGATCATAGTATGATGGCGGTTGGACACGTATGCCCCCATCGTCTAGTGG
TTCAGGACATCTCTCTTTCAAGGAGGCAGCGGGGATTCGAATTCCCCTGGGGG-
TAGGTTACTACA-AAAGAAAGTTAATCGTGCATTATCAATAA-------GCCTAAA----
ATGGAATTCATTCTTTCTGGGTCGATGCCCGAGGGGTTAATGGGGACGGACTGTAAATTCGT
TGGCAATATGTCTACGC
</sequence>
<sequence>
<taxon idref="Psybeludsh"/>
T-------------------------------------
TCCTGAATGCTTCATTATGTGGATAAGATAT------ATA-T-----------------
GCATGTAGT-ACATAC-TA-
AGTTATTATAGACTATAACTATATAAGTATA-------------------------------
----
AAAGTAGACTCATACTGGCTAGTGGCTTCTTCCGGAATGGTAAAGTTGATTTAACTATCGAG
TCTAGCAT-A--------ATTTTTTT-------
TTTCTTTTTAAAATAGGACTTCAATCCATCAAGCCGACC-TAAATTTTTTTT-------
TAA-TTAATACCC------------ATCAGAAAAAAA-
TTCACTTGATACATGTACCTACCAATTCGACATAGATTCCAAGATATTTCA-----------
--------TTAAAAAATGGATGGAGAAGTTAGATTTGTCCACAGAAATTCTTAG----
AAAAAAA---

------------GCCACTCATTTTTT----
CTATTCGAG---

TGGAGGAAGAATTCAAGGAATGCGG-----
AAAAAATCTAGGAAATCATGAAAGGCGGAAAGATCCTTTGGGTCGA------
ATTCGATTCTTACAGAATATTGACAATTTCAAAAAA-
CTGATCATACTATGATCATAGTATGATGGCGGTTGGACACGTATGCCCCCATCGTCTAGTGG
TTCAGGACATCTCTCTTTCAAGGAGGCAGCGGGGATTCGAATTCCCCTGGGGG-
TAGGTTACTACA-AAAGAAAGTTAATCGTGCATTATCAATAA-------GCCTAAA----
ATGGAATTCATTCTTTCTGGGTCGATGCCCGAGGGGTTAATGGGGACGGACTGTAAATTCGT
TGGCAATATGTCTACGC

</sequence>
<sequence>
<taxon idref="Psyleptosh"/>
T-------------------------------------
TCCTGAATGCTTCATTATGTGGATAAGATAT------ATA-T-----------------
GCATGTAGT-ACATAC-TA-
AGTTATTATAGACTATAACTATATAAGTATA-------------------------------
----
AAAGTAGACTCATACTGGCTAGTGGCTTCTTCCGGAATGGTAAAGTTGATTTAACTATCGAG
TCTAGCAT-A--------ATTTT----------
TTTCTTTTAAAAATAGGACTTCAATCCATCAAGCCGACC-
TAAATTTTTTT------------TTAATACCC------------ATCAGAAAAAAA-
TTCACTTGATACATGTACCTACCAATTCGACATAGATTCCAAGATATTTCA-----------
--------TTAAAAAATGGGATGGAGAAGTTAGATTTGTCCACAGAAATTCTTAG----
AAAAAAAAA---

------------GCCACTCATTTTTT----
CTATTCGAG---

TGGAGGAAGAATTCAAGGAATGCGGAACCCCAAAAATCTAGGAAATCATGAAAGGCGGAAAG
ATTCTTTGGGTCGA------ATTCGATTCTTACAGAATATTGACAATTTCAAAAAA-
CTGATCATACTATGATCATAGTATGATGGCGGTTGGACACGTATGCCCCCATCGTCTAGTGG
TTCAGGACATCTCTCTTTCAAGGAGGCAGCGGGGATTCGAATTCCCCTGGGGG-
TAGGTTACTACA-AAAGAAAGTTAATCGTGCATTATCAATAA-------GCCTAAA----
ATGGAATTCATTCTTTCTGGGTCGATGCCCGAGGGGTTAATGGGGACGGACTGTAAATTCGT
TGGCAATATGTCTACGC
</sequence>
<sequence>
<taxon idref="Armhirtaaa"/>

T--

</sequence>

<sequence>
<taxon idref="Armeriamar"/>
T-------------------------------------
TCCTGAATGCTTCATTATGTGGATAAGATAT------ATA-T-----------------
GCATGTAGT-ACATAC-TA-
AGTTATTATAGACTATAACTATATAAGTATA----------------
TAAG---------------
AAAGTAGACTCATACTGGCTAGTGGCTTCTTCCGGAATGATAAAGTTGATTTCACTATCGAG
TCTAGGAT-AAAATC---ATTTATT--------
GATCTTTTTAAAATAAGACTTCAATACATCAAGCCGACCCTAA-TTTATTTG-----
ATTAAATTGATACCC------------ATCAGCAAAAAA-
TTAACTTGATACATGTACCTACCAATTTGACATAGATTCCAAGATATTTCA-----------
--------TTAAAAAATGGATGGAGCAGTTAGATTTGTCCACATAAATTCTTAG----
AAAAAAA---

------------GACACTCATTTTTTT---
CTATTCGAG---

TTGAGGAAGAATTCAAGGAATGCGGAACCAAAAAAATCTATGAAATCATCAAAGGCGGAAAG
ATCCTTTGGGTCGA------ATTCGATTCTTACAGAATATTGACAATTTTAAAAAA-
CTGATCATACTATGATCATAGTATGATGGCGGTTGGACACGTATGCCCCCATCGTCTAGTGG
TTCAGGACATCTCTCTTTCAAGGAGGCAGCGGGGATTCGACTTCCCCTGGGGG-
TAGGTTACTACA-AAAGAAAGTGAATCGTGCATTATCAATAA-------
GCCTAAATAAAATGGAATTCATTCTTTCTGGGTCGATGCCCGAGGGGTTAATGGGGACGGAC
TGTAAATTCGTTGGCAATATGTCTACGC
</sequence>
<sequence>
<taxon idref="Armeriawel"/>
T-------------------------------------
TCCTGAATGCTTCATTATGTGGATAAGATAT------ATA-T-----------------
GCATGTAGT-ACATAC-TA-
AGTTATTATAGACTATAACTATATAAGTATA----------------
TAAG---------------
AAAGTAGACTCATACTGGCTAGTGGCTTCTTCCGGAATGATAAAGTTGATTTCACTATCGAG
TCTAGGAT-AAAATC---ATTTATT--------
GATCTTTTTAAAATAAGACTTCAATACATCAAGCCGACCCTAA-TTTATTTG-----
ATTAAATTGATACCC------------ATCAGCAAAAAA-
TTAACTTGATACATGTACCTACCAATTTGACATAGATTCCAAGATATTTCA-----------
--------TTAAAAAATGGATGGAGCAGTTAGATTTGTCCACATAAATTCTTAG----
AAAAAAA---

------------GACACTCATTTTTTT---
CTATTCGAG---

TTGAGGAAGAATTCAAGGAATGCGGAACCAAAAAAATCTATGAAATCATCAAAGGCGGAAAG
ATCCTTTGGGTCGA------ATTCGATTCTTACAGAATATTGACAATTTTAAAAAA-
CTGATCATACTATGATCATAGTATGATGGCGGTTGGACACGTATGCCCCCATCGTCTAGTGG
TTCAGGACATCTCTCTTTCAAGGAGGCAGCGGGGATTCGACTTCCCCTGGGGG-
TAGGTTACTACA-AAAGAAAGTGAATCGTGCATTATCAATAA-------
GCCTAAATAAAATGGAATTCATTCATTCTGGGTCGATGCCCGAGGGGTTAATGGGGACGGAC

TGTAAATTCATTGGCAATATGTCTACGC
</sequence>
<sequence>
<taxon idref="Armeriamad"/>
T-------------------------------------
TCCTGAATGCTTCATTATGTGGATAAGATAT------ATA-T-----------------
GCATGTAGT-ACATAC-TA-
AGTTATTATAGACTATAACTATATAAGTATA----------------
TAAG---------------
AAAGTAGACTCATACTGGCTAGTGGCTTCTTCCGGAATGATAAAGTTGATTTCACTATCGAG
TCTAGGAT-AAAATC---ATTTATT--------
GATCTTTTTAAAATAAGACTTCAATACATCAAGCCGACCCTAA-TTTATTTG-----
ATTAAATTGATACCC------------ATCAGCAAAAAA-
TTAACTTGATACATGTACCTACCAATTTGACATAGATTCCAAGATATTTCA-----------
--------TTAAAAAATGGATGGAGCAGTTAGATTTGTCCACATAAATTCTTAG----
AAAAAAA---

------------GACACTCATTTTTTT---
CTATTCGAG---

TTGAGGAAGAATTCAAGGAATGCGGAACCAAAAAAATCTATGAAATCATCAAAGGCGGAAAG
ATCCTTTGGGTCGA------ATTCGATTCTTACAGAATATTGACAATTTTAAAAAA-
CTGATCATACTATGATCATAGTATGATGGCGGTTGGACACGTATGCCCCCATCGTCTAGTGG
TTCAGGACATCTCTCTTTCAAGGAGGCAGCGGGGATTCGACTTCCCCTGGGGG-
TAGGTTACTACA-AAAGAAAGTGAATCGTGCATTATCAATAA-------
GCCTAAATAAAATGCAATTCATTCTTTCTGGGTCGATGCCCGAGGGGTTAATGGGGACGGAC
TGTAAATTCGTTGGCAATATGTCTACGC
</sequence>
<sequence>
<taxon idref="Armeriaspe"/>
T-------------------------------------
TCCTGAATGCTTCATTATGTGGATAAGATAT------ATA-T-----------------
GCATGTAGT-ACATAC-TA-
AGTTATTATAGACTATAACTATATAAGTATA----------------
TAAG---------------
AAAGTAGACTCATACTGGCTAGTGGCTTCTTCCGGAATGATAAAGTTGATTTCACTATCGAG
TCTAGGAT-AAAATC---ATTTATT--------
GATCTTTTTAAAATAAGACTTCAATACATCAAGCCGACCCTAA-TTTATTTG-----
ATTAAATTGATACCC------------ATCAGCAAAAAA-
TTAACTTGATACATGTACCTACCAATTTGACATAGATTCCAAGATATTTCA-----------
--------TTAAAAAATGGATGGAGCAGTTAGATTTGTCCACATAAATTCTTAG----
AAAAAAA---

------------GACACTCATTTTTTT---
CTATTCGAG---

TTGAGGAAGAATTCAAGGAATGCGGAACCAAAAAAATCTATGAAATCATCAAAGGCGGAAAG
ATCCTTTGGGTCGA------ATTCGATTCTTACAGAATATTGACAATTTTAAAAAA-
CTGATCATACTATGATCATAGTATGATGGCGGTTGGACACGTATGCCCCCATCGTCTAGTGG
TTCAGGACATCTCTCTTTCAAGGAGGCAGCGGGGATTCGACTTCCCCTGGGGG-

TAGGTTACTACA-AAAGAAAGTGAATCGTGCATTATCAATAA-------
GCCTAAATAAAATGGAATTCATTCTTTCTGGGTCGATGCCCGAGGGGTTAATGGGGACGGAC
TGTAAATTCGTTGGCAATATGTCTACGC
</sequence>
<sequence>
<taxon idref="Armeriasin"/>
T-------------------------------------
TCCTGAATGCTTCATTATGTGGATAAGATAT------ATA-T-----------------
GCATGTAGT-ACATAC-TA-
AGTTATTATAGACTATAACTATATAAGTATA----------------
TAAG---------------
AAAGTAGACTCATACTGGCTAGTGGCTTCTTCCGGAATGATAAAGTTGATTTCACTATCGAG
TCTAGGAT-AAAATC---ATTTATT--------
GATCTTTTTAAAATAAGACTTCAATACATCAAGCCGACCCTAA-TTTATTTG-----
ATTAAATTGATACCC------------ATCAGCAAAAAA-
TTAACTTGATACATGTACCTACCAATTTGACATAGATTCCAAGATATTTCA-----------
--------TTAAAAAATGGATGGAGCAGTTAGATTTGTCCACATAAATTCTTAG----
AAAAAAA---

------------GACACTCATTTTTTT---
CTATTCGAG---

TTGAGGAAGAATTCAAGGAATGCGGAACCAAAAAAATCTATGAAATCATCAAAGGCGGAAAG
ATCCTTTGGGTCGA------ATTCGATTCTTACAGAATATTGACAATTTTAAAAAA-
CTGATCATACTATGATCATAGTATGATGGCGGTTGGACACGTATGCCCCCATCGTCTAGTGG
TTCAGGACATCTCTCTTTCAAGGAGGCAGCGGGGATTCGACTTCCCCTGGGGG-
TAGGTTACTACA-AAAGAAAGTGAATCGTGCATTATCAATAA-------
GCCTAAATAAAATGGAATTCATTCTTTCTGGGTCGATGCCCGAGGGGTTAATGGGGACGGAC
TGTAAATTCGTTGGCAATATGTCTACGC
</sequence>
<sequence>
<taxon idref="Cephturcoc"/>
T-------------------------------------
TCCTGAATGCTTCATTATGTGAATAAGATAT------AGA-T-----------------
GCATGGAGT-ACATAC-TC-
AGTTATTCTAGACTATCAATATATAAA--------------------
TAAG---------------
AAAGTAGACTCATATTGGCTAGTGGCTTCTTCCGGAATGAGAAAGTGAATTTCACTATCGAT
TCTAGGAT-AAAATG---ATTTATG--------
GATCATTTTAAAATAGGATTTCAATACATCAATTCGACCCTAA-ATTCTTTT-----
ATGAAATTGATACTC------------ATCAGAACCAAA-
TTCACTTGATAGATGTACCTACCAATTCAAGAAAGATTCCAAGATATTTCA-----------
--------TTAAAAAATGGATGGAGAAGTTCGATTTGTCCACACAAATTCTTAGATC-
AAAAAAA---------TTTCCGAAACTCTCTTCATCCTTC----TTACT------
AGCTTTATCATCCCAGATTTCTTCTTTATTT-------ATTTT------
CTGGTATAGCTATTTCGTTTTT-----------------CTAGATTCTA-GAAA---T-
CTATTTTTT----GTT-----ATT-CAAAAGTGAGTTATATAACAAATC-
AAAAAAGAAAGAAA-------------------------------------
CTCATTTTT---

CGA------

TTTCGGTTCTTACACAATCTTGACAATTTCAAAAAAACGGATCATACTATGATCATAGTATG
ATGGCGGTTGGACACGTATGCCCCCATCGTCTAGTGGTTCAGGACATCTCTCTTTCAAGGAG
GCAGCGGGGATTCGACTTCCCCTGGGGG-TAGGTTACTACA-
AAAGAAAGTGAATCATGCATTATGAATAAGGAATAAGCCGAAA----
TTGGAATTTATTCTTCCTGGGTCGATGCCCGAGCGGTTAATGGGGACGGACTGTAAATTCGT
TGGCAATATGTCTACGC
</sequence>
<sequence>
<taxon idref="Cephcoe32"/>
T-------------------------------------
TCCTGAATGCTTCATTATGTGAATAAGATAT------AGA-T-----------------
GCATGGAGT-ACATAC-TC-
AGTTATTCTAGACTATCAATATATAAA--------------------
TAAGC--------------
AAAGTAGACTCATATTGGCTAGTGGCTTCTTCCGGAATGAGAAAGTGAATCTCACTAGCGAT
TCTAGGAT-AAAATA---ATTTATG--------
GATCATTTTAAAATAGGATTTCAATACATCAATTCGACCCCAA-TTTCTTTT-----
ATGAAATTGATACTC------------ATCAGAACCAAA-
TTCACTTGATAGATGTACCTACCAATTCAAGAAAGATTCCAAGATATTTCA-----------
--------TTAAAAAATGGGATGGAGAAGTTCGATTTGTCCACACAAATTCTTAGATC-
AAAAAAA---------TTTCCGAAACTCTCTTCATCCTTC----TTACT------
AGCTTTATCATCCCAGATTTCTTCTTTATTC-------ATTTT------
CTGGTATAGATATTTCGTTTTT-----------------CTAGATTCTA-GAAA---T-
CTATTTTTT----GTT-----ATT-CAAAAGTGAGTTATATAACAAATC-
AAAAAAGAAAGAAA-------------------------------------
CTCATTTTTT--

CGA------
TTTCGGTTCTTACACAATCTTGACAATTTCAAAAAAACGGATCATACTATGATCATAGTATG
ATGGCGGTTGGACACGTATGCCCCCATCGTCTAGTGGTTCAGGACATCTCTCTTTCAAGGAG
GCAGCGGGGATTCGACTTCCCCTGGGGG-TAGGTTACTACA-
AAAGAAAGTGAATCATGCATTATGAATAAGGAATAAGCCGAAA----
TTGGAATTTATTCTTCCTGGGTCGATGCCCGAGCGGTTAATGGGGACGGACTGTAAATTCGT
TGGCAATATGTCTACGC
</sequence>
<sequence>
<taxon idref="Bamianiaaa"/>
T-------------------------------------
TCCTGAATGCTTCATTATGTGAATAAGATAT------AGA-T-----------------
GCATGGAGT-ACATAC-TC-
AGTTATTCTAGACTATCAATATATAAA--------------------
TAAG---------------
AAAGTAGACTCATATTGGCTAGTGGCTTCTTCCGGAATGAGAAAGTGAATTTCACTATCGAT
TCTAGGAT-AAAATG---ATTTGTT--------
GATCATTTTAAAATAGGATTTCAATACATCAATTCGACCCCAA-TTTCTTTT-----
ATGAAATTGATACTC------------ATCAAAACCAAA-
TTCACTTGATAGATGTACCTACCAATTCAAGAAAGATTCCAAGATATTTCA-----------
--------TTAAAAAATGGGATGGAGAAGTTCGATTTGTCCACACAAATTCTTAGATC-
AAAAAAA---------TTTCCGAAACTCTCTTCATCCTTC----TTACT------
AGCTTTATCATCCCAGATTTCTTCTTTATTC-------ATTTT------
CTGGTATAGATATTTCGTTTTT-----------------CTAGATTCTA-GAAA---T-
CTATTTTTG----GTT-----ATT-CAAAAGTGAGTTATATAACAAATC-
AAAAAAGAAAGAAA-------------------------------------
CTCATTTTTT--

CGA------
TTTCGGTTCTTACACAATCTTGACAATTTCAAAAAAACGGATCATACTATGATCATAGTATG
ATGGCGGTTGGACACGTATGCCCCCATCGTCTAGTGGTTCAGGACATCTCTCTTTCAAGGAG
GCAGCGGGGATTCGACTTCCCCTGGGGG-TAGGTTACTACA-
AAAGAAAGTGAATCATGCATTATGAATAAGGAATAAGCCGAAA----
TTGGAATTTATTCTTCCTGGGTCGATGCCCGAGCGGTTAATGGGGACGGACTGTAAATTCGT
TGGCAATATGTCTACGC
</sequence>
<sequence>
<taxon idref="Dicgrif30"/>

T--

</sequence>
<sequence>
<taxon idref="Dicmacr34"/>
T-------------------------------------
TCCTGAATGCTTCATTATGTGAATAAGATAT------AGA-T-----------------
GCATGGAGT-ACATAC-TC-
AGTTATTCTAGACTATCAATATATAAA--------------------
TAAG---------------
AAAGTAGACTCATATTGGCTAGTGGCTTCTTCCGGAATGAGAAAGTGAATTTCACTATCGAT
TCTAGGAT-CAAATG---ATTTATT--------
GATCGTTTTAAAATAGGATTTCAATACATCAATTCGACCCCAA-TTTCTTTT-----
ATGAAATTGATACTC------------ATCAGAACCAAA-
TTCACTTGATAGATGTACCTACCAATTCAAGAAAGATTCCAAGATATTTCA-----------
--------TTAAAAAAATGGGATGGAGAAGTTCGATTTGTCCACACAAATTCTTAGATC-
AAAAAAA---------TTTCCGAAACTCTCTTCATCCTTC----TTACT------
AGCTTTATCATCCCAGATTTCTTCTTTATTTATTATTTATTTT------
CTGGTATAGATATTTCGTTTTT-----------------CTAGATTCTA-GAAA---T-
CTATTTTTT----GTT-----ATT-CAAAAGTGAGTTATATAACAAATC-
AAAAAAGAAAGAAA-------------------------------------
CTCATTTTT---

AGA------
TTTCGGTTCTTACACAATCTTGACAATTTCAAAAAAACGGATCATACTATGATCATAGTATG
ATGGCGGTTGGACACGTATGCCCCCATCGTCTAGTGGTTCAGGACATCTCTCTTTTAAGGAG
GCAGCGGGGATTCGACTTCCCTGGGGG-TAGGTTACTACA-
AAAGAAAGTGAATCATGCATTATGAATAA-------GCCGAAA----
TTGGAATTTATTCTTCCTGGGTCGATGCCCGAGCGGTTAATGGGGACGGACTGTAAATTCGT
TGGCAATATGTCTACGC
</sequence>
<sequence>
<taxon idref="Bukcabu39"/>
T-------------------------------------
TCCTGAATGCTTCATTATGTGAATAAGATAT------AGA-T-----------------
GCATGGAGT-ACATAC-TC-
AGTTATTCTAGACTATCAATATATAAA--------------------
TAAG---------------
AAAGTAGACTCATATTGGCTAGTGGCTTCTTCCGGAATGAGAAAGTGAATTTCACTATCGAT
TCTAGGAT-AAAATG---ATTTATT--------
GATCGTTTTAAAATAGGATTTCAATACATCAATTCGACCCCAA-TTTCTTTT-----
ATGAAATGGATACTC------------ATCAGAACCAAA-
TTCACTTGATAGATGTACCTACCAATTCAAGAAAGATTCCAAGATATTTCA-----------
--------TTAAAAAATGGGATAGAGAAGTTCGATTTGTCCACACAAATTCTTAGATC-
AAAAAAA---------TGTCCGAAACTCTCTTCATCCTTC----TTACT------
AGCTTTATCATCCCAGATTTCTTCTTTATTC-------ATTTT------
CTGGTATAGATATTTCGTTTTT-----------------CTAGATTCTA-GAAA---T-
CTATTTTTT----GTT-----ATT-CAAAAGTGAGTTATATAACAAATC-
AAAAAAGAAAGAAA-------------------------------------
CTCATTTTTT--

CGATTTCGATTTCGGTTCTTACACAATCTTGACAATTTCAAAAAAACGGATCATACTATGAT
CATAGTATGATGGCGGTTGGACACGTATGCCCCCATCGTCTAGTGGTTCAGGACATCTCTCT
TTTAAGGAGGCAGCGGGGATTCGACTTCCCTGGGGG-TAGGTTACTACA-
AAAGAAAGTGAATCATGCATTATGAATAA-------GCCGAAA----
TTGGAATTTATTCTTCCTGGGTCGATGCCCGAGCGGTTAATGGGGACGGACTGTAAATTCGT
TGGCAATATGTCTACGC
</sequence>
<sequence>
<taxon idref="Vassizso44"/>

T--

</sequence>
<sequence>
<taxon idref="ChaetolSet"/>

</sequence>
<sequence>
<taxon idref="Glaspeci25"/>
T-------------------------------------
TCCTGAATGCTTCATTATGTGAATAAGATAT------AGA-T-----------------
GCATGGAGT-ACATAC-TC-
AGTTATTCTAGACTATCAATATATAAA--------------------
TAAGTATATAAATAAG---
AAAGTAGACTCATATTGGCTAGTGGCTTCTTCCGGAATGAGAAAGTGAATTTCACTATCGAT
TCTAGGAT-AAAATG---ATTTATT--------
GATCGTTTTAAAATAGGATTTCAATACATCAATTCGACCCCAA-TTTCTTTT-----
ATGAAATTGATACTC------------ATCAGAACCAAA-
TTCACTTGATAGATGTACCTACCAATTCAAGAAAGATTCCAAGATATTTCA-----------
--------TTAAAAAATGGGATGGAGAAGTTCGATTTGTCCACACAAATTCTTAGATC-
AAAAAA----------TTTCCGAAACTCTCTTCATCCTTC----TTACT------
AGCTTTATCATCCCAGATTTCTTCTTTATTC-------ATTTT------
CTGGTATAGATATTTCGTTTTT-----------------CTAGATTCTA-GAAA---T-
CTATTTTTT----GTT-----ATT-CAAAAGTGAGTTATATAACAAATC-
AAAAAAGAAA---
CTCATTTTTT--

CGA------
TTTCGGTTCTTACACAATCTTGACAATTTCAAAAAAACGGGTCATACTATGATCATAGTATG
ATGGCGGTTGGACACGTATGCCCCCATCGTCTAGTGGTTCAGGACATCTCTCTTTCAAGGAG
ACAGCGGGGATTCGACTTCCCCTGGGGG-TAGGTTACTACA-
AAAGAAAGTGAATCATGCATTATGAATAA-------GCCGAAA----
TTGGAATTTATTCTTCCTGGGTCGATGCCCGAGCGGTTAATGGGGACGGACTGTAAATTCGT
TGGCAATATGTCTACGC
          </sequence>
          <sequence>
               <taxon idref="Alavaeaaaa"/>
               T-------------------------------------
TCCTGAATGCTTCATTATGTGAATAAGATAT------AGA-T-----------------
GCATGGAGT-ACATAC-TC-
AGTTATTCTAGACTATCAATATATAAA--------------------
TAAG---------------
AAAGTAGACTCATATTGGCTAGTGGCTTCTTCCGGAATGAGAAAGTGAATTTCACTATCGAT
TCTAGGAT-AAAATG---ATTTATT--------
GATCGTTTTAAAATAGGATTTCAATACATCAATTCGACCCCAA-TTTCTTTT-----
ATGAAATTGATACTC------------ATCAGAACCAAA-
TTCACTTGATAGATGTACCTACCAATTCAAGAAAGATTCCAAGATATTTCA-----------
--------TTAAAAAATGGGATGGAGAAGTTCGATTTGTCCACACAAATTCTTAGATC-
AAAAAA----------TTTCCGAAACTCTCTTCATCCTTC----TTACT------
AGCTTTATCATCCCAGATTTCTTCTTTATTC-------ATTTT------
CTGGTATAGATATTTCGTTTTT-----------------CTAGATTCTA-GAAA---T-
CTATTTTTT----GTT-----ATT-CAAAAGTGAGTTATATAACAAATC-
AAAAAAGAAA---
CTCATTTTTT--

CGA------
TTTCGGTTCTTACACAATCTTGACAATTTCAAAAAAACGGGTCATACTATGATCATAGTATG
ATGGCGGTTGGACACGTATGCCCCCATCGTCTAGTGGTTCAGGACATCTCTCTTTCAAGGAG
GCAGCGGGGATTCGACTTCCCCTGGGGG-TAGGTTACTACA-
AAAGAAAGTGAATCATGCATTATGAATAA-------GCCGAAA----
TTGGAATTTATTCTTCCTGGGTCGATGCCCGAGCGGTTAATGGGGACGGACTGTAAATTCGT
TGGCAATATGTCTACGC
          </sequence>
          <sequence>
               <taxon idref="Bodeanum1m"/>
               T-------------------------------------
TCCTGAATGCTTCATTATGTGAATAAGATAT------AGA-T-----------------
GCATGGAGT-ACATAC-TC-
AGTTATTCTAGACTATCAATATATAAA--------------------
TAAG---------------
AAAGTAGACTCATATTGGCTAGTGGCTTCTTCCGGAATGAGAAAGTGAGTTTCACTATCGAT
TCTAGGAT-AAAATG---ATTTATT--------
GATCGTTTTAAAATAGGATTTCAATACATCAATTCGACCCCAA-TTTCTTTT-----
ATGAAATTGATACTC------------ATCAGAACCAAA-
TTCACTTGATAGATGTACCTACCAATTCAAGAAAGATTCCAAGATATTTCA-----------
--------TTAAAAAATGGGATGGAGAAGTTCGATTTGTCCACACAAATTCTTAGATC-
AAAAAA----------TTTCCGAAACTCTCTTCATCCTTC----TTACT------
AGCTTTATCATCCCAGATTTCTTCTTTATTC-------ATTTT------
CTGGTATAGATATTTCGTTTTT-----------------CTAGATTCTA-GAAA---T-

CTATTTTTT----GTT-----ATT-CAAAAGTGAGTTATATAACAAATC-
AAAAAAGAAA---
CTCATTTTTT--

CGA------
TTTCGGTTCTTACACAATCTTGACAATTTCAAAAAAACGGGTCATACTATGATCATAGTATG
ATGGCGGTTGGACACGTATGCCCCCATCGTCTAGTGGTTCAGGACATCTCTCTTTCAAGGAG
GCAGCGGGGATTCGACTTCCCCTGGGGG-TAGGTTACTACA-
AAAGAAAGTGAATCATGCATTATGAATAA-------GCCGAAA----
TTGGAATTTATTCTTCCTGGGTCGATGCCCGAGCGGTTAATGGGGACGGACTGTAAATTCGT
TGGCAATATGTCTACGC
</sequence>
<sequence>
<taxon idref="scorpiu927"/>
T-------------------------------------
TCCTGAATGCTTCATTATGTGAATAAGATAT------AGA-T-----------------
GCATGGAGT-ACATAC-TC-
AGTTATTCTAGACTATCAATATATAAA--------------------
TAAG---------------
AAAGTAGACTCATATTGGCTAGTGGCTTCTTCCGGAATGAGAAAGTGAATTTCACTATCGAT
TCTAGGAT-AAAATG---ATTTATT--------
GATCGTTTTAAAATAGGATTTCAATACATCAATTCGACCCCAA-TTTCTTTT-----
ATGAAATTGATACTC------------ATCAGAACCAAA-
TTCACTTGATAGATGTACCTACCAATTCAAGAAAGATTCCAAGATATTTCA-----------
--------TTAAAAAATGGGATGGAGAAGTTCGATTTGTCCACACAAATTCTTAGATC-
AAAAAA----------TTTCCGAAACTCTCTTCATCCTTC----TTACT------
AGCTTTATCATCCCAGATTTCTTCTTTATTC-------ATTTT------
CTGGTATAGATATTTCGTTTTT-----------------CTAGATTCTA-GAAA---T-
CTATTTTTT----GTT-----ATT-CAAAAGTGAGTTATATAACAAATC-
AAAAAAGAAA---
CTCATTTTTT--

CGA------
TTTCGGTTCTTACACAATCTTGACAATTTCAAAAAAACGGGTCATACTATGATCATAGTATG
ATGGCGGTTGGACACGTATGCCCCCATCGTCTAGTGGTTCAGGACATCTCTTTTTCAAGGAG
GCAGCGGGGATTCGACTTCCCCTGGGGG-TAGGTTACTACA-
AAAGAAAGTGAATCATGCATTATGAATAA-------GCCGAAA----
TTGGAATTTATTCTTCCTGGGTCGATGCCCGAGCGGTTAATGGGGACGGACTGTAAATTCGT
TGGCAATATGTCTACGC
</sequence>
<sequence>
<taxon idref="Aspadanumm"/>
T------------------
AAGGGCCTTTTTCGTAAAGTCCTGAATGCTTCATTATGTGAATAAGATAT------AGA-
T------------TAGATGCATGGAGT-----AC-TC-
AGTTATTCTAGACTATCAATATATAAA--------------------
TAAG---------------AAA----
ACTCATATTGGCTAGTGGCTTCTTCCGGAATGAGAAAGTGAATTTCACTATCGATTCTAGGA
T-AAAATG---ATTTATT--------
GATCGTTTTAAAATAGGATTTCAATACATCAATTCGACCCCAA-TTTCTTGG-----
ATGAAATTGATACTC------------ATCAGAACCAAA-
TTCACTTGATAGATGTACCTACCAATTCAAGAAAGATTCCAAGATATTTCA-----------

--------TTAAAAAAATGGGATGGAGAAGTTCGATTTGTCCACACAAATTCTTAGATCC-
AAAAA----------TTTCCGAAACTCTCTTCATCCTTC----TTACT------
AGCTTTATCATCCCAGATTTCTTCTTTATTC-------ATTTT------
CTGGTATAGATATTTCGTTTTT-----------------CTAGATTCTA-GAAA---T-
CGATTTTTT----GTT-----ATT-CAAAAGTGAGTTATATAACAAATC-
AAAAAAGAAA---
CTCATTTTT---

CGA------
TTTCGGTTCTTACACAATCTTGACAATTTCAAAAAAACGGATCATACTATGATCATAGTATG
ATGGCGGTTGGACACGTATGCCCCCATCGTCTAGTGGTTCAGGACATCTCTCTTTCAAGGAG
GCAGCGGGGATTCGACTTCCCCTGGGGG-TAGGTTACTACA-
AAAGAAAGTGAATCATGCATTATGAATAA-------GCCGAAA----
TTGGAATTTATTCTTGCTGGGTCGATGCCCGAGCGGTTAATGGGGACGGACTGTAAATTCGT
TGGCAATATGTCTACGC
</sequence>
<sequence>
<taxon idref="Bromifoliu"/>
T-------------------------------------
TCCTGAATGCTTCATTATGTGAATAAGATAT------AGA-T------------
TAGATGCATGGAGT-----AC-TC-
AGTTATTCTAGACTATCAATATATAAA--------------------
TAAG---------------AAA----
ACTCATATTGGCTAGTGGCTTCTTCCGGAATGAGAAAGTGAATTTCACTATCGATTCTAGGA
T-AAAATG---ATTTATT--------
GATCGTTTTAAAATAGGATTTCAATACATCAATTCGACCCCAA-TTTCTTTG-----
ATGAAATTGATACTC------------ATCAGAACCAAA-
TTCACTTGATAGATGTACCTACCAATTCAAGAAAGATTCCAAGATATTTCA-----------
--------TTAAAAAAATGGGATGGAGAAGTTCGATTTGTCCACACAAATTCTTAGATCC-
AAAAA----------TTTCCGAAACTCTCTTCATCCTTC----TTACT------
AGCTTTATCATCCCAGATTTCTTCTTTATTC-------ATTTT------
CTGGTATAGATATTTCGTTTTT-----------------CTAGATTCTA-GAAA---T-
CGATTTTTT----GTT-----ATT-CAAAAGTGAGTTATATAACAAATC-
AAAAAAGAAA---
CTCATTTTT---

CGA------
TTTCGGTTCTTACACAATCTTGACAATTTCAAAAAAACGGATCATACTATGATCATAGTATG
ATGGCGGTTGGACACGTATGCCCCCATCGTCTAGTGGTTCAGGACATCTCTCTTTCAAGGAG
GCAGCGGGGATTCGACTTCCCCTGGGGG-TAGGTTACTACA-
AAAGAAAGTGAATCATGCATTATGAAAAA-------GCCGAAA----
TTGGAATTTATTCTTGCTGGGTCGATGCCCGAGCGGTTAATGGGGACGGACTGTAAATTCGT
TGGCAATATGTCTACGC
</sequence>
<sequence>
<taxon idref="Bracteatum"/>
T-------------------------------------
TCCTGAATGCTTCATTATGTGAATAAGATAT------AGA-T------------
TAGATGCATGGAGT-----AC-TC-
AGTTATTCTAGACTATCAATATATAAA--------------------
TAAG---------------AAA----
ACTCATATTGGCTAGTGGCTTCTTCCGGAATGAGAAAGTGAATTTCACTATCGATTCTAGGA

T-AAAATG---ATTTATT--------
GATCGTTTTAAAATAGGATTTCAATACATCAATTCGACCCCAA-TTTCTTTG-----
ATGAAATTGATACTC------------ATCAGAACCAAA-
TTCACTTGATAGATGTACCTACCAATTCAAGAAAGATTCCAAGATATTTCA-----------
--------TTCAAAAATGGGATGGAGAAGTTCGATTTGTCCACACAAATTCTTAGATC-
AAAAAA----------TTTCCGAAACTCTCTTCATCCTTC----TTACT------
AGCTTTATCATCCCAGATTTCTTCTTTATTC-------ATTTT------
CTGGTATAGATATTTTGTTTTT-----------------CTAGATTCTA-GAAA---T-
CTATTTTTT----GTT-----ATT-CAAAAGTGAGTTATATAACAAATC-
AAAAAAGAAA---
CTCATTTTT---

CGA------
TTTCGGTTCTTACACAATCTTGACAATTTCAAAAAAACGGATCATACTATGATCATAGTATG
ATGGCGGTTGGACACGTATGCCCCCATCGTCTAGTGGTTCAGGACATCTCTCTTTCAAGGAG
GCAGCGGGGATTCGACTTCCCCTGGGGG-TAGGTTACTACA-
AAAGAAAGTGAATCATGCATTATGAATAA-------GCCGAAA----
TTGGAATTTATTCTTGCTGGGTCGATGCCCGAGCGGTTAATGGGGACGGACTGTAAATTCGT
TGGCAATATGTCTACGC
</sequence>
<sequence>
<taxon idref="Flexuosumm"/>
T-------------------------------------
TCCTGAATGCTTCATTATGTGAATAAGATAT------AGA-T------------
TAGATGCATGGAGT-----AC-TC-
AGTTATTCTAGACTATCAATATATAAA--------------------
TAAG---------------AAA----
ACTCATATTGGCTAGTGGCTTCTTCCGGAATGAGAAAGTGAATTTCACTATCGATTCTAGGA
T-AAAATG---ATTTATT--------
GATCGTTTTAAAATAGGATTTCAATACATCAATTCGACCCCAA-TTTCTTGG-----
ATGAAATTGATACTC------------ATCAGAACCAAA-
TTCACTTGATAGATGTACCTACCAATTCAAGAAAGATTCCAAGATATTTCA-----------
--------TTAAAAAATGGGATGGAGAAGTTCGATTTGTCCACACAAATTCTTAGATCC-
AAAAA----------TTTCCGAAACTCTCTTCATCCTTC----TTACT------
AGCTTTATCATCCCAGATTTCTTCTTTATTC-------ATTTT------
CTGGTATAGATATTTCGTTTTT-----------------CTAGATTCTA-GAAA---T-
CGATTTTTT----GTT-----ATT-CAAAAGTGAGTTATATAACAAATC-
AAAAAAGAAA---
CTCATTTTT---

CGA------
TTTCGGTTCTTACACAATCTTGACAATTTCAAAAAAACGGATCATACTATGATCATAGTATG
ATGGCGGTTGGACACGTATGCCCCCATCGTCTAGTGGTTCAGGACATCTCTCTTTCAAGGAG
GCAGCGGGGATTCGACTTCCCCTGGGGG-TAGGTTACTACA-
AAAGAAAGTGAATCATGCATTATGAATAA-------GCCGAAA----
TTGGAATTTATTCTTGCTGGGTCGATGCCCGAGCGGTTAATGGGGACGGACTGTAAATTCGT
TGGCAATATGTCTACGC
</sequence>
<sequence>
<taxon idref="Pterostegi"/>
T-------------------------------------
TCCTGAATGCTTCATTATGTGAATAAGATAT------

AGAATGTGAATAAGATATAGATGCATGGAGT-ACATAC-TC-
AGTTATTCTAGACTATCAATATATAAA--------------------
TAAG---------------
AAAGTAGACTCATATTGGCTAGTGGCTTCTTCCGGAATGAGAAAGTGAATTTCACTATCGAT
TCTAGGAT-AAAATG---ATTTATT--------
GATCGTTTTAAAATAGGATTTCAATACATCAATTCGACCCCAA-TTTCTTTT-----
ATGAAATTGATACTC------------ATCAGAACCAAA-
TTCACTTGATAGATGTACCTACCAATTCAAGAAGGATTCCAAGATATTTCA-----------
--------TTAAAAAATGGGATGGAGAAGTTCGATTTGTCCACACAAATTCGTAGATC-
AAAAAA----------TTTCCGAAACTCTCTTCATCCTTC----TTACT------
AGCTTTATCATCCCAGATTTCTTCTTTATTC-------ATTTT----------
CATAGATATTTCGTTTTT-----------------CTAGATTCTA-GAAA---T-
CTATTTTTT----GTT-----ATT-CAAAAGTGAGTTATATAACAAATC-
AAAAAAGAAA---
CTCATTTTT---

CGA------
TTTCGGTTCTTACACAATCTTGACAATTTCAAAAAAACGGGTCATACTATGATCATAGTATG
ATGGCGGTTGGACACGTATGCCCCCATCGTCTAGTGGTTCAGGACATCTCTCTTTCAAGGAG
GCAGCGGGGATTCGACTTCCCCTGGGGG-TAGGTTACTACA-
AAAGAAAGTGAATCATGCATTATGAATAA-------GCCGAAA----
TTGGAATTTATTCTTCCTGGGTCGATGCCCGAGCGGTTAATGGGGACGGACTGTAAATTCGT
TGGCAATATGTCTACGC
</sequence>
<sequence>
<taxon idref="Cymosummmm"/>
T-------------------------------------
TCCTGAATGCTTCATTATGTGAATAAGATAT------
AGAATGTGAATAAGATATAGATGCATGGAGT-ACATAC-TC-
AGTTATTCTAGACTATCAATATATAAA--------------------
TAAG---------------
AAAGTAGACTCATATTGGCTAGTGGCTTCTTCCGGAATGAGAAAGTGAATTTCACTATCGAT
TCTAGGAT-AAAATG---ATTTATT--------
GATCGTTTTAAAATAGGATTTCAATACATCAATTCGACCCCAA-TTTCTTTT-----
ATGAAATTGATACTC------------ATCAGAACCAAA-
TTCACTTGATAGATGTACCTACCAATTCAAGAAGGATTCCAAGATATTTCA-----------
--------TTAAAAAATGGGATGGAGAAGTTCGATTTGTCCACACAAATTCGTAGATC-
AAAAAA----------TTTCCGAAACTCTCTTCATCCTTC----TTACT------
AGCTTTATCATCCCAGATTTCTTCTTTATTC-------ATTTT----------
CATAGATATTTCGTTTTT-----------------CTAGATTCTA-GAAA---T-
CTATTTTTT----GTT-----ATT-CAAAAGTGAGTTATATAACAAATC-
AAAAAAGAAA---
CTCATTTTT---

CGA------
TTTCGGTTCTTACACAATCTTGACAATTTCAAAAAAACGGGTCATACTATGATCATAGTATG
ATGGCGGTTGGACACGTATGCCCCCATCGTCTAGTGGTTCAGGACATCTCTCTTTCAAGGAG
GCAGCGGGGATTCGACTTCCCCTGGGGG-TAGGTTACTACA-
AAAGAAAGTGAATCATGCATTATGAATAA-------GCCGAAA----
TTGGAATTTATTCTTCCTGGGTCGATGCCCGAGCGGTTAATGGGGACGGACTGTAAATTCGT
TGGCAATATGTCTACGC
</sequence>

<sequence>
<taxon idref="Gilliatiii"/>
T--------------------------------
AAAGTCCTGAATGCTTCATTATGTGAATAAGATAT------AGA-T-----------
TAGATGCATGGAGT-----AC-TC-
AGTTATTCTAGACTATCAATATATAAA-------------------
TAAG---------------AAA----
ACTCATATTGGCTAGTGGCTTCTTCCGGAATGAGAAAGTGAATTTCACTATCGATTCTAGGA
T-AAAATG---ATTTATT--------
GATCGTTTTAAAATAGGATTTCAATACATCAATTCGACCCCAA-TTTCTTTG-----
ATGAAATTGATACTC-----------ATCAGAACCAAA-
TTCACTTGATAGATGTACCTACCAATTCAAGAAAGATTCCAAGATATTTCA----------
--------TTAAAAAATGGGATGGAGAAGTTCGATTTGTCCACACAAATTCTTAGATCC-
AAAAA----------TTTCCGAAACTCTCTTCATCCTTC----TTACT------
AGCTTTATCATCCCAGATTTATTCTTTATTC-------ATTTT------
CTGGTATAGATATTTCGTTTTT----------------CTAGATTCTA-GAAA---T-
CGATTTTTT----GTT-----ATT-CAAAAGTGAGTTATATAACAAATC-
AAAAAAGAAA--
CTCATTTTT--

CGA------
TTTCGGTTCTTACACAATCTTGACAATTTCAAAAAAACGGATCATACTATGATCATAGTATG
ATGGCGGTTGGACACGTATGCCCCCATCGTCTAGTGGTTCAGGACATCTCTCTTTCAAGGAG
GCAGCGGGGATTCGACTTCCCCTGGGGG-TAGGTTACTACA-
AAAGAAAGTGAATCATGCATTATGAATAA-------GCCGAAA----
TTGGAATTTATTCTTGCTGGGTCGATGCCCGAGCGGTTAATGGGGACGGACTGTAAATTCGT
TGGCAATATGTCTACGC
</sequence>
<sequence>
<taxon idref="Qunique lob"/>
T------------------------------------
TCCTGAATGCTTCATTATGTGAATAAGATAT------AGA-T----------------
GCATGGAGT-ACATAC-TC-
AGTTATTCTAGACTATCAATATATAAA-------------------
TAAG--------------
AAAGTAGACTCATATTGGCTAGTGGCTTCTTCCGGAATGAGAAAGTGAATTTCACTATCGAT
TCTAGGAT-AAAATG---ATTTATT--------
GATCGTTTTAAAATAGGATTTCAATACATCAATTCGACCCCAA-TTTCTTTT-----
ATGAAATTGATACTC-----------ATCAGAACCAAA-
TTCACTTGATAGATGTACCTACCAATTCAAGAAAGATTCCAAGATATTTCA----------
--------TTAAAAAATGGGATGGAGAAGTTCGATTTGTCCACACAAATTCTTAGATCC-
AAAAA----------TTTCCGAAACTCTCTTCATCCTTC----TTACT------
AACTTTATCATCCCAGATTTCTTCTTTATTC-------ATTTT------
CTGGTATAGATATTTCGTTTTT----------------CTAGATTCTA-GAAA---T-
CTATTTTTT----GTT-----ATT-CAAAAGTGAGTTATATAACAAATC-
AAAAAAGAAA--
CTCATTTTT--

CGA------
TTTCGGTTCTTACACAATCTTGACAATTTCAAAAAAACGGGTCATACTATGATCATAGTATG
ATGGCGGTTGGACACGTATGCCCCCATCGTCTAGTGGTTCAGGACATCTCTCTTTCAAGGAG
GCAGCGGGGATTCGACTTCCCCTGGGGG-TAGGTTACTACA-

AAAGAAAGTGAATCATGCATTATGAATAA-------GCCGAAA----
TTGGAATTTATTCTTCCTGGGTCGATGCCCGAGCGGTTAATGGGGACGGACTGTAAATTCGT
TGGCAATATGTCTACGC
</sequence>
<sequence>
<taxon idref="Scirpinumm"/>
T-------------------------------------
TCCTGAATGCTTCATTATGTGAATAAGATAT------AGA-T-----------------
GCATGGAGT-ACATAC-TC-
AGTTATTCTAGACTATCAATATATAAA--------------------
TAAG---------------
AAAGTAGACTCATATTGGCTAGTGGCTTCTTCCGGAATGAGAAAGTGAGTTTCACTATCGAT
TCTAGGAT-AAAATG---ATTTATT--------
GATCGTTTTAAAATAGGATTTCAATACATCAATTCGACCCCAA-TTTCTTTT-----
ATGAAATTGATACTC------------ATCAGAACCAAA-
TTCACTTGATAGATGTACCTACCAATTCAAGAAAGATTCCAAGATATTTCA-----------
--------TTAAAAAATGGGATGGAGAAGTTCGATTTGTCCACACAAATTCTTAGATC-
AAAAAA----------TTTCCGAAACTCTCTTCATCCTTC----TTACT------
AGCTTTATCATCCCAGATTTCTTCTTTATTC-------ATTTT------
CTGGTATAGATATTTCGTTTTT-----------------CTAGATTCTA-GAAA---T-
CTATTTTTT----GTT-----ATT-CAAAAGTGAGTTATATAACAAATC-
AAAAAAGAAA---
CTCATTTTTT--

CGA------
TTTCGGTTCTTACACAATCTTGACAATTTCAAAAAAACGGGTCATACTATGATCATAGTATG
ATGGCGGTTGGACACGTATGCCCCCATCGTCTAGTGGTTCAGGACATCTCTCTTTCAAGGAG
GCAGCGGGGATTCGACTTCCCCTGGGGG-TAGGTTACTACA-
AAAGAAAGTGAATCATGCATTATGAATAA-------GCCGAAA----
TTGGAATTTATTCTTCCTGGGTCGATGCCCGAGCGGTTAATGGGGACGGACTGTAAATTCGT
TGGCAATATGTCTACGC
</sequence>
<sequence>
<taxon idref="Zaefii1iii"/>
T---------------------------------
AAAGTCCTGAATGCTTCATTATGTGAATAAGATAT------AGA-T------------
TAGATGCATGGAGT-----AC-TC-
AGTTATTCTAGACTATCAATATATAAA--------------------
TAAG---------------AAA----
ACTCATATTGGCTAGTGGCTTCTTCCGGAATGAGAAAGTGAATTTCACTATCGATTCTAGGA
T-AAAATG---ATTTATT--------
GATCGTTTTAAAATAGGATTTCAATACATCAATTCGACCCCAA-TTTCTTTG-----
ATGAAATTGATACTC------------ATCAGAACCAAA-
TTCACTTGATAGATGTACCTACCAATTCAAGAAAGATTCCAAGATATTTCA-----------
--------TTAAAAAATGGGATGGAGAAGTTCGATTTGTCCACACAAATTCTTAGATC-
AAAAAA----------TTTCCGAAACTCTCTTCATCCTTC----TTACT------
AGCTTTATCATCCCAGATTTCTTCTTTATTC-------ATTTT------
CTGGTATAGATATTTCGTTTTT-----------------CTAGATTCTA-GAAA---T-
CTATTTTTTT---TTT-----ATT-CAAAAGTGAGTTATATAACAAATC-
AAAAAAGAAA---
CTCATTTTTT--

CGA------
TTTCGGTTCTTACACAATCTTGACAATTTCAAAAAAACGGATCATACTATGATCATAGTATG
ATGGCGGTTGGACACGTATGCCCCCATCGTCTAGTGGTTCAGGACATCTCTCTTTCAAGGAG
GCAGCGGGGATTCGACTTCCCCTGGGGG-TAGGTTACTACA-
AAAGAAAGTGAATCATGCATTATGAATAA-------GCCGAAA----
TTGGAATTTATTCTTGCTGGGTCGATGCCCGAGCGGTTAATGGGGACGGACTGTAAATTCGT
TGGCAATATGTCTACGC
</sequence>
<sequence>
<taxon idref="Kermanense"/>
T-------------------------------------
TCCTGAATGCTTCATTATGTGAATAAGATAT------AGA-T-----------------
GCATGGAGT-ACATAC-TC-
AGTTATTCTAGACTATCAATATATAAA--------------------
TAAG---------------
AAAGTAGACTCATATTGGCTAGTGGCTTCTTCCGGAATGAGAAAGTGAGTTTCACTATCGAT
TCTAGGAT-AAAATG---ATTTATT--------
GATCGTTTTAAAATAGGATTTCAATACATCAATTCGACCCCAA-TTTCTTTT-----
ATGAAATTGATACTC------------ATCAGAACCAAA-
TTCACTTGATAGATGTACCTACCAATTCAAGAAAGATTCCAAGATATTTCA-----------
--------TTAAAAAATGGGATGGAGAAGTTCGATTTGTCCACACAAATTCTTAGATC-
AAAAAA----------TTTCCGAAACTCTCTTCATCCTTC----TTACT------
AGCTTTATCATCCCAGATTTCTTCTTTATTC-------ATTTT------
CTGGTATAGATATTTCGTTTTT-----------------CTAGATTCTA-GAAA---T-
CTATTTTTT----GTT-----ATT-CAAAAGTGAGTTATATAACAAATC-
AAAAAAGAAA---
CTCATTTTTT--

CGA------
TTTCGGTTCTTACACAATCTTGACAATTTCAAAAAAACGGGTCATACTATGATCATAGTATG
ATGGCGGTTGGACACGTATGCCCCCATCGTCTAGTGGTTCAGGACATCTCTCTTTCAAGGAG
GCAGCGGGGATTCGACTTCCCCTGGGGG-TAGGTTACTACA-
AAAGAAAGTGAATCATGCATTATGAATAA-------GCCGAAA----
TTGGAATTTATTCTTCCTGGGTCGATGCCCGAGCGGTTAATGGGGACGGACTGTAAATTCGT
TGGCAATATGTCTACGC
</sequence>
<sequence>
<taxon idref="Chlorosteg"/>
T-------------------------------------
TCCTGAATGCTTCATTATGTGAATAAGATAT------AGA-T-----------------
GCATGGAGT-ACATAC-TC-
AGTTATTCTAGACTATCAATATATAAA--------------------
TAAG---------------
AAAGTAGACTCATATTGGCTAGTGGCTTCTTCCGGAATGAGAAAGTGAGTTTCACTATCGAT
TCTAGGAT-AAAATG---ATTTATT--------
GATCGTTTTAAAATAGGATTTCAATACATCAATTCGACCCCAA-TTTCTTTT-----
ATGAAATTGATACTC------------ATCAGAACCAAA-
TTCACTTGATAGATGTACCTACCAATTCAAGAAAGATTCCAAGATATTTCA-----------
--------TTAAAAAATGGGATGGAGAAGTTCGATTTGTCCACACAAATTCTTAGATC-
AAAAAA----------TTTCCGAAACTCTCTTCATCCTTC----TTACT------
AGCTTTATCATCCCAGATTTCTTCTTTATTC-------ATTTT------
CTGGTATAGATATTTCGTTTTT-----------------CTAGATTCTA-GAAA---T-
CTATTTTTT----GTT-----ATT-CAAAAGTGAGTTATATAACAAATC-
AAAAAAGAAA---

CTCATTTTTT--

CGA------
TTTCGGTTCTTACACAATCTTGACAATTTCAAAAAAACGGGTCATACTATGATCATAGTATG
ATGGCGGTTGGACACGTATGCCCCCATCGTCTAGTGGTTCAGGACATCTCTCTTTCAAGGAG
GCAGCGGGGATTCGACTTCCCCTGGGGG-TAGGTTACTACA-
AAAGAAAGTGAATCATGCATTATGAATAA-------GCCGAAA----
TTGGAATTTATTCTTCCTGGGTCGATGCCCGAGCGGTTAATGGGGACGGACTGTAAATTCGT
TGGCAATATGTCTACGC
</sequence>
<sequence>
<taxon idref="Albocalyci"/>
T-------------------------------------
TCCTGAATGCTTCATTATGTGAATAAGATAT------AGA-T-----------------
GCATGGAGT-ACATAC-TC-
AGTTATTCTAGACTATCAATATATAAA--------------------
TAAG---------------
AAAGTAGACTCATATTGGCTAGTGGCTTCTTCCGGAATGAGAAAGTGAGTTTCACTATCGAT
TCTAGGAT-AAAATG---ATTTATT--------
GATCGTTTTAAAATAGGATTTCAATACATCAATTCGACCCCAA-TTTCTTTT-----
ATGAAATTGATACTC------------ATCAGAACCAAA-
TTCACTTGATAGATGTACCTACCAATTCAAGAAAGATTCCAAGATATTTCA-----------
--------TTAAAAAATGGGATGGAGAAGTTCGATTTGTCCACACAAATTCTTAGATC-
AAAAAA----------TTTCCGAAACTCTCTTCATCCTTC----TTACT------
AGCTTTATCATCCCAGATTTCTTCTTTATTC-------ATTTT------
CTGGTATAGATATTTCGTTTTT-----------------CTAGATTCTA-GAAA---T-
CTATTTTTT----GTT-----ATT-CAAAAGTGAGTTATATAACAAATC-
AAAAAAGAAA---
CTCATTTTTT--

CGA------
TTTCGGTTCTTACACAATCTTGACAATTTCAAAAAAACGGGTCATACTATGATCATAGTATG
ATGGCGGTTGGACACGTATGCCCCCATCGTCTAGTGGTTCAGGACATCTCTCTTTCAAGGAG
GCAGCGGGGATTCGACTTCCCCTGGGGG-TAGGTTACTACA-
AAAGAAAGTGAATCATGCATTATGAATAA-------GCCGAAA----
TTGGAATTTATTCTTCCTGGGTCGATGCCCGAGCGGTTAATGGGGACGGACTGTAAATTCGT
TGGCAATATGTCTACGC
</sequence>
<sequence>
<taxon idref="Avenaceumm"/>
T---------------------------------
AAATTCCTGAATGCTTCATTATGTGAATAAGATAT------AGA-
T-----------------GCATGGAGT-ACATAC-TC-
AGTTATTCTAGACTATCAATATATAAA--------------------
TAAG---------------
AAAGTAGACTCATATTGGCTAGTGGCTTCTTCCGGAATGAGAAAGTGAGTTTCACTATCGAT
TCTAGGAT-AAAATG---ATTTATT--------
GATCGTTTTAAAATAGGATTTCAATACATCAATTCGACCCCAA-TTTCTTTT-----
ATGAAATTGATACTC------------ATCAGAACCAAA-
TTCACTTGATAGATGTACCTACCAATTCAAGAAAGATTCCAAGATATTTCA-----------
--------TTAAAAAATGGGATGGAGAAGTTCGATTTGTCCACACAAATTCTTAGATC-
AAAAAA----------TTTCCGAAACTCTCTTCATCCTTC----TTACT------

AGCTTTATCATCCCAGATTTCTTCTTTATTC-------ATTTT------
CTGGTATAGATATTTCGTTTTT-----------------CTAGATTCTA-GAAA---T-
CTATTTTTT----GTT-----ATT-CAAAAGTGAGTTATATAACAAATC-
AAAAAAGAAA---
CTCATTTTTT--

CGA------
TTTCGGTTCTTACACAATCTTGACAATTTCAAAAAAACGGGTCATACTATGATCATAGTATG
ATGGCGGTTGGACACGTATGCCCCCATCGTCTAGTGGTTCAGGACATCTCTCTTTCAAGGAG
GCAGCGGGGATTCGACTTCCCCTGGGGG-TAGGTTACTACA-
AAAGAAAGTGAATCATGCATTATGAATAA-------GCCGAAA----
TTGGAATTGATTCTTCCTGGGTCGATGCCCGAGCGGTTAATGGGGACGGACTGTAAATTCGT
TGGCAATATGTCTACGC
</sequence>
<sequence>
<taxon idref="Modestum1a"/>
T-------------------------------------
TCCTGAATGCTTCATTATGTGAATAAGATAT------AGA-T-----------------
GCATGGAGT-ACATAC-TC-
AGTTATTCTAGACTATCAATATATAAA--------------------
TAAG---------------
AAAGTAGACTCATATTGGCTAGTGGCTTCTTCCGGAATGAGAAAGTGAGTTTCACTATCGAT
TCTAGGAT-AAAATG---ATTTATT--------
GATCGTTTTAAAATAGGATTTCAATACATCAATTCGACCCCAA-TTTCTTTT-----
ATGAAATTGATACTC------------ATCAGAACCAAA-
TTCACTTGATAGATGTACCTACCAATTCAAG-----
TTCCAAGATATTTCA-------------------
TTAAAAAATGGGATGGAGAAGTTCGATTTGTCCACACAAATTCTTAGATC-
AAAAAA----------TTTCCGAAACTCTCTTCATCCTTC----TTACT------
AGCTTTATCATCCCAGATTTCTTCTTTATTC-------ATTTT------
CTGGTATAGATATTTCGTTTTT-----------------CTAGATTCTA-GAAA---T-
CTATTTTTT----GTT-----ATT-CAAAAGTGAGTTATATAACAAATC-
AAAAAAGAAA---
CTCATTTTTT--

CGA------
TTTCGGTTCTTACACAATCTTGACAATTTCAAAAAAACGGGTCATACTATGATCATAGTATG
ATGGCGGTTGGACACGTATGCCCCCATCGTCTAGTGGCTCAGGACATCTCTCTTTCAAGGAG
GCAGCGGGGATTCGACTTCCCCTGGGGGGTAGGTTACTACACAAAGAAAGTGAATCATGCAT
TATGAATAA-------GCCGAAA----
TTGGAATTTATTCTTCCTGGGTCGATGCCCGAGCGGTTAATGGGGACGGACTGTAAATTCGT
TGGCAATATGTCTACGC
</sequence>
<sequence>
<taxon idref="Oliganthum"/>
T-------------------------------------
TCCTGAATGCTTCATTATGTGAATAAGATAT------AGA-T------------
TAGATGCATGGAGT-----AC-TC-
AGTTATTCTAGACTATCAATATATAAA--------------------
TAAG---------------AAA----
ACTCATATTGGCTAGTGGCTTCTTCCGGAATGAGAAAGTGAATTTCACTATCGATTCTAGGA
T-AAAATG---ATTTATT--------

GATCGTTTTAAAATAGGATTTCAATACATCAATTCGACCCCAA-TTTCTTTG-----
ATGAAATTGATACTC-----------ATCAGAACCAAA-
TTCACTTGATAGATGTACCTACCAATTCAAGAAAGATTCCAAGATATTTCA----------
--------TTAAAAAATGGGATGGAGAAGTTCGATTTGTCCACACAAATTCTTAGATC-
AAAAAA---------TTTCCGAAACTCTCTTCATCCTTC----TTACT------
AGCTTTATCATCCCAGATTTCTTCTTTATTC-------ATTTT------
CTGGTATAGATATTTCGTTTTT----------------CTAGATTCTA-GAAA---T-
CTATTTTTTT---TTT-----ATT-CAAAAGTGAGTTATATAACAAATC-
AAAAAAGAAA---------------------------------------
CTCATTTTTT--

CGA------
TTTCGGTTCTTACACAATCTTGACAATTTCAAAAAAACGGATCATACTATGATCATAGTATG
ATGGCGGTTGGACACGTATGCCCCCATCGTCTAGTGGTTCAGGACATCTCTCTTTCAAGGAG
GCAGCGGGGATTCGACTTCCCCTGGGGG-TAGGTTACTACA-
AAAGAAAGTGAATCATGCATTATGAATAA-------GCCGAAA----
TTGGAATTTATTCTTGCTGGGTCGATGCCCGAGCGGTTAATGGGGACGGACTGTAAATTCGT
TGGCAATATGTCTACGC
</sequence>
<sequence>
<taxon idref="Blandummmm"/>
T-----------------------------------
TCCTGAATGCTTCATTATGTGAATAAGATAT------AGA-T----------------
GCATGGAGT-ACATAC-TC-
AGTTATTCTAGACTATCAATATATAAA-------------------
TAAG--------------
AAAGTAGACTCATATTGGCTAGTGGCTTCTTCCGGAATGAGAAAGTGAGTTTCACTATCGAT
TCTAGGAT-AAAATG---ATTTATT--------
GATCGTTTTAAAATAGGATTTCAATACATCAATTCGACCCCAA-TTTCTTTT-----
ATGAAATTGATACTC-----------ATCAGAACCAAA-
TTCACTTGATAGATGTACCTACCAATTCAAGAAAGATTCCAAGATATTTCA----------
--------TTAAAAAATGGGATGGAGAAGTTCGATTTGTCCACACAAATTCTTAGATC-
AAAAAA---------TTTCCGAAACTCTCTTCATCCTTC----TTACT------
AGCTTTATCATCCCAGATTTCTTCTTTATTC-------ATTTT------
CTGGTATAGATATTTCGTTTTT----------------CTAGATTCTA-GAAA---T-
CTATTTTTT----GTT-----ATT-CAAAAGTGAGTTATATAACAAATC-
AAAAAAGAAA---------------------------------------
CTCATTTTTT--

CGA------
TTTCGGTTCTTACACAATCTTGACAATTTCAAAAAAACGGGTCATACTATGATCATAGTATG
ATGGCGGTTGGACACGTATGCCCCCATCGTCTAGTGGTTCAGGACATCTCTCTTTCAAGGAG
GCAGCGGGGATTCGACTTCCCCTGGGGG-TAGGTTACTACA-
AAAGAAAGTGAGTCATGCATTATGAATAA-------GCCGAAG----
TTGGAATTTATTCTTCCTGGGTCGATGCCCGAGCGGTTAATGGGGACGGACTGTAAATTCGT
TGGCAATATGTCTACGC
</sequence>
<sequence>
<taxon idref="Raddeanumm"/>
T-----------------------------------
TCCTGAATGCTTCATTATGTGAATAAGATAT------AGA-T----------------
GCATGGAGT-ACATAC-TC-

AGTTATTCTAGACTATCAATATATAAA--------------------
TAAG---------------
AAAGTAGACTCATATTGGCTAGTGGCTTCTTCCGGAATGAGAAAGTGAGTTTCACTATCGAT
TCTAGGAT-AAAATG---ATTTATT--------
GATCGTTTTAAAATAGGATTTCAATACATCAATTCGACCCCAA-TTTCTTTT-----
ATGAAATTGATACTC------------ATCAGAACCAAA-
TTCACTTGATAGATGTACCTACCAATTCAAGAAAGATTCCAAGATATTTCA-----------
--------TTAAAAAATGGATGGAGAAGTTCGATTTGTCCACACAAATTCTTAGATC-
AAAAAA----------TTTCCGAAACTCTCTTCATCCTTC----TTACT------
AGCTTTATCATCCCAGATTTCTTCTTTATTC-------ATTTT------
CTGGTATAGATATTTCGTTTTT-----------------CTAGATTCTA-GAAA---T-
CTATTTTTT----GTT-----ATT-CAAAAGTGAGTTATATAACAAATC-
AAAAAAGAAA---
CTCATTTTT---

CGA------
TTTCGGTTCTTACACAATCTTGACAATTTCAAAAAAACGGGTCATACTATGATCATAGTATG
ATGGCGGTTGGACACGTATGCCCCCATCGTCTAGTGGTTCAGGACATCTCTCTTTCAAGGAG
GCAGCGGGGATTCGACTTCCCCTGGGGG-TAGGTTACTACA-
AAAGAAAGTGAATCATGCATTATGAATAA-------GCCGAAA----
TTGGAATTTATTCTTCCTGGGTCGATGCCCGAGCGGTTAATGGGGACGGACTGTAAATTCGT
TGGCAATATGTCTACGC
</sequence>
<sequence>
<taxon idref="Sirchensee"/>
T-------------------------------------
TCCTGAATGCTTCATTATGTGAATAAGATAT------AGA-T-----------------
GCATGGAGT-ACATAC-TC-
AGTTATTCTAGACTATCAATATATAAA--------------------
TAAG---------------
AAAGTAGACTCATATTGGCTAGTGGCTTCTTCCGGAATGAGAAAGTGAGTTTCACTATCGAT
TCTAGGAT-AAAATG---ATTTATT--------
GATCGTTTTAAAATAGGATTTCAATACATCAATTCGACCCCAA-TTTCTTTT-----
ATGAAATTGATACTC------------ATCAGAACCAAA-
TTCACTTGATAGATGTACCTACCAATTCAAGAAAGATTCCAAGATATTTCA-----------
--------TTAAAAAATGGATGGAGAAGTTCGATTTGTCCACACAAATTCTTAGATC-
AAAAAA----------TTTCCGAAACTCTCTTCATCCTTC----TTACT------
AGCTTTATCATCCCAGATTTCTTCTTTATTC-------ATTTT------
CTGGTATAGATATTTCGTTTTT-----------------CTAGATTCTA-GAAA---T-
CTATTTTTT----GTT-----ATT-CAAAAGTGAGTTATATAACAAATC-
AAAAAAGAAA---
CTCATTTTT---

CGA------
TTTCGGTTCTTACACAATCTTGACAATTTCAAAAAAACGGGTCATACTATGATCATAGTATG
ATGGCGGTTGGACACGTATGCCCCCATCGTCTAGTGGTTCAGGACATCTCTCTTTCAAGGAG
GCAGCGGGGATTCGACTTCCCCTGGGGG-TAGGTTACTACA-
AAAGAAAGTGAATCATGCATTATGAATAA-------GCCGAAA----
TTGGAATTTATTCTTCCTGGGTCGATGCCCGAGCGGTTAATGGGGACGGACTGTAAATTCGT
TGGCAATATGTCTACGC
</sequence>
<sequence>

<taxon idref="Collareeee"/>
T-------------------------------------
TCCTGAATGCTTCATTATGTGAATAAGATAT------AGA-T-----------------
GCATGGAGT-ACATAC-TC-
AGTTATTCTAGACTATCAATATATAAA--------------------
TAAG---------------
AAAGTAGACTCATATTGGCTAGTGGCTTCTTCCGGAATGAGAAAGTGAATTTCACTATCGAT
TCTAGGAT-AAAATG---ATTTATT--------
GATCGTTTTAAAATAGGATTTCAATACATCAATTCGACCCCAA-TTTCTTTT-----
ATGAAATTGATACTC------------ATCAGAACCAAA-
TTCACTTGATAGATGTACCTACCAATTCAAGAAAGATTCCAAGATATTTCA-----------
--------TTAAAAAATGGGATGGAGAAGTTCGATTTGTCCACACAAATTCTTAGATC-
AAAAAA----------TTTCCGAAACTCTCTTCATCCTTC----TTACT------
AGCTTTATCATCCCAGATTTCTTCTTTATTC-------ATTTT------
CTGGTATAGATATTTCGTTTTT-----------------CTAGATTCTA-GAAA---T-
CTATTTTTT----GTT-----ATT-CAAAAGTGAGTTATATAACAAATC-
AAAAAAGAAA---
CTCATTTTTT--

CGA------
TTTCGGTTCTTACACAATCTTGACAATTTCAAAAAAACGGGTCATACTATGATCATAGTATG
ATGGCGGTTGGACACGTATGCCCCCATCGTCTAGTGGTTCAGGACATCTCTCTTTCAAGGAG
GCAGCGGGGATTCGACTTCCCCTGGGGG-TAGGTTACTACA-
AAAGAAAGTGAATCATGCATTATGAATAA-------GCCGAAA----
TTGGAATTTATTCTTCCTGGGTCGATGCCCGAGCGGTTAATGGGGACGGACTGTAAATTCGT
TGGCAATATGTCTACGC
</sequence>
<sequence>
<taxon idref="Heweriiiii"/>
T-------------------------------------
TCCTGAATGCTTCATTATGTGAGTAAGATAT------AGA-T-----------------
GCATGGAGT-ACATAC-TC-
AGTTATTCTAGACTATCAATATATAAA--------------------
TAAG---------------
AAAGTAGACTCATATTGGCTAGTGGCTTCTTCCGGAATGAGAAAGTGAATTTCACTATCGAT
TCTAGGAT-AAAATG---ATTTATT--------
GATCGTTTTAAAATAGGATTTCAATACATCAATTCGACCCCAA-TTTCTTTT-----
ATGAAATTGATACTC------------ATCAGAACCAAA-
TTCACTTGATAGATGTACCTACCAATTCAAGAAAGATTCCAAGATATTTCA-----------
--------TTAAAAAATGGGATGGAGAAGTTCGATTTGTCCACACAAATTCTTAGATC-
AAAAAA----------TTTCCGAAACTCTCTTCATCCTTC----TTACT------
AGCTTTATCATCCCAGATTTCTTCTTTATTC-------ATTTT------
CTGGTATAGATATTTCGTTTTT-----------------CTAGATTCTA-GAAA---T-
CTATTTTTT----GTT-----ATT-CAAAAGTGAGTTATATAACAAATC-
AAAAAAGAAA---
CTCATTTTTT--

CGA------
TTTCGGATCTTACACAATCTTGACAATTTCAAAAAAACGGGTCATACTATGATCATAGTATG
ATGGCGGTTGGACACGTATGCCCCCATCGTCTAGTGGTTCAGGACATCTCTCTTTCAAGGAG
GCAGCGGGGATTCGACTTCCCCTGGGGG-TAGGTTACTACA-
AAAGAAAGTGAATCATGCATTATGAATAA-------GCCGAAA----

TTGGAATTTATTCTTCCTAGGTCGATGCCCGAGCGGTTAATGGGGACGGACTGTAAATTCGT
TGGCAATATGTCTACGC
</sequence>
<sequence>
<taxon idref="Restiacumm"/>

T--

</sequence>
<sequence>
<taxon idref="Spinicaly1"/>
T-------------------------------------
TCCTGAATGCTTCATTATGTGAATAAGATAT------AGA-T-----------------
GCATGGAGT-ACATAC-TC-
AGTTATTCTAGACTATCAATATATAAA--------------------
TAAG---------------
AAAGTAGACTCATATTGGCTAGTGGCTTCTTCCGGAATGAGAAAGTGAATTTCACTATCGAT
TCTAGGAT-AAAATG---ATTTATT--------
GATCGTTTTAAAATAGGATTTCAATACATCAATTCGACCCCAA-TTTCTTTT-----
ATGAAATTGATACTC------------ATCAGAACCAAA-
TTCACTTGATAGATGTACCTACCAATTCAAGAAAGATTCCAAGATATTTCA-----------
--------TTAAAAAATGGGATGGAGAAGTTCGATTTGTCCACACAAATTCTTAGATCC-
AAAAA----------TTTCCGAAACTCTCTTCATCCTTC----TTACT------
AACTTTATCATCCCAGATTTCTTCTTTATTC-------ATTTT------
CTGGTATAGATATTTCGTTTTT-----------------CTAGATTCTA-GAAA---T-
CTATTTTTT----GTT-----ATT-CAAAAGTGAGTTATATAACAAATC-
AAAAAAGAAA---
CTCATTTTTT--

CGA------
TTTCGGTTCTTACACAATCTTGACAATTTCAAAAAAAGGGGTCATACTATGATCATAGTATG
ATGGCGGTTGGACACGTATGCCCCCATCGTCTAGTGGTTCAGGACATCTCTCTTTCAAGGAG
GCAGCGGGGATTCGACTTCCCCTGGGGG-TAGGTTACTACA-
AAAGAAAGTGAATCATGCATTATGAATAA-------GCCGAAA----

TTGGAATTTATTCTTCCTGGGTCGATGCCCGAGCGGTTAATGGGGACGGACTGTAAATTCGT
TGGCAATATGTCTACGC
</sequence>
<sequence>
<taxon idref="Gulistanum"/>
T-------------------------------------
TCCTGAATGCTTCATTATGTGAATAAGATAT------AGA-T-----------------
GCATGGAGT-ACATAC-TC-
AGTTATTCTAGACTATCAATATATAAA--------------------
TAAG---------------
AAAGTAGACTCATATTGGCTAGTGGCTTCTTCCGGAATGAGAAAGTGAGTTTCACTATCGAT
TCTAGGAT-AAAATG---ATTTATT--------
GATCGTTTTAAAATAGGATTTCAATACATCAATTCGACCCCAA-TTTCTTTT-----
ATGAAATTGATACTC------------ATCAGAACCAAA-
TTCACTTGATAGATGTACCTACCAATTCAAGAAAGATTCCAAGATATTTCA-----------
--------TTAAAAAATGGGATGGAGAAGTTCGATTTGTCCACACAAATTCTTAGATC-
AAAAAA----------TTTCCGAAACTCTCTTCATCCTTC----TTACT------
AGCTTTATCATCCCAGATTTCTTCTTTATTC-------ATTTT------
CTGGTATAGATATTTCGTTTTT-----------------CTAGATTCTA-GAAA---T-
CTATTTTTT----GTT-----ATT-CAAAAGTGAGTTAGATAACAAATC-
AAAAAAGAAA---
CTCATTTTT---

CGA------
TTTCGGTTCTTACACAATCTTGACAATTTCAAAAAAACGGGTCATACTATGATCATAGTATG
ATGGCGGTTGGACACGTATGCCCCCATCGTCTAGTGGCTCAGGACATCTCTCTTTCAAGGAG
GCAGCGGGGATTCGACTTCCCCTGGGGGGTAGGTTACTACA-
AAAGAAAGTGAATCATGCATTATGAATAA-------GCCGAAA----
TTGGAATTTATTCTTCCTGGGTCGATGCCCGAGCGGTTAATGGGGACGGACTGTAAATTCGT
TGGCAATATGTCTACGC
</sequence>
<sequence>
<taxon idref="Austroiran"/>
T---------------
TAAAAGGGCCTTTTTCGTAAATTCCTGAATGCTTCATTATGTGAATAAGATAT------
AGA-T-----------------GCATGGAGT-ACATAC-TC-
AGTTATTCTAGACTATCAATATATAAA--------------------
TAAG---------------
AAAGTAGACTCATATTGGCTAGTGGCTTCTTCCGGAATGAGAAAGTGAGTTTCACTATCGAT
TCTAGGAT-AAAATG---ATTTATT--------
GATCGTTTTAAAATAGGATTTCAATACATCAATTCGACCCCAA-TTTCTTTT-----
ATGAAATTGATACTC------------ATCAGAACCAAA-
TTCACTTGATAGATGTACCTACCAATTCAAGAAAGATTCCAAGATATTTCA-----------
--------TTAAAAAATGGGATGGAGAAGTTCGATTTGTCCACACAAATTCTTAGATC-
AAAAAA----------TTTCCGAAACTCTCTTCATCCTTC----TTACT------
AGCTTTATCATCCCAGATTTCTTCTTTATTC-------ATTTT------
CTGGTATAGATATTTCGTTTTT-----------------CTAGATTCTA-GAAA---T-
CTATTTTTT----GTT-----ATT-CAAAAGTGAGTTATATAACAAATC-
AAAAAAGAAA---
CTCATTTTT---

CGA------

TTTCGGTTCTTACACAATCTTGACAATTTCAAAAAAACGGGTCATACTATGATCATAGTATG
ATGGCGGTTGGACACGTATGCCCCCATCGTCTAGTGGTTCAGGACATCTCTCTTTCAAGGAG
GCAGCGGGGATTCGACTTCCCTGGGGG-TAGGTTACTACA-
AAAGAAAGTGAATCATGCATTATGAATAA-------GCCGAAA----
TTGGAATTTATTCTTCCTGGGTCGATGCCCGAGCGGTTAATGGGGACGGACTGTAAATTCGT
TGGCAATATGTCTACGC
</sequence>
<sequence>
<taxon idref="Schahrudic"/>
T-------------------------------------
TCCTGAATGCTTCATTATGTGAATAAGATAT------AGA-T------------------
GCATGGAGT-ACATAC-TC-
AGTTATTCTAGACTATCAATATATAAA--------------------
TAAG---------------
AAAGTAGACTCATATTGGCTAGTGGCTTCTTCCGGAATGAGAAAGTGAGTTTCACTATCGAT
TCTAGGAT-AAAATG---ATTTATT--------
GATCGTTTTAAAATAGGATTTCAATACATCAATTCGACCCCAA-TTTCTTTT-----
ATGAAATTGATACTC------------ATCAGAACCAAA-
TTCACTTGATAGATGTACCTACCAATTCAAGAAAGATTCCAAGATATTTCA-----------
--------TTAAAAAATGGATGGAGAAGTTCGATTTGTCCACACAAATTCTTAGATC-
AAAAAA----------TTTCCGAAACTCTCTTCATCCTTC----TTACT------
AGCTTTATCATCCCAGATTTCTTCTTTATTC-------ATTTT------
CTGGTATAGATATTTCGTTTTT-----------------CTAGATTCTA-GAAA---T-
CTATTTTTT----GTT-----ATT-CAAAAGTGAGTTATATAACAAATC-
AAAAAAGAAA---
CTCATTTTTT--

CGA------
TTTCGGTTCTTACACAATCTTGACAATTTCAAAAAAACGGGTCATACTATGATCATAGTATG
ATGGCGGTTGGACACGTATGCCCCCATCGTCTAGTGGTTCAGGACATCTCTCTTTCAAGGAG
GCAGCGGGGATTCGACTTCCCTGGGGG-TAGGTTACTACA-
AAAGAAAGTGAATCATGCATTATGAATAA-------GCCGAAA----
TTGGAATTTATTCTTCCTGGGTCGATGCCCGAGCGGTTAATGGGGACGGACTGTAAATTCGT
TGGCAATATGTCTACGC
</sequence>
<sequence>
<taxon idref="Cephaltoid"/>
T---------------
TAAAAGGGCCTTTTTCGTAAAGTCCTGAATGCTTCATTATGTGAATAAGATATTAGATTAGA
-T-----------------GCATGGAGT-ACATAC-TC-
AGTTATTCTAGACTATCAATATATAAA--------------------
TAAG---------------AAA----
ACTCATATTGGCTAGTGGCTTCTTCCGGAATGAGAAAGTGAATTTCACTATCGATTCTAGGA
T-AAAATG---ATTTATT--------
GATCGTTTTAAAATAGGATTTCAATACATCAATTCGACCCCAA-TTTCTTTG-----
ATGAAATTGATACTC------------ATCAGAACCAAA-
TTCACTTGATAGATGTACCTACCAATTCAAGAAAGATTCCAAGATATTTCA-----------
--------TTAAAAAATGGATGGAGAAGTTCGATTTGTCCACACAAATTCTTAGATC-
AAAAAA----------TTTCCGAAACTCTCTTCATCCTTC----TTACT------
AGCTTTATCATCCCAGATTTCTTCTTTATTC-------ATTTT------
CTGGTATAGATATTTCGTTTTT-----------------CTAGATTCTA-GAAA---T-
CTATTTTTT----GTT-----ATT-CAAAAGTGAGTTATATAACAAATC-
AAAAAAGAAA---
CTCATTTTTT--

CGA------
TTTCGGTTCTTACACAATCTTGACAATTTCAAAAAAACGGATCATACTATGATCATAGTATG
ATGGCGGTTGGACACGTATGCCCCCATCGTCTAGTGGTTCAGGACATCTCTCTTTCAAGGAG
GCAGCGGGGATTCGACTTCCCCTGGGGG-TAGGTTACTACA-
AAAGAAAGTGAATCATGCATTATGAATAA-------GCCGAAA----
TTGGAATTTATTCTTGCTGGGTCGATGCCCGAGCGGTTAATGGGGACGGACTGTAAATTCGT
TGGCAATATGTCTACGC
</sequence>
<sequence>
<taxon idref="Demawendic"/>
T-------------------------------------
TCCTGAATGCTTCATTATGTGAATAAGATATTAGATTAGA-T-----------------
GCATGGAGT-ACATAC-TC-
AGTTATTCTAGACTATCAATATATAAA--------------------
TAAG---------------AAA----
ACTCATATTGGCTAGTGGCTTCTTCCGGAATGAGAAAGTGAATTTCACTATCGATTCTAGGA
T-AAAATG---ATTTATT--------
GATCGTTTTAAAATAGGATTTCAATACATCAATTCGACCCCAA-TTTCTTTG-----
ATGAAATTGATACTC------------ATCAGAACCAAA-
TTCACTTGATAGATGTACCTACCAATTCAAGAAAGATTCCAAGATATTTCA-----------
--------TTAAAAAATGGGATGGAGAAGTTCGATTTGTCCACACAAATTCTTAGATC-
AAAAAA----------TTTCCGAAACTCTCTTCATCCTTC----TTACT------
AGCTTTATCATCCCAGATTTCTTCTTTATTC-------ATTTT------
CTGGTATAGATATTTCGTTTTT-----------------CTAGATTCTA-GAAA---T-
CTATTTTTT----GTT-----ATT-CAAAAGTGAGTTATATAACAAATC-
AAAAAAGAAA---
CTCATTTTTT--

CGA------
TTTCGGTTCTTACACAATCTTGACAATTTCAAAAAAACGGATCATACTATGATCATAGTATG
ATGGCGGTTGGACACGTATGCCCCCATCGTCTAGTGGTTCAGGACATCTCTCTTTCAAGGAG
GCAGCGGGGATTCGACTTCCCCTGGGGG-TAGGTTACTACA-
AAAGAAAGTGAATCATGCATTATGAATAA-------GCCGAAA----
TTGGAATTTATTCTTGCTGGGTCGATGCCCGAGCGGTTAATGGGGACGGACTGTAAATTCGT
TGGCAATATGTCTACGC
</sequence>
<sequence>
<taxon idref="Gorganense"/>
T-------------------------------------
TCCTGAATGCTTCATTATGTGAATAAGATAT------AGA-T-----------------
GCATGGAGT-ACATAC-TC-
AGTTATTCTAGACTATCAATATATAAA--------------------
TAAG---------------
AAAGTAGACTCATATTGGCTAGTGGCTTCTTCCGGAATGAGAAAGTGAGTTTCACTATCGAT
TCTAGGAT-AAAATG---ATTTATT--------
GATCGTTTTAAAATAGGATTTCAATACATCAATTCGACCCCAA-TTTCTTTT-----
ATGAAATTGATACTC------------ATCAGAACCAAA-
TTCACTTGATAGATGTACCTACCAATTCAAGAAAGATTCCAAGATATTTCA-----------
--------TTAAAAAATGGGATGGAGAAGTTCGATTTGTCCACACAAATTCTTAGATC-
AAAAAA----------TTTCCGAAACTCTCTTCATCCTTC----TTACT------
AGCTTTATCATCCCAGATTTCTTCTTTATTC-------ATTTT------

CTGGTATAGATATTTCGTTTTT-----------------CTAGATTCTA-GAAA---T-
CTATTTTTT----GTT-----ATT-CAAAAGTGAGTTATATAACAAATC-
AAAAAAGAAA---
CTCATTTTTT--

CGA------
TTTCGGTTCTTACACAATCTTGACAATTTCAAAAAAACGGGTCATACTATGATCATAGTATG
ATGGCGGTTGGACACGTATGCCCCCATCGTCTAGTGGTTCAGGACATCTCTCTTTCAAGGAG
GCAGCGGGGATTCGACTTCCCCTGGGGG-TAGGTTACTACA-
AAAGAAAGTGAATCATGCATTATGAATAA-------GCCGAAA----
TTGGAATTTATTCTTCCTGGGTCGATGCCCGAGCGGTTAATGGGGACGGACTGTAAATTCGT
TGGCAATATGTCTACGC
</sequence>
<sequence>
<taxon idref="Heratensee"/>
T-------------------------------------
TCCTGAATGCTTCATTATGTGAATAAGATAT------AGA-T-----------------
GCATGGAGT-ACATAC-TC-
AGTTATTCTAGACTATCAATATATAAA--------------------
TAAG---------------
AAAGTAGACTCATATTGGCTAGTGGCTTCTTCCGGAATGAGAAAGTGAATTTCACTATCGAT
TCTAGGAT-AAAATG---ATTTATT--------
GATCGTTTTAAAATAGGATTTCAATACATCAATTCGACCCCAA-TTTCTTTT-----
ATGAAATTGATACTC------------ATCAGAACCAAA-
TTCACTTGATAGATGTACCTACCAATTCAAGAAAGATTCCAAGATATTTCA-----------
--------TTAAAAAATGGGATGGAGAAGTTCGATTTGTCCACACAAATTCTTAGATCC-
AAAAA----------TTTCCGAAACTCTCTTCATCCTTC----TTACT------
AACTTTATCATCCCAGATTTCTTCTTTATTC-------ATTTT------
CTGGTATAGATATTTCGTTTTT-----------------CTAGATTCTA-GAAA---T-
CTATTTTTT----GTT-----ATT-CAAAAGTGAGTTATATAACAAATC-
AAAAAAGAAA---
CTCATTTTTT--

CGA------
TTTCGGTTCTTACACAATCTTGACAATTTCAAAAAAAGGGGTCATACTATGATCATAGTATG
ATGGCGGTTGGACACGTATGCCCCCATCGTCTAGTGGTTCAGGACATCTCTCTTTCAAGGAG
GCAGCGGGGATTCGACTTCCCCTGGGGGGTAGGTTACTACA-
AAAGAAAGTGAATCATGCATTATGAATAA-------GCCGAAA----
TTGGAATTTATTCTTCCTGGGTCGATGCCCGAGCGGTTAATGGGGACGGACTGTAAATTCGT
TGGCAATATGTCTACGC
</sequence>
<sequence>
<taxon idref="Horridummm"/>
T-------------------------------------
TCCTGAATGCTTCATTATGTGAATAAGATAT------AGA-T-----------------
GCATGGAGT-ACATAC-TC-
AGTTATTCTAGACTATCAATATATAAA--------------------
TAAG---------------
AAAGTAGACTCATATTGGCTAGTGGCTTCTTCCGGAATGAGAAAGTGAGTTTCACTATCGAT
TCTAGGAT-AAAATG---ATTTATT--------
GATCGTTTTAAAATAGGATTTCAATACATCAATTCGACCCCAA-TTTCTTTT-----
ATGAAATTGATACTC------------ATCAGAACCAAA-

TTCACTTGATAGATGTACCTACCAATTCAAGAAAGATTCCAAGATATTTCA-----------
--------TTTAAAAATGGATGGAGAAGTTCGATTTGTCCACACAAATTCTTAGATC-
AAAAAA----------TTTCCGAAACTCTCTTCATCCTTC----TTACT------
AGCTTTATCATCCCAGATTTCTTCTTTATTC-------ATTTT------
CTGGTATAGATATTTCGTTTTT-----------------CTAGATTCTA-GAAA---T-
CTATTTTTT----GTT-----ATT-CAAAAGTGAGTTATATAACAAATC-
AAAAAAGAAA---
CTCATTTTTT--

CGA------
TTTCGGTTCTTACACAATCTTGACAATTTCAAAAAAACGGGTCATACTATGATCATAGTATG
ATGGCGGTTGGACACGTATGCCCCCATCGTCTAGTGGTTCAGGACATCTCTCTTTCAAGGAG
GCAGCGGGGATTCGACTTCCCCTGGGGGGTAGGTTACTACA-
AAAGAAAGTGAATCATGCATTATGAATAA-------GCCGAAA----
TTGGAATTTATTCTTCCTGGGTCGATGCCCGAGCGGTTAATGGGGACGGACTGTAAATTCGT
TGGCAATATGTCTACGC
</sequence>
<sequence>
<taxon idref="Rodopolium"/>
T-------------------------------------
TCCTGAATGCTTCATTATGTGAATAAGATAT------AGA-T------------------
GCATGGAGT-----AC-TC-
AGTTATTCTAGACTATCAATATATAAA--------------------
TAAG---------------
AAAGTAGACTCATATTGGCTAGTGGCTTCTTCCGGAATGAGAAAGTGAATTTCACTATCGAT
TCTAGGAT-AAAATG---ATTTATT--------
GATCGTTTTAAAATAGGATTTCAATACATCAATTCGACCCCAA-TTTCTTTT-----
ATGAAATTGATACTC------------ATCAGAACCAAA-
TTCACTTGATAGATGTACCTACCAATTCAAGAAAGATTCCAAGATATTTCA-----------
--------TTAAAAAATGGATGGAGAAGTTCGATTTGTCCACACAAATTCTTAGATC-
AAAAAA----------TTTCCGAAACTCTCTTCATCCTTC----TTACT------
AGCTTTATCATCCCAGATTTCTTCTTTATTC-------ATTTT------
CTGTTATAGATATTTCGTTTTT-----------------CTAGATTCTA-GAAA---T-
CTATTTTTT----GTT-----ATT-CACAAGTGAGTTATATAACAAATC-
AAAAAAGAAA---
CTCATTTTTT--

CGA------
TTTCGGTTCTTACACAATCTTGACAATTTCAAAAAAACGGGTCATACTATGATCATAGTATG
ATGGCGGTTGGACACGTATGCCCCCATCGTCTAGTGGTTCAGGACATCTCTCTTTCAAGGAG
GCAGCGGGGATTCGACTTCCCCTGGGGG-TAGGTTACTACA-
AAAGAAAGTGAATCATGCATTATGAATAA-------GCCGAAA----
TTGGAATTTATTCTTCCTGGGTCGATGCCCGAGCGGTTAATGGGGACGGACTGTAAATTCGT
TGGCAATATGTCTACGC
</sequence>
<sequence>
<taxon idref="Tragacanth"/>
T-------------------------------------
TCCTGAATGCTTCATTATGTGAATAAGATAT------AGA-T------------------
GCATGGAGT-ACATAC-TC-
AGTTATTCTAGACTATCAATATATAAA--------------------
TAAG---------------

AAAGTAGACTCATATTGGCTAGTGGCTTCTTCCGGAATGAGAAAGTGAATTTCACTATCGAT
TCTAGGAT-AAAATG---ATTTATT--------
GATCGTTTTAAAATAGGATTTCAATACATCAATTCGACCCCAA-TTTCTTTT-----
ATGAAATTGATACTC------------ATCAGAACCAAA-
TTCACTTGATAGATGTACCTACCAATTCAAGAAAGATTCCAAGATATTTCA-----------
--------TTAAAAAATGGGATGGAGAAGTTCGATTTGTCCACACAAATTCTTAGATC-
AAAAAA----------TTTCCGAAACTCTCTTCATCCTTC----TTACT------
AGCTTTATCATCCCAGATTTCTTCTTTATTC-------ATTTT------
CTGGTATAGATATTTCGTTTTT-----------------CTAGATTCTA-GAAA---T-
CTATTTTTT----GTT-----ATT-CAAAAGTGAGTTATATAACAAATC-
AAAAAAGAAA---------------------------------------
CTCATTTTTT--

CGA------
TTTCGGTTCTTACATAATCTTGACAATTTCAAAAAAACGGGTCATACTATGATCATAGTATG
ATGGCGGTTGGACACGTATGCCCCCATCGTCTAGTGGTTCAGGACATCTCTCTTTCAAGGAG
GCAGCGGGGATTCGACTTCCCCTGGGGG-TAGGTTACTAGA-
AAAGAAAGTGAATCATGCATTATGAATAA-------GCCGAAA----
TTGGAATTTATTCTTCCTGGGTCGATGCCCGAGCGGTTAATGGGGACGGACTGTAAATTCGT
TGGCAATATGTCTACGC
</sequence>
<sequence>
<taxon idref="Atropatanu"/>
T----------------------------------
AAAGTCCTGAATGCTTCATTATGTGAATAAGATAT------AGA-T------------
TAGATGCATGGAGT-----AC-TC-
AGTTATTCTAGACTATCAATATATAAA--------------------
TAAG---------------AAA----ACTCATATTGGCTAGT-----
CTTCCGGAATGAGAAAGTGAATTTCACTATCGATTCTAGGAT-AAAATG---
ATTTATT--------GATCGTTTTAAAATAGGATTTCAATACATCAATTCGACCCCAA-
TTTCTTTG-----ATGAAATTGATACTC------------ATCAGAACCAAA-
TTCACTTGATAGATGTACCTACCAATTCAAGAAAGATTCCAAGATATTTCA-----------
--------TTAAAAAATGGGATGGAGAAGTTCGATTTGTCCACACAAATTCTTAGATCC-
AAAAA----------TTTCCGAAACTCTCTTCATCCTTC----TTACT------
AGCTTTATCATCCCAGATTTCTTCTTTATTC-------ATTTT------
CTGGTATAGATATTTCGTTTTT-----------------CTAGATTCTA-GAAA---T-
CGATTTTTT----GTT-----ATT-CAAAAGTGAGTTATATAACAAATC-
AAAAAAGAAA---------------------------------------
CTCATTTTTT--

CGA------
TTTCGGTTCTTACACAATCTTGACAATTTCAAAAAAACGGATCATACTATGATCATAGTATG
ATGGCGGTTGGACACGTATGCCCCCATCGTCTAGTGGTTCAGGACATCTCTCTTTCAAGGAG
GCAGCGGGGATTCGACTTCCCCTGGGGG-TAGGTTACTACA-
AAAGAAAGTGAATCATGCATTATGAATAA-------GCCGAAA----
TTGGAATTTATTCTTGCTGGGTCGATGCCCGAGCGGTTAATGGGGACGGACTGTAAATTCGT
TGGCAATATGTCTACGC
</sequence>
<sequence>
<taxon idref="Sahendicu2"/>

TAAAGGGCCTTTGAGTTAAAAGGGCCTTTTTCGTAAAGTCCTGAATGCTTCATTATG

TGAATAAGATAT------AGA-T------------TAGATGCATGGAGT-----AC-TC-
AGTTATTCTAGACTATCAATATATAAA--------------------
TAAG---------------AAA----
ACTCATATTGGCTAGTGGCTTCTTCCGGAATGAGAAAGTGAATTTCACTATCGATTCTAGGA
T-AAAATG---ATTTATT--------
GATCGTTTTAAAATAGGATTTCAATACATCAATTCGACCCCAA-TTTCTTTG-----
ATGAAATTGATACTC------------ATCAGAACCAAA-
TTCACTTGATAGATGTACCTACCAATTCAAGAAAGATTCCAAGATATTTCA-----------
--------TTAAAAAATGGGATGGAGAAGTTCGATTTGTCCACACAAATTCTTAGATCC-
AAAAA----------TTTCCGAAACTCTCTTCATCCTTC----TTACT------
AGCTTTATCATCCCAGATTTCTTCTTTATTC-------ATTTT------
CTGGTATAGATATTTCGTTTTT-----------------CTAGATTCTA-GAAA---T-
CGATTTTTT----GTT-----ATT-CAAAAGTGAGTTATATAACAAATC-
AAAAAAGAAA---
CTCATTTTTT--

CGA------
TTTCGGTTCTTACACAATCTTGACAATTTCAAAAAAACGGATCATACTATGATCATAGTATG
ATGGCGGTTGGACACGTATGCCCCCATCGTCTAGTGGTTCAGGACATCTCTCTTTCAAGGAG
GCAGCGGGGATTCGACTTCCCCTGGGGG-TAGGTTACTACA-
AAAGAAAGTGAATCATGCATTATGAATAA-------GCCGAAA----
TTGGAATTTATTCTTGCTGGGTCGATGCCCGAGCGGTTAATGGGGACGGACTGTAAATTCGT
TGGCTATATGTCTACGC
</sequence>
<sequence>
<taxon idref="Latifolium"/>
T--------------------------------------
TCCTGAATGCTTCATTATGTGAATAAGATAT------AGA-T------------
TAGATGCATGGAGT-----AC-TC-
AGTTATTCTAGACTATCAATATATAAA--------------------
TAAG---------------AAA----
ACTCATATTGGCTAGTGGCTTCTTCCGGAATGAGAAAGTGAATTTCACTATCGATTCTAGGA
T-AAAATG---ATTTATT--------
GATCGTTTTAAAATAGGATTTCAATACATCAATTCGACCCCAA-TTTCTTTG-----
ATGAAATTGATACTC------------ATCAGAACCAAA-
TTCACTTGATAGATGTACCTACCAATTCAAGAAAGATTCCAAGATATTTCA-----------
--------TTCAAAAATGGGATGGAGAAGTTCGATTTGTCCACACAAATTCTTAGATC-
AAAAAA----------TTTCCGAAACTCTCTTCATCCTTC----TTACT------
AGCTTTATCATCCCAGATTTCTTCTTTATTC-------ATTTT------
CTGGTATAGATATTTTGTTTTT-----------------CTAGATTCTA-GAAA---T-
CTATTTTTT----GTT-----ATT-CAAAAGTGAGTTATATAACAAATC-
AAAAAAGAAA---
CTCATTTTTT--

CGA------
TTTCGGTTCTTACACAATCTTGACAATTTCAAAAAAACGGATCATACTATGATCATAGTATG
ATGGCGGTTGGACACGTATGCCCCCATCGTCTAGTGGTTCAGGACATCTCTCTTTCAAGGAG
GCAGCGGGGATTCGACTTCCCCTGGGGG-TAGGTTACTACA-
AAAGAAAGTGAATCATGCATTATGAATAA-------GCCGAAA----
TTGGAATTTATTCTTGCTGGGTCGATGCCCGAGCGGTTAATGGGGACGGACTGTAAATTCGT
TGGCAATATGTCTACGC
</sequence>

<sequence>
<taxon idref="Asphodelin"/>
T--------------------------------
AAAGTCCTGAATGCTTCATTATGTGAATAAGATAT------AGA-T------------
TAGATGCATGGAGT-----AC-TC-
AGTTATTCTAGACTATCAATATATAAA--------------------
TAAG---------------AAA----
ACTCATATTGGCTAGTGGCTTCTTCCGGAATGAGAAAGTGAATTTCACTATCGATTCTAGGA
T-AAAATG---ATTTATT--------
GATCGTTTTAAAATAGGATTTCAATACATCAATTCGACCCCAA-TTTCTTTG-----
ATGAAATTGATACTC------------ATCAGAACCAAA-
TTCACTTGATAGATGTACCTACCAATTCAAGAAAGATTCCAAGATATTTCA----------
--------TTAAAAAAATGGATGGAGAAGTTCGATTTGTCCACACAAATTCTTAGATC-
AAAAAA----------TTTCCGAAACTCTCTTCATCCTTC----TTACT------
AGCTTTATCATCCCAGATTTCTTCTTTATTC-------ATTTT------
CTGGTATAGATATTTCGTTTTT-----------------CTAGATTCTA-GAAA---T-
CTATTTTTTT---TTT-----ATT-CAAAAGTGAGTTATATAACAAATC-
AAAAAAGAAA---
CTCATTTTTT---

CGA------
TTTCGGTTCTTACACAATCTTGACAATTTCAAAAAAACGGATCATACTATGATCATAGTATG
ATGGCGGTTGGACACGTATGCCCCCATCGTCTAGTGGTTCAGGACATCTCTCTTTCAAGGAG
GCAGCGGGGATTCGACTTCCCCTGGGGG-TAGGTTACTACA-
AAAGAAAGTGAATCATGCATTATGAATAA-------GCCGAAA----
TTGGAATTTATTCTTGCTGGGTCGATGCCCGAGCGGTTAATGGGGACGGACTGTAAATTCGT
TGGCAATATGTCTACGC
</sequence>
<sequence>
<taxon idref="Acmostegi1"/>
T--------------------------------
AAATTCCTGAATGCTTCATTATGTGAATAAGATAT------AGA-
T-----------------GCATGGAGT-ACATAC-TC-
AGTTATTCTAGACTATCAATATATAAA--------------------
TAAG---------------
AAAGTAGACTCATATTGGCTAGTGGCTTCTTCCGGAATGAGAAAGTGAATTTCACTATCGAT
TCTAGGAT-AAAATG---ATTTATT--------
GATCGTTTTAAAATAGGATTTCAATACATCAATTCGACCCCAA-TTTCTTTT-----
ATGAAATTGATACTC------------ATCAGAACCAAA-
TTCACTTGATAGATGTACCTACCAATTCAAGAAAGATTCCAAGATATTTCA----------
--------TTAAAAAAATGGATGGAGAAGTTCGATTTGTCCACACAAATTCTTAGATC-
AAAAAA----------TTTCCGAAACTCTCTTCATCCTTC----TTACT------
AGCTTTATCATCCCAGATTTCTTCTTTATTC-------ATTTT------
CTGGTATAGATATTTCGTTTTT-----------------CTAGATTCTA-GAAA---T-
CTATTTTTT----GTT-----ATT-CAAAAGTGAGTTATATAACAAATC-
AAAAAAGAAA---
CTCATTTTTT---

CGA------
TTTCGGTTCTTACACAATCTTGACAATTTCAAAAAAACGGGTCATACTATGATCATAGTATG
ATGGCGGTTGGACACGTATGCCCCCATCGTCTAGTGGTTCAGGACATCTCTCTTTCAAGGAG
GCAGCGGGGATTCGACTTCCCCTGGGGG-TAGGTTACTACA-

AAAGAAAGTGAATCATGCATTATGAATAA-------GCCGAAA----
TTGGAATTTATTCTTCCTGGGTCGATGCCCGAGCGGTTAATGGGGACGGACTGTAAATTCGT
TGGCAATATGTCTACGC
</sequence>
<sequence>
<taxon idref="Brachystac"/>
T---------------------------------
AAAGTCCTGAATGCTTCATTATGTGAATAAGATAT------AGA-T------------
TAGATGCATGGAGT-----AC-TC-
AGTTATTCTAGACTATCAATATATAAA--------------------
TAAG---------------AAA----
ACTCATATTGGCTAGTGGCTTCTTCCGGAATGAGAAAGTGAATTTCACTATCGATTCTAGGA
T-AAAATG---ATTTATT--------
GATCGTTTTAAAATAGGATTTCAATACATCAATTCGACCCCAA-TTTCTTTG-----
ATGAAATTGATACTC------------ATCAGAACCAAA-
TTCACTTGATAGATGTACCTACCAATTCAAGAAAGATTCCAAGATATTTCA-----------
--------TTAAAAAATGGGATGGAGAAGTTCGATTTGTCCACACAAATTCTTAGATC-
AAAAAA----------TTTCCGAAACTCTCTTCATCCTTC----TTACT------
AGCTTTATCATCCCAGATTTCTTCTTTATTC-------ATTTT------
CTGGTATAGATATTTCGTTTTT-----------------CTAGATTCTA-GAAA---T-
CTATTTTTTT---TTT-----ATT-CAAAAGTGAGTTATATAACAAATC-
AAAAAAGAAA---
CTCATTTTTT--

CGA------
TTTCGGTTCTTACCCAATCTTGACAATTTCAAAAAAACGGATCATACTATGATCATAGTATG
ATGGCGGTTGGACACGTATGCCCCCATCGTCTAGTGGTTCAGGACATCTCTCTTTCAAGGAG
GCAGCGGGGATTCGACTTCCCCTGGGGG-TAGGTTACTACA-
AAAGAAAGTGAATCATGCATTATGAATAA-------GCCGAAA----
TTGGAATTTATTCTTGCTGGGTCGATGCCCGAGCGGTTAATGGGGACGGACTGTAAATTCGT
TGGCAATATGTCTACGC
</sequence>
<sequence>
<taxon idref="Caryophyll"/>
T---------------------------------
AAAGTCCTGAATGCTTCATTATGTGAATAAGATAT------AGA-T------------
TAGATGCATGGAGT-----AC-TC-
AGTTATTCTAGACTATCAATATATAAA--------------------
TAAG---------------AAA----
ACTCATATTGGCTAGTGGCTTCTTCCGGAATGAGAAAGTGAATTTCACTATCGATTCTAGGA
T-AAAATG---ATTTATT--------
GATCGTTTTAAAATAGGATTTCAATACATCAATTCGACCCCAA-TTTCTTTG-----
ATGAAATTGATACTC------------ATCAGAACCAAA-
TTCACTTGATAGATGTACCTACCAATTCAAGAAAGATTCCAAGATATTTCA-----------
--------TTAAAAAATGGGATGGAGAAGTTCGATTTGTCCACACAAATTCTTAGATC-
AAAAAA----------TTTCCGAAACTCTCTTCATCCTTC----TTACT------
AGCTTTATCATCCCAGATTTCTTCTTTATTC-------ATTTT------
CTGGTATAGATATTTCGTTTTT-----------------CTAGATTCTA-GAAA---T-
CGATTTTTT----GTT-----ATT-CAAAAGTGAGTTATATAACAAATC-
AAAAAAGAAA---
CTCATTTTTT--

CGA------
TTTCGGTTCTTACACAATCTTGACAATTTCAAAAAAACGGATCATACTATGATCATAGTATG
ATGGCGGTTGGACACGTATGCCCCCATCGTCTAGTGGTTCAGGACATCTCTCTTTCAAGGAG
GCAGCGGGGATTCGACTTCCCCTGGGGG-TAGGTTACTACA-
AAAGAAAGTGAATCATGCATTATGAATAA-------GCCGAAA----
TTGGAATTTATTCTTGCTGGGTCGATGCCCGAGCGGTTAATGGGGACGGACTGTAAATTCGT
TGGCAATATGTCTACGC
</sequence>
<sequence>
<taxon idref="Cupreoliva"/>
T---------------------------------
AAATTCCTGAATGCTTCATTATGTGAATAAGATAT------AGA-
T-----------------GCATGGAGT-ACATAC-TC-
AGTTATTCTAGACTATCAATATATAAA--------------------
TAAG---------------
AAAGTAGACTCATATTGGCTAGTGGCTTCTTCCGGAATGAGAAAGTGAGTTTCACTATCGAT
TCTAGGAT-AAAATG---ATTTATT--------
GATCGTTTTAAAATAGGATTTCAATACATCAATTCGACCCCAA-TTTCTTTT-----
ATGAAATTGATACTC------------ATCAGAACCAAA-
TTCACTTGATAGATGTACCTACCAATTCAAGAAAGATTCCAAGATATTTCA-----------
--------TTAAAAAATGGATGGAGAAGTTCGATTTGTCCACACAAATTCTTAGATC-
AAAAAA----------TTTCCGAAACTCTCTTCATCCTTC----TTACT------
AGCTTTATCATCCCAGATTTCTTCTTTATTC-------ATTTT------
CTGGTATAGATATTTCGTTTTT-----------------CTAGATTCTA-GAAA---T-
CTATTTTTT----GTT-----ATT-CAAAAGTGAGTTATATAACAAATC-
AAAAAAGAAA---
CTCATTTTTT--

CGA------
TTTCGGTTCTTACACAATCTTGACAATTTCAAAAAAACGGGTCATACTATGATCATAGTATG
ATGGCGGTTGGACACGTATGCCCCCATCGTCTAGTGGTTCAGGACATCTCTCTTTCAAGGAG
GCAGCGGGGATTCGACTTCCCCTGGGGG-TAGGTTACTACA-
AAAGAAAGTGAATCATGCATTATGAATAA-------GCCGAAA----
TTGGAATTTATTCTTCCTGGGTCGATGCCCGAGCGGTTAATGGGGACGGACTGTAAATTCGT
TGGCAATATGTCTACGC
</sequence>
<sequence>
<taxon idref="Eschkerese"/>
T---------------------------------
AAAGTCCTGAATGCTTCATTATGTGAATAAGATAT------AGA-T------------
TAGATGCATGGAGT-----AC-TC-
AGTTATTCTAGACTATCAATATATAAA--------------------
TAAG---------------AAA----
ACTCATATTGGCTAGTGGCTTCTTCCGGAATGAGAAAGTGAATTTCACTATCGATTCTAGGA
T-AAAATG---ATTTATT--------
GATCGTTTTAAAATAGGATTTCAATACATCAATTCGACCCCAA-TTTCTTTG-----
ATGAAATTGATACTC------------ATCAGAACCAAA-
TTCACTTGATAGATGTACCTACCAATTCAAGAAAGATTCCAAGATATTTCA-----------
--------TTAAAAAATGGATGGAGAAGTTCGATTTGTCCACACAAATTCTTAGATC-
AAAAAA----------TTTCCGAAACTCTCTTCATCCTTC----TTACT------
AGCTTTATCATCCCAGATTTCTTCTTTATTC-------ATTTT------
CTGGTATAGATATTTCGTTTTT-----------------CTAGATTCTA-GAAA---T-
CTATTTTTTT---TTT-----ATT-CAAAAGTGAGTTATATAACAAATC-
AAAAAAGAAA---

CTCATTTTTT--

CGA------
TTTCGGTTCTTACACAATCTTGACAATTTCAAAAAAACGGATCATACTATGATCATAGTATG
ATGGCGGTTGGACACGTATGCCCCCATCGTCTAGTGGTTCAGGACATCTCTCTTTCAAGGAG
GCAGCGGGGATTCGACTTCCCCTGGGGG-TAGGTTACTACA-
AAAGAAAGTGAATCATGCATTATGAATAA-------GCCGAAA----
TTGGAATTTATTCTTGCTGGGTCGATGCCCGAGCGGTTAATGGGGACGGACTGTAAATTCGT
TGGCAATATGTCTACGC
</sequence>
<sequence>
<taxon idref="Melananthu"/>
T---------------------------------
AAAGTCCTGAATGCTTCATTATGTGAATAAGATAT------AGA-T------------
TAGATGCATGGAGT-----AC-TC-
AGTTATTCTAGACTATCAATATATAAA--------------------
TAAG---------------AAA----
ACTCATATTGGCTAGTGGCTTCTTCCGGAATGAGAAAGTGAATTTCACTATCGATTCTAGGA
T-AAAATG---ATTTATT--------
GATCGTTTTAAAATAGGATTTCAATACATCAATTCGACCCCAA-TTTCTTGG-----
ATGAAATTGATACTC------------ATCAGAACCAAA-
TTCACTTGATAGATGTACCTACCAATTCAAGAAAGATTCCAAGATATTTCA-----------
--------TTAAAAAATGGGATGGAGAAGTTCGATTTGTCCACACAAATTCTTAGATC-
AAAAAA----------TTTCCGAAACTCTCTTCATCCTTC----TTACT------
AGCTTTATCATCCCAGATTTCTTCTTTATTC-------ATTTT------
CTGGTATAGATATTTCGTTTTT-----------------CTAGATTCTA-GAAA---T-
CGATTTTTT----GTT-----ATT-CAAAAGTGAGTTATATAACAAATC-
AAAAAAGAAA---
CTCATTTTTT--

CGA------
TTTCGGTTCTTACACAATCTTGACAATTTCAAAAAAACGGATCATACTATGATCATAGTATG
ATGGCGGTTGGACACGTATGCCCCCATCGTCTAGTGGTTCAGGACATCTCTCTTTCAAGGAG
GCAGCGGGGATTCGACTTCCCCTGGGGG-TAGGTTACTACA-
AAAGAAAGTGAATCATGCATTATGAATAA-------GCCGAAA----
TTGGAATTTATTCTTGCTGGGTCGATGCCCGAGCGGTTAATGGGGACGGACTGTAAATTCGT
TGGCAATATGTCTACGC
</sequence>
<sequence>
<taxon idref="Mischodage"/>
T---------------------------------
AAATTCCTGAATGCTTCATTATGTGAATAAGATAT------AGA-
T-----------------GCATGGAGT-ACATAC-TC-
AGTTATTCTAGACTATCAATATATAAA--------------------
TAAG---------------
AAAGTAGACTCATATTGGCTAGTGGCTTCTTCCGGAATGAGAAAGTGAGTTTCACTATCGAT
TCTAGGAT-AAAATG---ATTTATT--------
GATCGTTTTAAAATAGGATTTCAATACATCAATTCGACCCCAA-TTTCTTTT-----
ATGAAATTGATACTC------------ATCAGAACCAAA-
TTCACTTGATAGATGTACCTACCAATTCAAGAAAGATTCCAAGATATTTCA-----------
--------TTAAAAAATGGGATGGAGAAGTTCGATTTGTCCACACAAATTCTTAGATC-
AAAAAA----------TTTCCGAAACTCTCTTCATCCTTC----TTACT------

AGCTTTATCATCCCAGATTTCTTCTTTATTC-------ATTTT------
CTGGTATAGATATTTCGTTTTT-----------------CTAGATTCTA-GAAA---T-
CTATTTTTT----GTT-----ATT-CAAAAGTGAGTTAGATAACAAATC-
AAAAAAGAAA---
CTCATTGTTT--

CGA------
TTTCGGTTCTTACACAATCTTGACAATTTCAAAAAAACGGGTCATACTATGATCATAGTATG
ATGGCGGTTGGACACGTATGCCCCCATCGTCTAGTGGTTCAGGACATCTCTCTTTCAAGGAG
GCAGCGGGGATTCGACTTCCCCTGGGGG-TAGGTTACTACA-
AAAGAAAGTGAATCATGCATTATGAATAA-------GCCGAAA----
TTGGAATTTATTCTTCCTGGGTCGATGCCCGAGCGGTTAATGGGGACGGACTGTAAATTCGT
TGGCAATATGTCTACGC
</sequence>
<sequence>
<taxon idref="Nigricanse"/>
T---------------------------------
AAAGTCCTGAATGCTTCATTATGTGAATAAGATAT------AGA-T------------
TAGATGCATGGAGT-----AC-TC-
AGTTATTCTAGACTATCAATATATAAA--------------------
TAAG---------------AAA----
ACTCATATTGGCTAGTGGCTTCTTCCGGAATGAGAAAGTGAATTTCACTATCGATTCTAGGA
T-AAAATG---ATTTATT--------
GATCGTTTTAAAATAGGATTTCAATACATCAATTCGACCCCAA-TTTCTTGG-----
ATGAAATTGATACTC------------ATCAGAACCAAA-
TTCACTTGATAGATGTACCTACCAATTCAAGAAAGATTCCAAGATATTTCA-----------
--------TTAAAAAATGGATGGAGAAGTTCGATTTGTCCACACAAATTCTTAGATC-
AAAAAA----------TTTCCGAAACTCTCTTCATCCTTC----TTACT------
AGCTTTATCATCCCAGATTTCTTCTTTATTC-------ATTTT------
CTGGTATAGATATTTCGTTTTT-----------------CTAGATTCTA-GAAA---T-
CGATTTTTT----GTT-----ATT-CAAAAGTGAGTTATATAACAAATC-
AAAAAAGAAA---
CTCATTTTTT--

CGA------
TTTCGGTTCTTACACAATCTTGACAATTTCAAAAAAACGGATCATACTATGATCATAGTATG
ATGGCGGTTGGACACGTATGCCCCCATCGTCTAGTGGTTCAGGACATCTCTCTTTCAAGGAG
GCAGCGGGGATTCGACTTCCCCTGGGGG-TAGGTTACTACA-
AAAGAAAGTGAATCATGCATTATGAATAA-------GCCGAAA----
TTGGAATTTATTCTTGCTGGGTCGATGCCCGAGCGGTTAATGGGGACGGACTGTAAATTCGT
TGGCAATATGTCTACGC
</sequence>
<sequence>
<taxon idref="Fominiiiii"/>

T--

</sequence>
<sequence>
<taxon idref="Olivieri1i"/>
T---------------------------------
AAAGTCCTGAATGCTTCATTATGTGAATAAGATAT------AGA-T------------
TAGATGCATGGAGT-----AC-TC-
AGTTATTCTAGACTATCAATATATAAA--------------------
TAAG---------------AAA----
ACTCATATTGGCTAGTGGCTTCTTCCGGAATGAGAAAGTGAATTTCACTATCGATTCTAGGA
T-AAAATG---ATTTATT--------
GATCGTTTTAAAATAGGATTTCAATACATCAATTCGACCCCAA-TTTCTTTG-----
ATGAAATTGATACTC------------ATCAGAACCAAA-
TTCACTTGATAGATGTACCTACCAATTCAAGAAAGATTCCAAGATATTTCA-----------
--------TTCAAAAATGGATGGAGAAGTTCGATTTGTCCACACAAATTCTTAGATCC-
AAAAA----------TTTCCGAAACTCTCTTCATCCTTC----TTACT------
AGCTTTATCATCCCAGATTTCTTCTTTATTC-------ATTTT------
CTGGTATAGATATTTCGTTTTT-----------------CTAGATTCTA-GAAA---T-
CGATTTTTT----GTT-----ATT-CAAAAGTGAGTTATATAACAAATC-
AAAAAAGAAA---
CTCATTTTTT--

CGA------
TTTCGGTTCTTACACAATCTTGACAATTTCAAAAAAACGGATCATACTATGATCATAGTATG
ATGGCGGTTGGACACGTATGCCCCCATCGTCTAGTGGTTCAGGACATCTCTCTTTCAAGGAG
GCAGCGGGGATTCGACTTCCCCTGGGGG-TAGGTTACTACA-
AAAGAAAGTGAATCATGCATTATGAATAA-------GCCGAAA----
TTGGAATTTATTCTTGCTGGGTCGATGCCCGAGCGGTTAATGGGGACGGACTGTAAATTCGT
TGGCAATATGTCTACGC
</sequence>
<sequence>
<taxon idref="Ophiocladu"/>
T---------------------------------
AAAGTCCTGAATGCTTCATTATGTGAATAAGATAT------AGA-T------------
TAGATGCATGGAGT-----AC-TC-
AGTTATTCTAGACTATCAATATATAAA--------------------
TAAG---------------AAA----
ACTCATATTGGCTAGTGGCTTCTTCCGGAATGAGAAAGTGAATTTCACTATCGATTCTAGGA
T-AAAATG---ATTTATT--------
GATCGTTTTAAAATAGGATTTCAATACATCAATTCGACCCCAA-TTTCTTGG-----

ATGAAATTGATACTC------------ATCAGAACCAAA-
TTCACTTGATAGATGTACCTACCAATTCAAGAAAGATTCCAAGATATTTCA-----------
--------TTAAAAAATGGATGGAGAAGTTCGATTTGTCCACACAAATTCTTAGATC-
AAAAAA----------TTTCCGAAACTCTCTTCATCCTTC----TTACT------
AGCTTTATCATCCCAGATTTCTTCTTTATTC-------ATTTT------
CTGGTATAGATATTTCGTTTTT-----------------CTAGATTCTA-GAAA---T-
CGATTTTTT----GTT-----ATT-CAAAAGTGAGTTATATAACAAATC-
AAAAAAGAAA---
CTCATTTTTT--

CGA------
TTTCGGTTCTTACACAATCTTGACAATTTCAAAAAAACGGATCATACTATGATCATAGTATG
ATGGCGGTTGGACACGTATGCCCCCATCGTCTAGTGGTTCAGGACATCTCTCTTTCAAGGAG
GCAGCGGGGATTCGACTTCCCCTGGGGG-TAGGTTACTACA-
AAAGAAAGTGAATCATGCATTATGAATAA-------GCCGAAA----
TTGGAATTTATTCTTGCTGGGTCGATGCCCGAGCGGTTAATGGGGACGGACTGTAAATTCGT
TGGCAATATGTCTACGC
</sequence>
<sequence>
<taxon idref="wendORO10k"/>
T--------------------
GGGCCTTTTTCGTAAATTCCTGAATGCTTCATTATGTGAATAAGATAT------AGA-
T-----------------GCATGGAGT-ACATAC-TC-
AGTTATTCTAGACTATCAATATATAAA--------------------
TAAG---------------
AAAGTAGACTCATATTGGCTAGTGGCTTCTTCCGGAATGAGAAAGTGAGTTTCACTATCGAT
TCTAGGAT-AAAATG---ATTTATT--------
GATCGTTTTAAAATAGGATTTCAATACATCAATTCGACCCCAA-TTTCTTTT-----
ATGAAATTGATACTC------------ATCAGAACCAAA-
TTCACTTGATAGATGTACCTACCAATTCAAGAAAGATTCCAAGATATTTCA-----------
--------TTAAAAAATGGATGGAGAAGTTCGATTTGTCCACACAAATTCTTAGATC-
AAAAAA----------TTTCCGAAACTCTCTTCATCCTTC----TTACT------
AGCTTTATCATCCCAGATTTCTTCTTTATTC-------ATTTT------
CTGGTATAGATATTTCGTTTTT-----------------CTAGATTCTA-GAAA---T-
CTATTTTTT----GTT-----ATT-CAAAAGTGAGTTATATAACAAATC-
AAAAAAGAAA---
CTCATTTTTT--

CGA------
TTTCGGTTCTTACACAATCTTGACAATTTCAAAAAAACGGGTCATACTATGATCATAGTATG
ATGGCGGTTGGACACGTATGCCCCCATCGTCTAGTGGTTCAGGACATCTCTCTTTCAAGGAG
GCAGCGGGGATTCGACTTCCCCTGGGGG-TAGGTTACTACA-
AAAGAAAGTGAATCATGCATTATGAATAA-------GCCGAAA----
TTGGAATTTATTCTTCCTGGGTCGATGCCCGAGCGGTTAATGGGGACGGACTGTAAATTCGT
TGGCTATATGTCTACGC
</sequence>
<sequence>
<taxon idref="Serotinumo"/>
T---------------------------------
AAAGTCCTGAATGCTTCATTATGTGAATAAGATAT------AGA-T------------
TAGATGCATGGAGT-----AC-TC-
AGTTATTCTAGACTATCAATATATAAA--------------------

TAAG---------------AAA----
ACTCATATTGGCTAGTGGCTTCTTCCGGAATGAGAAAGTGAATTTCACTATCGATTCTAGGA
T-AAAATG---ATTTATT--------
GATCGTTTTAAAATAGGATTTCAATACATCAATTCGACCCCAA-TTTCTTGG-----
ATGAAATTGATACTC------------ATCAGAACCAAA-
TTCACTTGATAGATGTACCTACCAATTCAAGAAAGATTCCAAGATATTTCA-----------
--------TTAAAAAAATGGATGGAGAAGTTCGATTTGTCCACACAAATTCTTAGATC-
AAAAAA----------TTTCCGAAACTCTCTTCATCCTTC----TTACT------
AGCTTTATCATCCCAGATTTCTTCTTTATTC-------ATTTT------
CTGGTATAGATATTTCGTTTTT-----------------CTAGATTCTA-GAAA---T-
CGATTTTTT----GTT-----ATT-CAAAAGTGAGTTATATAACAAATC-
AAAAAAGAAA---
CTCATTTTT---

CGA------
TTTCGGTTCTTACACAATCTTGACAATTTCAAAAAAACGGATCATACTATGATCATAGTATG
ATGGCGGTTGGACACGTATGCCCCCATCGTCTAGTGGTTCAGGACATCTCTCTTTCAAGGAG
GCAGCGGGGATTCGACTTCCCCTGGGGG-TAGGTTACTACA-
AAAGAAAGTGAATCATGCATTATGAATAA-------GCCGAAA----
TTGGAATTTATTCTTGCTGGGTCGATGCCCGAGCGGTTAATGGGGACGGACTGTAAATTCGT
TGGCAATATGTCTACGC
</sequence>
<sequence>
<taxon idref="Curviflor1"/>
T-------------------------------------
TCCTGAATGCTTCATTATGTGAATAAGATAT------AGA-T-----------------
GCATGGAGT-ACATAC-TC-
AGTTATTCTAGACTATCAATATATAAA--------------------
TAAG---------------
AAAGTAGACTCATATTGGCTAGTGGCTTCTTCCGGAATGAGAAAGTGAATTTCACTATCGAT
TCTAGGAT-AAAATG---ATTTATT--------
GATCGTTTTAAAATAGGATTTCAATACATCAATTCGACCCCAA-TTTCTTTT-----
ATGAAATTGATACTC------------ATCAGAACCAAA-
TTCACTTGATAGATGTACCTACCAATTCAAGAAAGATTCCAAGATATTTCA-----------
--------TTAAAAAAATGGATGGAGAAGTTCGATTTGTCCACACAAATTCTTAGATC-
AAAAAA----------TTTCCGAAACTCTCTTCATCCTTC----TTACT------
AGCTTTATCATCCCAGATTTCTTCTTTATTC-------ATTTT------
CTGGTATAGATATTTCGTTTTT-----------------CTAGATTCTA-GAAA---T-
CTATTTTTT----GTT-----ATT-CAAAAGTGAGTTATATAACAAATC-
AAAAAAGAAA---
CTCATTTTT---

CGA------
TTTCGGTTCTTACACAATCTTGACAATTTCAAAAAAACGGGTCATACTATGATCATAGTATG
ATGGCGGTTGGACACGTATGCCCCCATCGTCTAGTGGTTCAGGACATCTCTCTTTCAAGGAG
GCAGCGGGGATTCGACTTCCCCTGGGGG-TAGGTTACTACA-
AAAGAAAGTGAATCATGCATTATGAATAA-------GCCGAAA----
TTGGAATTTATTCTTCCTGGGTCGATGCCCGAGCGGTTAATGGGGACGGACTGTAAATTCGT
TGGCAATATGTCTACGC
</sequence>
<sequence>
<taxon idref="Densiflor1"/>

TAAAGGGCCTTTGAGTTAAAAGGGCCTTTTTCGTAAAGTCCTGAATGCTTCATTATG
TGAATAAGATAT------AGA-T------------TAGATGCATGGAGT-----AC-TC-
AGTTATTCTAGACTATCAATATATAAA--------------------
TAAG---------------AAA----
ACTCATATTGGCTAGTGGCTTCTTCCGGAATGAGAAAGTGAATTTCACTATCGATTCTAGGA
T-AAAATG---ATTTATT--------
GATCGTTTTAAAATAGGATTTCAATACATCAATTCGACCCCAA-TTTCTTTG-----
ATGAAATTGATACTC------------ATCAGAACCAAA-
TTCACTTGATAGATGTACCTACCAATTCAAGAAAGATTCCAAGATATTTCA-----------
--------TTAAAAAATGGGATGGAGAAGTTCGATTTGTCCACACAAATTCTTAGATC-
AAAAAA----------TTTCCGAAACTCTCTTCATCCTTC----TTACT------
AGCTTTATCATCCCAGATTTCTTCTTTATTC-------ATTTT------
CTGGTATAGATATTTCGTTTTT-----------------CTAGATTCTA-GAAA---T-
CGATTTTTT----GTT-----ATT-CAAAAGTGAGTTATATAACAAATC-
AAAAAAGAAA---
CTCATTTTTT--

CGA------
TTTCGGTTCTTACACAATCTTGACAATTTCAAAAAAACGGATCATACTATGATCATAGTATG
ATGGCGGTTGGACACGTATGCCCCCATCGTCTAGTGGTTCAGGACATCTCTCTTTCAAGGAG
GCAGCGGGGATTCGACTTCCCCTGGGGG-TAGGTTACTACA-
AAAGAAAGTGAATCATGCATTATGAATAA-------GCCGAAA----
TTGGAATTTATTCTTGCTGGGTCGATGCCCGAGCGGTTAATGGGGACGGACTGTAAATTCGT
TGGCTATATGTCTACGC
</sequence>
<sequence>
<taxon idref="Hystrixxxx"/>

T--

</sequence>
<sequence>
<taxon idref="Karelinii1"/>

T-------------------------------------
TCCTGAATGCTTCATTATGTGAATAAGATAT------AGA-T-----------------
GCATGGAGT-ACATAC-TC-
AGTTATTCTAGACTATCAATATATAAA--------------------
TAAG---------------
AAAGTAGACTCATATTGGCTAGTGGCTTCTTCCGGAATGAGAAAGTGAATTTCACTATCGAT
TCTAGGAT-AAAATG---ATTTATT--------
GATCGTTTTAAAATAGGATTTCAATACATCAATTCGACCCCAA-TTTCTTTT-----
ATGAAATTGATACTC------------ATCAGAACCAAA-
TTCACTTGATAGATGTACCTACCAATTCAAGAAAGATTCCAAGATATTTCA-----------
--------TTAAAAAATGGATGGAGAAGTTCGATTTGTCCACACAAATTCTTAGATC-
AAAAAA----------TTTCCGAAACTCTCTTCATCCTTC----TTACT------
AGCTTTATCATCCCAGATTTCTTCTTTATTC-------ATTTT------
CTGGTATAGATATTTCGTTTTT-----------------CTAGATTCTA-GAAA---T-
CTATTTTTT----GTT-----ATT-CAAAAGTGAGTTATATAACAAATC-
AAAAAAGAAA---
CTCATTTTTT--

CGA------
TTTCGGTTCTTACACAATCTTGACAATTTCAAAAAAACGGGTCATACTATGATCATAGTATG
ATGGCGGTTGGACACGTATGCCCCCATCGTCTAGTGGTTCAGGACATCTCTCTTTCAAGGAG
GCAGCGGGGATTCGACTTCCCCTGGGGG-TAGGTTACTACA-
AAAGAAAGTGAATCATGCATTATGAATAA-------GCCGAAA----
TTGGAATTTATTCTTCCTGGGTCGATGCCCGAGCGGTTAATGGGGACGGACTGTAAATTCGT
TGGCTATATGTCTACGC
</sequence>
<sequence>
<taxon idref="Rudbaricu1"/>

TAAAGGGCCTTTGAGTTAAAAGGGCCTTTTTCGTAAATTCCTGAATGCTTCATTATG
TGAATAAGATAT------AGA-T-----------------GCATGGAGT-ACATAC-TC-
AGTTATTCTAGACTATCAATATATAAA--------------------
TAAG---------------
AAAGTAGACTCATATTGGCTAGTGGCTTCTTCCGGAATGAGAAAGTGAATTTCACTATCGAT
TCTAGGAT-AAAATG---ATTTATT--------
GATCGTTTTAAAATAGGATTTCAATACATCAATTCGACCCCAA-TTTCTTTT-----
ATGAAATTGATACTC------------ATCAGAACCAAA-
TTCACTTGATAGATGTACCTACCAATTCAAGAAAGATTCCAAGATATTTCA-----------
--------TTAAAAAATGGATGGAGAAGTTCGATTTGTCCACACAAATTCTTAGATC-
AAAAAA----------TTTCCGAAACTCTCTTCATCCTTC----TTACT------
AGCTTTATCATCCCAGATTTCTTCTTTATTC-------ATTTT------
CTGGTATAGATATTTCGTTTTT-----------------CTAGATTCTA-GAAA---T-
CTATTTTTT----GTT-----ATT-CAAAAGTGAGTTATATAACAAATC-
AAAAAAGAAA---
CTCATTTTTT--

CGA------
TTTCGGTTCTTACACAATCTTGACAATTTCAAAAAAACGGGTCATACTATGATCATAGTATG
ATGGCGGTTGGACACGTATGCCCCCATCGTCTAGTGGTTCAGGACATCTCTCTTTCAAGGAG
GCAGCGGGGATTCGACTTCCCCTGGGGG-TAGGTTACTACA-
AAAGAAAGTGAATCATGCATTATGAATAA-------GCCGAAA----
TTGGAATTTATTCTTCCTGGGTCGATGCCCGAGCGGTTAATGGGGACGGACTGTAAATTCGT

TGGCTATATGTCTACGC
</sequence>
<sequence>
<taxon idref="Mirtajadin"/>

T--

</sequence>
<sequence>
<taxon idref="Sacberllum"/>

T--

</sequence>
<sequence>
<taxon idref="Shirazianu"/>

T--

</sequence>
<sequence>
<taxon idref="Hormozgane"/>
T---------------------------------
AAAGTCCTGAATGCTTCATTATGTGAATAAGATAT------AGA-T------------
TAGATGCATGGAGT-----AC-TC-
AGTTATTCTAGACTATCAATATATAAA--------------------
TAAG---------------AAA----
ACTCATATTGGCTAGTGGCTTCTTCCGGAATGAGAAAGTGAATTTCACTATCGATTCTAGGA
T-AAAATG---ATTTATT--------
GATCGTTTTAAAATAGGATTTCAATACATCAATTCGACCCCAA-TTTCTTTG-----
ATGAAATTGATACTC------------ATCAGAACCAAA-
TTCACTTGATAGATGTACCTACCAATTCAAGAAAGATTCCAAGATATTTCA-----------
--------TTAAAAAATGGGATGGAGAAGTTCGATTTGTCCACACAAATTCTTAGATC-
AAAAAA----------TTTCCGAAACTCTCTTCATCCTTC----TTACT------
AGCTTTATCATCCCAGATTTCTTCTTTATTC-------ATTTT------
CTGGTATAGATATTTCGTTTTT-----------------CTAGATTCTA-GAAA---T-
CTATTTTTTT---TTT-----ATT-CAAAAGTGAGTTATATAACAAATC-
AAAAAAGAAA---
CTCATTTTTT--

CGA------
TTTCGGTTCTTACACAATCTTGACAATTTCAAAAAAACGGATCATACTATGATCATAGTATG
ATGGCGGTTGGACACGTATGCCCCCATCGTCTAGTGGTTCAGGACATCTCTCTTTCAAGGAG
GCAGCGGGGATTCGACTTCCCCTGGGGG-TAGGTTACTACA-
AAAGAAAGTGAATCATGCATTATGAATAA-------GCCGAAA----
TTGGAATTTATTCTTGCTGGGTCGATGCCCGAGCGGTTAATGGGGACGGACTGTAAATTCGT
TGGCAATATGTCTACGC
</sequence>
<sequence>
<taxon idref="Glabratum1"/>

T--

</sequence>
<sequence>
<taxon idref="Moradiiiii"/>
T---------------------------------
AAAGTCCTGAATGCTTCATTATGTGAATAAGATAT------AGA-T------------
TAGATGCATGGAGT-----AC-TC-
AGTTATTCTAGACTATCAATATATAAA--------------------
TAAG---------------AAA----
ACTCATATTGGCTAGTGGCTTCTTCCGGAATGAGAAAGTGAATTTCACTATCGATTCTAGGA
T-AAAATG---ATTTATT--------
GATCGTTTTAAAATAGGATTTCAATACATCAATTCGACCCCAA-TTTCTTTG-----
ATGAAATTGATACTC------------ATCAGAACCAAA-
TTCACTTGATAGATGTACCTACCAATTCAAGAAAGATTCCAAGATATTTCA-----------
--------TTAAAAAATGGGATGGAGAAGTTCGATTTGTCCACACAAATTCTTAGATC-
AAAAAA----------TTTCCGAAACTCTCTTCATCCTTC----TTACT------
AGCTTTATCATCCCAGATTTCTTCTTTATTC-------ATTTT------
CTGGTATAGATATTTCGTTTTT-----------------CTAGATTCTA-GAAA---T-
CTATTTTTTT---TTT-----ATT-CAAAAGTGAGTTATATAACAAATC-
AAAAAAGAAA---
CTCATTTTTT--

CGA------
TTTCGGTTCTTACACAATCTTGACAATTTCAAAAAAACGGATCATACTATGATCATAGTATG
ATGGCGGTTGGACACGTATGCCCCCATCGTCTAGTGGTTCAGGACATCTCTCTTTCAAGGAG
GCAGCGGGGATTCGACTTCCCCTGGGGG-TAGGTTACTACA-
AAAGAAAGTGAATCATGCATTATGAATAA-------GCCGAAA----
TTGGAATTTATTCTTGCTGGGTCGATGCCCGAGCGGTTAATGGGGACGGACTGTAAATTCGT
TGGCAATATGTCTACGC
</sequence>
<sequence>
<taxon idref="Tomentellu"/>

                T-------------------------------------
TCCTGAATGCTTCATTATGTGAATAAGATAT------AGA-T-----------------
GCATGGAGT-ACATAC-TC-
AGTTATTCTAGACTATCAATATATAAA--------------------
TAAG---------------
AAAGTAGACTCATATTGGCTAGTGGCTTCTTCCGGAATGAGAAAGTGAGTTTCACTATCGAT
TCTAGGAT-AAAATG---ATTTATT--------
GATCGTTTTAAAATAGGATTTCAATACATCAATTCGACCCCAA-TTTCTTTT-----
ATGAAATTGATACTC------------ATCAGAACCAAA-
TTCACTTGATAGATGTACCTACCAATTCAAGAAAGATTCCAAGATATTTCA-----------
--------TTAAAAAATGGGATGGAGAAGTTCGATTTGTCCACACAAATTCTTAGATC-
AAAAAA----------TTTCCGAAACTCTCTTCATCCTTC----TTACT------
AGCTTTATCATCCCAGATTTCTTCTTTATTC-------ATTTT------
CTGGTATAGATATTTCGTTTTT-----------------CTAGATTCTA-GAAA---T-
CTATTTTTT----GTT-----ATT-CAAAAGTGAGTTATATAACAAATC-
AAAAAAGAAA---
CTCATTTTTT--

CGA------
TTTCGGTTCTTACACAATCTTGACAATTTCAAAAAAACGGGTCATACTATGATCATAGTATG
ATGGCGGTTGGACACGTATGCCCCCATCGTCTAGTGGTTCAGGACATCTCTCTTTCAAGGAG
GCAGCGGGGATTCGACTTCCCCTGGGGG-TAGGTTACTACA-
AAAGAAAGTGAATCATGCATTATGAATAA-------GCCGAAA----
TTGGAATTTATTCTTCCTGGGTCGATGCCCGAGCGGTTAATGGGGACGGACTGTAAATTCGT
TGGCAATATGTCTACGC
            </sequence>
            <sequence>
                <taxon idref="Hohenacer1"/>
                T-------------------------------------
TCCTGAATGCTTCATTATGTGAATAGGATAT------AGA-T------------
TAGATGCATGGAGT-----AC-TC-
AGTTATTCTAGACTATCAATATATAAA--------------------
TAAG---------------AAA----
ACTCATATTGGCTAGTGGCTTCTTCCGGAATGAGAAAGTGAATTTCACTATCGATTCTAGGA
T-AAAATG---ATTTATT--------
GATCGTTTTAAAATAGGATTTCAATACATCAATTCGACCCCAA-TTTCTTTG-----
ATGAAATTGATACTC------------ATCAGAACCAAA-
TTCACTTGATAGATGTACCTACCAATTCAAGAAAGATTCCAAGATATTTCA-----------
--------TTAAAAAATGGGATGGAGAAGTTCGATTTGTCCACACAAATTCTTAGATCC-
AAAAA----------TTTCCGAAACTCTCTTCATCCTTC----TTACT------
AGCTTTATCATCCCAGATTTCTTCTTTATTC-------ATTTT------
CTGGTATAGATATTTCGTTTTT-----------------CTAGATTCTA-GAAA---T-
CGATTTTTT----GTT-----ATT-CAAAAGTGAGTTATATAACAAATC-
AAAAAAGAAA---
CTCATTTTTT--

CGA------
TTTCGGTTCTTACACAATCTTGACAATTTCAAAAAAACGGATCATACTATGATCATAGTATG
ATGGCGGTTGGACACGTATGCCCCCATCGTCTAGTGGTTCAGGACATCTCTCTTTCAAGGAG
GCAGCGGGGATTCGACTTCCCCTGGGGG-TAGGTTACTACA-
AAAGAAAGTGAATCATGCATTATGAATAA-------GCCGAAA----
TTGGAATTTATTCTTGCTGGGTCGATGCCCGAGCGGTTAATGGGGACGGACTGTAAATTCGT

TGGCAATATGTCTACGC
</sequence>
<sequence>
<taxon idref="Acanthob40"/>
T-------------------------------------
TCCTGAATGCTTCATTATGTGAATAAGATAT------AGA-T-----------------
GCATGGAGT-ACATAC-TC-
AGTTATTCTAGACTATCAATATATAAA--------------------
TAAG---------------
AAAGTAGACTCATATTGGCTAGTGGCTTCTTCCGGAATGAGAAAGTGAGTTTCACTATCGAT
TCTAGGAT-AAAATG---ATTTATT--------
GATCGTTTTAAAATAGGATTTCAATACATCAATTCGACCCCAA-TTTCTTTT-----
ATGAAATTGATACTC------------ATCAGAACCAAA-
TTCACTTGATAGATGTACCTACCAATTCAAGAAAGATTCCAAGATATTTCA-----------
--------TTAAAAAATGGGATGGAGAAGTTCGATTTGTCCACACAAATTCTTAGATC-
AAAAAA----------TTTCCGAAACTCTCTTCATCCTTC----TTACT------
AGCTTTATCATCCCAGATTTCTTCTTTATTC-------ATTTT------
CTGGTATAGATATTTCGTTTTT-----------------CTAGATTCTA-GAAA---T-
CTATTTTTT----GTT-----ATT-CAAAAGTGAGTTAGATAACAAATC-
AAAAAAGAAA---
CTCATTGTTT--

CGA------
TTTCGGTTCTTACACAATCTTGACAATTTCAAAAAAACGGGTCATACTATGATCATAGTATG
ATGGCGGTTGGACACGTATGCCCCCATCGTCTAGTGGCTCAGGACATCTCTCTTTCAAGGAG
GC--

</sequence>
<sequence>
<taxon idref="ahangare92"/>
T-------------------------------------
TCCTGAATGCTTCATTATGTGAATAAGATAT------AGA-T-----------------
GCATGGAGT-ACATAC-TC-
AGTTATTCTAGACTATCAATATATAAA--------------------
TAAG---------------
AAAGTAGACTCATATTGGCTAGTGGCTTCTTCCGGAATGAGAAAGTGAGTTTCACTATCGAT
TCTAGGAT-AAAATG---ATTTATT--------
GATCGTTTTAAAATAGGATTTCAATACATCAATTCGACCCCAA-TTTCTTTT-----
ATGAAATTGATACTC------------ATCAGAACCAAA-
TTCACTTGATAGATGTACCTACCAATTCAAGAAAGATTCCAAGATATTTCA-----------
--------TTAAAAAATGGGATGGAGAAGTTCGATTTGTCCACACAAATTCTTAGATC-
AAAAAA----------TTTCCGAAACTCTCTTCATCCTTC----TTACT------
AGCTTTATCATCCCAGATTTCTTCTTTATTC-------ATTTT------
CTGGTATAGATATTTCGTTTTT-----------------CTAGATTCTA-GAAA---T-
CTATTTTTT----GTT-----ATT-CAAAAGTGAGTTAGATAACAAATC-
AAAAAAGAAA---
CTCATTGTTT--

CGA------
TTTCGGTTCTTACACAATCTTGACAATTTCAAAAAAACGGGTCATACTATGATCATAGTATG
ATGGCGGTTGGACACGTATGCCCCCATCGTCTAGTGGTTCAGGACATCTCTCTTTCAAGGAG

GC--

</sequence>
<sequence>
<taxon idref="AuganumW86"/>
T-------------------------------------
TCCTGAATGCTTCATTATGTGAATAAGATAT------AGA-T-----------------
GCATGGAGT-ACATAC-TC-
AGTTATTCTAGACTATCAATATATAAA--------------------
TAAG---------------
AAAGTAGACTCATATTGGCTAGTGGCTTCTTCCGGAATGAGAAAGTGAGTTTCACTATCGAT
TCTAGGAT-AAAATG---ATTTATT--------
GATCGTTTTAAAATAGGATTTCAATACATCAATTCGACCCCAA-TTTCTTTT-----
ATGAAATTGATACTC------------ATCAGAACCAAA-
TTCACTTGATAGATGTACCTACCAATTCAAGAAAGATTCCAAGATATTTCA-----------
--------TTAAAAAATGGGATGGAGAAGTTCGATTTGTCCACACAAATTCTTAGATC-
AAAAAA----------TTTCCGAAACTCTCTTCATCCTTC----TTACT------
AGCTTTATCATCCCAGATTTCTTCTTTATTC-------ATTTT------
CTGGTATAGATATTTCGTTTTT-----------------CTAGATTCTA-GAAA---T-
CTATTTTTT----GTT-----ATT-CAAAAGTGAGTTAGATAACAAATC-
AAAAAAGAAA---
CTCATTTTT---

CGA------
TTTCGGTTCTTACACAATCTTGACAATTTCAAAAAAACGGGTCATACTATGATCATAGTATG
ATGGCGGTTGGACACGTATGCCCCCATCGTCTAGTGGCTCAGGACATCTCTCTTTCAAGGAG
GCAGCGGGGATTCGACTTCCCCTGGGGG----------------------------------
----

</sequence>
<sequence>
<taxon idref="Cabulicu99"/>
T-------------------------------------
TCCTGAATGCTTCATTATGTGAATAAGATAT------AGA-T-----------------
GCATGGAGT-ACATAC-TC-
AGTTATTCTAGACTATCAATATATAAA--------------------
TAAG---------------
AAAGTAGACTCATATTGGCTAGTGGCTTCTTCCGGAATGAGAAAGTGAGTTTCACTATCGAT
TCTAGGAT-AAAATG---ATTTATT--------
GATCGTTTTAAAATAGGATTTCAATACATCAATTCGACCCCAA-TTTCTTTT-----
ATGAAATTGATACTC------------ATCAGAACCAAA-
TTCACTTGATAGATGTACCTACCAATTCAAGAAAGATTCCAAGATATTTCA-----------
--------TTAAAAAATGGGATGGAGAAGTTCGATTTGTCCACACAAATTCTTAGATC-
AAAAAA----------TTTCCGAAACTCTCTTCATCCTTC----TTACT------
AGCTTTATCATCCCAGATTTCTTCTTTATTC-------ATTTT------
CTGGTATAGATATTTCGTTTTT-----------------CTAGATTCTA-GAAA---T-
CTATTTTTT----GTT-----ATT-CAAAAGTGAGTTATATAACAAATC-
AAAAAAGAAA---
CTCATTTTT---

CGA------

TTTCGGTTCTTACACAATCTTGACAATTTCAAAAAAACGGGTCATACTATGATCATAGTATG
ATGGCGGTTGGACACGTATGCCCCCATCGTCTAGTGGTTCAGGACATCTCTCTTTCAAGGAG
GCAGCGGGGATTCGACTTCCCCTGGGGG-TAGGTTACTACA-
AAAGAAAGTGAATCATGCATTATGAATAA-------GCCGAAA----
TTGGAATTTATTCTTCCTGGGTCGATGCCCGAGCGGTTAATGGGGACGGACTGTAAATTCGT
TGGCAATATGTCTACGC
</sequence>
<sequence>
<taxon idref="Carinatu25"/>
T-------------------------------------
TCCTGAATGCTTCATTATGTGAATAAGATAT------AGA-T-----------------
GCATGGAGT-ACATAC-TC-
AGTTATTCTAGACTATCAATATATAAA--------------------
TAAG---------------
AAAGTAGACTCATATTGGCTAGTGGCTTCTTCCGGAATGAGAAAGTGAGTTTCACTATCGAT
TCTAGGAT-AAAATG---ATTTATT--------
GATCGTTTTAAAATAGGATTTCAATACATCAATTCGACCCCAA-TTTCTTTT-----
ATGAAATTGATACTC------------ATCAGAACCAAA-
TTCACTTGATAGATGTACCTACCAATTCAAGAAAGATTCCAAGATATTTCA-----------
--------TTAAAAAATGGGATGGAGAAGTTCGATTTGTCCACACAAATTCTTAGATC-
AAAAAA----------TTTCCGAAACTCTCTTCATCCTTC----TTACT------
AGCTTTATCATCCCAGATTTCTTCTTTATTC-------ATTTT------
CTGGTATAGATATTTCGTTTTT-----------------CTAGATTCTA-GAAA---T-
CTATTTTTT----GTT-----ATT-CAAAAGTGAGTTAGATAACAAATC-
AAAAAAGAAA---
CTCATTGTTT--

CGA------
TTTCGGTTCTTACACAATCTTGACAATTTCAAAAAAACGGGTCATACTATGATCATAGTATG
ATGGCGGTTGGACACGTATGCCCCCATCGTCTAGTGGTTCAGGACATCTCTCTTTCAAGGAG
GC--

</sequence>
<sequence>
<taxon idref="Cephalot39"/>

T--

</sequence>
<sequence>
<taxon idref="erinaceu70"/>
T-------------------------------------
TCCTGAATGCTTCATTATGTGAATGAAATAT------AGA-T-----------------
GCATGGAGT-ACATAC-TC-
AGTTATTCTAGACTATCAATATATAAA--------------------
TAAG---------------
AAAGTAGACTCATATTGGCTAGTGGCTTCTTCCGGAATGAGAAAGTGAGTTTCACTATCGAT
TCTAGGAT-AAAATG---ATTTATT--------
GATCGTTTTAAAATAGGATTTCAATACATCAATTCGACCCCAA-TTTCTTTT-----
ATGAAATTGATACTC------------ATCAGAACCAAA-
TTCACTTGATAGATGTACCTACCAATTCAAGAAAGATTCCAAGATATTTCA-----------
--------TTAAAAAATGGATGGAGAAGTTCGATTTGTCCACACAAATTCTTAGATC-
AAAAAA----------TTTCCGAAACTCTCTTCATCCTTC----TTACT------
AGCTTTATCATCCCAGATTTCTTCTTTATTC-------ATTTT------
CTGGTATAGATATTTCGTTTTT-----------------CTAGATTCTA-GAAA---T-
CTATTTTTT----GTT-----ATT-CAAAAGTGAGTTAGATAACAAATC-
AAAAAAGAAA---
CTCATTTTTT--

CGA------
TTTCGGTTCTTACACAATCTTGACAATTTCAAAAAAACGGGTCATACTATGATCATAGTATG
ATGGCGGTTGGACACGTATGCCCCCATCGTCTAGTGGCTCAGGACATCTCTCTTTCAAGGAG
GCAGCGGGGATTCGACTTCCCCTGGGGG-TAGGTTACTACA-
AAAGAAAGTGAATCATGCATTATGAATAA-------GCCGAAA----
TTGGAATTTATTCTTCCTGGGTCGATGCCCGAGCGGTTAATGGGGACGGACTGTAAATTCGT
TGGCAATATGTCTACGC
</sequence>
<sequence>
<taxon idref="fascicul45"/>

T--

</sequence>
<sequence>
<taxon idref="Festucas88"/>
T-------------------------------------
TCCTGAATGCTTCATTATGTGAATAAGATAT------AGA-T------------
TAGATGCATGGAGT-----AC-TC-
AGTTATTCTAGACTATCAATATATAAA--------------------
TAAG---------------AAA----
ACTCATATTGGCTAGTGGCTTCTTCCGGAATGAGAAAGTGAATTTCACTATCGATTCTAGGA
T-AAAATG---ATTTATT--------
GATCGTTTTAAAATAGGATTTCAATACATCAATTCGACCCCAA-TTTCTTGG-----
ATGAAATTGATACTC------------ATCAGAACCAAA-
TTCACTTGATAGATGTACCTACCAATTCAAGAAAGATTCCAAGATATTTCA-----------
--------TTAAAAAATGGGATGGAGAAGTTCGATTTGTCCACACAAATTCTTAGATC-
AAAAAA----------TTTCCGAAACTCTCTTCATCCTTC----TTACT------
AGCTTTATCATCCCAGATTTCTTCTTTATTC-------ATTTT------
CTGGTATAGATATTTCGTTTTT-----------------CTAGATTCTA-GAAA---T-
CGATTTTTT----GTT-----ATT-CAAAAGTGAGTTATATAACAAATC-
AAAAAAGAAA---
CTCATTTTTT--

CGA------
TTTCGGTTCTTACACAATCTTGACAATTTCAAAAAAACGGATCATACTATGATCATAGTATG
ATGGCGGTTGGACACGTATGCCCCCATCGTCTAGTGGTTCAGGACATCTCTCTTTCAAGGAG
GCAGCGGGGATTCGACTTCCCCTGGGGG-TAGGTTACTACA-
AAAGAAAGTGAATCATGCATTATGAATAA-------GCCGAAA----
TTGGAATTTATTCTTGCTGGGTCGATGCCCGAGCGATTAATGGGGACGGACTGTAAATTCGT
TGGCAATATGTCTACGC
</sequence>
<sequence>
<taxon idref="Chitrali79"/>

T--

</sequence>
<sequence>
<taxon idref="gilliiW141"/>

T--

</sequence>
<sequence>
<taxon idref="inermeM004"/>
T-------------------------------------
TCCTGAATGCTTCATTATGTGAATAAGATAT------AGA-T-----------------
GCATGGAGT-ACATAC-TC-
AGTTATTCTAGACTATCAATATATAAA--------------------
TAAG---------------
AAAGTAGACTCATATTGGCTAGTGGCTTCTTCCGGAATGAGAAAGTGAGTTTCACTATCGAT
TCTAGGAT-AAAATG---ATTTATT--------
GATCGTTTTAAAATAGGATTTCAATACATCAATTCGACCCCAA-TTTCTTTT-----
ATGAAATTGATACTC------------ATCAGAACCAAA-
TTCACTTGATAGATGTACCTACCAATTCAAGAAAGATTCCAAGATATTTCA-----------
--------TTAAAAAATGGGATGGAGAAGTTCGATTTGTCCACACAAATTCTTAGATC-
AAAAAA----------TTTCCGAAACTCTCTTCATCCTTC----TTACT------
AGCTTTATCATCCCAGATTTCTTCTTTATTC-------ATTTT------
CTGGTATAGATATTTCGTTTTT-----------------CTAGATTCTA-GAAA---T-
CTATTTTTT----GTT-----ATT-CAAAAGTGAGTTAGATAACAAATC-
AAAAAAGAAA---
CTCATTGTTT--

CGA------
TTTCGGTTCTTACACAATCTTGACAATTTCAAAAAAACGGGTCATACTATGATCATAGTATG

ATGGCGGTTGGACACGTATGCCCCCATCGTCTAGTGGTTCAGGACATCTCTCTTTCAAGGAG
GC--

</sequence>
<sequence>
<taxon idref="koeieiW153"/>
T-------------------------------------
TCCTGAATGCTTCATTATGTGAATAAGATAT------AGA-T-----------------
GCATGGAGT-ACATAC-TC-
AGTTATTCTAGACTATCAATATATAAA--------------------
TAAG---------------
AAAGTAGACTCATATTGGCTAGTGGCTTCTTCCGGAATGAGAAAGTGAGTTTCACTATCGAT
TCTAGGAT-AAAATG---ATTTATT--------
GATCGTTTTAAAATAGGATTTCAATACATCAATTCGACCCCAA-TTTCTTTT-----
ATGAAATTGATACTC------------ATCAGAACCAAA-
TTCACTTGATAGATGTACCTACCAATTCAAGAAAGATTCCAAGATATTTCA-----------
--------TTAAAAAATGGGATGGAGAAGTTCGATTTGTCCACACAAATTCTTAGATC-
AAAAAA----------TTTCCGAAACTCTCTTCATCCTTC----TTACT------
AGCTTTATCATCCCAGATTTCTTCTTTATTC-------ATTTT------
CTGGTATAGATATTTCGTTTTT-----------------CTAGATTCTA-GAAA---T-
CTATTTTTT----GTT-----ATT-CAAAAGTGAGTTAGATAACAAATC-
AAAAAAGAAA---
CTCATTGTTT--

CGA------
TTTCGGTTCTTACACAATCTTGACAATTTCAAAAAAACGGGTCATACTATGATCATAGTATG
ATGGCGGTTGGACACGTATGCCCCCATCGTCTAGTGGCTCAGGACATCTCTCTTTCAAGGAG
GC--

</sequence>
<sequence>
<taxon idref="leucochl50"/>
T-------------------------------------
TCCTGAATGCTTCATTATGTGAATAAGATAT------AGA-T-----------------
GCATGGAGT-ACATAC-TC-
AGTTATTCTAGACTATCAATATATAAA--------------------
TAAG---------------
AAAGTAGACTCATATTGGCTAGTGGCTTCTTCCGGAATGAGAAAGTGAGTTTCACTATCGAT
TCTAGGAT-AAAATG---ATTTATT--------
GATCGTTTTAAAATAGGATTTCAATACATCAATTCGACCCCAA-TTTCTTTT-----
ATGAAATTGATACTC------------ATCAGAACCAAA-
TTCACTTGATAGATGTACCTACCAATTCAAGAAAGATTCCAAGATATTTCA-----------
--------TTAAAAAATGGGATGGAGAAGTTCGATTTGTCCACACAAATTCTTAGATC-
AAAAAA----------TTTCCGAAACTCTCTTCATCCTTC----TTACT------
AGCTTTATCATCCCAGATTTCTTCTTTATTC-------ATTTT------
CTGGTATAGATATTTCGTTTTT-----------------CTAGATTCTA-GAAA---T-
CTATTTTTT----GTT-----ATT-CAAAAGTGAGTTAGATAACAAATC-
AAAAAAGAAA---
CTCATTGTTT--

CGA------
TTTCGGTTCTTACACAATCTTGACAATTTCAAAAAAACGGGTCATACTATGATCATAGTATG
ATGGCGGTTGGACACGTATGCCCCCATCGTCTAGTGGTTCAGGACATCTCTCTTTCAAGGAG
GCAGCGGGGATTCGACTT--

</sequence>
<sequence>
<taxon idref="lycopoio55"/>
T-------------------------------------
TCCTGAATGCTTCATTATGTGAATAAGATAT------AGA-T-----------------
GCATGGAGT-ACATAC-TC-
AGTTATTCTAGACTATCAATATATAAA--------------------
TAAG---------------
AAAGTAGACTCATATTGGCTAGTGGCTTCTTCCGGAATGAGAAAGTGAGTTTCACTATCGAT
TCTAGGAT-AAAATG---ATTTATT--------
GATCGTTTTAAAATAGGATTTCAATACATCAATTCGACCCCAA-TTTCTTTT-----
ATGAAATTGATACTC------------ATCAGAACCAAA-
TTCACTTGATAGATGTACCTACCAATTCAAGAAAGATTCCAAGATATTTCA-----------
--------TTAAAAAATGGGATGGAGAAGTTCGATTTGTCCACACAAATTCTTAGATC-
AAAAAA----------TTTCCGAAACTCTCTTCATCCTTC----TTACT------
AGCTTTATCATCCCAGATTTCTTCTTTATTC-------ATTTT------
CTGGTATAGATATTTCGTTTTT-----------------CTAGATTCTA-GAAA---T-
CTATTTTTT----GTT-----ATT-CAAAAGTGAGTTATATAACAAATC-
AAAAAAGAAA---
CTCATTTTTT--

CGA------
TTTCGGTTCTTACACAATCTTGACAATTTCAAAAAAACGGGTCATACTATGATCATAGTATG
ATGGCGGTTGGACACGTATGCCCCCATCGTCTAGTGGTTCAGGACATCTCTCTTTCAAGGAG
GCAGCGGGGATTCGACTTCCCCTGGGGG-TAGGTTACTACA-
AAAGAAAGTGAATCATGCATTATGAATAA-------GCCGAAA----
TTGGAATTTATTCTTCCTGGGTCGATGCCCGAGCGGTTAATGGGGACGGACTGTAAATTCGT
TGGCAATATGTCTACGC
</sequence>
<sequence>
<taxon idref="macrathu10"/>
T-------------------------------------
TCCTGAATGCTTCATTATGTGAATAAGATAT------AGA-T-----------------
GCATGGAGT-ACATAC-TC-
AGTTATTCTAGACTATCAATATATAAA--------------------
TAAG---------------
AAAGTAGACTCATATTGGCTAGTGGCTTCTTCCGGAATGAGAAAGTGAGTTTCACTATCGAT
TCTAGGAT-AAAATG---ATTTATT--------
GATCGTTTTAAAATAGGATTTCAATACATCAATTCGACCCCAA-TTTCTTTT-----
ATGAAATTGATACTC------------ATCAGAACCAAA-
TTCACTTGATAGATGTACCTACCAATTCAAGAAAGATTCCAAGATATTTCA-----------
--------TTAAAAAATGGGATGGAGAAGTTCGATTTGTCCACACAAATTCTTAGATC-
AAAAAA----------TTTCCGAAACTCTCTTCATCCTTC----TTACT------
AGCTTTATCATCCCAGATTTCTTCTTTATTC-------ATTTT------
CTGGTATAGATATTTCGTTTTT-----------------CTAGATTCTA-GAAA---T-
CTATTTTTT----GTT-----ATT-CAAAAGTGAGTTATATAACAAATC-
AAAAAAGAAA---
CTCATTTTTT--

CGA------
TTTCGGTTCTTACACAATCTTGACAATTTCAAAAAAACGGGTCATACTATGATCATAGTATG
ATGGCGGTTGGACACGTATGCCCCCATCGTCTAGTGGTTCAGGACATCTCTCTTTCAAGGAG
GCAGCGGGGATTCGACTTCCCCTGGGGG-TAGGTTACTACA-
AAAGAAAGTGAATCATGCATTATGAATAA-------GCCGAAA----
TTGGAATTTATTCTTCCTGGGTCGATGCCCGAGCGGTTAATGGGGACGGACTGTAAATTCGT
TGGCAATATGTCTACGC
</sequence>
<sequence>
<taxon idref="peculiar19"/>
T-------------------------------------
TCCTGAATGCTTCATTATGTGAATAAGATAT------AGA-T-----------------
GCATGGAGT-ACGTAC-TC-
AGTTATTCTAGACTATCAATATATAAA--------------------
TAAG--------------
AAAGTAGACTCATATTGGCTAGTGGCTTCTTCCGGAATGAGAAAGTGAGTTTCACTATCGAT
TCTAGGAT-AAAATG---ATTTATT--------
GATCGTTTTAAAATAGGATTTCAATACATCAATTCGACCCCAA-TTTCTTTT-----
ATGAAATTGATACTC------------ATCAGAACCAAA-
TTCACTTGATAGATGTACCTACCAATTCAAGAAAGATTCCAAGATATTTCA-----------
--------TTAAAAAATGGGATGGAGAAGTTCGATTTGTCCACACAAATTCTTAGATC-
AAAAAA----------TTTCCGAAACTCTCTTCATCCTTC----TTACT------
AGCTTTATCATCCCAGATTTCTTCTTTATTC-------ATTTT------
CTGGTATAGATATTTCGTTTTT-----------------CTAGATTCTA-GAAA---T-
CTATTTTTT----GTT-----ATT-CAAAAGTGAGTTAGATAACAAATC-
AAAAAAGAAA---
CTCATTGTTT--

CGA------
TTTCGGTTCTTACACAATCTTGACAATTTCAAAAAAACGGGTCATACTATGATCATAGTATG
ATGGCGGTTGGACACGTATGCCCCCATCGTCTAGTGGTTCAGGACATCTCTCTTTCAAGGAG
GC--

</sequence>
<sequence>
<taxon idref="physoste55"/>

T--

</sequence>
<sequence>
<taxon idref="pulchell79"/>

T--

</sequence>
<sequence>
<taxon idref="revolutu15"/>
T-------------------------------------
TCCTGAATGCTTCATTATGTGAATAAGATAT------AGA-T-----------------
GCATGGAGT-ACATAC-TC-
AGTTATTCTAGACTATCAATATATAAA--------------------
TAAG---------------
AAAGTAGACTCATATTGGCTAGTGGCTTCTTCCGGAATGAGAAAGTGAGTTTCACTATCGAT
TCTAGGAT-AAAATG---ATTTATT--------
GATCGTTTTAAAATAGGATTTCAATACATCAATTCGACCCCAA-TTTCTTTT-----
ATGAAATTGATACTC------------ATCAGAACCAAA-
TTCACTTGATAGATGTACCTACCAATTCAAGAAAGATTCCAAGATATTTCA-----------
--------TTAAAAAATGGGATGGAGAAGTTCGATTTGTCCACACAAATTCTTAGATC-
AAAAAA----------TTTCCGAAACTCTCTTCATCCTTC----TTACT------
AGCTTTATCATCCCAGATTTCTTCTTTATTC-------ATTTT------
CTGGTATAGATATTTCGTTTTT-----------------CTAGATTCTA-GAAA---T-
CTATTTTTT----GTT-----ATT-CAAAAGTGAGTTATATAACAAATC-
AAAAAAGAAA---
CTCATTTTT---

CGA------
TTTCGGTTCTTACACAATCTTGACAATTTCAAAAAAACGGGTCATACTATGATCATAGTATG
ATGGCGGTTGGACACGTATGCCCCCATCGTCTAGTGGTTCAGGACATCTCTCTTTCAAGGAG
GCAGCGGGGATTCGACTTCCCCTGGGGG-TAGGTTACTACA-
AAAGAAAGTGAATCATGCATTATGAATAA-------GCCGAAA----
TTGGAATTTATTCTTCCTGGGTCGATGCCCGAGCGGTTAATGGGGACGGACTGTAAATTCGT
TGGCAATATGTCTACGC
</sequence>
<sequence>
<taxon idref="sakeniW295"/>
T-------------------------------------
TCCTGAATGCTTCATTATGTGAATAAGATAT------AGA-T-----------------
GCATGGAGT-ACATAC-TC-
AGTTATTCTAGACTATCAATATATAAA--------------------
TAAG---------------
AAAGTAGACTCATATTGGCTAGTGGCTTCTTCCGGAATGAGAAAGTGAGTTTCACTATCGAT
TCTAGGAT-AAAATG---ATTTATT--------
GATCGTTTTAAAATAGGATTTCAATACATCAATTCGACCCCAA-TTTCTTTT-----
ATGAAATTGATACTC------------ATCAGAACCAAA-
TTCACTTGATAGATGTACCTACCAATTCAAGAAAGATTCCAAGATATTTCA-----------
--------TTCAAAAATGGATGGAGAAGTTCGATTTGTCCACACAAATTCTTAGATC-
AAAAAA----------TTTCCGAAACTCTCTTCATCCTTC----TTACT------
AGCTTTATCATCCCAGATTTCTTCTTTATTC-------ATTTT------
CTGGTATAGATATTTCGTTTTT-----------------CTAGATTCTA-GAAA---T-
CTATTTTTT----GTT-----ATT-CAAAAGTGAGTTATATAACAAATC-
AAAAAAGAAA---
CTCATTTTTT--

CGA------
TTTCGGTTCTTACACAATCTTGACAATTTCAAAAAAACGGGTCATACTATGATCATAGTATG
ATGGCGGTTGGACACGTATGCCCCCATCGTCTAGTGGTTCAGGACATCTCTCTTTCAAGGAG
GC--

</sequence>
<sequence>
<taxon idref="schizost31"/>

T--

</sequence>
<sequence>
<taxon idref="senga85995"/>
T-------------------------
TTTTTCGTAAAGTCCTGAATGCTTCATTATGTGAATAAGATAT------AGA-
T------------TAGATGCATGGAGT-----AC-TC-
AGTTATTCTAGACTATCAATATATAAA--------------------
TAAG---------------AAA----
ACTCATATTGGCTAGTGGCTTCTTCCGGAATGAGAAAGTGAATTTCACTATCGATTCTAGGA
T-AAAATG---ATTTATT--------
GATCGTTTTAAAATAGGATTTCAATACATCAATTCGACCCCAA-TTTCTTGG-----
ATGAAATTGATACTC------------ATCAGAACCAAA-
TTCACTTGATAGATGTACCTACCAATTCAAGAAAGATTCCAAGATATTTCA-----------
--------TTAAAAAATGGATGGAGAAGTTCGATTTGTCCACACAAATTCTTAGATC-
AAAAAA----------TTTCCGAAACTCTCTTCATCCTTC----TTACT------
AGCTTTATCATCCCAGATTTCTTCTTTATTC-------ATTTT------
CTGGTATAGATATTTCGTTTTT-----------------CTAGATTCTA-GAAA---T-
CGATTTTTT----GTT-----ATT-CAAAAGTGAGTTATATAACAAATC-
AAAAAAGAAA---
CTCATTTTT---

CGA------
TTTCGGTTCTTACACAATCTTGACAATTTCAAAAAAACGGATCATACTATGATCATAGTATG
ATGGCGGTTGGACACGTATGCCCCCATCGTCTAGTGGTTCAGGACATCTCTCTTTCAAGGAG
GCAGCGGGGATTCGACTTCCCCTGGGGG-TAGGTTACTACA-
AAAGAAAGTGAATCATGCATTATGAATAA-------GCCGAAA----
TTGGAATTTATTCTTGCTGGGTCGATGCCCGAGCGGTTAATGGGGACGGACTGTAAATTCGT
TGGCAATATGTCTACGC
</sequence>
<sequence>
<taxon idref="solidum174"/>

T--

</sequence>
<sequence>
<taxon idref="stereop252"/>
T-------------------------------------
TCCTGAATGCTTCATTATGTGAATAAGATAT------AGA-T-----------------
GCATGGAGT-ACATAC-TC-
AGTTATTCTAGACTATCAATATATAAA--------------------
TAAG---------------
AAAGTAGACTCATATTGGCTAGTGGCTTCTTCCGGAATGAGAAAGTGAGTTTCACTATCGAT
TCTAGGAT-AAAATG---ATTTATT--------
GATCGTTTTAAAATAGGATTTCAATACATCAATTCGACCCCAA-TTTCTTTT-----
ATGAAATTGATACTC------------ATCAGAACCAAA-
TTCACTTGATAGATGTACCTACCAATTCAAGAAAGATTCCAAGATATTTCA-----------
--------TTAAAAAATGGGATGGAGAAGTTCGATTTGTCCACACAAATTCTTAGATC-
AAAAAA----------TTTCCGAAACTCTCTTCATCCTTC----TTACT------
AGCTTTATCATCCCAGATTTCTTCTTTATTC-------ATTTT------
CTGGTATAGATATTTCGTTTTT-----------------CTAGATTCTA-GAAA---T-
CTATTTTTT----GTT-----ATT-CAAAAGTGAGTTAGATAACAAATC-
AAAAAAGAAA---
CTCATTGTTT--

CGA------
TTTCGGTTCTTACACAATCTTGACAATTTCAAAAAAACGGGTCATACTATGATCATAGTATG
ATGGCGGTTGGACACGTATGCCCCCATCGTCTAGTGGTTCAGGACATCTCTCTTTCAAGGAG
GC--

</sequence>
<sequence>
<taxon idref="subulat221"/>
T-------------------------------------
TCCTGAATGCTTCATTATGTGAATAAGATAT------AGA-T-----------------
GCATGGAGT-ACATAC-TC-
AGTTATTCTAGACTATCAATATATAAA--------------------
TAAG---------------
AAAGTAGACTCATATTGGCTAGTGGCTTCTTCCGGAATGAGAAAGTGAGTTTCACTATCGAT
TCTAGGAT-AAAATG---ATTTATT--------
GATCGTTTTAAAATAGGATTTCAATACATCAATTCGACCCCAA-TTTCTTTT-----
ATGAAATTGATACTC------------ATCAGAACCAAA-
TTCACTTGATAGATGTACCTACCAATTCAAGAAAGATTCCAAGATATTTCA-----------
--------TTAAAAAATGGGATGGAGAAGTTCGATTTGTCCACACAAATTCTTAGATC-
AAAAAA----------TTTCCGAAACTCTCTTCATCCTTC----TTACT------
AGCTTTATCATCCCAGATTTCTTCTTTATTC-------ATTTT------
CTGGTATAGATATTTCGTTTTT-----------------CTAGATTCTA-GAAA---T-
CTATTTTTT----GTT-----ATT-CAAAAGTGAGTTATATAACAAATC-
AAAAAAGAAA---

CTCATTTTTT--

CGA------
TTTCGGTTCTTACACAATCTTGACAATTTCAAAAAAACGGGTCATACTATGATCATAGTATG
ATGGCGGTTGGACACGTATGCCCCCATCGTCTAGTGGTTCAGGACATCTCTCTTTCAAGGAG
GCAGCGGGGATTCGACTTCCCCTGGGGG-TAGGTTACTACA-
AAAGAAAGTGAATCATGCATTATGAATAA-------GCCGAAA----
TTGGAATTTATTCTTCCTGGGTCGATGCCCGAGCGGTTAATGGGGACGGACTGTAAATTCGT
TGGCAATATGTCTACGC
</sequence>
<sequence>
<taxon idref="Talagon103"/>
T---------------------
GGCCTTTTTCGTAAATTCCTGAATGCTTCATTATGTGAATAAGATAT------AGA-
T-----------------GCATGGAGT-ACATAC-TC-
AGTTATTCTAGACTATCAATATATAAA--------------------
TAAG---------------
AAAGTAGACTCATATTGGCTAGTGGCTTCTTCCGGAATGAGAAAGTGAGTTTCACTATCGAT
TCTAGGAT-AAAATG---ATTTATT--------
GATCGTTTTAAAATAGGATTTCAATACATCAATTCGACCCCAA-TTTCTTTT-----
ATGAAATTGATACTC------------ATCAGAACCAAA-
TTCACTTGATAGATGTACCTACCAATTCAAGAAAGATTCCAAGATATTTCA-----------
--------TTAAAAAATGGGATGGAGAAGTTCGATTTGTCCACACAAATTCTTAGATC-
AAAAAA----------TTTCCGAAACTCTCTTCATCCTTC----TTACT------
AGCTTTATCATCCCAGATTTCTTCTTTATTC-------ATTTT------
CTGGTATAGATATTTCGTTTTT-----------------CTAGATTCTA-GAAA---T-
CTATTTTTT----GTT-----ATT-CAAAAGTGAGTTATATAACAAATC-
AAAAAAGAAA---
CTCATTTTTT--

CGA------
TTTCGGTTCTTACACAATCTTGACAATTTCAAAAAAACGGGTCATACTATGATCATAGTATG
ATGGCGGTTGGACACGTATGCCCCCATCGTCTAGTGGTTCAGGACATCTCTCTTTCAAGGAG
GCAGCGGGGATTCGACTTCCCCTGGGGG-TAGGTTACTACA-
AAAGAAAGTGAATCATGCATTATGAATAA-------GCCGAAA----
TTGGAATTTATTCTTCCTGGGTCGATGCCCGAGCGGTTAATGGGGACGGACTGTAAATTCGT
TGGCTATATGTCTACGC
</sequence>
<sequence>
<taxon idref="Zaprjaga24"/>

T--

</sequence>
<sequence>
<taxon idref="acerosum08"/>
T-------------------------------------
TCCTGAATGCTTCATTATGTGAATAAGATAT------AGA-T-----------------
GCATGGAGT-----AC-TC-
AGTTATTCTAGACTATCAATATATAAA--------------------
TAAG---------------AAA----
ACTCATATTGGCTAGTGACTTCTTCCGGAATGAGAAAGTGAATTTCACTATCGATTCTAGGA
T-AAAATG---ATTTATT--------
GATCGTTTTAAAATAGGATTTCAATACATCAATTCGACCCCAA-TTTCTTTG-----
ATGAAATTGATACTC------------ATCAGAACCAAA-
TTCACTTGATAGATGTACCTACCAATTCAAGAAAGATTCCAAGATATTTCA-----------
--------TTAAAAAATGGGATGGAGAAGTTCGATTTGTCCACACAAATTCTTAGATC-
AAAAAA----------TTTCCGAAACTCTCTTCATCCTTC----TTACT------
AGCTTTATCATCCCAGATTTCTTCTTTATTC-------ATTTT------
CTGGTATAGATATTTCGTTTTT-----------------CTAGATTCTA-GAAA---T-
CGATTTTTT----GTT-----ATT-CAAAAGTGAGTTATATAACAAATC-
AAAAAAGAAA---
CTCATTTTTT--

CGA------
TTTCGGTTCTTACACAATCTTGACAATTTCAAAAAAACGGATCATACTATGATCATAGTATG
ATGGCGGTTGGACACGTATGCCCCCATCGTCTAGTGGTTCAGGACATCTCTCTTTCAAGGAG
GCAGCGGGGATTCGACTTCCCCTGGGGG-TAGGTTACTACA-
AAAGAAAGTGAATCATGCATTATGAATAA-------GCCGAAA----
TTGGAATTTATTCTTGCTGGGTCGATGCCCGAGCGGTTAATGGGGACGGACTGTAAATTCGT
TGGCAATATGTCTACGC
</sequence>
<sequence>
<taxon idref="alatavic81"/>
T-------------------------------------
TCCTGAATGCTTCATTATGTGAATAAGATAT------AGA-T-----------------
GCATGGAGT-ACATAC-TC-
AGTTATTCTAGACTATCAATATATAAA--------------------
TAAG---------------
AAAGTAGACTCATATTGGCTAGTGGCTTCTTCCGGAATGAGAAAGTGAGTTTCACTATCGAT
TCTAGGAT-AAAATG---ATTTATT--------
GATCGTTTTAAAATAGGATTTCAATACATCAATTCGACCCCAA-TTTCTTTT-----
ATGAAATTGATACTC------------ATCAGAACCAAA-
TTCACTTGATAGATGTACCTACCAATTCAAGAAAGATTCCAAGATATTTCA-----------
--------TTAAAAAATGGGATGGAGAAGTTCGATTTGTCCACACAAATTCTTAGATC-
AAAAAA----------TTTCCGAAACTCTCTTCATCCTTC----TTACT------

AGCTTTATCATCCCAGATTTCTTCTTTATTC-------ATTTT------
CTGGTATAGATATTTCGTTTTT-----------------CTAGATTCTA-GAAA---T-
CTATTTTTT----GTT-----ATT-CAAAAGTGAGTTAGATAACAAATC-
AAAAAAGAAA---
CTCATTGTTT--

CGA------
TTTCGGTTCTTACACAATCTTGACAATTTCAAAAAAACGGGTCATACTATGATCATAGTATG
ATGGCGGTTGGACACGTATGCCCCCATCGTCTAGTGGTTCAGGACATCTCTCTTTCAAGGAG
GC--

</sequence>
<sequence>
<taxon idref="armenum647"/>
T-------------------------------------
TCCTGAATGCTTCATTATGTGAATAAGATAT------AGA-T-----------------
GCATGGAGT-----AC-TC-
AGTTATTCTAGACTATCAATATATAAA--------------------
TAAG---------------AAA----
ACTCATATTGGCTAGTGGCTTCTTCCGGAATGAGAAAGTGAATTTCACTATCGATTCTAGGA
T-AAAATG---ATTTATT--------
GATCGTTTTAAAATAGGATTTCAATACATCAATTCGACCCCAA-TTTCTTTG-----
ATGAAATTGATACTC------------ATCAGAACCAAA-
TTCACTTGATAGATGTACCTACCAATTCAAGAAAGATTCCAAGATATTTCA-----------
--------TTAAAAAATGGGATGGAGAAGTTCGATTTGTCCACACAAATTCTTAGATC-
AAAAAA----------TTTCCGAAACTCTCTTCATCCTTC----TTACT------
AGCTTTATCATCCCAGATTTCTTCTTTATTC-------ATTTT------
CTGGTATAGATATTTCGTTTTT-----------------CTAGATTCTA-GAAA---T-
CGATTTTTT----GTT-----ATT-CAAAAGTGAGTTATATAACAAATC-
AAAAAAGAAA---
CTCATTTTTT--

CGA------
TTTCGGTTCTTACACAATCTTGACAATTTCAAAAAAACGGATCATACTATGATCATAGTATG
ATGGCGGTTGGACACGTATGCCCCCATCGTCTAGTGGTTCAGGACATCTCTCTTTCAAGGAG
GCAGCGGGGATTCGACTTCCCCTGGGGG-TAGGTTACTACA-
AAAGAAAGTGAATCATGCATTATGAATAA-------GCCGAAA----
TTGGAATTTATTCTTGCTGGGTCGATGCCCGAGCGGTTAATGGGGACGGACTGTAAATTCGT
TGGCAATATGTCTACGC
</sequence>
<sequence>
<taxon idref="Takhtaj678"/>
T-------------------------------------
TCCTGAATGCTTCATTATGTGAATAAGATAT------AGA-T-----------------
GCATGGAGT-----AC-TC-
AGTTATTCTAGACTATCAATATATAAA--------------------
TAAG---------------AAA----
ACTCATATTGGCTAGTGGCTTCTTCCGGAATGAGAAAGTGAATTTCACTATCGATTCTAGGA
T-AAAATG---ATTTATT--------
GATCGTTTTAAAATAGGATTTCAATACATCAATTCGACCCCAA-TTTCTTTG-----
ATGAAATTGATACTC------------ATCAGAACCAAA-

TTCACTTGATAGATGTACCTACCAATTCAAGAAAGATTCCAAGATATTTCA-----------
--------TTAAAAAATGGATGGAGAAGTTCGATTTGTCCACACAAATTCTTAGATC-
AAAAAA----------TTTCCGAAACTCTCTTCATCCTTC----TTACT------
AGCTTTATCATCCCAGATTTCTTCTTTATTC-------ATTTT------
CTGGTATAGATATTTCGTTTTT-----------------CTAGATTCTA-GAAA---T-
CGATTTTTT----GTT-----ATT-CAAAAGTGAGTTATATAACAAATC-
AAAAAAGAAA---
CTCATTTTTT--

CGA------
TTTCGGTTCTTACACAATCTTGACAATTTCAAAAAAACGGATCATACTATGATCATAGTATG
ATGGCGGTTGGACACGTATGCCCCCATCGTCTAGTGGTTCAGGACATCTCTCTTTCAAGGAG
GCAGCGGGGATTCGACTTCCCCTGGGGG-TAGGTTACTACA-
AAAGAAAGTGAATCATGCATTATGAATAA-------GCCGAGA----
TTGGAATTTATTCTTGCTGGGTCGATGCCCGAGCGGTTAATGGGGACGGACTGTAAATTCGT
TGGCAATATGTCTACGC
</sequence>
<sequence>
<taxon idref="aulieten67"/>
T-------------------------------------
TCCTGAATGCTTCATTATGTGAATAAGATAT------AGA-T-----------------
GCATGGAGT-ACATAC-TC-
AGTTATTCTAGACTATCAATATATAAA--------------------
TAAG---------------
AAAGTAGACTCATATTGGCTAGTGGCTTCTTCCGGAATGAGAAAGTGAGTTTCACTATCGAT
TCTAGGAT-AAAATG---ATTTATT--------
GATCGTTTTAAAATAGGATTTCAATACATCAATTCGACCCCAA-TTTCTTTT-----
ATGAAATTGATACTC------------ATCAGAACCAAA-
TTCACTTGATAGATGTACCTACCAATTCAAGAAAGATTCCAAGATATTTCA-----------
--------TTAAAAAATGGATGGAGAAGTTCGATTTGTCCACACAAATTCTTAGATC-
AAAAAA----------TTTCCGAAACTCTCTTCATCCTTC----TTACT------
AGCTTTATCATCCCAGATTTCTTCTTTATTC-------ATTTT------
CTGGTATAGATATTTCGTTTTT-----------------CTAGATTCTA-GAAA---T-
CTATTTTTT----GTT-----ATT-CAAAAGTGAGTTAGATAACAAATC-
AAAAAAGAAA---
CTCATTGTTT--

CGA------
TTTCGGTTCTTACACAATCTTGACAATTTCAAAAAAACGGGTCATACTATGATCATAGTATG
ATGGCGGTTGGACACGTATGCCCCCATCGTCTAGTGGTTCAGGACATCTCTCTTTCAAGGAG
GCAGCGGGGATTCGACTTCCCCTGGGGG-TAGGTTACTACA-
AAAGAAAGTGAATCATGCATTATGAATAA-------GCCGAAA----
TTGGAATTTATTCTTCCTGGGTCGATGCCCGAGCGGTTAATGGGGACGGACTGTAAATTCGT
TGGCAATATGTCTACGC
</sequence>
<sequence>
<taxon idref="compact047"/>

T--


```
--------------------------------------------------------------
--------------------------------------------------------------
--------------------------------------------------------------
--------------------------------------------------------------
--------------------------------------------------------------
--------------------------------------------------------------
--------------------------------------------------------------
--------------------------------------------------------------
--------------------------------------------------------------
--------------------------------------------------------------
--------------------------------------------------------------
--------------------------------------------------------------
--------------------------------------------------------------
--------------------------------------------------------------
--------------------------------------------------------------
--------------------------------------------------------------
--------------------------------------------------------------
------------
           </sequence>
           <sequence>
                <taxon idref="diapens252"/>

     T--------------------------------------------------------
--------------------------------------------------------------
--------------------------------------------------------------
--------------------------------------------------------------
--------------------------------------------------------------
--------------------------------------------------------------
--------------------------------------------------------------
--------------------------------------------------------------
--------------------------------------------------------------
--------------------------------------------------------------
--------------------------------------------------------------
--------------------------------------------------------------
--------------------------------------------------------------
--------------------------------------------------------------
--------------------------------------------------------------
--------------------------------------------------------------
--------------------------------------------------------------
--------------------------------------------------------------
--------------------------------------------------------------
--------------------------------------------------------------
--------------------------------------------------------------
------------
           </sequence>
           <sequence>
                <taxon idref="ecae024754"/>
                T-------------------------------------
TCCTGAATGCTTCATTATGTGAATAAGATAT------AGA-T-----------------
GCATGGAGT-ACATAC-TC-
AGTTATTCTAGACTATCAATATATAAA--------------------
TAAG---------------
AAAGTAGACTCATATTGGCTAGTGGCTTCTTCCGGAATGAGAAAGTGAATTTCACTATCGAT
TCTAGGAT-AAAATG---ATTTATT--------
GATCGTTTTAAAATAGGATTTCAATACATCAATTCGACCCCAA-TTTCTTTT-----
ATGAAATTGATACTC------------ATCAGAACCAAA-
```

TTCACTTGATAGATGTACCTACCAATTCAAGAAAGATTCCAAGATATTTCA-----------
--------TTAAAAAATGGGATGGAGAAGTTCGATTTGTCCACACAAATTCTTAGATC-
AAAAAA----------TTTCCGAAACTCTCTTCATCCTTC----TTACT------
AGCTTTATCATCCCAGATTTCTTCTTTATTC-------ATTTT-------
CTGGTATAGATATTTCGTTTTT----------------CTAGATTCTA-GAAA---T-
CTATTTTTT----GTT-----ATT-CAAAAGTGAGTTATATAACAAATC-
AAAAAAGAAA---
CTCATTTTTT--

CGA------
TTTCGGTTCTTACACAATCTTGACAATTTCAAAAAAACGGGTCATACTATGATCATAGTATG
ATGGCGGTTGGACACGTATGCCCCCATCGTCTAGTGGTTCAGGACATCTCTCTTTCAAGGAG
GCAGCGGGGATTCGACTTCCCCTGGGGG-TAGGTTACTACA-
AAAGAAAGTGAATCATGCATTATGAATAA-------GCCGAAA----
TTGGAATTTATTCTTCCTGGGTCGATGCCCGAGCGGTTAATGGGGACGGACTGTAAATTCGT
TGGCTATATGTCTACGC
</sequence>
<sequence>
<taxon idref="ekatheri80"/>

C--

</sequence>
<sequence>
<taxon idref="ekbergia42"/>
T-------------------------------------
TCCTGAATGCTTCATTATGTGAATAAGATATTAGATTAGA-T-----------------
GCATGGAGT-ACATAC-TC-
AGTTATTCTAGACTATCAATATATAAA--------------------
TAAG---------------AAAA----
CTCATATTGGCTAGTGGCTTCTTCCGGAATGAGAAAGTGAATTTCACTATCGATTCTAGGAT
-AAAATG---ATTTATT--------
GATCGTTTTAAAATAGGATTTCAATACATCAATTCGACCCCAA-TTTCTTTG-----
ATGAAATTGATACTC------------ATCAGAACCAAA-

TTCACTTGATAGATGTACCTACCAATTCAAGAAAGATTCCAAGATATTTCA-----------
--------TTAAAAAATGGGATGGAGAAGTTCGATTTGTCCACACAAATTCTTAGATC-
AAAAAA----------TTTCCGAAACTCTCTTCATCCTTC----TTACT------
AGCTTTATCATCCCAGATTTCTTCTTTATTC-------ATTTT-------
CTGGTATAGATATTTCGTTTTT----------------CTAGATTCTA-GAAA---T-
CTATTTTTT----GTT-----ATT-CAAAAGTGAGTTATATAACAAATC-
AAAAAAGAAA---
CTCATTTTTT--

CGA------
TTTCGGTTCTTACACAATCTTGACAATTTCAAAAAAACGGATCATACTATGATCATAGTATG
ATGGCGGTTGGACACGTATGCCCCCATCGTCTAGTGGTTCAGGACATCTCTCTTTCAAGGAG
GCAGCGGGGATTCGACTTCCCCTGGGGG-TAGGTTACTACA-
AAAGAAAGTGAATCATGCATTATGAATAA-------GCCGAAA----
TTGGAATTTATTCTTGCTGGGTCGATGCCCGAGCGGTTAATGGGGACGGACTGTAAATTCGT
TGGCAATATGTCTACGC
</sequence>
<sequence>
<taxon idref="erythrae98"/>
T-------------------------------------
TCCTGAATGCTTCATTATGTGAATAAGATAT------AGA-T-----------------
GCATGGAGT-ACATAC-TC-
AGTTATTCTAGACTATCAATATATAAA--------------------
TAAG---------------
AAAGTAGACTCATATTGGCTAGTGGCTTCTTCCGGAATGAGAAAGTGAGTTTCACTATCGAT
TCTAGGAT-AAAATG---ATTTATT--------
GATCGTTTTAAAATAGGATTTCAATACATCAATTCGACCCCAA-TTTCTTTT-----
ATGAAATTGATACTC------------ATCAGAACCAAA-
TTCACTTGATAGATGTACCTACCAATTCAAGAAAGATTCCAAGATATTTCA-----------
--------TTAAAAAATGGGATGGAGAAGTTCGATTTGTCCACACAAATTCTTAGATC-
AAAAAA----------TTTCCGAAACTCTCTTCATCCTTC----TTACT------
AGCTTTATCATCCCAGATTTCTTCTTTATTC-------ATTTT------
CTGGTATAGATATTTCGTTTTT-----------------CTAGATTCTA-GAAA---T-
CTATTTTTT----GTT-----ATT-CAAAAGTGAGTTATATAACAAATC-
AAAAAAGAAA---
CTCATTTTTT--

CGA------
TTTCGGTTCTTACACAATCTTGACAATTTCAAAAAAACGGGTCATACTATGATCATAGTATG
ATGGCGGTTGGACACGTATGCCCCCATCGTCTAGTGGTTCAGGACATCTCTCTTTCAAGGAG
GCAGCGGGGATTCGACTTCCCCTGGGGG-TAGGTTACTACA-
AAAGAAAGTGAATCATGCATTATGAATAA-------GCCGAAA----
TTGGAATTTATTCTTCCTGGGTCGATGCCCGAGCGGTTAATGGGGACGGACTGTAAATTCGT
TGGCAATATGTCTACGC
</sequence>
<sequence>
<taxon idref="federov277"/>
T-------------------------------------
TCCTGAATGCTTCATTATGTGAATAAGATAT------AGA-T-----------------
GCATGGAGT-ACATAC-TC-
AGTTATTCTAGACTATCAATATATAAA--------------------
TAAG---------------

AAAGTAGACTCATATTGGCTAGTGGCTTCTTCCGGAATGAGAAAGTGAGTTTCACTATCGAT
TCTAGGAT-AAAATG---ATTTATT--------
GATCGTTTTAAAATAGGATTTCAATACATCAATTCGACCCCAA-TTTCTTTT-----
ATGAAATTGATACTC------------ATCAGAACCAAA-
TTCACTTGATAGATGTACCTACCAATTCAAGAAAGATTCCAAGATATTTCA-----------
--------TTAAAAAATGGGATGGAGAAGTTCGATTTGTCCACACAAATTCTTAGATC-
AAAAAA----------TTTCCGAAACTCTCTTCATCCTTC----TTACT------
AGCTTTATCATCCCAGATTTCTTCTTTATTC-------ATTTT------
CTGGTATAGATATTTCGTTTTT-----------------CTAGATTCTA-GAAA---T-
CTATTTTTT----GTT-----ATT-CAAAAGTGAGTTATATAACAAATC-
AAAAAAGAAA---
CTCATTTTTT--

CGA------
TTTCGGTTCTTACACAATCTTGACAATTTCAAAAAAACGGGTCATACTATGATCATAGTATG
ATGGCGGTTGGACACGTATGCCCCCATCGTCTAGTGGTTCAGGACATCTCTCTTTCAAGGAG
GCAGCGGGGATTCGACTTCCCCTGGGGG-TAGGTTACTACA-
AAAGAAAGTGAATCATGCATTATGAATAA-------GCCGAAA----
TTGGAATTTATTCTTCCTGGGTCGATGCCCGAGCGGTTAATGGGGACGGACTGTAAATTCGT
TGGCAATATGTCTACGC
</sequence>
<sequence>
<taxon idref="glumace690"/>
T-------------------------------------
TCCTGAATGCTTCATTATGTGAATAAGATAT------AGA-T-----------------
GCATGGAGT-ACATAC-TC-
AGTTATTCTAGACTATCAATATATAAA--------------------
TAAG---------------
AAAGTAGACTCATATTGGCTAGTGGCTTCTTCCGGAATGAGAAAGTGAGTTTCACTATCGAT
TCTAGGAT-AAAATG---ATTTATT--------
GATCGTTTTAAAATAGGATTTCAATACATCTATTCGACCCCAA-TTTCTTTT-----
ATGAAGTTGATACTC-AATTGATACTCGTCACAACCACA-
TTCCCTTGATAGATGTACCTACCAATTCAAGAAAGATTCCAAGATATTTCA-----------
--------TTAAAAAATGGGATGGAGAAGTTCGATTTGTCCACACAAATTCTTAGATC-
AAAAAA----------TTTCCGAAACTCTCTTCATCCTTC----TTACT------
AGCTTTATCATCCCAGATTTCTTCTTTATTC-------ATTTT------
CTGGTATAGATATTTCGTTTTT-----------------CTAGATTCTA-GAAA---T-
CTATTTTTT----GTT-----ATT-CAAAAGTGAGTTAGATAACAAATC-
AAAAAAGAAA---
CTCATTGTTT--

CGA------
TTTCGGTTCTTACACAATCTTGACAATTTCAAAAAAACGGGTCATACTATGATCATAGTATG
ATGGCGGTTGGACACGTATGCCCCCATCGTCTAGTGGTTCAGGACATCTCTCTTTCAAGGAG
GCAGCGGGGATTCGACTTCCCCTGGGGG-TAGGTTACTACA-
AAAGAAAGTGAATCATGCATTATGAATAA-------GCCGAAA----
TTGGAATTTATTCTTCCTGGGTCGATGCCCGAGCGGTTAATGGGGACGGACTGTAAATTCGT
TGGCAATATGTCTACGC
</sequence>
<sequence>
<taxon idref="glutinos37"/>
T-------------------------------------

TCCTGAATGCTTCATTATGTGAATAAGATAT------AGA-T-----------------
GCATGGAGT-ACATAC-TC-
AGTTATTCTAGACTATCAATATATAAA--------------------
TAAG---------------
AAAGTAGACTCATATTGGCTAGTGGCTTCTTCCGGAATGAGAAAGTGAGTTTCACTATCGAT
TCTAGGAT-AAAATG---ATTTATT--------
GATCGTTTTAAAATAGGATTTCAATACATCAATTCGACCCCAA-TTTCTTTT-----
ATGAAATTGATACTC------------ATCAGAACCAAA-
TTCACTTGATAGATGTACCTACCAATTCAAGAAAGATTCCAAGATATTTCA-----------
--------TTAAAAAATGGGATGGAGAAGTTCGATTTGTCCACACAAATTCTTAGATC-
AAAAAA----------TTTCCGAAACTCTCTTCATCCTTC----TTACT------
AGCTTTATCATCCCAGATTTCTTCTTTATTC-------ATTTT------
CTGGTATAGATATTTCGTTTTT-----------------CTAGATTCTA-GAAA---T-
CTATTTTTT----GTT-----ATT-CAAAAGTGAGTTATATAACAAATC-
AAAAAAGAAA---
CTCATTTTT---

CGA------
TTTCGGTTCTTACACAATCTTGACAATTTCAAAAAAACGGGTCATACTATGATCATAGTATG
ATGGCGGTTGGACACGTATGCCCCCATCGTCTAGTGGTTCAGGACATCTCTCTTTCAAGGAG
GCAGCGGGGATTCGACTTCCCCTGGGGG-TAGGTTACTACA-
AAAGAAAGTGAATCATGCATTATGAATAA-------GCCGAAA----
TTGGAATTTATTCTTCCTGGGTCGATGCCCGAGCGGTTAATGGGGACGGACTGTAAATTCGT
TGGCAATATGTCTACGC
</sequence>
<sequence>
<taxon idref="hypochar7h"/>
T-------------------------------------
TCCTGAATGCTTCATTATGTGAATAAGATAT------AGA-T-----------------
GCATGGAGT-ACATAC-TC-
AGTTATTCTAGACTATCAATATATAAA--------------------
TAAG---------------
AAAGTAGACTCATATTGGCTAGTGGCTTCTTCCGGAATGAGAAAGTGAGTTTCACTATCGAT
TCTAGGAT-AAAATG---ATTTATT--------
GATCGTTTTAAAATAGGATTTCAATACATCAATTCGACCCCAA-TTTCTTTT-----
ATGAAATTGATACTC------------ATCAGAACCAAA-
TTCACTTGATAGATGTACCTACCAATTCAAGAAAGATTCCAAGATATTTCA-----------
--------TTAAAAAATGGGATGGAGAAGTTCGATTTGTCCACACAAATTCTTAGATC-
AAAAAA----------TTTCCGAAACTCTCTTCATCCTTC----TTACT------
AGCTTTATCATCCCAGATTTCTTCTTTATTC-------ATTTT------
CTGGTATAGATATTTCGTTTTT-----------------CTAGATTCTA-GAAA---T-
CTATTTTTT----GTT-----ATT-CAAAAGTGAGTTAGATAACAAATC-
AAAAAAGAAA---
CTCATTGTTT--

CGA------
TTTCGGTTCTTACACAATCTTGACAATTTCAAAAAAACGGGTCATACTATGATCATAGTATG
ATGGCGGTTGGACACGTATGCCCCCATCGTCTAGTGGTTCAGGACATCTCTCTTTCAAGGAG
GCAGCGGGGATTCGACTTCCCCTGGGGG-TAGGTTACTACA-
AAAGAAAGTGAATCATGCATTATGAATAA-------GCCGAAA----
TTGGAATTTATTCTTCCTGGGTCGATGCCCGAGCGGTTAATGGGGACGGACTGTAAATTCGT
TGGCAATATGTCTACGC

</sequence>
<sequence>
<taxon idref="knorri9KYG"/>
T-------------------------------------
TCCTGAATGCTTCATTATGTGAATAAGATAT------AGA-T-----------------
GCATGGAGT-ACATAC-TC-
AGTTATTCTAGACTATCAATATATAAA--------------------
TAAG---------------
AAAGTAGACTCATATTGGCTAGTGGCTTCTTCCGGAATGAGAAAGTGAGTTTCACTATCGAT
TCTAGGAT-AAAATG---ATTTATT--------
GATCGTTTTAAAATAGGATTTCAATACATCAATTCGACCCCAA-TTTCTTTT-----
ATGAAATTGATACTC------------ATCAGAACCAAA-
TTCACTTGATAGATGTACCTACCAATTCAAGAAAGATTCCAAGATATTTCA-----------
--------TTCAAAAATGGGATGGAGAAGTTCGATTTGTCCACACAAATTCTTAGATC-
AAAAAA----------TTTCCGAAACTCTCTTCATCCTTC----TTACT------
AGCTTTATCATCCCAGATTTCTTCTTTATTC-------ATTTT------
CTGGTATAGATATTTCGTTTTT-----------------CTAGATTCTA-GAAA---T-
CTATTTTTT----GTT-----ATT-CAAAAGTGAGTTATATAACAAATC-
AAAAAAGAAA---
CTCATTTTTT--

CGA------
TTTCGGTTCTTACACAATCTTGACAATTTCAAAAAAACGGGTCATACTATGATCATAGTATG
ATGGCGGTTGGACACGTATGCCCCCATCGTCTAGTGGTTCAGGACATCTCTCTTTCAAGGAG
GC--

</sequence>
<sequence>
<taxon idref="laxuW2055K"/>
T-------------------------------------
TCCTGAATGCTTCATTATGTGAATAAGATAT------AGA-T-----------------
GCATGGAGT-ACATAC-TC-
AGTTATTCTAGACTATCAATATATAAA--------------------
TAAG---------------
AAAGTAGACTCATATTGGCTAGTGGCTTCTTCCGGAATGAGAAAGTGAGTTTCACTATCGAT
TCTAGGAT-AAAATG---ATTTATT--------
GATCGTTTTAAAATAGGATTTCAATACATCAATTCGACCCCAA-TTTCTTTT-----
ATGAAATTGATACTC------------ATCAGAACCAAA-
TTCACTTGATAGATGTACCTACGAATTCAAGAAAGATTCCAAGATATTTCA-----------
--------TTAAAAAATGGGATGGAGAAGTTCGATTTGTCCACACAAATTCTTAGATC-
AAAAAA----------TTTCCGAAACTCTCTTCATCCTTC----TTACT------
AGCTTTATCATCCCAGATTTCTTCTTTATTC-------ATTTT------
CTGGTATAGATATTTCGTTTTT-----------------CTAGATTCTA-GAAA---T-
CTATTTTTT----GTT-----ATT-CAAAAGTGAGTTAGATAACAAATC-
AAAAAAGAAA---
CTCATTGTTT--

CGA------
TTTCGGTTCTTACACAATCTTGACAATTTCAAAAAAACGGGTCATACTATGATCATAGTATG
ATGGCGGTTGGACACGTATGCCCCCATCGTCTAGTGGTTCAGGACATCTCTCTTTCAAGGAG
GCAGCGGGGATTCGACTTCCCCTGGGGG-TAGGTTACTACA-

AAAGAAAGTGAATCATGCATTATGAATAA-------GCCGAAA----
TTGGAATTTATTCTTCCTGGGTCGATGCCCGAGCGGTTAATGGGGACGGACTGTAAATTCGT
TGGCAATATGTCTACGC
</sequence>
<sequence>
<taxon idref="leucant552"/>
T-------------------------------------
TCCTGAATGCTTCATTATGTGAATAAGATAT------AGA-T-----------------
GCATGGAGT-ACATAC-TC-
AGTTATTCTAGACTATCAATATATAAA--------------------
TAAG---------------
AAAGTAGACTCATATTGGCTAGTGGCTTCTTCCGGAATGAGAAAGTGAATTTCACTATCGAT
TCTAGGAT-AAAATG---ATTTATT--------
GATCGTTTTAAAATAGGATTTCAATACATCAATTCGACCCCAA-TTTCTTTT-----
ATGAAATTGATACTC------------ATCAGAACCAAA-
TTCACTTGATAGATGTACCTACCAATTCAAGAAAGATTCCAAGATATTTCA-----------
--------TTAAAAAATGGGATGGAGAAGTTCGATTTGTCCACACAAATTCTTAGATC-
AAAAAA----------TTTCCGAAACTCTCTTCATCCTTC----TTACT------
AGCTTTATCATCCCAGATTTCTTCTTTATTC-------ATTTT------
CTGGTATAGATATTTCGTTTTT-----------------CTAGATTCTA-GAAA---T-
CTATTTTTT----GTT-----ATT-CAAAAGTGAGTTATATAACAAATC-
AAAAAAGAAA---
CTCATTTTT---

CGA------
TTTCGGTTCTTACACAATCTTGACAATTTCAAAAAAACGGGTCATACTATGATCATAGTATG
ATGGCGGTTGGACACGTATGCCCCCATCGTCTAGTGGTTCAGGACATCTCTCTTTCAAGGAG
GCAGCGGGGATTCGACTTCCCCTGGGGG-TAGGTTACTACA-
AAAGAAAGTGAATCATGCATTATGAATAA-------GCCGAAA----
TTGGAATTTATTCTTCCTGGGTCGATGCCCGAGCGGTTAATGGGGACGGACTGTAAATTCGT
TGGCAATATGTCTACGC
</sequence>
<sequence>
<taxon idref="nabievi887"/>
T-------------------------------------
TCCTGAATGCTTCATTATGTGAATAAGATAT------AGA-T-----------------
GCATGGAGT-ACATAC-TC-
AGTTATTCTAGACTATCAATATATAAA--------------------
TAAG---------------
AAAGTAGACTCATATTGGCTAGTGGCTTCTTCCGGAATGAGAAAGTGAGTTTCACTATCGAT
TCTAGGAT-AAAATG---ATTTATT--------
GATCGTTTTAAAATAGGATTTCAATACATCAATTCGACCCCAA-TTTCTTTT-----
ATGAAATTGATACTC------------ATCAGAACCAAA-
TTCACTTGATAGATGTACCTACCAATTCAAGAAAGATTCCAAGATATTTCA-----------
--------TTCAAAAATGGGATGGAGAAGTTCGATTTGTCCACACAAATTCTTAGATC-
AAAAAA----------TTTCCGAAACTCTCTTCATCCTTC----TTACT------
AGCTTTATCATCCCAGATTTCTTCTTTATTC-------ATTTT------
CTGGTATAGATATTTCGTTTTT-----------------CTAGATTCTA-GAAA---T-
CTATTTTTT----GTT-----ATT-CAAAAGTGAGTTATATAACAAATC-
AAAAAAGAAA---
CTCATTTTT---

CGA------
TTTCGGTTCTTACACAATCTTGACAATTTCAAAAAAACGGGTCATACTATGATCATAGTATG
ATGGCGGTTGGACACCTATGCCCCCATCGTCTAGTGGTTCAGGACATCTCTCTTTCAAGGAG
GCAGCGGGGATTCGACTTCCCCTGGGGG-TAGGTTACTACA-
AAAGAAAGTGAATCATGCATTATGAATAA-------GCCGAAA----
TTGGAATTTATTCTTCCTGGGTCGATGCCCGAGCGGTTAATGGGGACGGACTGTAAATTCGT
TGGCAATATGTCTACGC
</sequence>
<sequence>
<taxon idref="sarytavi90"/>
T-------------------------------------
TCCTGAATGCTTCATTATGTGAATAAGATAT------AGA-T-----------------
GCATGGAGT-ACATAC-TC-
AGTTATTCTAGACTATCAATATATAAA--------------------
TAAG---------------
AAAGTAGACTCATATTGGCTAGTGGCTTCTTCCGGAATGAGAAAGTGAGTTTCACTATCGAT
TCTAGGAT-AAAATG---ATTTATT--------
GATCGTTTTAAAATAGGATTTCAATACATCAATTCGACCCCAA-TTTCTTTT-----
ATGAAATTGATACTC------------ATCAGAACCAAA-
TTCACTTGATAGATGTACCTACCAATTCAAGAAAGATTCCAAGATATTTCA-----------
--------TTCAAAAATGGGATGGAGAAGTTCGATTTGTCCACACAAATTCTTAGATC-
AAAAAA----------TTTCCGAAACTCTCTTCATCCTTC----TTACT------
AGCTTTATCATCCCAGATTTCTTCTTTATTC-------ATTTT------
CTGGTATAGATATTTCGTTTTT-----------------CTAGATTCTA-GAAA---T-
CTATTTTTT----GTT-----ATT-CAAAAGTGAGTTATATAACAAATC-
AAAAAAGAAA---
CTCATTTTTT--

CGA------
TTTCGGTTCTTACACAATCTTGACAATTTCAAAAAAACGGGTCATACTATGATCATAGTATG
ATGGCGGTTGGACACGTATGCCCCCATCGTCTAGTGGTTCAGGACATCTCTCTTTCAAGGAG
GCAGCGGGGATTCGACTTCCCCTGGGGG-TAGGTTACTACA-
AAAGAAAGTGAATCATGCATTATGAATAA-------GCCGAAA----
TTGGAATTTATTCTTCCTGGGTCGATGCCCGAGCGGTTAATGGGGACGGACTGTAAATTCGT
TGGCAATATGTCTACGC
</sequence>
<sequence>
<taxon idref="tricol21AN"/>

T--

</sequence>
<sequence>
<taxon idref="venustum99"/>

TAAAGGGCCTTTGAGTTAAAAGGGCCTTTTTCGTAAAGTCCTGAATGCTTCATTATG
TGAATAAGATAT------AGA-T------------TAGATGCATGGAGT-----AC-TC-
AGTTATTCTAGACTATCAATATATAAA--------------------
TAAG---------------AAA----
ACTCATATTGGCTAGTGGCTTCTTCCGGAATGAGAAAGTGAATTTCACTATCGATTCTAGGA
T-AAAATG---ATTTATT--------
GATCGTTTTAAAATAGGATTTCAATACATCAATTCGACCCCAA-TTTCTTTG-----
ATGAAATTGATACTC------------ATCAGAACCAAA-
TTCACTTGATAGATGTACCTACCAATTCAAGAAAGATTCCAAGATATTTCA-----------
--------TTAAAAAATGGATGGAGAAGTTCGATTTGTCCACACAAATTCTTAGATC-
AAAAAA----------TTTCCGAAACTCTCTTCATCCTTC----TTACT------
AGCTTTATCATCCCAGATTTCTTCTTTATTC-------ATTTT------
CTGGTATAGATATTTCGTTTTT-----------------CTAGATTCTA-GAAA---T-
CGATTTTTT----GTT-----ATT-CAAAAGTGAGTTATATAACAAATC-
AAAAAAGAAA---
CTCATTTTT---

CGA------
TTTCGGTTCTTACACAATCTTGACAATTTCAAAAAAACGGATCATACTATGATCATAGTATG
ATGGCGGTTGGACACGTATGCCCCCATCGTCTAGTGGTTCAGGACATCTCTCTTTCAAGGAG
GCAGCGGGGATTCGACTTCCCCTGGGGG-TAGGTTACTACA-
AAAGAAAGTGAATCATGCATTATGAATAA-------GCCGAAA----
TTGGAATTGATTCTTGCTGGGTCGATGCCCGAGCGGTTAATGGGGACGGACTGTAAATTCGT
TGGCAATATGTCTACGC
</sequence>
<sequence>
<taxon idref="ulicinum79"/>
T-------------------------------------
TCCTGAATGCTTCATTATGTGAATAAGATAT------AGA-T-----------------
GCATGGAGT-ACATAC-TC-
AGTTATTCTAGACTATCAATATATAAA--------------------
TAAG---------------
AAAGTAGACTCATATTGGCTAGTGGCTTCTTCCGGAATGAGAAAGTGAGTTTCACTATCGAT
TCTAGGAT-AAAATG---ATTTATT--------
GATCGTTTTAAAATAGGATTTCAATACATCAATTCGACCCCAA-TTTCTTTT-----
ATGAAATTGATACTC-AATTGATACTCATCAGAACCAAA-
TTCACTTGATAGATGTACCTACCAATTCAAGAAAGATTCCAAGATATTTCA-----------
--------TTAAAAAATGGATGGAGAAGTTCGATTTGTCCACACAAATTCTTAGATC-
AAAAAA----------TTTCCGAAACTCTCTTCATCCTTC----TTACT------
AGCTTTATCATCCCAGATTTCTTCTTTATTC-------ATTTT------
CTGGTATAGATATTTCGTTTTT-----------------CTAGATTCTA-GAAA---T-
CTATTTTTT----GTT-----ATT-CAAAAGTGAGTTAGATAACAAATC-
AAAAAAGAAA---

CTCATTGTTT--

CGA------
TTTCGGTTCTTACACAATCTTGACAATTTCAAAAAAACGGGTCATACTATGATCATAGTATG
ATGGCGGTTGGACACGTATGCCCCCATCGTCTAGTGGTTCAGGACATCTCTCTTTCAAGGAG
GCAGCGGGGATTCGACTTCCCTGGGGG-TAGGTTACTACA-
AAAGAAAGTGAATCATGCATTATGAATAA-------GCCGAAA----
TTGGAATTTATTCTTCCTGGGTCGATGCCCGAGCGGTTAATGGGGACGGACTGTAAATTCGT
TGGCAATATGTCTACGC
</sequence>
<sequence>
<taxon idref="wiedema346"/>
T-------------------------------------
TCCTGAATGCTTCATTATGTGAATAAGATAT------AGA-T-----------------
GCATGGAGT-ACATAC-TC-
AGTTATTCTAGACTATCAATATATAAA--------------------
TAAG---------------
AAAGTAGACTCATATTGGCTAGTGGCTTCTTCCGGAATGAGAAAGTGAGTTTCACTATCGAT
TCTAGGAT-AAAATG---ATTTATT--------
GATCGTTTTAAAATAGGATTTCAATACATCAATTCGACCCCAA-TTTCTTTT-----
ATGAAATTGATACTCAAATTGAGACTCATCAGAACCAAA-
TTCACTTGATAGATGTACCTACCAGTTCAAGAAAGATTCCAAGATATTTCA-----------
--------TTAAAAAATGGGATGGAGAAGTTCGATTTGTCCACACAAATTCTTAGATC-
AAAAAA----------TTTCCGAAACTCTCTTCATCCTTC----TTACT------
AGCTTTATCATCCCAGATTTCTTCTTTATTC-------ATTTT------
CTGGTATAGATATTTCGTTTTT-----------------CTAGATTCTA-GAAA---T-
CTATTTTTT----GTT-----ATT-CAAAAGTGAGTTAGATAACAAATC-
AAAAAAGAAA---
CTCATTGTTT--

CGA------
TTTCGGTTCTTACACAATCTTGACAATTTCAAAAAAACGGGTCATACTATGATCATAGTATG
ATGGCGGTTGGACACGTATGCCCCCATCGTCTAGTGGCTCAGGACATCTCTCTTTCAAGGAG
GCAGCGGGGATTCGACTTCCCTGGGGG-TAGGTTACTACA-
AAAGAGAGTGAATCATGCATTATGAATAA-------GCCGAAA----
TTGGAATTTATTCTTCCTGGGTCGATGCCCGAGGCGTTAATGGGGACGGACTGTAAATTCGT
TGGCAATATGTCTACGC
</sequence>
<sequence>
<taxon idref="AcaAraxanu"/>
T-------------------------------------
TCCTGAATGCTTCATTATGTGAATAAGATAT------AGA-T------------
TAGATGCATGGAGT-----AC-TC-
AGTTATTCTAGACTATCAATATATAAA--------------------
TAAG---------------AAA----
ACTCATATTGGCTAGTGGCTTCTTCCGGAATGAGAAAGTGAATTTCACTATCGATTCTAGGA
T-AAAATG---ATTTATT--------
GATCGTTTTAAAATAGGATTTCAATACATCAATTCGACCCCAA-TTTCTTTG-----
ATGAAATTGATACTC------------ATCAGAACCAAA-
TTCACTTGATAGATGTACCTACCAATTCAAGAAAGATTCCAAGATATTTCA-----------
--------TTAAAAAATGGGATGGAGAAGTTCGATTTGTCCACACAAATTCTTAGATCC-
AAAAA----------TTTCCGAAACTCTCTTCATCCTTC----TTACT------

AGCTTTATCATCCCAGATTTATTCTTTATTC-------ATTTT------
CTGGTATAGATATTTCGTTTTT-----------------CTAGATTCTA-GAAA---T-
CGATTTTTT----GTT-----ATT-CAAAAGTGAGTTATATAACAAATC-
AAAAAAGAAA---
CTCATTTTT---

CGA------
TTTCGGTTCTTACACAATCTTGACAATTTCAAAAAAACGGATCATACTATGATCATAGTATG
ATGGCGGTTGGACACGTATGCCCCCATCGTCTAGTGGTTCAGGACATCTCTCTTTCAAGGAG
GCAGCGGGGATTCGACTTCCCCTGGGGG-TAGGTTACTACA-
AAAGAAAGTGAATCATGCATTATGAATAA-------GCCGAAA----
TTGGAATTTATTCTTGCTGGGTCGATGCCCGAGCGGTTAATGGGGACGGACTGTAAATTCGT
TGGCAATATGTCTACGC
</sequence>
<sequence>
<taxon idref="AcaFlabeum"/>

TCCTGAATGCTTCATTATGTGAATAAGATAT------AGA-T-----------------
GCATGGAGT-ACATAC-TC-
AGTTATTCTAGACTATCAATATATAAA--------------------
TAAG---------------
AAAGTAGACTCATATTGGCTAGTGGCTTCTTCCGGAATGAGAAAGTGAGTTTCACTATCGAT
TCTAGGAT-AAAATG---ATTTATT--------
GATCGTTTTAAAATAGGATTTCAATACATCAATTCGACCCCAA-TTTCTTTT-----
ATGAAATTGATACTC------------ATCAGAACCAAA-
TTCACTTGATAGATGTACCTACCAATTCAAGAAAGATTCCAAGATATTTCA-----------
--------TTAAAAAATGGGATGGAGAAGTTCGATTTGTCCACACAAATTCTTAGATC-
AAAAAA----------TTTCCGAAACTCTCTTCATCCTTC----TTACT------
AGCTTTATCATCCCAGATTTCTTCTTTATTC-------ATTTT------
CTGGTATAGATATTTCGTTTTT-----------------CTAGATTCTA-GAAA---T-
CTATTTTTT----GTT-----ATT-CAAAAGTGAGTTATATAACAAATC-
AAAAAAGAAA---
CTCATTTTT---

CGA------
TTTCGGTTCTTACACAATCTTGACAATTTCAAAAAAACGGGTCATACTATGATCATAGTATG
ATGGCGGTTGGACACGTATGCCCCCATCGTCTAGTGGTTCAGGACATCTCTCTTTCAAGGAG
GCAGCGGGGATTCGACTTCCCCTGGGGG-TAGGTTACTACA-
AAAGAAAGTGAATCATGCATTATGAATAA-------GCCGAAA----
TTGGAATTTATTCTTCCTGGGTCGATGCCCGAGCGGTTAATGGGGACGGACTGTAAATTCGT
TGGCAATATGTCTACGC
</sequence>
<sequence>
<taxon idref="Balohani75"/>
T-------------------------------------
TCCTGAATGCTTCATTATGTGAATAAGATAT------AGA-T-----------------
GCATGGAGT-ACATAC-TC-
AGTTATTCTAGACTATCAATATATAAA--------------------
TAAG---------------
AAAGTAGACTCATATTGGCTAGTGGCTTCTTCCGGAATGAGAAAGTGAATTTCACTATCGAT
TCTAGGAT-AAAATG---ATTTATT--------
GATCGTTTTAAAATAGGATTTCAATACATCAATTCGACCCCAA-TTTCTTTT-----

ATGAAATTGATACTC------------ATCAGAACCAAA-
TTCACTTGATAGATGTACCTACCAATTCAAGAAAGATTCCAAGATATTTCA-----------
--------TTAAAAAATGGATGGAGAAGTTCGATTTGTCCACACAAATTCTTAGATCC-
AAAAA----------TTTCCGAAACTCTCTTCATCCTTC----TTACT------
AACTTTATCATCCCAGATTTCTTCTTTATTC-------ATTTT------
CTGGTATAGATATTTCGTTTTT-----------------CTAGATTCTA-GAAA---T-
CTATTTTTT----GTT-----ATT-CAAAAGTGAGTTATATAACAAATC-
AAAAAAGAAA---
CTCATTTTTT--

CGA------
TTTCGGTTCTTACACAATCTTGACAATTTCAAAAAAAGGGGTCATACTATGATCATAGTATG
ATGGCGGTTGGACACGTATGCCCCCATCGTCTAGTGGTTCAGGACATCTCTCTTTCAAGGAG
GCAGCGGGGATTCGACTTCCCCTGGGGG-TAGGTTACTACA-
AAAGAAAGTGAATCATGCATTATGAATAA-------GCCGAAA----
TTGGAATTTATTCTTCCTGGGTCGATGCCCGAGCGGTTAATGGGGACGGACTGTAAATTCGT
TGGCAATATGTCTACGC
</sequence>
<sequence>
<taxon idref="Gonitilcum"/>
T-------------------------------------
TCCTGAATGCTTCATTATGTGAATAAGATAT------AGA-T-----------------
GCATGGAGT-ACATAC-TA-
AGTTATTCTAGACTATAAATAGATAAGTATA----------------
TAAG---------------
AAAGTAGGCTCATATTGGCTAGTGGCTTCTTCCGGAATGAGAAAGTGAATTTCACTATCGAT
TCTAGGAT-AAAATG---ATTTTTT--------
GATCGTTTTCAAATAGGACTTCAATACATCAATTCGACCCCAA-TTTCTTTT-----
ATGAAATGGATACTC------------ATCAGAACCAAA-
TTCACTTGATACATGGACTTACCAATTCAAGATAGATTCCAAGATATTTCA-----------
--------TTCAAAAATGGATGGAGAAGTTCGATCTGTCCACACAAATTCTTAGATA-
AAAAAA--

</sequence>
<sequence>
<taxon idref="Gonispecoi"/>
T-------------------------------------
TCCTGAATGCTTCATTATGTGAATAAGATAT------AGA-T-----------------
GCATGGAGT-ACATAC-TA-
AGTTATTCTAGACTATAAATAGATAAGTATATAAGA-----------
TAAG---------------
AAAGTAGACTCATATTGGCTAGTGGCTTCTTCCGGAATGAGAAAGTGAATTTCACTATCGAT

```
TCTAGGAT-AAAATG---ATTTTTT--------
GATCGTTTTCAAATAGGACTTCAATACATCAATTCGACCCCAA-TTTCTTTT-----
ATTAAATGGATACTC------------ATCAGAACCAAA-
TTCACTTGATACATGGACTTACCAATCCAAGATAGATTCCAAGATATTTCA-----------
--------TTCAAAAATGGGATGGAGAAGTTCGATCTGTCCACACAAATTCTTAGATA-
AAAAAA--------------------------------------------------------
--------------------------------------------------------------
--------------------------------------------------------------
--------------------------------------------------------------
--------------------------------------------------------------
--------------------------------------------------------------
--------------------------------------------------------------
--------------------------------------------------------------
--------------------------------------------------------------
--------------------------------------------------------------
--------------------------------------------------------------
--------------------------------------------------------------
--------------------------------------------------------------
-------
            </sequence>
      </alignment>

      <!-- The unique patterns from 1 to end
-->
      <!-- npatterns=509
-->
      <patterns id="ITS.patterns" from="1" strip="false">
            <alignment idref="alignment1"/>
      </patterns>

      <!-- The unique patterns from 1 to end
-->
      <!-- npatterns=454
-->
      <patterns id="CP.patterns" from="1" strip="false">
            <alignment idref="alignment2"/>
      </patterns>

      <!-- A prior on the distribution node heights defined
given                 -->

      <!-- Using birth-death model on tree: Gernhard T (2008) J
Theor Biol, Volume 253, Issue 4, Pages 769-778 In press-->
      <birthDeathModel id="birthDeath" units="years">
            <birthMinusDeathRate>
                  <parameter id="birthDeath.meanGrowthRate"
value="2.0" lower="0.0"/>
            </birthMinusDeathRate>
            <relativeDeathRate>
                  <parameter id="birthDeath.relativeDeathRate"
value="0.5" lower="0.0"/>
            </relativeDeathRate>
      </birthDeathModel>

      <!-- This is a simple constant population size coalescent
model              -->
```

```
	<!-- that is used to generate an initial tree for the
chain.                  -->
	<constantSize id="initialDemo" units="years">
		<populationSize>
			<parameter id="initialDemo.popSize"
value="100.0"/>
		</populationSize>
	</constantSize>

	<!-- Generate a random starting tree under the coalescent
process            -->
	<coalescentSimulator id="startingTree">
		<coalescentSimulator>
			<taxa idref="stem"/>
			<constantSize idref="initialDemo"/>
		</coalescentSimulator>
		<taxa idref="taxa"/>
		<constantSize idref="initialDemo"/>
	</coalescentSimulator>

	<!-- Generate a tree model
-->
	<treeModel id="treeModel">
		<coalescentTree idref="startingTree"/>
		<rootHeight>
			<parameter id="treeModel.rootHeight"/>
		</rootHeight>
		<nodeHeights internalNodes="true">
			<parameter
id="treeModel.internalNodeHeights"/>
		</nodeHeights>
		<nodeHeights internalNodes="true" rootNode="true">
			<parameter
id="treeModel.allInternalNodeHeights"/>
		</nodeHeights>
	</treeModel>

	<!-- Taxon Sets
-->
	<tmrcaStatistic id="tmrca(crown)" includeStem="false">
		<mrca>
			<taxa idref="crown"/>
		</mrca>
		<treeModel idref="treeModel"/>
	</tmrcaStatistic>
	<tmrcaStatistic id="tmrca(stem)" includeStem="false">
		<mrca>
			<taxa idref="stem"/>
		</mrca>
		<treeModel idref="treeModel"/>
	</tmrcaStatistic>
	<monophylyStatistic id="monophyly(stem)">
		<mrca>
			<taxa idref="stem"/>
		</mrca>
		<treeModel idref="treeModel"/>
```

```
      </monophylyStatistic>

      <!-- Generate a speciation likelihood for Yule or Birth
Death                -->
      <speciationLikelihood id="speciation">
           <model>
                <birthDeathModel idref="birthDeath"/>
           </model>
           <speciesTree>
                <treeModel idref="treeModel"/>
           </speciesTree>
      </speciationLikelihood>

      <!-- The uncorrelated relaxed clock (Drummond, Ho,
Phillips & Rambaut (2006) PLoS Biology 4, e88 )-->
      <discretizedBranchRates id="branchRates">
           <treeModel idref="treeModel"/>
           <distribution>
                <logNormalDistributionModel
meanInRealSpace="true">
                     <mean>
                          <parameter id="ucld.mean"
value="1.0" lower="0.0"/>
                     </mean>
                     <stdev>
                          <parameter id="ucld.stdev"
value="0.3333333333333333" lower="0.0"/>
                     </stdev>
                </logNormalDistributionModel>
           </distribution>
           <rateCategories>
                <parameter id="branchRates.categories"/>
           </rateCategories>
      </discretizedBranchRates>
      <rateStatistic id="meanRate" name="meanRate" mode="mean"
internal="true" external="true">
           <treeModel idref="treeModel"/>
           <discretizedBranchRates idref="branchRates"/>
      </rateStatistic>
      <rateStatistic id="coefficientOfVariation"
name="coefficientOfVariation" mode="coefficientOfVariation"
internal="true" external="true">
           <treeModel idref="treeModel"/>
           <discretizedBranchRates idref="branchRates"/>
      </rateStatistic>
      <rateCovarianceStatistic id="covariance"
name="covariance">
           <treeModel idref="treeModel"/>
           <discretizedBranchRates idref="branchRates"/>
      </rateCovarianceStatistic>

      <!-- The general time reversible (GTR) substitution model
-->
      <gtrModel id="gtr">
           <frequencies>
                <frequencyModel dataType="nucleotide">
```

```
                    <alignment idref="alignment1"/>
                    <alignment idref="alignment2"/>
                    <frequencies>
                         <parameter id="frequencies"
dimension="4"/>
                    </frequencies>
               </frequencyModel>
          </frequencies>
          <rateAC>
               <parameter id="ac" value="1.0" lower="0.0"/>
          </rateAC>
          <rateAG>
               <parameter id="ag" value="1.0" lower="0.0"/>
          </rateAG>
          <rateAT>
               <parameter id="at" value="1.0" lower="0.0"/>
          </rateAT>
          <rateCG>
               <parameter id="cg" value="1.0" lower="0.0"/>
          </rateCG>
          <rateGT>
               <parameter id="gt" value="1.0" lower="0.0"/>
          </rateGT>
     </gtrModel>

     <!-- site model
-->
     <siteModel id="siteModel">
          <substitutionModel>
               <gtrModel idref="gtr"/>
          </substitutionModel>
          <gammaShape gammaCategories="4">
               <parameter id="alpha" value="0.5"
lower="0.0"/>
          </gammaShape>
          <proportionInvariant>
               <parameter id="pInv" value="0.5" lower="0.0"
upper="1.0"/>
          </proportionInvariant>
     </siteModel>

     <!-- Likelihood for tree given sequence data
-->
     <treeLikelihood id="ITS.treeLikelihood"
useAmbiguities="false">
          <patterns idref="ITS.patterns"/>
          <treeModel idref="treeModel"/>
          <siteModel idref="siteModel"/>
          <discretizedBranchRates idref="branchRates"/>
     </treeLikelihood>

     <!-- Likelihood for tree given sequence data
-->
     <treeLikelihood id="CP.treeLikelihood"
useAmbiguities="false">
          <patterns idref="CP.patterns"/>
```

```
			<treeModel idref="treeModel"/>
			<siteModel idref="siteModel"/>
			<discretizedBranchRates idref="branchRates"/>
		</treeLikelihood>

		<!-- Define operators
-->
		<operators id="operators" optimizationSchedule="default">
			<scaleOperator scaleFactor="0.75" weight="1">
				<parameter idref="ac"/>
			</scaleOperator>
			<scaleOperator scaleFactor="0.75" weight="1">
				<parameter idref="ag"/>
			</scaleOperator>
			<scaleOperator scaleFactor="0.75" weight="1">
				<parameter idref="at"/>
			</scaleOperator>
			<scaleOperator scaleFactor="0.75" weight="1">
				<parameter idref="cg"/>
			</scaleOperator>
			<scaleOperator scaleFactor="0.75" weight="1">
				<parameter idref="gt"/>
			</scaleOperator>
			<scaleOperator scaleFactor="0.75" weight="1">
				<parameter idref="alpha"/>
			</scaleOperator>
			<scaleOperator scaleFactor="0.75" weight="1">
				<parameter idref="pInv"/>
			</scaleOperator>
			<scaleOperator scaleFactor="0.75" weight="30">
				<parameter idref="ucld.mean"/>
			</scaleOperator>
			<scaleOperator scaleFactor="0.75" weight="30">
				<parameter idref="ucld.stdev"/>
			</scaleOperator>
			<subtreeSlide size="3.4" gaussian="true"
weight="150">
				<treeModel idref="treeModel"/>
			</subtreeSlide>
			<narrowExchange weight="150">
				<treeModel idref="treeModel"/>
			</narrowExchange>
			<wideExchange weight="30">
				<treeModel idref="treeModel"/>
			</wideExchange>
			<wilsonBalding weight="30">
				<treeModel idref="treeModel"/>
			</wilsonBalding>
			<scaleOperator scaleFactor="0.75" weight="30">
				<parameter idref="treeModel.rootHeight"/>
			</scaleOperator>
			<uniformOperator weight="300">
				<parameter
idref="treeModel.internalNodeHeights"/>
			</uniformOperator>
			<scaleOperator scaleFactor="0.75" weight="30">
```

```
                <parameter idref="birthDeath.meanGrowthRate"/>
            </scaleOperator>
            <scaleOperator scaleFactor="0.75" weight="30">
                <parameter
idref="birthDeath.relativeDeathRate"/>
            </scaleOperator>
            <upDownOperator scaleFactor="0.75" weight="30">
                <up>
                    <parameter idref="ucld.mean"/>
                </up>
                <down>
                    <parameter
idref="treeModel.allInternalNodeHeights"/>
                </down>
            </upDownOperator>
            <swapOperator size="1" weight="100"
autoOptimize="false">
                <parameter idref="branchRates.categories"/>
            </swapOperator>
            <uniformIntegerOperator weight="100">
                <parameter idref="branchRates.categories"/>
            </uniformIntegerOperator>
        </operators>

        <!-- Define MCMC
-->
        <mcmc id="mcmc" chainLength="50000000"
autoOptimize="true" operatorAnalysis="beast-xml.ops.txt">
            <posterior id="posterior">
                <prior id="prior">
                    <booleanLikelihood>
                        <monophylyStatistic
idref="monophyly(stem)"/>
                    </booleanLikelihood>
                    <normalPrior mean="41.67" stdev="10.8">
                        <statistic idref="tmrca(crown)"/>
                    </normalPrior>
                    <logNormalPrior mean="0.0" stdev="1.15"
offset="5.33" meanInRealSpace="false">
                        <statistic idref="tmrca(stem)"/>
                    </logNormalPrior>
                    <gammaPrior shape="0.05" scale="10.0"
offset="0.0">
                        <parameter idref="ac"/>
                    </gammaPrior>
                    <gammaPrior shape="0.05" scale="20.0"
offset="0.0">
                        <parameter idref="ag"/>
                    </gammaPrior>
                    <gammaPrior shape="0.05" scale="10.0"
offset="0.0">
                        <parameter idref="at"/>
                    </gammaPrior>
                    <gammaPrior shape="0.05" scale="10.0"
offset="0.0">
                        <parameter idref="cg"/>
```

```
                        </gammaPrior>
                        <gammaPrior shape="0.05" scale="10.0"
offset="0.0">
                              <parameter idref="gt"/>
                        </gammaPrior>
                        <exponentialPrior mean="0.5"
offset="0.0">
                              <parameter idref="alpha"/>
                        </exponentialPrior>
                        <uniformPrior lower="0.0" upper="1.0">
                              <parameter idref="pInv"/>
                        </uniformPrior>
                        <exponentialPrior
mean="0.3333333333333333" offset="0.0">
                              <parameter idref="ucld.stdev"/>
                        </exponentialPrior>
                        <ctmcScalePrior>
                              <ctmcScale>
                                    <parameter idref="ucld.mean"/>
                              </ctmcScale>
                              <treeModel idref="treeModel"/>
                        </ctmcScalePrior>
                        <uniformPrior lower="0.0"
upper="100000.0">
                              <parameter
idref="birthDeath.meanGrowthRate"/>
                        </uniformPrior>
                        <uniformPrior lower="0.0" upper="1.0">
                              <parameter
idref="birthDeath.relativeDeathRate"/>
                        </uniformPrior>
                        <speciationLikelihood
idref="speciation"/>
                  </prior>
                  <likelihood id="likelihood">
                        <treeLikelihood
idref="ITS.treeLikelihood"/>
                        <treeLikelihood
idref="CP.treeLikelihood"/>
                        <discretizedBranchRates
idref="branchRates"/>
                        <discretizedBranchRates
idref="branchRates"/>
                  </likelihood>
            </posterior>
            <operators idref="operators"/>

            <!-- write log to screen
-->
            <log id="screenLog" logEvery="1000">
                  <column label="Posterior" dp="4" width="12">
                        <posterior idref="posterior"/>
                  </column>
                  <column label="Prior" dp="4" width="12">
                        <prior idref="prior"/>
                  </column>
```

```
                <column label="Likelihood" dp="4" width="12">
                     <likelihood idref="likelihood"/>
                </column>
                <column label="rootHeight" sf="6" width="12">
                     <parameter idref="treeModel.rootHeight"/>
                </column>
                <column label="ucld.mean" sf="6" width="12">
                     <parameter idref="ucld.mean"/>
                </column>
           </log>

           <!-- write log to file
-->
           <log id="fileLog" logEvery="1000" fileName="beast-
xml.log.txt" overwrite="false">
                <posterior idref="posterior"/>
                <prior idref="prior"/>
                <likelihood idref="likelihood"/>
                <parameter idref="treeModel.rootHeight"/>
                <tmrcaStatistic idref="tmrca(crown)"/>
                <tmrcaStatistic idref="tmrca(stem)"/>
                <parameter idref="birthDeath.meanGrowthRate"/>
                <parameter
idref="birthDeath.relativeDeathRate"/>
                <parameter idref="ac"/>
                <parameter idref="ag"/>
                <parameter idref="at"/>
                <parameter idref="cg"/>
                <parameter idref="gt"/>
                <parameter idref="alpha"/>
                <parameter idref="pInv"/>
                <parameter idref="ucld.mean"/>
                <parameter idref="ucld.stdev"/>
                <rateStatistic idref="meanRate"/>
                <rateStatistic
idref="coefficientOfVariation"/>
                <rateCovarianceStatistic idref="covariance"/>
                <treeLikelihood idref="ITS.treeLikelihood"/>
                <treeLikelihood idref="CP.treeLikelihood"/>
                <discretizedBranchRates idref="branchRates"/>
                <discretizedBranchRates idref="branchRates"/>
                <speciationLikelihood idref="speciation"/>
           </log>

           <!-- write tree log to file
-->
           <logTree id="treeFileLog" logEvery="1000"
nexusFormat="true" fileName="beast-xml.(time).trees.txt"
sortTranslationTable="true">
                <treeModel idref="treeModel"/>
                <trait name="rate" tag="rate">
                     <discretizedBranchRates
idref="branchRates"/>
                </trait>
                <posterior idref="posterior"/>
           </logTree>
```

```
            <logTree id="substTreeFileLog" logEvery="1000"
nexusFormat="true" fileName="beast-xml.(subst).trees"
branchLengths="substitutions">
                  <treeModel idref="treeModel"/>
                  <discretizedBranchRates idref="branchRates"/>
                  <trait name="rate" tag="rate">
                        <discretizedBranchRates
idref="branchRates"/>
                  </trait>
            </logTree>
      </mcmc>
      <report>
            <property name="timer">
                  <mcmc idref="mcmc"/>
            </property>
      </report>
</beast>
```
