# Supplementary material for: Morphological Innovations and Vast Extensions of Mountain Habitats Triggered Rapid Diversification Within the Species-Rich Irano-Turanian Genus Acantholimon (Plumbaginaceae)
Source: Front Genet. 2019 Jan 21;9:698. doi: 10.3389/fgene.2018.00698 (PMC6360523; doi:10.3389/fgene.2018.00698)
Supplement: Data Sheet S3 — Maximum clade credibility (MCC) tree showing mean estimates and 95% high-posterior-density (HPD) credibility intervals obtained from the BEAST analysis of the molecular dataset comprising 130 species from Acantholimon s.l. and 26 outgroups taxa, using the two-calibration point strategy (see Table S1). The tree is the same as the one shown in Figure 1. [file Data_Sheet_3.pdf]

```
#NEXUS

[Maximum clade credibility (MCC) tree showing mean estimates and 95%
high-posterior-density (HPD) credibility intervals obtained from the
BEAST analysis of the molecular dataset comprising 130 species from
Acantholimon s.l. and 26 outgroups taxa, using the two-calibration point
strategy (see Table S1). The tree is the same as the one shown in Figure
1]

begin taxa;
	dimensions ntax=156;
	taxlabels
	AcaAraxanu
	AcaFlabeum
	Acanthob40
	Acmostegi1
	Alavaeaaaa
	Albocalyci
	Armeriamad
	Armeriamar
	Armeriasin
	Armeriaspe
	Armeriawel
	Armhirtaaa
	Aspadanumm
	Asphodelin
	Atropatanu
	AuganumW86
	Austroiran
	Avenaceumm
	Balohani75
	Bamianiaaa
	Blandummmm
	Bodeanum1m
	Brachystac
	Bracteatum
	Bromifoliu
	Bukcabu39
	Cabulicu99
	Carinatu25
	Caryophyll
	Cephalot39
	Cephaltoid
	Cephcoe32
	Cephturcoc
	ChaetolSet
	Chitrali79
	Chlorosteg
	Collareeee
	Cupreoliva
	Curviflor1
	Cymosummmm
	Demawendic
	Densiflor1
	Dicgrif30
	Dicmacr34
	DyerSoconu
	Eschkerese
```

Festucas88
Flexuosumm
Fominiiiii
Gilliatiii
Glabratum1
Glaspeci25
Gonispecoi
Gonitilcum
Gorganense
Gulistanum
Heratensee
Heweriiiii
Hohenacer1
Hormozgane
Horridummm
Hystrixxxx
Karelinii1
Kermanense
Latifolium
Limaxillar
Limcarnos1
Limgmelini
Limiranicm
Limmeyerii
Limnarbone
Limnudummm
Limotoleps
Limrenifoe
Limsogdiam
Limsuffruo
Limvulgare
Melananthu
Mirtajadin
Mischodage
Modestum1a
Moradiiiii
Nigricanse
Oliganthum
Olivieri1i
Ophiocladu
Plumeuropa
Psybeludsh
Psyleptosh
Psyspicata
Psysuworou
Pterostegi
Quniquelob
Raddeanumm
Restiacumm
Rodopolium
Rudbaricu1
Sacberllum
Sahendicu2
Schahrudic
Scirpinumm
Serotinumo
Shirazianu
Sirchensee

```
	Spinicaly1
	Takhtaj678
	Talagon103
	Tomentellu
	Tragacanth
	Vassizso44
	Zaefii1iii
	Zaprjaga24
	acerosum08
	ahangare92
	alatavic81
	armenum647
	aulieten67
	compact047
	diapens252
	ecae024754
	ekatheri80
	ekbergia42
	erinaceu70
	erythrae98
	fascicul45
	federov277
	gilliiW141
	glumace690
	glutinos37
	hypochar7h
	inermeM004
	knorri9KYG
	koeieiW153
	laxuW2055K
	leucant552
	leucochl50
	lycopoio55
	macrathu10
	nabievi887
	peculiar19
	physoste55
	pulchell79
	revolutu15
	sakeniW295
	sarytavi90
	schizost31
	scorpiu927
	senga85995
	solidum174
	stereop252
	subulat221
	tricol21AN
	ulicinum79
	venustum99
	wendORO10k
	wiedema346
;
end;

begin trees;
	tree tree_1 = [&R]
((((Psybeludsh:0.784215,(Psyleptosh:0.461462,(Psysuworou:0.17631,Psyspic
```

ata:0.17631):0.285152):0.322753):5.312776,(Armeriamar:0.364875,(Armeriawe
l:0.275779,((Armeriaspe:0.146516,Armeriamad:0.146516):0.062282,(Armhirtaa
a:0.02334,Armeriasin:0.02334):0.185458):0.066981):0.089096):5.732116):4.6
77934,((((Limrenifoe:0.940015,Limotoleps:0.940015):1.736721,(Limsogdiam:2
.186083,Limnudummm:2.186083):0.490652):1.017657,Limaxillar:3.694392):0.34
1855,(((Limnarbone:0.273282,(Limgmelini:0.197419,Limmeyerii:0.197419):0.0
75864):0.166575,Limvulgare:0.439857):1.268323,((Limsuffruo:0.273877,Limca
rnos1:0.273877):0.261777,Limiranicm:0.535654):1.172526):2.328066):6.73867
8):4.633749,((((ChaetolSet:0.686023,Vassizso44:0.686023):2.098718,((Bamia
niaaa:1.441661,(Cephcoe32:1.036762,Cephturcoc:1.036762):0.404899):0.77671
6,(((Zaefii1iii:0.554373,((((Nigricanse:0.082615,(Gilliatiii:0.04807,(Asp
adanumm:0.016258,Flexuosumm:0.016258):0.031812):0.034545):0.023993,Melana
nthu:0.106608):0.077324,((Festucas88:0.069462,(Ophiocladu:0.023864,senga8
5995:0.023864):0.045598):0.043555,Serotinumo:0.113017):0.070915):0.151892
,((Asphodelin:0.084266,(Hormozgane:0.018753,Eschkerese:0.018753):0.065513
):0.106546,((Sacberllum:0.026221,Moradiiiii:0.026221):0.080176,(Oliganthu
m:0.06759,Brachystac:0.06759):0.038806):0.084416):0.145012):0.218548):0.2
39045,((Bromifoliu:0.312591,(((Atropatanu:0.089736,Olivieri1i:0.089736):0
.050453,Hohenacer1:0.140189):0.097661,(acerosum08:0.078118,armenum647:0.0
78118):0.159732):0.074741):0.193951,((((Latifolium:0.075005,Bracteatum:0.
075005):0.114072,(Takhtaj678:0.082914,venustum99:0.082914):0.106163):0.04
4603,(Densiflor1:0.071149,Caryophyll:0.071149):0.16253):0.127986,AcaAraxa
nu:0.361665):0.144876):0.286876):0.291163,((diapens252:0.030496,ekbergia4
2:0.030496):0.578204,((Cephalot39:0.101074,compact047:0.101074):0.248848,
(Cephaltoid:0.061341,Demawendic:0.061341):0.288581):0.258778):0.475881):1
.133797):0.566364):0.64793,((((Pterostegi:0.376936,Cymosummmm:0.376936):0
.665839,((((((Karelinii1:0.077811,(Rudbaricu1:0.043055,wendORO10k:0.04305
5):0.034756):0.143592,((Hystrixxxx:0.042121,Rodopolium:0.042121):0.132926
,(scorpiu927:0.137105,Austroiran:0.137105):0.037941):0.046357):0.042476,T
ragacanth:0.263879):0.153607,(Quniquelob:0.150161,(Balohani75:0.059286,(S
pinicaly1:0.03066,Heratensee:0.03066):0.028626):0.090875):0.267325):0.070
276,Acmostegi1:0.487762):0.355769,ecae024754:0.843531):0.199244):0.197348
,((((((((glumace690:0.197359,(((Mischodage:0.022609,Fominiiiii:0.022609)
:0.059602,ulicinum79:0.082211):0.032803,alatavic81:0.115014):0.082345):0.
071683,(hypochar7h:0.114085,wiedema346:0.114085):0.154957):0.186693,((aul
ieten67:0.200788,ekatheri80:0.200788):0.088224,(((knorri9KYG:0.047668,nab
ievi887:0.047668):0.023092,sakeniW295:0.07076):0.09592,laxuW2055K:0.16668
):0.122332):0.166723):0.105478,(((inermeM004:0.237776,schizost31:0.237776
):0.080668,(Carinatu25:0.200966,(ahangare92:0.135077,stereop252:0.135077)
:0.065889):0.117479):0.112115,((koeieiW153:0.022909,Acanthob40:0.022909):
0.26226,(peculiar19:0.234405,(leucochl50:0.083059,AuganumW86:0.083059):0.
151347):0.050764):0.145391):0.130653):0.054311,((tricol21AN:0.081778,soli
dum174:0.081778):0.128837,physoste55:0.210614):0.404909):0.058304,(erinac
eu70:0.26895,Gulistanum:0.26895):0.404878):0.09447,(AcaFlabeum:0.227989,(
(((Raddeanumm:0.028527,Bodeanum1m:0.028527):0.035234,Scirpinumm:0.063761)
:0.060242,(Gorganense:0.063865,Avenaceumm:0.063865):0.060138):0.037628,Bl
andummmm:0.161631):0.066358):0.540309):0.050118,(((pulchell79:0.117217,sa
rytavi90:0.117217):0.17671,glutinos37:0.293927):0.040491,macrathu10:0.334
418):0.483998):0.104643,((((Collareeee:0.138679,Heweriiiii:0.138679):0.48
0337,(Sahendicu2:0.53524,(Glaspeci25:0.278425,(Restiacumm:0.104555,Alavae
aaaa:0.104555):0.17387):0.256815):0.083776):-
0.062089,((Zaprjaga24:0.026013,gilliiW141:0.026013):0.05839,Chitrali79:0.
084403):0.472524):0.093686,((((((Kermanense:0.014343,Albocalyci:0.014343)
:0.018068,Chlorosteg:0.032411):0.029278,Mirtajadin:0.061689):0.075794,Tom
entellu:0.137484):0.16566,(((Glabratum1:0.13911,(Curviflor1:0.054468,leuc
ant552:0.054468):0.084641):0.06761,(((federov277:0.073341,Shirazianu:0.07
3341):0.032672,Talagon103:0.106013):0.053971,(Cupreoliva:0.107793,(Horrid
ummm:0.088535,(Schahrudic:0.027623,Sirchensee:0.027623):0.060912):0.01925

```
7):0.052191):0.046736):0.038333,Modestum1a:0.245052):0.058092):0.087236,(
(subulat221:0.114619,(revolutu15:0.080967,erythrae98:0.080967):0.033652):
0.099991,((Cabulicu99:0.051566,fascicul45:0.051566):0.044186,lycopoio55:0
.095753):0.118856):0.175771):0.260234):0.272446):0.317063):1.284252,(Bukc
abu39:1.436868,(Dicgrif30:0.608668,Dicmacr34:0.608668):0.8282):1.087507):
0.908297):3.400472,(Gonitilcum:0.981293,Gonispecoi:0.981293):6.403851):7.
990053):17.782592,(DyerSoconu:8.555768,Plumeuropa:8.495768):24.705498);
end;

begin figtree;
	set appearance.backgroundColorAttribute="Default";
	set appearance.backgroundColour=#ffffff;
	set appearance.branchColorAttribute="User selection";
	set appearance.branchColorGradient=false;
	set appearance.branchLineWidth=1.0;
	set appearance.branchMinLineWidth=0.0;
	set appearance.branchWidthAttribute="Fixed";
	set appearance.foregroundColour=#000000;
	set appearance.hilightingGradient=false;
	set appearance.selectionColour=#2d3680;
	set branchLabels.colorAttribute="User selection";
	set branchLabels.displayAttribute="Branch times";
	set branchLabels.fontName="Agency FB";
	set branchLabels.fontSize=8;
	set branchLabels.fontStyle=0;
	set branchLabels.isShown=false;
	set branchLabels.significantDigits=4;
	set layout.expansion=0;
	set layout.layoutType="RECTILINEAR";
	set layout.zoom=0;
	set legend.attribute=null;
	set legend.fontSize=10.0;
	set legend.isShown=false;
	set legend.significantDigits=4;
	set nodeBars.barWidth=4.0;
	set nodeBars.displayAttribute=null;
	set nodeBars.isShown=false;
	set nodeLabels.colorAttribute="User selection";
	set nodeLabels.displayAttribute="Node ages";
	set nodeLabels.fontName="Agency FB";
	set nodeLabels.fontSize=8;
	set nodeLabels.fontStyle=0;
	set nodeLabels.isShown=true;
	set nodeLabels.significantDigits=4;
	set nodeShape.colourAttribute="User selection";
	set nodeShape.isShown=false;
	set nodeShape.minSize=10.0;
	set nodeShape.scaleType=Width;
	set nodeShape.shapeType=Circle;
	set nodeShape.size=4.0;
	set nodeShape.sizeAttribute="Fixed";
	set polarLayout.alignTipLabels=false;
	set polarLayout.angularRange=0;
	set polarLayout.rootAngle=0;
	set polarLayout.rootLength=100;
	set polarLayout.showRoot=true;
	set radialLayout.spread=0.0;
	set rectilinearLayout.alignTipLabels=false;
```

```
	set rectilinearLayout.curvature=0;
	set rectilinearLayout.rootLength=100;
	set scale.offsetAge=0.0;
	set scale.rootAge=1.0;
	set scale.scaleFactor=1.0;
	set scale.scaleRoot=false;
	set scaleAxis.automaticScale=true;
	set scaleAxis.fontSize=8.0;
	set scaleAxis.isShown=false;
	set scaleAxis.lineWidth=1.0;
	set scaleAxis.majorTicks=1.0;
	set scaleAxis.origin=0.0;
	set scaleAxis.reverseAxis=false;
	set scaleAxis.showGrid=true;
	set scaleBar.automaticScale=true;
	set scaleBar.fontSize=10.0;
	set scaleBar.isShown=true;
	set scaleBar.lineWidth=1.0;
	set scaleBar.scaleRange=0.0;
	set tipLabels.colorAttribute="User selection";
	set tipLabels.displayAttribute="Names";
	set tipLabels.fontName="Agency FB";
	set tipLabels.fontSize=8;
	set tipLabels.fontStyle=0;
	set tipLabels.isShown=true;
	set tipLabels.significantDigits=4;
	set trees.order=false;
	set trees.orderType="increasing";
	set trees.rooting=false;
	set trees.rootingType="User Selection";
	set trees.transform=false;
	set trees.transformType="cladogram";
end;
```
